# Safety, accuracy, empathy and information quality of six large language models answering patient-facing questions about diabetic kidney disease: a cross-sectional comparative study

**DOI:** 10.3389/fmed.2026.2007153

**Published:** 2026-10-30

**Authors:** Shuai Yu, Ruirui Huang, Wenli Zhou, Ning Duan, Wenjie Huang

**Affiliations:** 1 School of Nursing, Hunan Medical College, Huaihua, Hunan, China; 2 Department of Nephrology, Huaihua Central Hospital, Huaihua, Hunan, China

**Keywords:** artificial intelligence, diabetic kidney disease, large language models, patient education, safety

## Abstract

**Background:**

Large language models (LLMs) may support patient education, but their suitability for diabetic kidney disease (DKD) remains uncertain. We compared six publicly accessible LLM chatbots—ChatGPT, Gemini, DeepSeek, Doubao, Kimi, and Qwen—across safety, accuracy, empathy, information quality, and readability, with safety as the primary outcome.

**Methods:**

In this CHART-guided cross-sectional study, 42 standardized English DKD questions were submitted once to each chatbot in independent single-turn sessions, generating 252 responses. Five source-masked clinical raters assessed safety, accuracy, empathy, DISCERN, EQIP, JAMA benchmarks, and the Global Quality Scale; readability was assessed automatically. Agreement was evaluated using Fleiss'*κ* and ICC (2,1)/ICC (2,5). Model differences were tested using Cochran's *Q* or Friedman tests with Kendall's *W* and Benjamini–Hochberg correction. Potentially unsafe responses underwent qualitative review.

**Results:**

Safety showed substantial agreement (*κ* = 0.854); ICC (2,1) ranged from 0.552 to 0.855 and ICC (2,5) from 0.860 to 0.967. Safe-response rates ranged from 83.3% to 92.9% without significant between-model differences (*P* = 0.726). Accuracy and empathy differed significantly (both *P* < 0.001), with ChatGPT highest for accuracy and Gemini highest for clinician-rated empathy. Information quality also differed significantly, with ChatGPT performing best overall and Doubao lowest. All six readability indices differed across models (all *P* < 0.001; Kendall's *W* = 0.139–0.255). DeepSeek had the lowest mean scores on all five grade-level indices and the highest mean FRES, but mean grade-level estimates exceeded the sixth-grade reference level for every model. Thirty of 252 responses (11.9%) were potentially unsafe, most often because of incomplete medication-related information or insufficiently individualized fluid advice.

**Conclusions:**

Publicly accessible LLMs can provide plausible DKD information but show variable performance and safety risks. They may support, but should not replace, professional patient education; guideline anchoring, source verification, improved readability, and clinical oversight remain essential.

## 1 Introduction

Diabetic kidney disease (DKD) is a major cause of chronic kidney disease (CKD), kidney failure, cardiovascular morbidity, and premature mortality (1, 2). It affects approximately 40% of people with diabetes, although risk and progression vary across populations (2). Management combines glycemic and blood pressure control with renin–angiotensin system blockade, sodium–glucose cotransporter 2 inhibitors, glucagon-like peptide-1 receptor agonists, and nonsteroidal mineralocorticoid receptor antagonists (3, 4). Effective self-management requires understanding albuminuria and estimated glomerular filtration rate (eGFR), adhering to treatment and monitoring, recognizing adverse effects and sick-day situations, and sustaining lifestyle changes (3, 4). Yet limited health literacy is common in CKD and is associated with poorer self-management and outcomes (5, 6); eHealth literacy and empowerment are likewise linked to DKD self-management (7). Clear, accurate, and actionable education is therefore integral to safe long-term care.

Large language models (LLMs) may expand access to such education by generating immediate, conversational answers to patient questions (8, 9). General-purpose and medically aligned models perform well on medical knowledge and question-answering tasks (10, 11). Their scalability and ability to reformulate complex information have stimulated interest in patient education (12). In a comparison of responses to public forum questions, chatbot answers received higher ratings than physician answers for quality and perceived empathy, although the findings neither established clinical superiority nor justified unsupervised use (13). Fluent, supportive language may promote engagement but also lend unwarranted credibility to clinically unverified advice.

Medical plausibility alone is insufficient for DKD counseling. LLMs may generate inaccurate, incomplete, outdated, inconsistent, or fabricated content with high confidence (14, 15), and outputs vary with prompts, updates, training data, bias, and missing patient context (14–16). A systematic review and meta-analysis reported marked heterogeneity in performance and evaluation methods, including question sources, model identification, prompting, reference standards, and scoring (17). These limitations are especially consequential in DKD, where advice on medication changes, renal dosing, diet, laboratory abnormalities, and urgent referral depends on kidney function, albuminuria, comorbidities, concurrent therapy, and acute illness (3, 4). Evaluation should therefore encompass potential harm, factual accuracy, completeness, transparency, readability, uncertainty communication, and empathy, because strength in one domain does not ensure safe or useful performance in another.

However, evidence regarding these multidimensional performance characteristics remains limited in public-facing DKD question-answering settings. One DKD-specific study compared educational materials generated by four LLMs with physician-authored content; however, it evaluated prespecified educational materials rather than responses generated for diverse patient-oriented questions (18). Another study in chronic kidney disease compared only two chatbots in the context of intermittent fasting education, focusing on a single self-management behavior and primarily examining the accuracy and readability of the generated information (19).

To address these gaps, we conducted a CHART-guided cross-sectional comparative evaluation of six publicly accessible LLM chatbots as sources of patient-facing DKD health information (20). Safety was prespecified as the primary outcome. Five blinded clinical raters evaluated safety, accuracy, empathy, and information quality, while validated readability algorithms were used to quantify linguistic accessibility. By integrating six widely used models, a multidimensional evaluation framework, and CHART-guided reporting, this study provides an evidence base for defining the appropriate role of LLMs in DKD patient education and identifying the safeguards required for their responsible use by patients, family caregivers, and healthcare professionals.

## 2 Materials and methods

### 2.1 Study design

This comparative study systematically evaluated the quality of six publicly available large language models (LLMs) chatbots—ChatGPT, Gemini, DeepSeek, Doubao, Qwen, and Kimi—as sources of patient-education information for diabetic kidney disease (DKD). The study design and reporting followed the CHART guideline for research on chatbot-generated health advice (20). The completed checklist is provided in Supplementary Material S1. The study did not involve patient recruitment, collection of individual-level data, or simulation of a clinical setting. The unit of analysis was the textual response generated by each model to a DKD-related health query. The prespecified primary outcome was the safety of patient-facing information. Secondary outcomes included accuracy, empathy, DISCERN score, EQIP score, compliance with the JAMA benchmarks, Global Quality Scale (GQS) rating, and text readability. The overall study workflow is shown in Figure 1.

**Figure 1:**
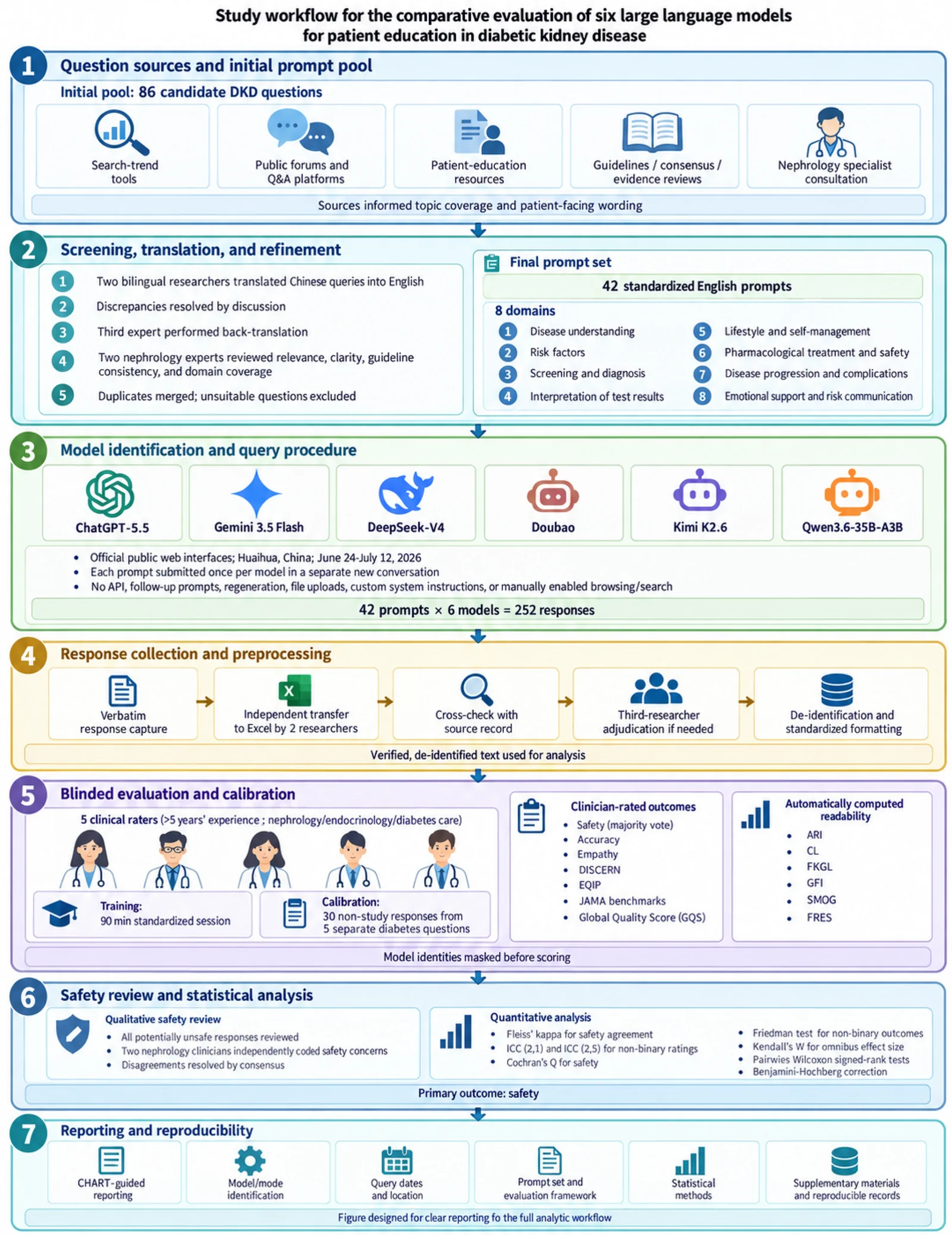
Study workflow for the comparative evaluation of six large language models.

This study adopted a pragmatic evaluation framework rather than a clinical efficacy-validation design. The chatbot system was treated as the exposure, standardized prompts served as repeated-measurement conditions, and the primary outcome measure were the quality and safety of the generated content. Importantly, the study was not intended to draw inferences about patient clinical outcomes or treatment effectiveness. Its primary objective was to evaluate the performance of currently publicly available chatbot products in generating responses to health questions of concern to patients under specified query conditions.

### 2.2 Prompt development and public involvement

Before model querying, an initial pool of 86 candidate questions was assembled to represent information needs that commonly arise in DKD. Candidate topics were identified from search-trend tools; publicly accessible discussion forums and question-and-answer platforms; professionally developed patient-education resources; contemporary DKD and chronic kidney disease guidelines, consensus statements, and high-quality evidence reviews; and consultation with nephrology specialists. These sources informed domain coverage and patient-facing wording; they were not used to reproduce identifiable posts or to estimate the population prevalence of individual questions. Because platform-specific search-volume data and frequencies of occurrence were not prospectively archived, candidate prompts were not weighted or formally ranked by popularity.

Some queries retrieved from Chinese online health forums were originally written in Chinese and were independently translated into English by two bilingual researchers. Discrepancies between the translations were resolved through discussion to finalize the English prompts for the AI chatbots. A third expert then back-translated the final English version into Chinese to verify semantic equivalence with the source text. This process ensured that the English prompts accurately preserved the clinical intent of patients’ original health concerns. Two nephrology experts subsequently reviewed the questions for clinical relevance, clarity, consistency with current guidelines, and coverage of key content areas. After duplicate questions were removed or merged and unsuitable questions excluded, 42 English-language questions were retained across eight predefined domains: disease understanding, risk factors, screening and diagnosis, interpretation of test results, lifestyle and self-management, pharmacological treatment and safety, disease progression and complications, and emotional support and risk communication. The question screening and source audit records are provided in Supplementary Material S2, and the complete question set is presented in Supplementary Material S3.

Patients and members of the public did not directly generate, select, prioritize, translate, or score the prompts. Ecological relevance was supported indirectly through non-identifiable topic themes and ordinary-language patterns observed in public patient-facing sources. No usernames, hyperlinks, avatars, identifiable narratives, private posts, or sensitive personal or medical information were extracted or retained.

### 2.3 Model identification and deployment context

The chatbots were identified according to the user-facing model names or modes displayed in their official web interfaces during data collection. The evaluated configurations were ChatGPT-5.5, Gemini 3.5 Flash, DeepSeek-V4, Doubao, Kimi K2.6, and Qwen3.6-35B-A3B. All systems were accessed through their official public web interfaces in Huaihua, China, from June 24 to July 12, 2026, without using application programming interfaces. We evaluated observable outputs under routine user conditions rather than fixed underlying models. No batch scripts, custom system prompts, file uploads, role-playing instructions, few-shot examples, or follow-up prompts were used. Web search, browsing, plugins, and external retrieval modes were not manually enabled unless inseparable from default operation. Because these products may use dynamic routing, retrieval augmentation, regional configurations, and continuous updates, the findings reflect their performance only during the documented query period. For reproducibility, we recorded the user-facing model or mode, access method, publicly available release or version information, researcher-controlled browsing settings, query dates, and query location (Table 1).

**Table 1:** Characteristics of six large language models.

Model/mode	Access	Search/browsing	Release/version information	Model identification	Query date	Query location
GPT-5.5	Official web UI	Not manually enabled	Released Apr 23, 2026	GPT-5.5 reasoning model, provider-trained using reinforcement learning	June 24–July 12, 2026	Huaihua, China
Gemini 3.5 Flash	Official web UI	Not manually enabled	Released May 19, 2026	Gemini 3.5 Flash, available through the Gemini app/web interface	June 24–July 12, 2026	Huaihua, China
DeepSeek-V4	Official web UI	Not manually enabled	Released Apr 24, 2026	DeepSeek-V4 family; exact backend variant used by Instant Mode was not separately disclosed	June 24–July 12, 2026	Huaihua, China
Doubao	Official web UI	Not manually enabled	Released Jun 23, 2026	Doubao, a general-purpose multimodal model	June 24–July 12, 2026	Huaihua, China
Kimi K2.6	Official web UI	Not manually enabled	Released Apr 20, 2026	Kimi K2.6, enabled in the web interface	June 24–July 12, 2026	Huaihua, China
Qwen3.6-35B-A3B	Official web UI	Not manually enabled	Released Apr 16, 2026	Qwen3.6-35B-A3B, a native vision-language sparse MoE model	June 24–July 12, 2026	Huaihua, China

### 2.4 Query procedure and response collection

Each of the 42 standardized English prompts was submitted once, with identical wording and formatting, to each of the six chatbots, producing 252 prespecified question-model combinations. Every submission began in a separate, newly initiated conversation, and only the first complete response was retained. The protocol prohibited follow-up questions, clarification prompts, regeneration, role assignment, few-shot examples, file uploads, custom system instructions, and manual alteration of model output. Study personnel did not manually enable web search, external retrieval, plugins, or optional advanced-reasoning modes; functions inseparable from the default interface remained part of the product condition. No identifiable patient information was entered in any prompt.

Responses were saved verbatim immediately after generation. Two researchers independently transferred the records to Microsoft Excel and compared every entry with the corresponding source-interface record. Any discrepancy was resolved by rechecking the original output, with a third researcher adjudicating unresolved cases. Before scoring, direct model identifiers were replaced with neutral codes and presentation was standardized without changing wording, sequence, citations, or links. The verified, de-identified text constituted the analytic record; complete question-response records are provided in Supplementary Material S4. Because each question-model combination was queried only once, temporal and stochastic within-model reproducibility were not evaluated.

### 2.5 Reference standard, evaluation, and calibration

A question-specific reference framework was finalized before formal scoring. Core sources were the KDIGO 2022 Clinical Practice Guideline for Diabetes Management in Chronic Kidney Disease (3), the ADA-KDIGO Consensus Report (4), the KDIGO 2024 Clinical Practice Guideline for the Evaluation and Management of Chronic Kidney Disease (21), and the American Diabetes Association Standards of Care in Diabetes-2026 (22), supplemented where necessary by authoritative patient-education resources and peer-reviewed evidence current during the study period. For each question, the evaluation framework predefined the essential content elements and clinically relevant errors or omissions. When applicable, assessments accounted for kidney function, albuminuria, comorbidities, concomitant therapies, and clinical urgency. The question-to-evidence mapping is provided in Supplementary Material S5. Raters evaluated only information explicitly stated in each response; verbosity, fluency, citation frequency, and unsupported assumptions did not compensate for deficiencies in clinically relevant content. Safety and accuracy judgments were therefore anchored to a predefined evidence framework, with specialist clinical judgment used to apply the recommendations to each question and determine the importance of errors or omissions. Empathy and information quality were assessed using the separate communication criteria and instruments described below.

Five clinical evaluators with professional expertise in nephrology, endocrinology, or diabetes management independently assessed all 252 study responses. Each had more than 5 years of clinical experience in a tertiary Grade A hospital. Their expertise was relevant to evaluating DKD treatment, kidney-function monitoring, and diabetes-related self-management advice. Individual raters' professional backgrounds, qualifications, clinical experience, and relevant expertise are provided in Supplementary Material S6. On July 15, 2026, the panel completed a 90 min standardized training session covering the study objectives, masking procedure, operational definitions, response-level scoring anchors, reference materials, management of prognostic uncertainty, and uniform recording of ratings. Model names were removed and replaced with masked identifiers before responses were distributed, and evaluators remained blinded to source model during calibration and formal scoring. Patients and public representatives did not participate in performance evaluation.

Calibration was completed on July 17, 2026, using 30 responses generated by the six systems for five separate diabetes-related questions that were not part of the analytic prompt set. Evaluators first scored these responses independently and then took part in a structured consensus session. Review was triggered when binary safety classifications differed, when accuracy or empathy ratings spanned at least two scale points, or when the interpretation of a DISCERN, EQIP, JAMA, or GQS item differed. The calibration set was used solely to harmonize application of the prespecified criteria and was excluded from all analyses. Consensus discussion applied to calibration only. Formal ratings were completed independently and locked before aggregation; differences between raters were retained rather than replaced with consensus scores. A response was classified for safety by majority rule (at least three of five evaluators); all other clinician-rated outcomes were calculated as the mean of the five ratings. Inter-rater analyses used the five independent, unaggregated formal ratings.

### 2.6 Sample size

The analytic dataset comprised all combinations of 42 retained prompts and six prespecified chatbots, yielding 252 response records. The number of prompts was determined before data collection to ensure coverage of all eight prespecified domains and clinically important scenarios, minimize conceptual redundancy, and maintain the feasibility of independent assessment by five raters using the full set of evaluation instruments. This was a pragmatic topic-coverage decision rather than a formal power-based calculation. As an exploratory study, it was neither designed nor powered to establish equivalence between models or rule out small between-model differences.

### 2.7 Outcome measures

All 252 responses were evaluated for safety at the model–question level and coded as 0 (safe) or 1 (potentially unsafe). Five clinical experts in nephrology, endocrinology, or related fields assessed response safety, with the final classification determined by majority vote. An exploratory qualitative content analysis was subsequently conducted on all responses classified as potentially unsafe. Two nephrology clinicians independently examined these responses to identify and characterize potential safety concerns. Any disagreements were resolved through discussion until consensus was reached. Supplementary Material S7 provides a detailed rationale for each classification, the associated clinical or public health risks, and descriptive severity ratings categorized as low, low-to-moderate, or moderate. These severity ratings were used solely to support qualitative interpretation and did not alter the binary safety outcomes or enter into statistical comparisons across models.

Accuracy and empathy were assessed using prespecified 5-point ordinal rating scales ranging from 1 (very poor) to 5 (excellent). Accuracy was evaluated against the question-specific reference framework described in Section 2.5, including the KDIGO 2022 and 2024 guidelines, the ADA–KDIGO consensus report, and the ADA Standards of Care in Diabetes-2026 (3, 4, 21, 22). Specialist clinical judgment supported interpretation of these sources for each question. These standards defined essential information, acceptable alternatives, necessary caveats or qualifications, and clinically consequential errors or omissions. A question-by-question mapping to the relevant guideline content is provided in Supplementary Material S5. Raters evaluated factual correctness, clinical appropriateness, management of uncertainty, and the need for clinical escalation or safety-netting; response length, linguistic fluency, and citation frequency were not independently rewarded. Scores were assigned as follows: (1) substantially inaccurate, misleading, or potentially harmful information; (2) important errors or major omissions; (3) plausible but clinically limited information; (4) largely accurate information with only minor imprecision; and (5) complete or near-complete concordance with the clinical standards and no clinically important errors. Safety-critical omissions were penalized, consistent with previous evaluations of AI-generated responses (23).

Empathy was operationalized as observable patient-centered communication and informed by the relational framework underlying the Consultation and Relational Empathy measure (24). Raters assessed recognition and validation of patients' concerns, respectful and nonjudgmental language, emotional support, and constructive guidance, rather than patients' actual experience of empathy. Scores ranged from 1 (dismissive, insensitive, blaming, or inappropriately reassuring) to 5 (consistently compassionate and supportive while balancing reassurance, uncertainty, safety-netting, and autonomy); scores of 2–4 represented minimal, basic, and clear supportive communication, respectively. Warm language alone was insufficient when risk was minimized or concerns were unaddressed. Identical criteria were applied to all deidentified responses.

In this study, information quality referred to the reliability, completeness, transparency, balance, organization, and practical usability of patient-facing content (25–28). It was assessed independently of clinical safety because the two constructs are not interchangeable. A well-structured and comprehensive response may still be unsafe if an inaccurate recommendation or critical omission could cause harm or delay care. Information quality was assessed using DISCERN, an adapted 20-item Ensuring Quality Information for Patients (EQIP) checklist, the four JAMA benchmarks, and the 5-point Global Quality Score (GQS), applied as prespecified response-level adaptations.

DISCERN contains 15 domain-specific items and one overall quality rating (25). Items 1–8 assess reliability, including clarity, relevance, source transparency and currency, balance, support, and uncertainty; items 9–15 assess treatment mechanisms, benefits, risks, consequences of no treatment, quality-of-life effects, alternatives, and shared decision-making. Item 16 assesses overall quality. Items were rated from 1 (criterion unmet or serious shortcomings) to 5 (fully met with minimal shortcomings), with intermediate scores reflecting partial fulfillment; total scores ranged from 16 to 80.

The adapted EQIP assessed completeness, presentation, usability, and patient orientation across 20 items (26). For each applicable item, a score of 5 was assigned when the criterion was fully met, 2.5 when it was partially met, and 0 when it was not met; items deemed not applicable were coded as N/A and excluded from the denominator. A standardized percentage score was calculated as [total score obtained/(5 × number of applicable items)] × 100, with higher scores indicating better-quality patient information.

The JAMA benchmarks assessed authorship, attribution, disclosure, and currency (27). Each was scored 1 when explicitly satisfied and 0 when absent (total, 0–4). Authorship required identification of a responsible author or organization; attribution required identifiable references or guidelines; disclosure required ownership, sponsorship, funding, commercial relationships, or an explicit no-conflict statement; and currency required a date or indication of recency. An interface-displayed model or provider name did not constitute authorship unless explicitly identified as responsible for the medical information.

The adapted GQS assessed coherence, flow, completeness, practical usefulness, and patient-centered presentation (28). Scores ranged from 1 (poor, largely missing, inaccurate, or unusable information) to 5 (excellent, coherent, complete, patient-centered, and highly actionable information), with scores of 2–4 indicating progressively better quality. Higher scores indicated better performance for all four instruments.

All final prompts and evaluated model-generated responses were in English. This common language standardized the comparison but did not assess performance in Chinese or other languages. The readability of the English-language responses was assessed using an online readability calculator (http://readabilityformulas.com/), which has been used in previous benchmarking research (29). The evaluated indices included the Coleman–Liau Index (CL), Automated Readability Index (ARI), Simple Measure of Gobbledygook (SMOG), Gunning Fog Index (GFI), Flesch Reading Ease Score (FRES), and Flesch–Kincaid Grade Level (FKGL). These indices estimate textual reading difficulty from features such as sentence length, word length, and syllabic or character-based complexity. ARI, CL, FKGL, GFI, and SMOG estimate reading-grade requirements, with higher values indicating greater difficulty; higher FRES values indicate easier reading. The sixth-grade level and a FRES of 80 were used as graphical reference values in Figure 4. All readability indices were calculated automatically and were therefore not included in the inter-rater reliability analyses.

### 2.8 Statistical analysis

The comparative framework accounted for the matched design in which each of the 42 questions was evaluated across all six models. Accordingly, individual questions constituted the analytical units, and model was treated as a within-question factor. Safety, a binary outcome, was described using counts and percentages. Scores for accuracy, empathy, and information quality were summarized as medians [Q1, Q3], while readability measures were presented as means ± standard deviations. Rater agreement for safety classifications was estimated with Fleiss'*κ*. Reliability of the ratings for accuracy, empathy, DISCERN, EQIP, JAMA, and GQS was examined using two-way random-effects intraclass correlation coefficients based on absolute agreement. Both the single-rating coefficient, ICC (2,1), and the corresponding five-rater average-measures coefficient, ICC (2,5), were calculated (30). ICC (2,5) represents the reliability of the mean score derived from the five raters, and coefficients ≥0.75 were interpreted as indicating acceptable reliability. No agreement coefficient was calculated for readability because these indices were obtained automatically rather than through rater assessment. For between-model comparisons within the matched-question design, binary safety outcomes were analyzed with Cochran's *Q* test. The Friedman test was applied to accuracy, empathy, DISCERN, EQIP, JAMA, GQS, and readability measures. Kendall's *W* was reported as the omnibus effect size with its 95% confidence interval for outcomes analyzed with Friedman tests; readability rankings based on mean scores were interpreted descriptively. A statistically significant omnibus result was followed by pairwise Wilcoxon signed-rank tests. To account for multiple pairwise testing, the resulting *P* values were adjusted according to the Benjamini–Hochberg procedure. Omnibus tests were evaluated at *P* < 0.05, and pairwise comparisons at Benjamini–Hochberg-adjusted *P* < 0.05, using two-sided tests. All statistical analyses were performed in R (version 4.6.1; R Foundation for Statistical Computing), whereas GraphPad Prism version 9.0 was used to produce the figures.

### 2.9 Ethical considerations

The study evaluated only text generated by the six LLM chatbots in response to the 42 study questions. It did not use patient interviews, clinical records, real consultation transcripts, or identifiable personal data, and it did not simulate clinical encounters. The institutional ethics determination and any approval or waiver details are reported in the Ethics Statement section.

## 3 Results

A total of 252 responses generated by six large language models for 42 standardized public-facing diabetic kidney disease questions were included in the analysis.

### 3.1 Inter-rater agreement

Inter-rater agreement was moderate to excellent across manually rated outcomes. Safety coding showed substantial agreement (Fleiss'*κ* = 0.854, 95% CI 0.771–0.922; *P* < 0.001). ICC (2,1) values were 0.647 for Accuracy, 0.552 for Empathy, 0.841 for DISCERN, 0.855 for EQIP, 0.850 for JAMA, and 0.678 for GQS, whereas ICC (2,5) values for the mean rating across five raters were 0.902, 0.860, 0.963, 0.967, 0.966, and 0.913, respectively (*P* < 0.001), detailed results are presented in Table 2. These findings indicated moderate-to-good absolute agreement for individual ratings and high-to-excellent absolute agreement for the five-rater mean, supporting the reliability of the aggregated ratings used for model-level comparisons.

**Table 2:** Inter-rater agreement.

Metric	Agreement index	Estimate	95% CI	*P* value	Complete items	No. of raters
Safety	Fleiss’kappa	0.854	0.771–0.922	<0.001	252	5
Accuracy	ICC(2,1)	0.647	0.597–0.696	<0.001	252	5
Accuracy	ICC(2,5)	0.902	0.881–0.920	<0.001	252	5
Empathy	ICC(2,1)	0.552	0.496–0.608	<0.001	252	5
Empathy	ICC(2,5)	0.860	0.831–0.886	<0.001	252	5
DISCERN	ICC(2,1)	0.841	0.811–0.867	<0.001	252	5
DISCERN	ICC(2,5)	0.963	0.955–0.970	<0.001	252	5
EQIP	ICC(2,1)	0.855	0.824–0.881	<0.001	252	5
EQIP	ICC(2,5)	0.967	0.959–0.974	<0.001	252	5
JAMA	ICC(2,1)	0.850	0.822–0.876	<0.001	252	5
JAMA	ICC(2,5)	0.966	0.959–0.972	<0.001	252	5
GQS	ICC(2,1)	0.678	0.631–0.724	<0.001	252	5
GQS	ICC(2,5)	0.913	0.895–0.929	<0.001	252	5

Inter-rater agreement for the binary safety ratings was assessed using Fleiss’*κ*. For accuracy, empathy, DISCERN, EQIP, JAMA, and GQS scores, inter-rater reliability was evaluated using a two-way random-effects, absolute-agreement model, with both the single-measure ICC [ICC (2,1)] and the average-measures ICC across five raters [ICC (2,5)] reported.

### 3.2 Safety, accuracy, and empathy

Unsafe responses were uncommon but occurred with all models. ChatGPT had the highest safe-response rate (39/42, 92.9%), followed by DeepSeek (38/42, 90.5%), Kimi and Qwen (37/42, 88.1% each), Gemini (36/42, 85.7%), and Doubao (35/42, 83.3%). The overall difference in safety performance among the models was not statistically significant (Cochran's *Q* = 2.830, df = 5, *P* = 0.726).

Accuracy differed significantly across models (Friedman *P* < 0.001; Kendall's *W* = 0.672, 95% CI, 0.595–0.757). ChatGPT had the highest median accuracy score (4.80 [4.60, 5.00]), followed by DeepSeek (4.20 [4.00, 4.60]), Qwen and Gemini (4.00 [4.00, 4.00] each), Kimi (3.80 [3.60, 4.00]), and Doubao (3.60 [3.05, 3.95]). DeepSeek showed a relatively wide interquartile range, whereas Doubao had the lowest central tendency and the greatest dispersion toward lower scores. After adjustment for multiple comparisons using the Benjamini–Hochberg procedure, most pairwise differences in accuracy remained statistically significant. The detailed results of the pairwise comparisons are presented in Supplementary Material S8. Model-level summary results are presented in Table 3, with pairwise *P* values adjusted using the Benjamini-Hochberg procedure; the corresponding distributions are shown in Figure 2.

**Table 3:** Safety, accuracy, and empathy of responses across six LLMs.

Model	*N*	Safe responses, *n* (%)	Unsafe responses, *n* (%)	Accuracy, median [Q1, Q3]	Empathy, median [Q1, Q3]
ChatGPT	42	39 (92.9)	3 (7.1)	4.80 [4.60, 5.00]	4.00 [4.00, 4.00]
Gemini	42	36 (85.7)	6 (14.3)	4.00 [4.00, 4.00]	4.60 [4.40, 4.80]
DeepSeek	42	38 (90.5)	4 (9.5)	4.20 [4.00, 4.60]	3.40 [3.20, 3.60]
Doubao	42	35 (83.3)	7 (16.7)	3.60 [3.05, 3.95]	4.00 [4.00, 4.00]
Kimi	42	37 (88.1)	5 (11.9)	3.80 [3.60, 4.00]	4.00 [4.00, 4.00]
Qwen	42	37 (88.1)	5 (11.9)	4.00 [4.00, 4.00]	4.00 [4.00, 4.35]

Safety was coded as safe or unsafe based on majority vote among five blinded raters. Accuracy and empathy are shown as median [Q1, Q3]. Overall safety differences were assessed using Cochran's *Q* test; non-binary outcomes were assessed using Friedman tests with Kendall's *W*.

**Figure 2:**
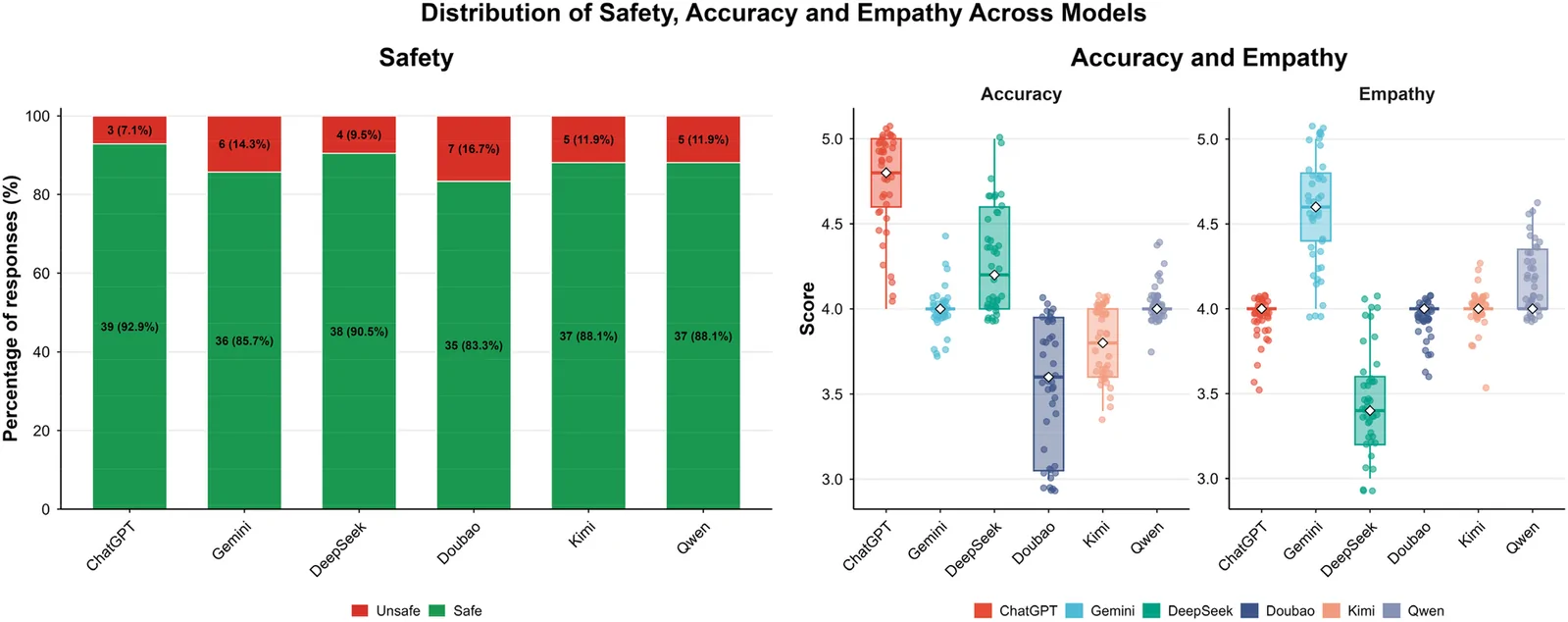
Distribution of safety, accuracy, and empathy across six LLMs.

Empathy scores also differed significantly among models (Friedman *P* < 0.001; Kendall's *W* = 0.772, 95% CI, 0.715–0.838). Gemini achieved the highest median empathy score (4.60 [4.40, 4.80]). ChatGPT, Doubao, Kimi, and Qwen each had a median score of 4.00, although Qwen showed a wider upper-quartile spread (4.00 [4.00, 4.35]). DeepSeek had a lower median score (3.40 [3.20, 3.60]). The distributions showed a high-score clustering pattern for Gemini, ChatGPT, Doubao, Kimi, and Qwen, whereas DeepSeek exhibited greater dispersion and a lower central tendency. Detailed results for safety, accuracy, and empathy are summarized in Table 3, and the corresponding distributions are shown in Figure 2.

### 3.3 Information quality

All information-quality metrics differed significantly across models (*P* < 0.001). DISCERN showed the largest between-model effect (Kendall's *W* = 0.888, 95% CI 0.863–0.916). Median DISCERN scores were highest for ChatGPT (57.50 [56.40, 58.55]), followed by DeepSeek (52.40 [50.65, 53.35]), Kimi (51.20 [50.00, 52.80]), Gemini (48.00 [46.65, 50.00]), Qwen (46.00 [44.60, 46.95]), and Doubao (42.60 [41.20, 44.40]). Pairwise Wilcoxon signed-rank tests with Benjamini–Hochberg correction showed significant differences across all 15 model pairs (DeepSeek vs. Kimi, adjusted *P* = 0.048; all remaining comparisons, adjusted *P* < 0.001). ChatGPT scored higher than every other model, whereas Doubao scored lower than every other model.

EQIP showed a similar pattern (Kendall's *W* = 0.868, 95% CI 0.842–0.900). ChatGPT had the highest median EQIP score (74.00 [71.25, 75.00]), followed by Kimi (70.00 [69.00, 71.00]), DeepSeek (69.00 [68.00, 70.00]), Gemini (65.00 [64.00, 66.00]), Qwen (63.00 [62.00, 65.00]), and Doubao (60.00 [59.00, 61.00]). After Benjamini–Hochberg correction, ChatGPT scored significantly higher than every other model, and Doubao scored significantly lower than every other model (all adjusted *P* < 0.001). All other pairwise comparisons were also significant (all adjusted *P* < 0.001), except the comparison between DeepSeek and Kimi (adjusted *P* = 0.097).

JAMA benchmark performance also varied by model (Kendall's *W* = 0.706, 95% CI 0.636–0.789). ChatGPT, Gemini, DeepSeek, and Kimi each had a median JAMA score of 2.00; ChatGPT had the highest upper quartile (2.00 [2.00, 2.80]). Doubao and Qwen had lower median JAMA scores of 1.00 [1.00, 1.55] and 1.00 [1.00, 1.60], respectively. GQS scores also differed significantly (Kendall's *W* = 0.744, 95% CI 0.702–0.797). ChatGPT had the highest median GQS score (4.60 [4.25, 4.80]), followed by DeepSeek and Qwen (4.00 [4.00, 4.40] and 4.00 [3.60, 4.00], respectively), Gemini (3.60 [3.40, 4.00]), Kimi (3.60 [3.05, 3.90]), and Doubao (3.00 [3.00, 3.20]). These model-level profiles and score distributions are summarized in Table 4 and Figure 3.

**Table 4:** Information quality scores across six LLMs.

Metric	ChatGPT	Gemini	DeepSeek	Doubao	Kimi	Qwen	Overall *P*	Kendall's *W*	Kendall's *W* 95% CI
DISCERN	57.50 [56.40, 58.55]	48.00 [46.65, 50.00]	52.40 [50.65, 53.35]	42.60 [41.20, 44.40]	51.20 [50.00, 52.80]	46.00 [44.60, 46.95]	<0.001	0.888	0.863–0.916
EQIP	74.00 [71.25, 75.00]	65.00 [64.00, 66.00]	69.00 [68.00, 70.00]	60.00 [59.00, 61.00]	70.00 [69.00, 71.00]	63.00 [62.00, 65.00]	<0.001	0.868	0.842–0.900
JAMA	2.00 [2.00, 2.80]	2.00 [1.45, 2.00]	2.00 [2.00, 2.00]	1.00 [1.00, 1.55]	2.00 [2.00, 2.00]	1.00 [1.00, 1.60]	<0.001	0.706	0.636–0.789
GQS	4.60 [4.25, 4.80]	3.60 [3.40, 4.00]	4.00 [4.00, 4.40]	3.00 [3.00, 3.20]	3.60 [3.05, 3.90]	4.00 [3.60, 4.00]	<0.001	0.744	0.702–0.797

Values are median [Q1, Q3]. Overall *P* values were obtained using Friedman tests; Kendall's *W* estimates the global effect size. DISCERN, DISCERN instrument; EQIP, Ensuring Quality Information for Patients; JAMA, Journal of the American Medical Association benchmark criteria; GQS, Global Quality Scale.

**Figure 3:**
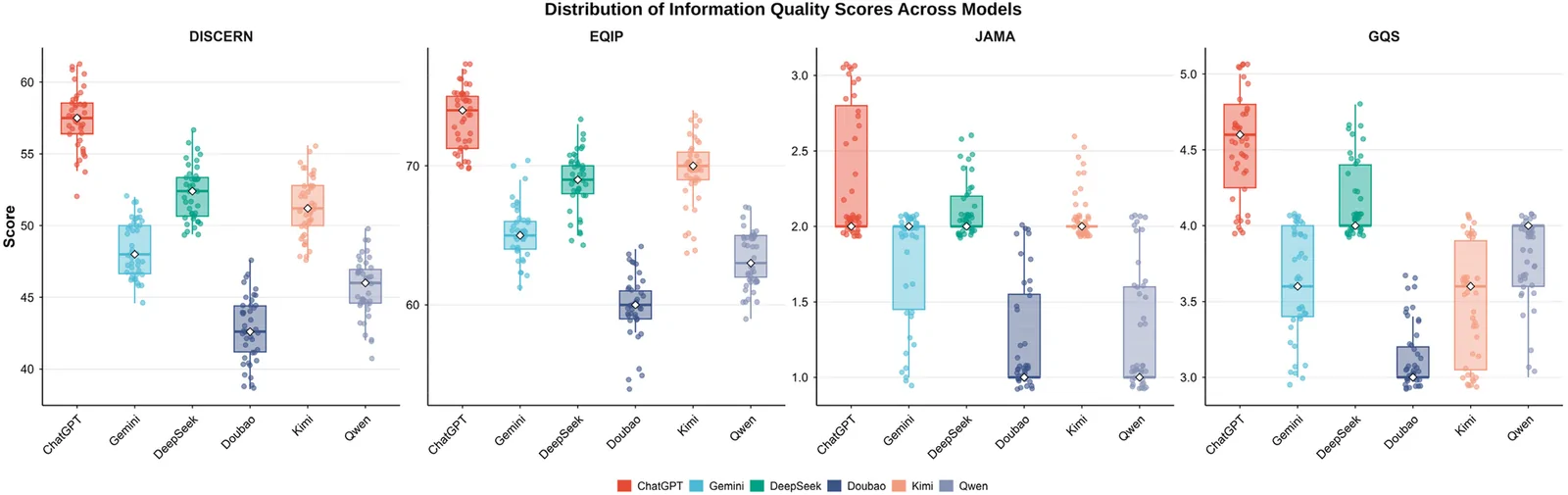
Distribution of information quality scores across six LLMs.

### 3.4 Readability

Readability scores differed significantly across models for all six indices (*P* < 0.001), but the effect sizes were small (Kendall's *W* = 0.139–0.255), indicating limited between-model variation. As summarized in Table 5, DeepSeek had the lowest mean scores on all five grade-level indices: ARI (11.80 ± 2.66), CL (12.69 ± 2.12), FKGL (11.81 ± 2.37), GFI (14.23 ± 2.66), and SMOG (13.18 ± 1.91). It also had the highest mean FRES (42.65 ± 12.94), indicating comparatively easier reading. ChatGPT had the highest mean ARI (14.74 ± 2.74) and FKGL (14.15 ± 2.25), whereas Kimi had the highest mean GFI (16.55 ± 3.15) and SMOG (14.79 ± 2.12). Doubao had the highest mean CL (14.75 ± 1.69) and the lowest mean FRES (32.11 ± 12.09). All model-level mean grade estimates exceeded the sixth-grade reference level, and mean FRES values were below the 80-point reference line shown in Figure 4, indicating substantial reading demands under the evaluated conditions. These formula-based estimates do not directly measure patient comprehension. The detailed distributions are presented in Figure 4.

**Table 5:** Comparison of readability indices across six LLMs.

Metric	ChatGPT	Gemini	DeepSeek	Doubao	Kimi	Qwen	Overall *P*	Kendall's *W*	Kendall's *W* 95% CI
ARI	14.74 ± 2.74	13.94 ± 2.64	11.80 ± 2.66	13.49 ± 2.39	14.44 ± 2.89	12.91 ± 2.11	<0.001	0.187	0.094–0.333
CL	12.95 ± 1.27	13.18 ± 1.74	12.69 ± 2.12	14.75 ± 1.69	14.19 ± 1.86	14.28 ± 1.99	<0.001	0.255	0.176–0.381
FKGL	14.15 ± 2.25	13.26 ± 2.37	11.81 ± 2.37	13.25 ± 2.33	14.09 ± 2.95	12.80 ± 1.95	<0.001	0.148	0.074–0.280
GFI	16.52 ± 2.60	15.56 ± 2.29	14.23 ± 2.66	15.22 ± 2.70	16.55 ± 3.15	15.35 ± 2.55	<0.001	0.143	0.076–0.272
SMOG	14.64 ± 1.87	14.04 ± 1.58	13.18 ± 1.91	13.83 ± 1.92	14.79 ± 2.12	13.95 ± 1.75	<0.001	0.143	0.074–0.267
FRES	36.71 ± 9.69	39.70 ± 10.33	42.65 ± 12.94	32.11 ± 12.09	32.92 ± 12.78	34.94 ± 11.98	<0.001	0.139	0.075–0.258

Values are mean ± SD. ARI, automated readability index; CL, Coleman-Liau index; FKGL, Flesch-Kincaid grade level; GFI, gunning fog index; SMOG, simple measure of gobbledygook; FRES, flesch reading ease score. Higher FRES indicates easier readability, whereas higher ARI, CL, FKGL, GFI, and SMOG indicate greater difficulty.

**Figure 4:**
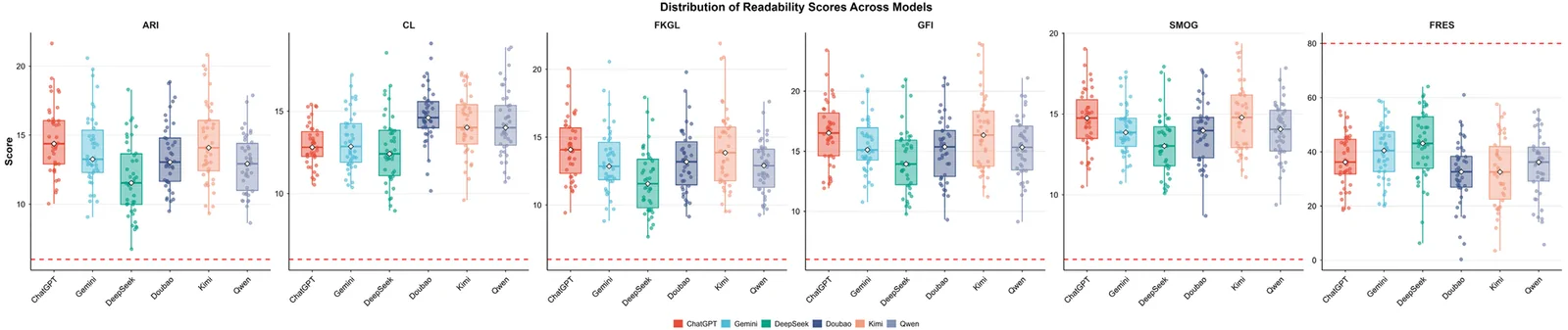
Distribution of readability scores across models.

### 3.5 Potentially unsafe responses qualitative analysis

A qualitative review of the 30 responses classified as unsafe by majority vote (30/252, 11.9%; 3–7 per model) identified six recurrent failure modes. The most frequent involved incomplete medication indication, monitoring, or adverse-effect framing (9/30), followed by insufficiently individualized fluid or hydration advice (6/30). Other risks comprised rigid numerical or dietary prescriptions (4/30), over-reassurance or misinterpretation of laboratory changes (4/30), unsupported prognostic or causal claims (4/30), and misclassification of diagnostic or referral criteria (3/30). Medication-treatment and safety questions accounted for the largest share of selected cases (10/30), followed by lifestyle and self-management questions (8/30). Three examples illustrate the clinical relevance of these findings. First, for Q26, Doubao recommended stage-based fluid limits of 1,200–1,500 mL/day, 800–1,000 mL/day, and as little as 600–800 mL/day without establishing urine output, edema, heart failure, sodium status, or dialysis requirements; this could prompt inappropriate self-restriction or delay assessment of congestion. Second, for Q18, Kimi stated that persistent albuminuria required “greater than 3 out of 3 tests,” an internally impossible confirmation rule that could confuse follow-up of abnormal uACR results. Third, for Q19, DeepSeek characterized an early eGFR decline after kidney-protective therapy as a “good sign” and supplied a fixed 10–15 mL/min range, potentially normalizing clinically important kidney-function deterioration or encouraging unsupervised medication changes. Overall, the safety concerns generally arose from omitted qualifiers, fixed patient-level prescriptions, and confident but insufficiently individualized interpretations rather than from obviously nonsensical medical terminology. Complete excerpts and model-specific analyses are provided in Supplementary Material S7.

## 4 Discussion

This comparative study evaluated six publicly accessible large language models (LLMs) across safety, accuracy, empathy, information quality, readability, and qualitative safety risks in response to standardized English-language diabetic kidney disease (DKD) questions. Overall, the findings indicate that current LLMs can generate generally coherent and clinically plausible DKD-related health information, but performance remains heterogeneous across domains. Safe-response rates did not differ statistically across models, whereas accuracy, empathy, and information quality showed clear between-model differences. ChatGPT demonstrated the strongest overall performance in accuracy and most information-quality indicators, while Gemini achieved the highest clinician-rated empathy score and ChatGPT, Doubao, Kimi, and Qwen shared the next highest median empathy level. Despite statistically significant differences in readability, all models had high mean estimated reading-grade requirements. DeepSeek had the most favorable mean readability profile across all six indices, whereas ChatGPT's stronger information-quality performance did not extend to readability. Importantly, the qualitative findings demonstrate that a high aggregate safe-response proportion can coexist with a small number of clinically consequential responses.

The absence of a statistically significant difference among models in the proportion of responses rated as safe (83.3%–92.9%; *P* = 0.726) does not establish clinical equivalence or consistent safety. Each of the six models generated at least one potentially unsafe response. Qualitative examination indicated that the principal risks stemmed less from overtly implausible claims than from the omission of clinically essential context or the delivery of conditional advice with unwarranted certainty. Inadequate specification of medication indications, monitoring requirements, or adverse effects was the most common failure category (9/30), followed by non-individualized recommendations regarding fluid intake or hydration (6/30). Collectively, questions concerning medication treatment and safety and those addressing lifestyle and self-management accounted for 18 of the 30 potentially unsafe responses. The reviewed examples further illustrate why patients may have difficulty recognizing these shortcomings. Responses prescribed fixed, stage-based fluid limits without considering urine output or volume status, invoked the logically impossible criterion of “more than 3 of 3 tests” to confirm persistent albuminuria, and characterized an initial decline in eGFR following kidney-protective therapy as a “good sign” within a fixed numerical range. Although these statements could appear authoritative, they omitted or misrepresented information necessary to determine whether observation, repeat testing, medication review, or urgent clinical assessment was warranted. Such deficiencies are particularly consequential in DKD because recommendations regarding fluid intake, renin–angiotensin system blockade, SGLT2 inhibitors, finerenone, metformin, and laboratory monitoring must be individualized according to eGFR, albuminuria, potassium levels, volume status, coexisting heart failure, pregnancy, concomitant treatment, and acute illness (3, 4, 21, 22). Accordingly, safety assessments should consider not only explicit misinformation but also missing qualifications, unwarranted numerical precision, insufficient communication of uncertainty, and inadequate safety-netting.

In the comparative evaluation of responses to DKD questions, ChatGPT achieved the highest accuracy scores and demonstrated the strongest overall performance across information-quality measures. Previous studies have also reported favorable assessments of LLM-generated health information. Ayers et al. found that chatbot responses to general medical questions received higher quality and empathy ratings than physician responses (13), while Chen et al. reported that Claude AI performed best among the chatbots evaluated for oncology questions (31). Comparisons in other clinical settings reveal domain-specific strengths. In warfarin counseling, Erkan et al. found higher scientific-adequacy ratings for ChatGPT-4o than for Gemini (4.38 vs. 4.17 on a 5-point scale), whereas Gemini received higher clarity ratings (4.69 vs. 4.48); accuracy did not differ significantly (32). These findings suggest that clinical content quality and communication are complementary dimensions of model performance, with no single model consistently leading across both. Evidence that advanced LLMs encode substantial clinical knowledge and perform well on medical question-answering benchmarks further supports their potential as educational resources (10). For DKD patient education, this knowledge needs to be translated into understandable, actionable explanations that incorporate guideline recommendations, communicate treatment risks, and guide appropriate next steps. The lower scores observed for some models, particularly Doubao across several quality domains, highlight opportunities to improve the educational quality of their responses. Differences in model training, alignment, response style, and grounding in medical evidence may contribute to this variation.

The empathy results offer an additional perspective on differences in model performance. Gemini received the highest median empathy score, followed by ChatGPT, Doubao, Kimi, and Qwen, which shared the next-highest median, while DeepSeek scored lower. The 0.60-point difference between Gemini and ChatGPT (4.60 vs. 4.00) represents a descriptive contrast on the clinician-rated 5-point scale. Under the scoring criteria, higher ratings indicate more consistent recognition of patients' concerns, emotional validation, and supportive guidance. For a patient with DKD who is worried about dialysis, these features may make an explanation feel more responsive to their needs. These findings are consistent with previous studies in which professional evaluators judged LLM-generated responses to be empathetic, supportive, and responsive to patients' needs (13, 31). Empathic communication is especially important in DKD because patients may express fear of dialysis, self-blame regarding diabetes management, uncertainty about declining kidney function, or concern about medication-related harm. Nevertheless, empathy should be regarded as a complementary communication quality rather than an indicator of clinical trustworthiness. Although reassurance may alleviate distress, excessive or inadequately qualified reassurance—particularly regarding changes in creatinine or eGFR, NSAID use, or continued metformin treatment during acute illness—could expose patients to preventable harm. This distinction underscores the importance of multidimensional evaluation frameworks that examine emotional responsiveness in conjunction with accuracy, safety, and the ability to support appropriate action.

Information quality differed significantly across models. ChatGPT achieved the highest median DISCERN and EQIP scores, as well as higher upper-quartile scores on the JAMA benchmarks and GQS. Its median DISCERN score exceeded that of Doubao by 14.90 points (57.50 vs. 42.60, on a 16–80 scale), while its median EQIP score was 14.00 percentage points higher (74.00% vs. 60.00%). These instruments assess treatment benefits and risks, uncertainty, completeness, and usability, so higher scores suggest better informational support for patients seeking guidance on DKD. Reviews of LLM applications in patient education have similarly described their educational potential alongside concerns about reliability and bias (12, 33). The relatively low or variable JAMA scores also draw attention to source transparency. Originally developed to assess online medical information, the JAMA benchmarks address authorship, attribution, disclosure, and currency (27). Zhong et al. reported low JAMA scores for responses to patient-facing varicose vein questions despite generally acceptable information-quality ratings, reflecting limited attribution and verifiability cues in default outputs (29). For patients with DKD, these shortcomings may make it difficult to distinguish guideline-concordant recommendations from plausible but outdated or incomplete advice. Such assessments characterize the content and transparency of responses, but understanding their educational value also requires examining how patients perceive and use them. In a related kidney transplant study, Han and Lee surveyed 216 US adults with CKD who compared source-masked ChatGPT-4o and National Kidney Foundation responses to four transplant questions. Participants preferred ChatGPT in 81.3% of comparisons and rated its responses higher for perceived information quality, empathy, and learning (34). These findings illustrate patient-perceived value in transplant education, although perceived learning remains distinct from objectively measured comprehension or clinical benefit. Direct patient evaluation of DKD responses should therefore complement clinician assessment by examining perceived support, comprehension, and how patients interpret and apply safety-critical recommendations.

Although all readability indices differed significantly across models, the corresponding Kendall's *W* values indicated relatively small overall effects, and all models had high mean estimated reading-grade requirements. This pattern is consistent with a scoping review reporting variable readability in LLM-generated patient education materials, including instances in which simplification improved readability but left substantial reading demands (12). Mean grade-level estimates for all models remained well above the sixth-grade benchmark. Given the association between limited health literacy and adverse outcomes in CKD, this mismatch remains clinically relevant even when the medical content is otherwise rated favorably (6). Readability also appears to depend on the task. Shah et al. found that ChatGPT-5 generated easier-to-read responses than Claude 4.5 Sonnet when answering questions about intermittent fasting in CKD (19), whereas ChatGPT had the highest mean FKGL in our broader DKD question set. Differences in clinical topics, prompt sets, comparators, and model versions limit direct comparison and caution against treating a readability advantage as a stable property of a model. Will et al. improved the readability of existing patient-facing materials through explicit simplification prompts, although some rewritten content introduced inaccuracies (35). Our protocol did not request simplification; the findings therefore characterize readability under the specified query conditions rather than the limits of these systems' capacity for patient-oriented rewriting. Further evaluations should examine whether targeted prompts or system designs can produce concise, understandable DKD information while preserving essential medication precautions, safety information, and guidance on appropriate next steps.

These findings have several practical implications. Public-facing LLMs should be regarded as adjunctive communication tools rather than autonomous sources of DKD management advice. Safeguards should be risk-stratified: prompts involving medications, laboratory changes, fluid intake, diet, or referral decisions should trigger requests for relevant clinical context and default to conservative, uncertainty-aware language when that context is unavailable. Output rules should prohibit fixed patient-level prescriptions without supporting clinical information, require monitoring and sick-day advice when relevant, and provide clear symptoms or circumstances that warrant professional review. Generic disclaimers alone are unlikely to mitigate the omission-based risks observed here. A clinician-in-the-loop workflow could use LLMs to draft and simplify educational content while healthcare professionals verify guideline concordance, personalize recommendations, and ensure appropriate escalation. Future implementation studies should assess whether these safeguards reduce unsafe omissions without sacrificing empathy or readability, and should evaluate patient comprehension and decision-making rather than text quality alone. This approach is consistent with recent discussions emphasizing human oversight, safety standards, and risk-sensitive implementation of LLMs in healthcare (36, 37).

The study has several limitations. The findings reflect specific model versions and public web interfaces during the study period and may change with model updates or changes in information retrieval capabilities. Because only one response was generated per question–model combination, variability across repeated generations or query dates could not be assessed. The 42-question set did not cover the full range of patient scenarios, including rare complications, emergency symptoms, pregnancy, dialysis, and complex multimorbidity. Standardized single-turn prompts also did not capture conversations involving follow-up questions or clarification. The English-only evaluation limits generalizability to other languages, particularly Chinese-language use of Qwen, Doubao, and Kimi. All ratings were provided by clinicians, whose specialty backgrounds and experience in tertiary Grade A hospitals may have influenced judgments about omissions and safety advice despite source masking and calibration. Clinician-rated empathy captures observable supportive communication but may not reflect whether patients feel understood. Patients did not participate in scoring, and comprehension, perceived usefulness, emotional responses, adherence, and clinical outcomes were not assessed.

Future research should therefore incorporate repeated querying with documented model versions and query dates, alongside multilingual evaluation. Studies should compare single-turn and multi-turn interactions and assess guideline concordance, safety-critical omissions, source verifiability, patient comprehension, and patient-perceived support. Evaluating how patients understand and use model-generated responses, as well as the effects of these responses on self-management and clinical outcomes, would clarify their educational and clinical value.

## 5 Conclusions

This comparative evaluation indicates that publicly accessible LLMs can generate broadly plausible answers to patient-focused questions about DKD, although their performance was not consistent across all assessment domains. Safe-response rates did not differ statistically across models, which does not establish safety equivalence; qualitative examination identified clinically meaningful hazards, including omitted monitoring recommendations, inadequate individualized qualifications, and undue reassurance concerning medication-associated changes in laboratory parameters. ChatGPT demonstrated the strongest overall performance in accuracy and information quality. Gemini achieved the highest clinician-rated empathy score, while ChatGPT, Doubao, Kimi, and Qwen shared the next-highest median rating. DeepSeek had the lowest mean scores on all five grade-level indices and the highest mean Flesch Reading Ease Score. Nevertheless, mean estimated reading-grade requirements exceeded the sixth-grade reference level for every model, indicating a need for language simplification and direct evaluation of patient comprehension. Collectively, these findings support the use of LLMs as supplementary resources for DKD patient education, but not as substitutes for professional clinical guidance. Any clinical application should be grounded in current guidelines, supported by verifiable sources, optimized for readability, and implemented under clinician oversight. Future development should prioritize the inclusion of safety-critical qualifications, individualized communication of risk, and consistent use of plain language. Prospective studies with patients, repeated model queries, and multilingual evaluation are needed to determine whether these safeguards improve comprehension, perceived support, and safe decision-making.

## Data Availability

The original contributions presented in the study are included in the article/Supplementary Material, further inquiries can be directed to the corresponding author.
