# Supplementary material for: Safety, accuracy, empathy and information quality of six large language models answering patient-facing questions about diabetic kidney disease: a cross-sectional comparative study
Source: Front Med (Lausanne). 2026 Oct 30;13:2007153. doi: 10.3389/fmed.2026.2007153 (PMC13759355; doi:10.3389/fmed.2026.2007153)
Supplement: Supplementary file 1 [file Table1.docx]

**Supplementary material 1**

**CHART Checklist**

| Heading | No | Chart checklist item | **Manuscript section/page(s)** |
| --- | --- | --- | --- |
| Title and abstract |  |  | Title and Abstract, p. 1. |
| Title | 1a | State that the study is assessing one or more generative AI-driven chatbots for clinical evidence or health advice. | Title, p. 1: comparative evaluation of six LLMs for DKD patient education. |
| Abstract/summary | 1b | Apply a structured format, if applicable. | Abstract, p. 1: Background, Methods, Results, and Conclusions. |
| Introduction |  |  | Section 1 Introduction, pp. 2–3. |
| Background | 2a | State the scientific background, rationale, and healthcare context for evaluating the generative AI-driven chatbot(s), referencing relevant literature when applicable. | Section 1 Introduction, p. 2: DKD education needs, prior evidence, and rationale for multidimensional evaluation. |
|  | 2b | State the aims and research questions including the target audience, intervention, comparator(s), and outcome(s). | Section 1 Introduction, pp. 2–3; Section 2.1, p. 3: six chatbots; patients, caregivers, and professionals; primary safety and secondary outcomes. |
| Methods |  |  | Sections 2.1–2.9 Materials and Methods, pp. 3–8. |
| Model identifiers | 3a | State the name and version identifier(s) of the generative AI model(s) and chatbot(s) under evaluation, as well as their date of release or last update. | Section 2.3, p. 4; Table 1, p. 18. Interface names and available release information are reported; Doubao’s backend version and the exact DeepSeek-V4 variant are not disclosed. |
|  | 3b | State whether the generative AI model(s) and chatbot(s) are open-source or closed-source/proprietary. | Not reported. Section 2.3 (p. 4) and Table 1 (p. 18) do not explicitly identify open-source/open-weight versus closed-source/proprietary status for each system. |
| Model details | 4a | State whether the generative AI model was a base model or a novel base model, tuned model, or fine-tuned model. | Partially reported. Section 2.3, p. 4; Table 1, p. 18. Public end-user configurations and selected model details are described, but base/tuned/fine-tuned status is not consistently classified. |
|  | 4b | If a base model is used, cite its development in sufficient detail to identify the model. | Not explicitly addressed. Section 2.3, p. 4; Table 1, p. 18. End-user products are identified, but model-development citations sufficient to characterize any underlying base models are not provided. |
|  | 4c | If a novel base model, tuned model, or fine-tuned model is used, describe the pre- and/or post-implementation/deployment data and parameters. | Not applicable to study-specific model development: the study evaluates existing public chatbot products and describes no novel model training or tuning (Section 2.3, p. 4). |
| Prompt engineering | 5a | Describe the evolution of study prompt development. | Section 2.2, pp. 3–4: 86 candidate questions, translation/back-translation, expert review, and selection of 42 prompts across eight domains; SM2 cited. |
|  | 5ai | Describe the sources of prompts. | Section 2.2, p. 3: search trends, public forums, patient-education resources, guidelines/reviews, and specialist consultation; source audit cited as SM2. |
|  | 5aii | State the number and characteristics of the individual(s) involved in prompt engineering. | Section 2.2, p. 3: two bilingual translators, a third back-translation expert, and two nephrology reviewers. Overlap between roles and the total unique contributor count are not specified. |
|  | 5aiii | Provide details of any patient and public involvement during prompt engineering. | Section 2.2, p. 4: no direct patient/public involvement in prompt development; public patient-facing sources informed topics and wording indirectly. |
|  | 5b | Provide study prompts. | Section 2.2, pp. 3–4; SM3 is identified as the complete set of 42 standardized English DKD prompts. |
| Query strategy | 6a | State route of access to generative AI model. | Section 2.3, p. 4; Table 1, p. 18: official public web interfaces; no APIs. |
|  | 6b | State the date(s) and location(s) of queries for the generative AI-driven chatbot(s) including the day, month, and year as well as city and country. | Section 2.3, p. 4; Table 1, p. 18: June 24–July 12, 2026, in Huaihua, China. Individual query dates are not listed in the main manuscript. |
|  | 6c | Describe whether prompts were input into separate chat session(s). | Section 2.4, p. 4: each submission started a separate new conversation; only the first complete response was retained. |
|  | 6d | Provide all generative AI-driven chatbot output/responses | Section 2.4, p. 4; Data Availability Statement, p. 14. SM4 is identified as the complete question–response record for all six chatbots. |
| Performance evaluation | 7a | Define the ground truth or reference standard used to define successful generative AI-driven chatbot performance. | Section 2.5, p. 5; Section 2.7, pp. 5–7: question-specific guideline/evidence framework and outcome criteria; evidence mapping cited as SM5. |
|  | 7b | Describe the process undertaken for generative AI-driven chatbot performance evaluation. | Sections 2.4–2.5, pp. 4–5; Section 2.7, pp. 5–7: masking, training, separate calibration set, independent ratings, aggregation, qualitative review, and automated readability assessment. |
|  | 7bi | State the number and characteristics of team members involved in performance evaluation. | Section 2.5, p. 5: five clinicians with nephrology/endocrinology/diabetes expertise and >5 years of tertiary-hospital experience. Section 2.7, pp. 5–6: two nephrology clinicians performed qualitative review. |
|  | 7bii | Provide details of any patients and public involvement during the evaluation process. | Section 2.5, p. 5: patients and public representatives did not participate in performance evaluation. |
|  | 7biii | State whether evaluators were blinded to the identity of the generative AI-driven chatbot(s) under assessment. | Sections 2.4–2.5, pp. 4–5: model identifiers were replaced by neutral codes; evaluators were blinded during calibration and formal scoring. |
| Sample size | 8 | Report how the sample size was determined. | Section 2.6, p. 5: pragmatic domain coverage and feasibility determined 42 prompts × six models = 252 responses; no formal power calculation. |
| Data analysis | 9a | Describe statistical analysis methods, including any evaluation of reproducibility of generative AI-driven chatbot responses. | Section 2.8, pp. 7–8: matched-question analyses, agreement statistics, effect sizes, and multiplicity adjustment. Section 2.4, p. 4: temporal/stochastic output reproducibility was not evaluated; each combination was queried once. |
|  | 9ai | Report the measures used for performance evaluation. | Section 2.7, pp. 5–7: binary safety; 5-point accuracy/empathy; DISCERN; adapted EQIP; JAMA; GQS; and six automated readability indices. |
| Results |  |  | Sections 3.1–3.5 Results, pp. 8–10; Tables 2–5, pp. 19–21. |
|  | 10a | Report the performance evaluation undertaken including the alignment between generative AI-driven chatbot output and ground truth or reference standard using quantitative or mixed methods approaches as applicable. | Sections 3.1–3.5, pp. 8–10; Tables 2–5, pp. 19–21; Figures 2–4, pp. 23–24. Quantitative performance and qualitative safety findings are reported; SM6–SM7 cited. |
|  | 10b | For responses deviating from the ground truth or reference standard, state the nature of the difference(s). | Section 3.5, p. 10: six failure modes among 30 potentially unsafe responses, with examples involving fluid limits, albuminuria confirmation, and eGFR decline; SM7 cited. |
|  | 10c | Report the evaluation for potentially harmful, biased, or misleading responses. | Sections 3.2 and 3.5, pp. 8–10; Table 3, p. 20; SM7 cited. Potential harm and misleading advice are evaluated; a separate systematic bias/fairness assessment is not reported. |
| Discussion |  |  | Section 4 Discussion, pp. 10–13. |
|  | 11a | Interpret study findings in the context of relevant evidence. | Section 4 Discussion, pp. 10–12: findings are interpreted against DKD guidance and prior LLM research. |
|  | 11b | Describe the strengths and limitations of the study. | Section 4, pp. 10–13: comparative scope and limitations are discussed; limitations appear in the final paragraph (pp. 12–13). Design strengths are also described in Sections 2.1–2.5 (pp. 3–5). |
|  | 11c | Describe the potential implications for practice, education, policy, regulation, and research. | Section 4, pp. 12–13; Section 5 Conclusions, p. 13: clinician oversight, risk-sensitive safeguards, understandable education, safety standards, and future evaluation. |
| Open science |  |  | Conflict of Interest and Funding, p. 13; Ethics Statement, pp. 13–14; Data Availability Statement, p. 14. |
| Disclosures | 12a | Report any relevant conflicts of interest for all authors. | Conflict of Interest, p. 13: the authors declare no conflicts of interest. |
| Funding | 12b | Report sources of funding and their role in the conduct and reporting of the study. | Partially reported. Funding, p. 13: funding sources and grant identifiers are listed; the funders’ role in study conduct and reporting is not stated. |
| Ethics | 12c | Describe the process undertaken for ethical approval. | Section 2.9, p. 8; Ethics Statement, pp. 13–14. The manuscript states that formal ethics review was not required and consent was not applicable; no named committee determination or approval/waiver identifier is provided. |
|  | 12ci | Describe the measures taken to safeguard data privacy of patient health information, as applicable. | Section 2.2, p. 4; Section 2.4, p. 4; Ethics Statement and Data Availability Statement, p. 14: no identifiable patient information or private user content was entered, collected, or retained. |
|  | 12cii | State whether permission/licensing was obtained for the use of original, copyrighted data. | Not reported. Sections 2.2 (pp. 3–4) and the Ethics Statement (p. 14) describe use of public sources for broad themes, but do not state whether copyright permissions/licences were obtained or unnecessary. |
| Protocol | 12d | Provide a study protocol. | Not reported. No standalone study protocol, protocol access location, or registration statement is provided. References to study procedures in Section 2.4 (p. 4) do not provide an accessible protocol. |
| Data availability | 12e | State where study data, code repository, and model parameters can be accessed. | Partially reported. Data Availability Statement, p. 14, identifies article/SM2–SM7 materials. No analysis-code repository, full rater-level dataset location, or access statement for model parameters is specified. |

Footer Note: AI, artificial intelligence.
