# Supplementary material for: Safety, accuracy, empathy and information quality of six large language models answering patient-facing questions about diabetic kidney disease: a cross-sectional comparative study
Source: Front Med (Lausanne). 2026 Oct 30;13:2007153. doi: 10.3389/fmed.2026.2007153 (PMC13759355; doi:10.3389/fmed.2026.2007153)
Supplement: Supplementary file 4 [file Table4.docx]

**Supplementary material 4**

**Complete Question and Answer Records**

**Dimension 1-Disease understanding**

**Q1.What is diabetic kidney disease, and is it the same as ordinary chronic kidney disease?**

model: ChatGPT

**answer:**

Diabetic kidney disease (DKD) is kidney damage caused by diabetes. Over time, high blood sugar can injure the small blood vessels and filtering units in the kidneys; high blood pressure, which is common in diabetes, can make this worse. It usually develops slowly over years and often has no symptoms in the early stages.

It is not exactly the same as "ordinary" chronic kidney disease (CKD). CKD is the broader umbrella term: it means the kidneys have been damaged or have reduced function for at least 3 months, from any cause. Diabetes is one of the most common causes, but CKD can also be caused by high blood pressure, glomerulonephritis, IgA nephropathy, polycystic kidney disease, lupus, repeated infections, kidney stones, and other conditions.

So the relationship is:

DKD is one specific type/cause of CKD

CKD is the general category of long term kidney disease

A simple analogy: CKD is like "lung disease"; DKD is like "smoking related lung disease" one is the broad category, the other names a specific cause.

Clinically, doctors usually check for DKD/CKD using two key tests: a blood test called eGFR, which estimates kidney filtering function, and a urine test called uACR, which checks for albumin/protein leakage. Persistent eGFR below 60 and/or uACR above 30 for 3 months or more can indicate CKD.

In practice, a person with diabetes can have kidney disease from diabetes, from another cause, or from mixed causes so "has diabetes plus has CKD" does not automatically prove DKD. Doctors judge this using diabetes history, urine albumin pattern, eGFR trend, blood pressure, eye disease, urine findings, imaging, and sometimes kidney biopsy.

model: Gemini

**answer:**

Diabetic kidney disease (DKD), also known as diabetic nephropathy, is not exactly the same as "ordinary" chronic kidney disease (CKD) rather, DKD is a specific type of CKD.Think of Chronic Kidney Disease as a broad umbrella term for any condition that causes gradual, long term loss of kidney function. Diabetic Kidney Disease is the specific damage that happens under that umbrella when the root cause is diabetes. In fact, diabetes is the leading cause of CKD worldwide, accounting for roughly one third of all cases. What is Diabetic Kidney Disease?Your kidneys are filled with millions of microscopic filtering units called glomeruli. When you have diabetes, prolonged high blood sugar (hyperglycemia) changes how blood flows through your body and creates an inflammatory environment. Over time, this extra glucose forces the kidneys to filter too much blood (hyperfiltration). The strain damages the small blood vessels inside the glomeruli, causing their walls to thicken and leak.

Because the filters become leaky, the earliest sign of DKD is often the presence of a protein called albumin in the urine (albuminuria). If the blood vessels continue to take damage, the kidneys gradually lose their ability to filter waste and extra fluid from the blood.

How DKD Compares to General CKD

While the end result a gradual decline in kidney function looks very similar across the board, the starting point and specific structural damage differ.

Feature Chronic Kidney Disease (CKD) Diabetic Kidney Disease (DKD)

Definition An umbrella term for a long term decline in kidney function. A specific diagnosis of kidney damage under the CKD umbrella.

Primary Causes High blood pressure, diabetes, genetics, autoimmune issues, or recurrent infections. Exclusively caused by the vascular damage of Type 1 or Type 2 diabetes.

Early Indicators Often completely silent; specific signs vary by the root cause. Typically marked by high levels of albumin (protein) leaking into the urine.

Structural Changes Varies widely (e.g, cysts in polycystic kidney disease, scarring in hypertension). Characterized by thickened filter membranes and enlarged, strained blood vessels.

Ultimately, treating DKD requires managing the underlying diabetes specifically through tight blood sugar control and blood pressure management to protect the remaining healthy filters and prevent the condition from advancing to later stages of CKD.

model: DeepSeek

**answer:**

In short: No, diabetic kidney disease is not the same as "ordinary" chronic kidney disease (CKD), but it is the most common cause of it.

Think of it this way:

Chronic Kidney Disease (CKD) is the broad, "umbrella" term for any long term damage to your kidneys that affects their ability to filter blood.

Diabetic Kidney Disease (DKD) is a specific type of CKD caused by diabetes. It is also called diabetic nephropathy.

Here's a detailed breakdown of what each is and how they differ.

What is Diabetic Kidney Disease (DKD)?

DKD is kidney damage caused specifically by the stress of high blood sugar levels from diabetes (type 1 or type 2).

The Mechanism: Over time, high blood sugar damages the millions of tiny blood vessels (glomeruli) inside the kidneys that act as filters. This damage makes the filters "leaky" (letting protein like albumin spill into the urine) and then scarred (causing the filter to work poorly).

Key Feature: One of the earliest signs of DKD is albuminuria (protein, specifically albumin, in the urine). It often develops slowly over many years.

Typical Progression: DKD has a fairly predictable pattern, often starting with microalbuminuria (small amounts of protein), then macroalbuminuria (larger amounts), followed by a slow, steady decline in kidney function (measured by eGFR). It is a leading cause of end stage renal disease (ESRD) requiring dialysis.

What is "Ordinary" Chronic Kidney Disease (CKD)?

"Ordinary" CKD is the term often used for CKD that is not caused by diabetes. Common causes include:

Hypertension (High Blood Pressure): The second most common cause of CKD. High pressure damages the small arteries in the kidneys.

Glomerulonephritis: A group of diseases that cause inflammation of the kidney's filtering units (the glomeruli). This is an immune system issue, not a metabolic one.

Polycystic Kidney Disease (PKD): An inherited genetic disorder where fluid filled cysts grow in the kidneys.

Obstruction: From kidney stones, an enlarged prostate, or structural problems.

Autoimmune diseases: Like Lupus (lupus nephritis).

Key Differences at a Glance

Feature Diabetic Kidney Disease (DKD) "Ordinary" Chronic Kidney Disease (Non Diabetic)

Root Cause Chronic high blood sugar from diabetes Hypertension, glomerulonephritis, PKD, toxins, etc.

Early Sign Albuminuria (leaking protein) is a very early and prominent sign. May or may not have protein. Often shows as lower eGFR first, or blood in urine (hematuria).

Typical Findings Enlarged kidneys (in early stages), protein in urine, with or without diabetic eye disease (retinopathy). Normal or shrunken kidneys (in late stages), possible blood in urine, red blood cell casts.

Primary Treatment Strict blood glucose control plus blood pressure control (ACE/ARB drugs). Treat underlying cause (e.g, strict blood pressure control for hypertension, immunosuppression for glomerulonephritis).

Why Is the Distinction Crucial?

Because the treatment is different, especially in the early stages.

Blood Sugar Control is Paramount for DKD: For a patient with DKD, lowering blood sugar (HbA1c) is the single most important thing to slow progression. For someone with hypertensive CKD, tight glucose control makes no difference.

Specific Medications: Newer drug classes like SGLT2 inhibitors (e.g, empagliflozin, dapagliflozin) are powerfully protective for kidneys in people with DKD. They also benefit other CKD patients, but the evidence is strongest and they are standard of care first for DKD.

Blood Pressure Targets: While blood pressure control is vital for all CKD, the targets and preferred medications (e.g, ACE inhibitors or ARBs) are especially critical in DKD to reduce protein leakage.

Screening Recommendations: Someone with diabetes needs a urine test for albumin annually to screen for early DKD, even if their blood sugar seems well controlled. This specific screening is not routine for everyone without risk factors.

Can someone have both?

Absolutely. This is very common. A person with long standing diabetes can develop DKD. If they also have poorly controlled high blood pressure (which often coexists with diabetes), the combination can cause a "mixed picture" of both DKD and hypertensive CKD. In this case, the kidney damage is driven by two separate but overlapping mechanisms.

The Bottom Line

Diabetic Kidney Disease is CKD caused by diabetes. It has a distinct cause, progression, and treatment focus (glucose control).

"Ordinary" CKD is CKD from any other cause (like high blood pressure or genetics).

All DKD is CKD, but not all CKD is DKD. Knowing the specific cause is essential for choosing the right treatment to slow or stop the damage. If you or a loved one has CKD, determining whether diabetes is the underlying cause is the first critical step.

model: Doubao

**answer:**

Diabetic kidney disease (DKD) is a chronic kidney complication caused by long term poorly controlled diabetes (type 1 or type 2 diabetes).

High blood sugar damages the tiny blood vessels and filtering units (glomeruli) in the kidneys over years. Early signs include mild protein leakage in urine; as it worsens, patients develop swelling, high blood pressure, declining kidney function, and may eventually progress to kidney failure. It is the leading cause of end stage renal disease (ESRD) worldwide.

No, they are not the same. DKD is one specific type of chronic kidney disease (CKD). The relationship and key differences are as follows:

Core relationship

Chronic kidney disease (CKD) is a general umbrella term: It refers to all long term kidney damage lasting more than 3 months, with impaired filtration or structural abnormalities. It has many causes.

Diabetic kidney disease (DKD) is a secondary CKD, triggered exclusively by diabetes.

Main differences

Cause

DKD: Long term diabetes (high blood glucose, high blood pressure linked to diabetes).

Ordinary CKD: Multiple causes, e.g, chronic nephritis, hypertension alone, urinary tract diseases, drug toxicity, autoimmune kidney issues, genetic kidney diseases, etc.

Pathological features

DKD: Typical damage to renal glomeruli and blood vessels caused by hyperglycemia.

Other CKD: Lesions vary greatly based on different primary illnesses.

Population and prevalence

DKD: Mainly occurs in people with a long history of diabetes.

Ordinary CKD: Affects people of all ages, with diverse patient groups.

Treatment focus

DKD: Prioritize strict blood sugar and blood pressure control, plus kidney protection.

Other CKD: Treat the original disease first (e.g, anti inflammatory therapy for nephritis), combined with kidney supportive care.

In short: All DKD is CKD, but not all CKD is diabetic kidney disease.

model: Kimi

**answer:**

No, diabetic kidney disease (DKD) and ordinary chronic kidney disease (CKD) are not the same though they are closely related. In simple terms, DKD is a specific type of CKD, while CKD is a broad umbrella term that includes many different causes of long term kidney damage.

What is diabetic kidney disease (DKD)?

DKD also called diabetic nephropathy is kidney disease that develops as a direct complication of diabetes. It is defined as CKD in a person with diabetes where the kidney damage is attributable to diabetes related pathology. It is typically characterized by:

A gradually falling glomerular filtration rate (eGFR), and/or

A steadily rising level of albumin in the urine (albuminuria), usually measured by the urinary albumin to creatinine ratio (uACR)

Clinically, DKD is diagnosed when a patient with diabetes has an eGFR less than or equal to 60 mL/min/1.73 m2 or a uACR greater than or equal to 3 mg/mmol (or greater than or equal to 30 mg/g), and other primary kidney diseases have been reasonably excluded. It is considered a microvascular complication of diabetes, alongside diabetic retinopathy and neuropathy.

What is chronic kidney disease (CKD)?

CKD is a general clinical syndrome defined as abnormal kidney structure or function that persists for more than three months, with implications for health. It can be caused by a wide variety of conditions, including:

Diabetes (the most common cause globally)

High blood pressure (hypertensive nephrosclerosis)

Glomerulonephritis and other autoimmune diseases

Polycystic kidney disease and other genetic disorders

Recurrent infections, obstructive uropathy, and certain medications

How are they different?

Feature DKD Ordinary CKD

Scope A specific cause of CKD A broad category of kidney disease

Cause Diabetes related metabolic and hemodynamic damage Multiple possible causes (diabetes, hypertension, genetics, etc.)

Diagnosis Requires diabetes plus CKD criteria plus exclusion of other causes Requires only greater than 3 months of abnormal kidney function/structure

Pathology Specific glomerular changes from chronic hyperglycemia (e.g, mesangial expansion, glomerulosclerosis, podocyte damage) Varies by underlying cause

Importantly, not all CKD in people with diabetes is DKD. Diabetic patients can also develop kidney disease from high blood pressure, ischemic injury, glomerulonephritis, or other unrelated conditions. In such cases, it is classified as CKD from another cause, not DKD.

Why does the distinction matter?

While both conditions share common features like proteinuria and declining kidney function, identifying DKD specifically is important because:

Treatment emphasis: DKD management prioritizes strict glycemic control and kidney protective medications such as ACE inhibitors/ARBs, SGLT2 inhibitors, and GLP 1 receptor agonists that have proven benefit in diabetic kidney disease.

Prognosis: DKD is currently the single most common cause of end stage kidney disease (ESKD) requiring dialysis or transplantation worldwide.

Screening: All patients with diabetes should be screened annually for DKD with uACR and eGFR testing, whereas CKD screening in non diabetic populations targets different risk groups like those with hypertension or cardiovascular disease.

In summary: All DKD is CKD, but not all CKD is DKD. DKD is the specific form of chronic kidney disease caused by diabetes, while CKD encompasses any long term kidney damage regardless of origin.

model: Qwen

**answer:**

Diabetic kidney disease is a specific type of chronic kidney disease, and the two are not the same. Chronic kidney disease is an umbrella term, while diabetic kidney disease is a specific cause within that category.

The Relationship Between the Two

Think of it like the relationship between "fruit" and "apple." Chronic kidney disease (CKD) is the broad category for any long term kidney damage, whereas diabetic kidney disease (DKD) is specifically caused by high blood sugar.

How Diabetic Kidney Disease Develops

Long term high blood sugar damages the tiny filtering units in the kidneys. This process usually follows a predictable pattern:

Early Stage: The first sign is small amounts of protein leaking into the urine (microalbuminuria). At this point, kidney function tests are often normal, and there are no symptoms.

Progression: As damage worsens, protein loss increases, often accompanied by swelling and high blood pressure. Kidney function begins to decline significantly.

End Stage: Without intervention, it can lead to irreversible kidney failure.

Key Differences in Management

Causes: CKD has many causes, including high blood pressure or autoimmune diseases. DKD has only one cause: diabetes.

Treatment Focus: Managing CKD depends on the specific cause. For DKD, the priority is strict control of blood sugar and blood pressure. Some modern medicines lower blood sugar and directly protect the kidneys at the same time.

Summary

While all diabetic kidney disease is a form of chronic kidney disease, not all chronic kidney disease is diabetic. For people with diabetes, regular screening of urine and blood is essential to catch kidney problems early.

**Q2. How does high blood sugar damage the kidneys over time?**

model: ChatGPT

**answer:**

High blood sugar damages the kidneys mainly by injuring their tiny filtering blood vessels.

Your kidneys contain millions of microscopic filters called nephrons. Inside them are tiny blood vessels called glomeruli, which clean waste and extra fluid from the blood while keeping useful proteins in the bloodstream. Over time, high blood sugar can damage these vessels and filters, so they stop working normally.

The damage usually develops in stages:

Overwork of the filters

High glucose makes the kidneys filter more blood than usual. This "extra workload" stresses the delicate filters. The American Diabetes Association describes this as the kidneys "filtering too much blood," which is hard on the filters over many years.

Thickening and scarring

Persistent high blood sugar causes structural changes, including thickening of the glomerular basement membrane, expansion of supporting kidney tissue, and damage to podocytes, which are cells that help keep protein in the blood. These changes make the filters stiff and leaky.

Protein leaks into urine

As the filters become damaged, proteins such as albumin can pass into the urine. Early leakage is often called microalbuminuria. This can be one of the first signs of diabetic kidney disease.

Filtering ability declines

With continued damage and scarring, the kidneys gradually lose their ability to remove waste and extra fluid from the blood. This can progress to chronic kidney disease and, in severe cases, kidney failure.

High blood pressure often develops alongside diabetes and speeds up kidney damage by putting even more pressure on the tiny kidney blood vessels.

In simple terms: high blood sugar makes the kidney filters work too hard, injures their small blood vessels, makes them leaky, and eventually causes scarring that reduces kidney function.

model: Gemini

**answer:**

High blood sugar damages the kidneys through a slow, progressive process that essentially overworks and physically alters the kidneys' microscopic filtering units. This condition is known as diabetic nephropathy.

To understand how this happens, it helps to look closely at the glomerulus the tiny ball of blood vessels inside the kidney that acts as the actual filter.

Here is the step by step breakdown of how chronic high blood sugar breaks down this filtration system:

1. The Pressure Cooker Effect (Hyperfiltration)

When blood sugar levels are chronically high, the body tries to clear the excess glucose by pushing more blood through the kidneys. This increases the blood pressure inside the fragile capillaries of the glomerulus. Over time, this constant high pressure state (hyperfiltration) stretches and physically stresses the filtering vessels.

2. "Sugar Coating" the Filters (Glycation)

Excess glucose in the blood doesn't just float harmlessly; it sticks to proteins in the blood vessels, forming molecules called Advanced Glycation End products (AGEs).

This "sugar coating" triggers inflammation and causes the glomerular basement membrane (the main physical barrier that separates waste from blood) to become abnormally thick and stiff, as seen in the green outline in the diagram above.

At the same time, the supporting cells inside the filter (mesangial cells) multiply and produce excess tissue, clogging up the filter's working space.

3. The Leaky Filter (Podocyte Damage)

The outside of the glomerular filter is lined with specialized, octopus like cells called podocytes. Their "tentacles" interlock to create tiny slits that prevent essential proteins from escaping into the urine.

The combination of high physical pressure and toxic sugar byproducts damages these podocytes, causing them to detach and die off. Once these cells are gone, the filter loses its structural integrity and begins to leak. This is why the presence of albumin (a blood protein) in the urine is one of the earliest medical signs of kidney damage.

4. Scarring and Failure (Glomerulosclerosis)

As the damage continues, the constant inflammation and cellular stress lead to the formation of scar tissue a process called glomerulosclerosis. As more and more glomeruli turn into useless scar tissue, the kidneys lose their overall capacity to filter toxins and waste out of the blood, eventually leading to chronic kidney disease or kidney failure.

model: DeepSeek

**answer:**

Persistently high blood sugar can gradually damage the kidneys through excessive pressure inside the kidney’s filtering units and direct injury to kidney cells.

At first, high blood sugar makes the kidneys filter blood too forcefully. Over time, this extra pressure damages the delicate filtering membranes. Excess glucose can also attach to proteins in kidney tissue, forming harmful substances called advanced glycation end products. These substances promote inflammation, oxidative stress, and stiffening of the kidney’s filtration barrier.

High blood sugar may also overactivate hormonal systems such as the renin–angiotensin–aldosterone system, further increasing pressure inside the kidneys and promoting scarring. Kidney cells may enlarge and produce excess scar tissue, while inflammation and oxidative stress damage blood vessel cells and podocytes, which normally help prevent protein from leaking into the urine.

Over the years, these changes may cause small amounts of protein to appear in the urine, followed by heavier proteinuria, declining kidney filtration, and eventually end-stage kidney disease. Good blood sugar and blood pressure control, together with kidney-protective medicines such as ACE inhibitors, ARBs, and SGLT2 inhibitors, can help slow this progression.

model: Doubao

**answer:**

Long term high blood sugar harms the kidneys through several progressive mechanisms:

First, excess glucose raises pressure inside the glomeruli, the tiny filtering units of the kidneys. This constant high pressure thickens and stiffens the delicate blood vessel walls over time, damaging their filtering function.

As the glomeruli become impaired, they can no longer keep protein in the bloodstream. Protein leaks into the urine, a key early sign of diabetic kidney disease.

With more filtering units destroyed gradually, the kidneys lose their ability to remove waste products and extra fluid from the body effectively.

In addition, kidney damage often leads to high blood pressure. Elevated blood pressure in turn puts more strain on the kidneys, creating a vicious cycle. If left uncontrolled, this ongoing damage will eventually result in chronic kidney disease or even complete kidney failure.

model: Kimi

**answer:**

High blood sugar damages the kidneys through a gradual, multi step process called diabetic nephropathy. Here is how it unfolds over time:

1. Excess Filtration (Early Stage)

When blood sugar is chronically high, the kidneys filter more blood than usual to clear the extra glucose. This hyperfiltration puts excessive strain on the tiny filtering units (glomeruli), stretching and stressing their delicate blood vessels.

2. Structural Damage to the Filters

Over years, several harmful processes occur simultaneously:

Advanced Glycation End products (AGEs): Excess glucose sticks to proteins in the kidney tissue, forming AGEs. These cross link and stiffen the filtering membranes, making them leaky and less selective.

Oxidative Stress: High glucose increases production of reactive oxygen species, which damage kidney cells and their DNA.

Inflammation: Damaged cells release inflammatory signals that recruit immune cells, causing chronic low grade inflammation that scars kidney tissue.

3. Hemodynamic Changes

High blood sugar activates the renin angiotensin system, causing constriction of the blood vessels leading out of the glomerulus. This raises pressure inside the filter, much like stepping on a garden hose increases pressure upstream. The elevated glomerular pressure progressively injures the filtration barrier.

4. Protein Leakage (Albuminuria)

As the glomerular membrane becomes damaged, it starts leaking protein (albumin) into the urine. This is often the first clinically detectable sign of kidney damage. The leaked protein is also toxic to the kidney tubules, accelerating injury.

5. Scarring and Decline (Progressive Stage)

With ongoing damage:

Glomerulosclerosis: The filtering units scar and become obsolete.

Tubulointerstitial fibrosis: The supporting tissue between filters turns fibrotic.

Blood vessel narrowing: The small arteries supplying the kidneys harden and narrow.

The kidneys slowly lose functional nephrons. The remaining ones work even harder, which paradoxically speeds up damage to them as well a vicious cycle.

6. End Stage Kidney DiseaseEventually, so much kidney tissue is scarred that the organs can no longer maintain adequate blood filtration, electrolyte balance, or blood pressure control. At this stage, dialysis or transplantation becomes necessary.

Key Timeline

Years 1 to 5: Hyperfiltration and subtle changes; often no symptoms.

Years 5 to 15: Microalbuminuria appears; blood pressure may rise.

Years 15 to 25: Declining glomerular filtration rate (GFR); rising creatinine.

Years 20 plus: Progressive loss of function; risk of kidney failure increases substantially.

What Slows It DownTight blood sugar control, blood pressure management (especially ACE inhibitors or ARBs), and reducing protein in the diet can significantly slow or halt this progression.

If you or someone you know is dealing with this, a nephrologist can monitor urine albumin to creatinine ratio and eGFR to catch changes early.

model: Qwen

**answer:**

Long term high blood sugar damages kidneys primarily by destroying the filtration membrane and causing vascular hardening.

This process often takes years and can eventually lead to kidney failure if left unmanaged.

Three Core Mechanisms of Damage

Leaking Filter Membranes: The kidneys act as a precise sieve to filter waste. Chronic hyperglycemia compromises the structural integrity of this membrane, causing it to become porous. This allows essential proteins, such as albumin, to leak into the urine, which is the earliest warning sign of kidney injury.

Overloading the Filtering System: To process excess sugar, the kidneys must work overtime. This increased workload raises the internal pressure within the glomeruli (the kidney's tiny filtering units). Similar to a water pipe under sustained high pressure, the glomeruli can eventually become damaged and harden.

Vascular Stiffening and Blockage: Excess glucose triggers oxidative stress, which accelerates the hardening of small arteries within the kidneys. This narrowing reduces blood flow, leaving the kidneys in a state of chronic ischemia and hypoxia, which progressively degrades their filtering capacity.

Hidden Progression and Consequences

This damage occurs very slowly and stealthily, often taking a decade or more to become clinically significant. The kidneys are remarkably resilient; even when function is lost by more than half, the body usually shows no symptoms. Many people do not realize the severity of the damage until kidney function drops precipitously, potentially leading to end stage renal disease (kidney failure).

Key Protective Actions

Strict Blood Sugar Control: Regular monitoring of blood glucose levels and glycated hemoglobin (HbA1c) every three months is the most critical investment for kidney preservation.

Blood Pressure Management: Hypertension works synergistically with hyperglycemia to accelerate kidney damage. Keeping blood pressure within an ideal range is essential.

Regular Urine Testing: Conducting an annual Urine Albumin to Creatinine Ratio (UACR) test is the most sensitive method for early detection of kidney injury.

In summary, high blood sugar initiates a slow progression from microscopic protein leakage to vascular hardening and eventual organ failure. However, by rigorously controlling glucose and blood pressure levels and regularly screening for urinary proteins, most severe kidney damage can be effectively prevented or delayed.

**Q3. Can I have diabetic kidney disease even if I feel completely well?**

model: ChatGPT

**answer:**

Yes. You can have diabetic kidney disease even if you feel completely well. In the early stages, it often causes no symptoms, so many people do not know they have it unless they are tested. The NIDDK states that "most people with diabetic kidney disease do not have symptoms," and CDC similarly notes that chronic kidney disease often has no early signs.

The usual tests are simple:

Urine albumin to creatinine ratio, or uACR checks whether protein/albumin is leaking into the urine, which can be an early sign of kidney damage.

Blood creatinine with eGFR estimates how well the kidneys are filtering blood.

Current ADA Standards of Care recommend checking both urine albumin and eGFR at least annually in people with diabetes, because these tests can detect diabetic kidney disease before symptoms appear.

Symptoms, when they do occur, usually appear later and may include swelling in the feet or ankles, foamy urine, fatigue, poor appetite, nausea, or worsening blood pressure. But feeling normal does not rule it out.

So the practical answer is: yes, it is possible ask your doctor whether you have had a recent uACR urine test and eGFR blood test.

model: Gemini

**answer:**

Yes, absolutely. It is very common to have diabetic kidney disease (also known as diabetic nephropathy) and feel completely fine.

In fact, early stage kidney disease is almost always "silent" and produces zero noticeable symptoms.

Why Early Kidney Disease is Silent

Your kidneys are highly adaptable and have a massive reserve capacity. Even if a significant portion of the kidney's filtering units (nephrons) are damaged by high blood sugar, the remaining healthy units will simply work harder to compensate. Because of this, your body continues to filter waste and balance fluids effectively enough that you won't feel sick, fatigued, or notice any physical changes.

People often do not experience symptoms such as swelling in the ankles, changes in urination, fatigue, or nausea until the kidney disease has progressed to a very advanced stage.

How It Is Caught Without Symptoms

Because you cannot rely on how you feel to tell you if your kidneys are healthy, routine medical screening is critical for anyone with diabetes. Doctors monitor for diabetic kidney disease using two simple tests:

Urine Test (UACR): This checks for a protein called albumin in your urine. Healthy kidneys keep protein in the blood, so even a tiny amount of albumin in the urine (microalbuminuria) is one of the earliest signs of kidney stress.

Blood Test (eGFR): This measures creatinine (a waste product from muscles) in your blood to calculate your estimated Glomerular Filtration Rate (eGFR), which tells your doctor exactly how well your kidneys are filtering waste.

The Importance of Screening

Finding diabetic kidney disease early is crucial because that is when treatments are most effective. If it is caught before you have symptoms, your doctor can prescribe medications and help you make lifestyle or diet adjustments that can protect your kidneys and significantly slow or even stop the disease from progressing.

If you have diabetes and haven't had your kidney function checked recently, it is highly recommended to schedule a routine screening with your doctor, regardless of how healthy you feel.

model: DeepSeek

**answer:**

Yes, it is absolutely possible to have diabetic kidney disease (diabetic nephropathy) even if you feel completely well. In fact, early stage kidney damage from diabetes typically causes no noticeable symptoms. The condition often develops gradually over years, and by the time symptoms like swelling (edema), fatigue, or changes in urination appear, the disease may already be advanced.

Regular screening is essential for people with diabetes. Your doctor should check your urine for albumin (a protein that leaks into urine when the kidneys are damaged) and measure your blood creatinine levels to calculate your eGFR (estimated glomerular filtration rate). These tests can detect kidney disease long before you feel unwell.

If you have diabetes especially if it has been present for several years or if your blood glucose or blood pressure has not been well controlled do not rely on how you feel. Stick to recommended annual kidney function tests, as early detection allows for treatments (like ACE inhibitors, ARBs, or SGLT2 inhibitors) that can slow or even halt progression.

model: Doubao

**answer:**

Yes, you can absolutely have diabetic kidney disease (DKD) while feeling perfectly healthy.

In the early stages, DKD usually causes no obvious symptoms. High blood sugar gradually damages the kidneys slowly over years, and you will not feel pain, fatigue or discomfort at first. Many people only detect kidney problems through routine urine and blood tests.

As the condition worsens, symptoms like swelling, tiredness or frequent urination may appear. That is why regular medical check ups are essential for people with diabetes.

model: Kimi

**answer:**

Yes absolutely. Diabetic kidney disease (DKD) often develops silently, and you can have it for years while feeling completely fine.

Here's why:

Early stage DKD usually has no symptoms. Your kidneys are remarkably resilient and can lose a significant amount of function before you notice anything. By the time symptoms like swelling, fatigue, or changes in urination appear, the disease may already be moderately advanced.

This is exactly why screening matters. If you have diabetes, routine kidney checks are essential even when you feel well. The standard tests are simple: Urine albumin to creatinine ratio (uACR) detects small amounts of protein leaking into urine, often the first sign of DKD eGFR blood test estimates how well your kidneys are filtering waste

How often to check: Most guidelines recommend at least annual screening for people with type 1 diabetes (after 5 years of diagnosis) and for everyone with type 2 diabetes from diagnosis onward. Your doctor may suggest more frequent testing if any results are abnormal.

Risk factors that make screening especially important: Longer duration of diabetes Poor blood sugar control High blood pressure Family history of kidney disease Smoking

The good news: When caught early, DKD progression can often be slowed or even prevented with tight blood sugar and blood pressure control, and medications like SGLT2 inhibitors or ACE inhibitors/ARBs.

If you have diabetes and haven't had your kidney function checked recently, it's worth bringing this up with your healthcare provider even if you feel perfectly healthy.

I'm an AI, not a doctor. This information is for educational purposes and doesn't replace personalized medical advice.

model: Qwen

**answer:**

Yes, you can absolutely have diabetic kidney disease even if you feel completely well. In fact, early stage kidney damage is usually silent.

Why You Feel Nothing

Diabetic kidney disease (DKD) is a chronic injury caused by long term high blood sugar damaging the tiny filtering vessels in the kidneys. A key feature of this condition is that the kidneys have a massive ability to compensate. Even if up to 50% of your kidney function is already damaged, the remaining healthy tissue works harder to make up for it, and you will likely feel perfectly normal. Symptoms like foamy urine, swelling in the legs or face, or fatigue typically only appear once the disease has progressed significantly.

The Silent Progression

The damage happens slowly over years. In the very early stages, the kidney filters develop tiny leaks that allow small amounts of protein to escape into the urine. This stage is called microalbuminuria, and while you feel no different, it is the most critical time to catch and reverse the condition.

Essential Screening Tests

Because you cannot rely on how you feel, regular blood and urine tests are the only way to detect this disease early.

Urine Albumin to Creatinine Ratio (UACR): This simple urine test checks for the presence of albumin (a type of protein). It is the most convenient and effective way to screen for early kidney damage.

Blood Creatinine Test: This is used to calculate your estimated Glomerular Filtration Rate (eGFR), which measures how well your kidneys are filtering waste from your blood.

Action Plan

If you have diabetes, you should not wait until you feel sick to check your kidneys.

Type 1 diabetes mellitus:Screen for kidney disease 5 years after diagnosis.

Type 2 diabetes mellitus:Screen immediately upon diagnosis, as kidney damage may have existed before the diabetes was even detected.

Frequency:Perform these tests at least once a year. If abnormalities are found, your doctor may recommend more frequent monitoring.

Feeling well is excellent, but it is not a guarantee of kidney health. The most effective way to protect your kidneys is to maintain good blood sugar and blood pressure control and to adhere to a strict schedule of annual urine and blood testing.

**Q4. Does foamy urine always mean I have diabetic kidney disease?**

model: ChatGPT

**answer:**

No. Foamy urine does not always mean diabetic kidney disease (DKD). Occasional bubbles can happen from a fast urine stream, dehydration, or even toilet cleaning chemicals. But persistent, thick, recurrent foam can be a sign of protein in the urine especially albumin which can occur with DKD or other kidney conditions.

For people with diabetes, the key point is that DKD often has no symptoms early on. NIDDK notes that the only way to know is through kidney checks: a urine albumin test and a blood test for kidney filtration/eGFR. The ADA 2026 Standards also recommend monitoring both albuminuria and eGFR at least annually in people with diabetes.

You should arrange testing sooner if the foamy urine is frequent or worsening, or if it comes with leg/ankle/face swelling, high blood pressure, fatigue, reduced urine, blood in urine, or shortness of breath. The most useful tests to ask about are:

Urine albumin to creatinine ratio, or UACR

Serum creatinine with eGFR

Urinalysis, to check protein, blood, infection, and other abnormalities

So the safest interpretation is: foamy urine is not a diagnosis, but in someone with diabetes it is worth checking rather than ignoring.

model: Gemini

**answer:**

No. While foamy urine can be a sign of diabetic kidney disease, it is very often caused by completely harmless, everyday factors or other unrelated medical conditions.

Here is a breakdown of why it happens and what else could be causing it.

The Connection to Kidney Disease

When kidneys are damaged whether by diabetes (a condition called diabetic nephropathy) or something else their filtering system can leak protein into the urine (a condition called proteinuria). Because protein acts as a surfactant, it interacts with air and water to create a stable, bubbly foam, very similar to whipping egg whites.

However, diabetes is far from the only reason this happens.

Common Harmless Causes

If you occasionally notice foam, it is often due to something benign:

High velocity: A rapid, forceful stream hitting the toilet water can whip up bubbles simply from the physical impact. This type of foam usually dissipates quickly, unlike protein foam which tends to stick around.

Dehydration: When you don't drink enough water, your urine becomes highly concentrated. The higher concentration of normal waste products can cause foaming.

Toilet chemicals: Sometimes the foam isn't from the urine itself, but from its reaction with residual cleaning chemicals or deodorizers in the toilet bowl.

Strenuous exercise: Intense, prolonged physical activity can cause a temporary, harmless spike of protein in the urine.

Other Medical Causes

If the foam is consistently present and actually caused by protein, diabetic kidney disease is just one possibility. Other medical causes include:

High blood pressure: The second leading cause of kidney damage after diabetes.

Urinary tract infections (UTIs): Infections can cause protein and white blood cells to enter the urine.

Other kidney conditions: Diseases like glomerulonephritis or nephrotic syndrome.

Certain medications: Some drugs, particularly NSAIDs (like ibuprofen), can affect kidney function and cause temporary proteinuria if taken in high doses.

What to Do Next

You cannot diagnose the cause of foamy urine just by looking at it. If you notice the foam happening consistently over several days especially if it is accompanied by other symptoms like swelling in your hands or feet, fatigue, or changes in urine color you should see a doctor.

A doctor can perform a simple, inexpensive urinalysis to quickly check for the presence of protein and determine if your kidneys need further evaluation.

model: DeepSeek

**answer:**

That's an excellent question, and it gets at a very common concern. Here's the short answer: No, foamy urine does not always mean you have diabetic kidney disease (DKD).In fact, it often means something else entirely.

However, because it can be a sign of DKD, it's something you should understand and discuss with your doctor.

Let's break down what foamy urine means, what causes it, and when it's linked to diabetic kidney disease.

What Causes Foamy Urine?

The most common reason for foamy urine is simply thespeed and forceof urination. When you hit the toilet water with enough force, it creates bubbles, just like when you run a faucet quickly into a sink. These bubbles are usually large, clear, and disappear quickly.

The concerning type of foam is different:It looks more like the froth or foam you'd see on a beer or on top of egg whites. It's made ofmany small, tight bubblesthat don't disappear quickly, even after you flush.

This type of persistent foam is often caused byprotein in the urine (proteinuria).

Think of it like this:Protein acts like a detergent. It lowers the surface tension of the liquid, which allows bubbles to form easily and stay stable for much longer.

Foamy Urine and Diabetic Kidney Disease (DKD)

Diabetic kidney disease damages the tiny filtering units in your kidneys (glomeruli). When these filters are damaged, they can leak a protein calledalbuminfrom your blood into your urine.

Yes, persistent foamy urine can be an early sign of DKD.If you have diabetes and your kidneys begin to leak protein, it's a key indicator of diabetic kidney disease.

However,the amount of protein needed to cause visible foam is often fairly significant. Many people in the early stages of DKD have "microalbuminuria" (small amounts of protein) that willnotcause foamy urine. The foam typically appears when protein leakage is more advanced.

Other Important Causes of Foamy Urine

This is critical to remember. Many other, often less serious, things can cause protein in the urine or directly cause foamy urine:

Dehydration:Highly concentrated urine is more likely to be bubbly.

Rapid Urination (Force):As mentioned, this is the most common cause.

Toilet Cleaners/Chemicals:Residual cleaning products in the toilet bowl can react with urine to create foam.

Other Kidney Diseases:Many non diabetic kidney diseases (like glomerulonephritis, IgA nephropathy, or hypertensive nephrosclerosis) cause protein leakage.

Urinary Tract Infection (UTI):Bacteria and white blood cells can change the surface tension of urine.

Retrograde Ejaculation (in men):This is a surprisingly common cause. A small amount of semen enters the bladder and is released with urine. Semen has a very high protein content, creating a lot of foam. This is often harmless, especially in younger men.

Preeclampsia:This serious pregnancy complication is marked by high blood pressure and protein in the urine.

So, What Should You Do?

Don't panic, but don't ignore it either. Follow these steps:

Examine the Foam:Next time you urinate, notice the bubbles. Are they large and clear and disappear in a few seconds? Or are they small, tight, white, and linger for minutes? The latter is more concerning.

Hydrate:Drink a glass of water and see if your next urine is less foamy.

See Your Doctor This is the most important step.The only way to know for sure is with simple, non invasive tests.

Urinalysis:A dipstick test can instantly detect if there is protein in your urine.

ACR (Albumin to Creatinine Ratio):This is the gold standard test, especially for people with diabetes. It measures exactly how much albumin is leaking.

Blood Test:To check your kidney function (Creatinine and eGFR).

The Bottom Line

Feature Normal Bubbles (Not Worrisome) Protein Foam (See a Doctor)

Appearance Large, clear, uneven bubbles Dense, small, white, frothy like beer/egg whites

How fast it disappears Within a few seconds Persists for minutes, or even after flushing

Common causes Forceful stream, dehydration Kidney disease, retrograde ejaculation, UTI

If you have diabetesand you're seeing persistent, small bubble foam,contact your doctor for a kidney function check.It's a crucial early warning sign.

If you do NOT have diabetes,foam is still worth mentioning to your doctor, but it is far more likely to be due to a benign cause like force, dehydration, or retrograde ejaculation.

Don't diagnose yourself.See your doctor for a simple urine test. It's the only way to get a clear answer and peace of mind.

model: Doubao

**answer:**

No, foamy urine does not always indicate diabetic kidney disease (DKD).

Harmless causes (most common)

Rapid urination, concentrated urine from dehydration

Diet high in protein

Temporary stress or fatigue

These foams usually disappear quickly.

Pathological causes

Diabetic kidney disease: Persistent, fine foam that lingers for a long time, caused by protein leakage in urine.

Other issues: Urinary tract infection, hypertension related kidney damage, liver disease, etc.

Advice

If you have long lasting foamy urine, especially with diabetes, get a urine albumin test and kidney function check for a clear diagnosis.

model: Kimi

**answer:**

No. Foamy urine does not always mean you have diabetic kidney disease, or any kidney disease at all. While it can be a sign of protein in the urine (proteinuria), which is associated with kidney damage, there are many other common and harmless causes.

Here are the main possibilities:

Benign causes of foamy urine:

Dehydration or concentrated urine

Fast/forceful urination hitting the toilet water

Residual semen in the urethra (after ejaculation)

Certain foods or medications

Toilet cleaning chemicals in the bowl

Medical causes to consider:

Proteinuria (protein leaking into urine) this is the link to diabetic kidney disease, but also occurs with urinary tract infections, high blood pressure, fever, or other kidney conditions

Diabetes if blood sugar is poorly controlled, it can cause temporary foamy urine even without permanent kidney damage

What you should do:

If the foaminess is persistent (lasts more than a few days), occurs every time you urinate, or is accompanied by swelling in legs/face, fatigue, or decreased appetite, you should see a doctor.

A simple urinalysis (dipstick test) can quickly check for protein in your urine.

If you have diabetes, regular screening for microalbuminuria is standard care.

Bottom line: Occasional foamy urine is usually nothing to worry about. Persistent foamy urine warrants a check up, but only a medical test can determine if it's related to diabetic kidney disease or something else entirely.

This information is for educational purposes and does not replace professional medical advice. If you're concerned, please consult your healthcare provider.

model: Qwen

**answer:**

No, foamy urine does not always indicate diabetic kidney disease. It is often harmless and can simply be a normal physiological phenomenon.

Common Physiological Causes

Often, foamy urine is just a normal occurrence. For instance, urinating too quickly, drinking too little water which causes urine concentration, or eating too much high protein food in a short period can all increase the surface tension of urine, creating foam.

These types of foam usually have large bubbles and dissipate within 1 to 2 minutes, having no impact on health.

Warning Signs to Watch For

The kind of foamy urine that truly needs attention is pathological, most commonly caused by proteinuria.

If urine mixes with protein, it significantly increases surface tension, forming small, dense foam that looks like beer foam.

This type of foam can remain for 5 minutes or longer even after standing still. Besides diabetic kidney disease, this could also be caused by glomerulonephritis or urinary tract infections.

Correct Action Steps

If you notice persistent foam, observe it for a few days first.

If the foam persists or you also experience puffiness around the eyelids or ankles, along with symptoms like frequent urination or pain during urination, go to the hospital for a urinalysis and urine microalbumin test.

These simple and quick tests can directly determine if there are abnormal substances in the urine.

In conclusion, foamy urine does not automatically mean diabetic kidney disease. Observe how quickly the foam dissipates, and if small bubbles persist, a simple urinalysis can clarify the cause, so there is no need for excessive anxiety.

**Q5. If my urine protein is high, does that mean my kidneys are already failing?**

model: ChatGPT

**answer:**

Not necessarily. High urine protein does not automatically mean your kidneys are failing. It means protein is leaking into the urine, which can be a warning sign of kidney stress or kidney damage, but kidney failure is usually judged by kidney filtering function, especially blood creatinine/eGFR, not urine protein alone.

Protein in urine can happen with kidney disease, especially from diabetes, high blood pressure, glomerulonephritis, or diabetic kidney disease. It can also rise temporarily from dehydration, fever, strenuous exercise, or acute illness, and may need repeat testing.

The key tests to ask your doctor about are:

Test What it tells you

Urine ACR/uACR How much albumin/protein is leaking into urine

Blood creatinine plus eGFR How well the kidneys are filtering blood

Blood pressure, blood sugar/HbA1c Common causes of proteinuria

Repeat urine test Confirms whether it is persistent or temporary

A urine albumin to creatinine ratio is the recommended test to assess and monitor urine albumin, and eGFR plus urine albumin are the two key markers used to evaluate kidney disease.

A simple way to think about it: protein in urine can be an early warning light; eGFR tells you more about how much kidney function remains. Chronic kidney disease is generally defined by kidney damage markers or eGFR below 60 that persist for at least 3 months.

You should arrange follow up promptly, especially if the protein is moderate/high, persistent, or you also have diabetes, high blood pressure, swelling, foamy urine, blood in urine, or reduced eGFR. Seek urgent care if you have severe swelling, shortness of breath, very little urine, chest pain, confusion, or very high blood pressure.

model: Gemini

**answer:**

Finding out you have high protein in your urine (a condition called proteinuria) can definitely be scary, but the short answer is no it does not automatically mean your kidneys are failing.

While it is a sign that your kidneys are under stress or experiencing some damage, it is very often an early warning sign, not a late stage one. In fact, catching it early gives you and your doctor a chance to intervene and protect your kidney function before serious failure occurs.

Here is a breakdown of what might be happening and what it means.

How Protein Ends Up in Urine

Your kidneys act like coffee filters for your blood. They filter out waste and extra water to make urine, but they are designed to keep important, large molecules like protein in your bloodstream.

If those filters (called glomeruli) are inflamed, stressed, or damaged, they can become "leaky," allowing protein to slip through into your urine.

Temporary vs. Persistent Causes

Protein in the urine isn't always permanent. It can be categorized into two main buckets:

1. Temporary (Benign) Causes

Sometimes, healthy kidneys leak a little protein due to temporary physical stress. If you take another test in a few weeks, the protein might be completely gone. Common temporary causes include:

Dehydration or not drinking enough water.

Intense exercise or heavy physical exertion shortly before the test.

Fever or a recent infection.

Urinary tract infections (UTIs).

High physical or emotional stress.

2. Persistent Causes

If the protein shows up on multiple tests over a few months, it points to an underlying issue that needs to be managed. Even in these cases, it doesn't mean your kidneys have failed just that they need help. Common persistent causes include:

High blood pressure (Hypertension): The leading cause of kidney stress. High pressure damages the delicate blood vessels in the kidneys over time.

Diabetes: High blood sugar can overwork the kidneys' filters.

Glomerulonephritis: Inflammation of the kidney's filtering units.

Certain medications: Long term use of certain drugs, like NSAIDs (ibuprofen), can stress the kidneys.

What You Should Do Next

The absolute best next step is to follow up with your doctor. Because a single test doesn't tell the whole story, they will likely want to:

Repeat the test: They will usually do another urinalysis in a few weeks to see if the protein was just a temporary spike.

Check your eGFR: This stands for estimated Glomerular Filtration Rate. It's a simple blood test that tells you exactly how well your kidneys are functioning as a percentage. This is the true measure of kidney failure not just the presence of protein.

Check your blood pressure and blood sugar: Since these are the two biggest culprits for kidney stress, getting them under control can often stop or slow down protein leakage.

Try to take a deep breath. High protein is a check engine light, not a sign that the engine has already broken down.

model: DeepSeek

**answer:**

It's understandable to be worried, but not automatically. A high urine protein level (proteinuria) is a sign that your kidneys may be under stress, but it does not always mean they are "failing" in the sense of irreversible kidney failure.

Here's a more helpful way to think about it:

Possible causes other than kidney failure: Temporary proteinuria can happen after intense exercise, fever, dehydration, emotional stress, or a urinary tract infection. Orthostatic proteinuria (protein only when upright) is harmless and common in young people.

Early kidney damage, not failure: Conditions like early diabetic kidney disease or mild hypertension related kidney changes can cause proteinuria without significantly reducing kidney function (eGFR). This is often called chronic kidney disease (CKD) stage 1 or 2 not failure.

When it suggests failing kidneys: If proteinuria is heavy (e.g, greater than 3 to 3.5g/day) and your eGFR is persistently low ( less than 30 to 60 mL/min) for months, that points to significant kidney dysfunction. But "failure" usually refers to eGFR less than 15 or needing dialysis.

Acute kidney injury (AKI): Sudden rises in protein with a rapid drop in urine output and rising creatinine could signal acute injury, which may be reversible if treated quickly.

Key point: Kidney failure is about loss of filtration function (measured by blood creatinine and eGFR). Proteinuria is more often a marker of damage or leakiness in the kidney filters which can exist without failure. Many people live for decades with mild proteinuria and stable kidney function.

What you should do:

Get a urine albumin to creatinine ratio (UACR) or protein to creatinine ratio (UPCR) to quantify the level.

Check blood creatinine, eGFR, and blood pressure.

Ask your doctor if imaging or a kidney biopsy is needed, depending on the amount of protein and other findings.

In short: High urine protein alone does not mean your kidneys are already failing, but it does mean you need a medical workup to find the cause and protect your kidney health long term.

model: Doubao

**answer:**

High urine protein does not equal kidney failure, but it is an important warning sign of kidney damage. Here's a clear breakdown:

1. The key difference

Proteinuria (high urine protein): A symptom. It means the kidney's filtering function is impaired, letting protein leak into urine. Many mild or early kidney problems cause this.

Kidney failure: A severe end stage condition. It means most kidney tissue has lost function, accompanied by sharply rising blood creatinine, urea, electrolyte disorders, swelling, fatigue, etc.

2. Common causes of high urine protein (not just severe kidney disease)

Temporary factors: Strenuous exercise, fever, dehydration, cold stress, or excessive protein intake. This type is usually mild and reversible.

Early stage kidney diseases: Mild glomerulonephritis, hypertensive kidney damage, diabetic kidney disease (early phase). With timely intervention, progression can be controlled well.

Other issues: Urinary tract infection can also lead to slight protein elevation.

3. What you should do next

Repeat the test: Do a 24 hour urine protein quantification or urine microalbumin test to check the exact amount. Trace or mild proteinuria is often manageable.

Check related indicators: Test blood creatinine, blood urea nitrogen, blood sugar and blood pressure to comprehensively evaluate kidney function.

See a nephrologist: Identify the root cause early. Most cases of early proteinuria can be improved with lifestyle adjustments and standard treatment.

In short, take it seriously but don't panic. Early detection and treatment can effectively protect your kidneys.

model: Kimi

**answer:**

Not necessarily. High urine protein (proteinuria) is a warning sign, not a definitive diagnosis of kidney failure. Here's what it actually means:

What High Urine Protein Indicates

Protein in urine means your kidneys' filtering system (the glomeruli) are allowing protein to leak through, which they normally shouldn't. This suggests kidney damage or stress, but the severity varies widely:

Mild elevation (trace to 1 plus ): Can be temporary from exercise, dehydration, fever, urinary tract infection, or even standing for long periods (orthostatic proteinuria)

Moderate to high (2 plus to 4 plus ): More concerning, often indicates ongoing kidney disease

The Spectrum of Kidney Disease

Kidney conditions progress in stages:

Stage Description eGFR Range

1 Kidney damage with normal function greater than or equal to 90

2 Mild decrease in function 60 to 89

3a Mild to moderate decrease 45 to 59

3b Moderate to severe decrease 30 to 44

4 Severe decrease 15 to 29

5 Kidney failure (dialysis/transplant needed) less than 15

High urine protein can appear in Stage 1 or 2 long before kidneys are "failing." In fact, catching it early is exactly what allows doctors to slow or stop progression.

What You Should Do Next

Confirm the finding A single test isn't enough. Your doctor will likely order:

Repeat urinalysis

Urine albumin to creatinine ratio (UACR)

Blood tests for creatinine and eGFR

Possibly a 24 hour urine collection

Identify the cause Common causes include diabetes, high blood pressure, glomerulonephritis, certain medications (like NSAIDs), and autoimmune conditions

Monitor and manage If confirmed, treatment focuses on controlling blood pressure, blood sugar, and reducing protein loss with medications like ACE inhibitors or ARBs

Bottom Line

High urine protein means your kidneys need attention, but it does not mean they are failing. Many people with proteinuria live with stable kidney function for decades with proper management. However, it should never be ignored.

Please consult a healthcare professional for proper evaluation and personalized guidance. If you have test results in hand, bring them to your doctor to discuss what they mean for your specific situation.

model: Qwen

**answer:**

Don't panic elevated urine protein does not necessarily mean kidney failure.

It is a warning sign indicating potential kidney damage, but the condition is often reversible or manageable in its early stages.

The Function of the Kidneys

K2 greater than Urine protein indicates that the kidneys have filtered out excess protein, which is a sign of damage from causes like diabetes or hypertension. However, kidney failure is defined by a significant drop in the kidneys' ability to filter waste, measured by blood creatinine levels.

Common Causes of Proteinuria

Physiological Factors: Temporary increases can occur due to strenuous exercise, high fever, dehydration, or extreme emotional stress. These usually resolve on their own once the trigger is removed.

Early Stage Kidney Disease: The most common causes include diabetic kidney disease, hypertensive nephropathy, or glomerulonephritis. In these stages, the kidneys are damaged but still functioning effectively.

Other Medical Conditions: Urinary tract infections or certain autoimmune diseases like lupus can also cause protein to leak into the urine.

Next Steps for Evaluation

Repeat Testing: A single positive result is rarely enough for a diagnosis. Doctors typically recommend repeating the urinalysis using a first morning urine sample to rule out temporary factors.

Advanced Urine Tests: A 24 hour urine collection or the urine albumin creatinine ratio (UACR) is often used to accurately measure the amount of protein being excreted.

Blood Tests: A blood test to check creatinine and the estimated glomerular filtration rate (eGFR) is necessary to determine the actual filtering capacity of the kidneys.

Proactive Management

Control Underlying Conditions: Strictly managing blood sugar and blood pressure is the most effective way to slow or reverse early kidney damage.

Lifestyle Adjustments: Reducing salt intake, staying hydrated, and avoiding unnecessary use of non steroidal anti inflammatory drugs (NSAIDs) can help protect kidney health.

Elevated urine protein should be treated as an early warning sign that warrants further investigation rather than a diagnosis of kidney failure. Consulting a nephrologist for comprehensive testing is the best course of action to identify the underlying cause and initiate appropriate treatment.

**Dimension 2-Risk factors**

**Q6. Who is more likely to get diabetic kidney disease?**

model: ChatGPT

**answer:**

People with diabetes are most likely to get diabetic kidney disease, especially when diabetes has been present for many years. About 1 in 3 adults with diabetes has kidney disease.

Risk is higher if you have diabetes and also have:

High blood sugar over time

High blood pressure

Longer duration of diabetes

Smoking

High salt diet or difficulty following a diabetes eating plan

Physical inactivity

Overweight or obesity

Heart disease

Family history of kidney failure

Some groups, including African Americans, American Indians, and Hispanics/Latinos, have higher rates of diabetes, kidney disease, and kidney failure, partly because of higher rates of risk factors and unequal access to care.

A key point: early diabetic kidney disease often has no symptoms, so people with type 2 diabetes should usually be checked yearly, and people with type 1 diabetes should be checked yearly after having diabetes for more than 5 years. Testing usually includes a urine albumin test and a blood test for kidney filtration function.

model: Gemini

**answer:**

Diabetic kidney disease (DKD), also known as diabetic nephropathy, affects roughly 1 in 3 adults living with diabetes. While anyone with type 1 or type 2 diabetes can develop it, certain factors drastically increase the likelihood.

Medical experts generally split these risks into two categories: things you can change or manage (modifiable) and things you cannot (non modifiable).

High Risk Categories

1. Key Medical Drivers (Modifiable)

High Blood Sugar (Hyperglycaemia): Chronic high blood sugar stresses the kidneys' delicate filtering systems over time. Keeping blood glucose levels steady is the primary defense.

High Blood Pressure (Hypertension): This is a massive factor. High blood pressure strains the small blood vessels inside your kidneys, accelerating damage.

Obesity and High Cholesterol (Dyslipidaemia): Extra weight and high levels of blood lipids (fats) increase metabolic stress on the kidneys.

Smoking: Smoking damages blood vessels throughout the entire body, including the renal arteries that supply your kidneys.

2. Time and Genetics (Non Modifiable)

Duration of Diabetes: The longer you have lived with diabetes, the higher the cumulative risk to your kidneys.

Family History: A genetic predisposition to kidney disease or high blood pressure makes you significantly more vulnerable.

Age and Sex: Risk generally increases with age, and statistically, men are slightly more prone to developing DKD than women.

3. Demographic Disparities

Due to a complex mix of genetics, socio economic barriers, and systemic healthcare access issues, certain racial and ethnic groups experience much higher rates of diabetic kidney disease:

African Americans

Hispanic/Latino Americans

American Indians/Alaska Natives

Asian Americans

The Good News: Because the heaviest risk factors like blood sugar, blood pressure, and smoking are modifiable, proactive management can significantly slow down or even prevent the progression of kidney damage.

model: DeepSeek

**answer:**

That's an excellent and important question. The likelihood of developing diabetic kidney disease (DKD), also known as diabetic nephropathy, isn't a simple matter of one type of diabetes versus another. Instead, it depends on a complex interaction of factors.

However, if we look at population statistics, people with Type 2 diabetes (T2D) account for the vast majority of cases of diabetic kidney disease. This is simply because Type 2 diabetes is far more common (90 to 95% of all diabetes cases).

But if we look at individual risk based on diabetes type, the picture is more nuanced.

Comparing Risk: Type 1 vs. Type 2 Diabetes

Factor Type 1 Diabetes (T1D) Type 2 Diabetes (T2D)

Lifetime Risk Historically about 30 to 40%, now declining significantly with better glucose control. about 30 to 50%, but varies widely due to other risk factors.

Progression Speed Often slower progression from microalbuminuria to end stage renal disease (ESRD). Often faster progression, partly due to older age and more cardiovascular risk factors.

Key Onset Factor Primarily driven by long duration of high blood glucose. A combination of glucose control, high blood pressure, obesity, and insulin resistance (often present at diagnosis).

Role of Genetics Strong genetic component, but different genes may be involved than in T2D. Very strong genetic component (e.g, variants in the APOL1 gene in African ancestry).

The Real Answer: Who is at HIGHEST risk?

The people most likely to get diabetic kidney disease are not defined just by diabetes type, but by the presence of multiple major risk factors. A person with Type 2 diabetes who has several of these is at far greater risk than a person with Type 1 diabetes with none of them.

The strongest predictors of DKD are:

Poor Blood Glucose Control (High HbA1c): This is the 1 modifiable risk factor for both types. Consistently high blood sugar damages the delicate filtering units (nephrons) of the kidneys.

High Blood Pressure (Hypertension): This is the 1 accelerant. High pressure damages the blood vessels in the kidneys. The two conditions (diabetes and hypertension) are a devastating combination for kidneys.

Long Duration of Diabetes: The longer you have diabetes (especially over 10 to 15 years), the higher the cumulative risk of damage.

Ethnicity: This is a major factor for Type 2 diabetes.

Highest risk groups: People of African American, Hispanic/Latino, Native American (especially Pima), and Asian American (particularly South Asian) descent have a significantly higher risk of DKD.

Family History of Kidney Disease: Having a parent or sibling with diabetic kidney disease or other forms of chronic kidney disease (CKD) greatly increases your own risk, indicating a strong genetic predisposition.

Smoking: Tobacco use directly damages blood vessels and worsens both high blood pressure and kidney damage.

Obesity and Insulin Resistance: Common in Type 2 diabetes, these contribute to a pro inflammatory state that harms the kidneys.

High Cholesterol (Dyslipidemia): High triglycerides and LDL ("bad") cholesterol contribute to vascular damage in the kidneys.

Conclusion: Who is more likely?

By sheer numbers: People with Type 2 diabetes are more likely to be diagnosed with DKD because there are so many more of them.

By individual risk: A person with Type 2 diabetes who also has high blood pressure, is a smoker, has poor glucose control, and is of African American or Hispanic descent is extremely likely to develop DKD.

A person with Type 1 diabetes who has maintained excellent blood glucose control (e.g, near normal HbA1c) and normal blood pressure for 40 years has a low risk.

The most important takeaway: The question shouldn't just be "who is more likely?" but "what can be done?" Modern medicine is very effective at preventing or delaying DKD in both types of diabetes through:

Rigorous blood sugar management (using medications like SGLT2 inhibitors or GLP 1 agonists which also protect kidneys).

Strict blood pressure control (using ACE inhibitors or ARBs as first line drugs).

Lifestyle changes (diet, exercise, smoking cessation).

If you or someone you know has diabetes, the best action is to ask their doctor for two simple tests:

Urine Albumin to Creatinine Ratio (UACR): Checks for protein leakage.

Blood test for eGFR: Checks how well the kidneys are filtering.

Catching DKD early is the key to preventing it from progressing to kidney failure.

model: Doubao

**answer:**

People in the following groups have a higher risk of developing diabetic kidney disease:

Long term diabetes patients

Those with diabetes for more than 10 years are at notably higher risk.

Poor blood sugar control

Persistently high blood glucose greatly damages kidney blood vessels over time.

Uncontrolled high blood pressure

Hypertension is a major accelerator of kidney damage in diabetics.

Older adults

Advanced age weakens kidney function and raises disease risk.

People with high blood lipids / obesity

Excess fat and abnormal lipids worsen vascular and kidney injuries.

Smokers and heavy drinkers

Tobacco and alcohol impair renal blood circulation.

Family history of kidney disease

Genetic susceptibility increases the chance of DKD.

Type 1 diabetes patients

Type 1 diabetics tend to develop kidney complications earlier than most Type 2 patients.

model: Kimi

**answer:**

People with diabetes are more likely to develop diabetic kidney disease when they have had diabetes for many years, especially more than 10–15 years, or when their blood sugar is poorly controlled. The risk is also higher with high blood pressure, obesity, metabolic syndrome, high cholesterol or triglycerides, heart disease, smoking, older age, or a family history of kidney disease or diabetes.

Some ethnic groups, including African American, Hispanic/Latino, Native American, Asian American, and Pacific Islander populations, may also have a higher risk. Certain genetic factors, such as APOL1 variants, can increase susceptibility.

The risk can be reduced by controlling blood sugar and blood pressure, maintaining a healthy weight, stopping smoking, taking kidney-protective medicines when prescribed, and having regular kidney tests, including urine albumin and eGFR tests. A healthcare professional can assess an individual’s risk using their medical history and test results.

model: Qwen

**answer:**

Long duration diabetes with poor control is the primary risk. The likelihood increases with age, co existing hypertension, obesity, smoking, and a family history of the disease.

Primary Risk Factors

Duration of Diabetes: The longer a person lives with diabetes, the higher the risk. For Type 1 diabetes, significant kidney damage typically appears after 10 years. For Type 2, kidney issues may already be present at the time of diagnosis.

Poor Glycemic Control: Consistently high blood sugar damages the tiny filtering units (glomeruli) in the kidneys, causing them to leak protein into the urine and eventually lose function.

Hypertension: High blood pressure is often found alongside diabetes. It puts extra strain on the kidney's delicate blood vessels, accelerating damage.

Obesity and Metabolic Issues: Being overweight, along with high cholesterol (especially triglycerides) and high uric acid, increases insulin resistance and alters kidney blood flow, making kidney disease more likely.

Secondary Risk Factors

Genetics and Family History: Some people are naturally more susceptible due to their genetic makeup. Having a family history of diabetes or kidney disease raises personal risk.

Lifestyle Choices: Long term smoking significantly accelerates the decline of kidney function in diabetic patients.

Age and Race: The risk increases with age. Certain ethnic groups, including African American, Hispanic, Native American, and Asian populations, have a higher predisposition to developing this condition.

Prevention and Early Detection

Regular Screening: People with Type 1 diabetes should start screening 5 years after diagnosis, while those with Type 2 should be screened immediately upon diagnosis. Annual tests for urine albumin and estimated glomerular filtration rate (eGFR) are essential.

Holistic Management: Strict control of blood sugar, blood pressure, and lipids is the most effective way to prevent kidney damage. In some cases, doctors may prescribe specific medications, such as SGLT2 inhibitors or ACE inhibitors, which offer direct kidney protection beyond just lowering glucose or blood pressure.

In summary, while a long history of diabetes combined with high blood pressure and poor blood sugar control creates the highest risk, the condition is largely preventable. Consistent management of metabolic factors and regular medical checkups are key to protecting kidney health.

**Q7. Does high blood pressure make diabetic kidney disease worse?**

model: ChatGPT

**answer:**

Yes. High blood pressure can make diabetic kidney disease worse.

In diabetic kidney disease, high blood sugar can damage the small blood vessels in the kidneys. High blood pressure adds extra force on those already vulnerable vessels, which can reduce kidney blood flow, increase protein leakage into the urine, and speed up loss of kidney function. NIDDK specifically notes that many people with diabetes also develop high blood pressure, which can further damage the kidneys.

It can also become a vicious cycle: damaged kidneys retain extra fluid and salt, which raises blood pressure even more, causing further kidney damage. NIDDK states that "no matter what the cause" of kidney disease, high blood pressure can make the kidneys worse.

The good news is that controlling blood pressure is one of the most important ways to slow DKD progression. Doctors often use ACE inhibitors or ARBs medicines ending in pril or sartan because they can lower blood pressure and help protect the kidneys, especially when albumin/protein is present in the urine.

A practical takeaway: people with diabetic kidney disease should regularly monitor blood pressure, urine albumin to creatinine ratio, and eGFR, and ask their clinician what individualized blood pressure goal is safest for them.

model: Gemini

**answer:**

Yes, absolutely. High blood pressure (hypertension) is one of the primary factors that makes diabetic kidney disease progress faster. When you have both conditions, they compound each other's effects, accelerating damage to the kidneys much more rapidly than either condition would on its own. In fact, keeping blood pressure tightly controlled is one of the most effective ways to slow or even prevent the progression of diabetic kidney disease. Here is why they are such a destructive combination.

How the Damage HappensYour kidneys are filled with millions of microscopic filtering units called nephrons. Inside each nephron is a cluster of tiny, delicate blood vessels called a glomerulus, which is responsible for filtering waste out of your blood. The Diabetes Factor: Persistently high blood sugar levels physically damage these tiny blood vessels over time, causing them to thicken, scar, and become leaky. This allows proteins that should stay in your blood (like albumin) to leak into your urine. The High Blood Pressure Factor: High blood pressure means blood is pumping through your body with excessive force. When this high pressure blood slams into the already weakened, scarred glomeruli, it stretches and damages them even further. Imagine a garden hose that has become brittle and stiff (from diabetes). If you turn the water pressure up to the maximum (high blood pressure), that hose is going to wear out and spring leaks much faster.The Vicious CycleThe relationship between your kidneys and your blood pressure goes both ways, creating a dangerous feedback loop if left untreated: High blood pressure damages the kidneys' filtering ability. As the kidneys become damaged, they lose their ability to properly regulate salt and fluid levels in your body. The damaged kidneys mistakenly release hormones (like renin) that tell your blood vessels to constrict.The extra fluid retention and constricted vessels drive your blood pressure even higher, which in turn causes more kidney damage. Breaking the CycleBecause high blood pressure is so harmful to diabetic kidneys, the blood pressure targets for someone with diabetic kidney disease are often stricter frequently aiming for less than 130/80 mmHg. The good news is that this cycle can be slowed down significantly:Targeted Medications: Doctors frequently prescribe specific blood pressure medications namely ACE inhibitors or ARBs (Angiotensin Receptor Blockers). These drugs don't just lower blood pressure system wide; they specifically relax the blood vessels inside the kidney, reducing the pressure directly on the glomeruli. Sodium Reduction: Because damaged kidneys struggle to process sodium, eating a low sodium diet is crucial for keeping blood pressure manageable.Newer Therapies: Medications like SGLT2 inhibitors (originally used just for blood sugar) have recently been shown to offer incredible protection for the kidneys as well.

model: DeepSeek

**answer:**

Yes, high blood pressure can significantly worsen diabetic kidney disease. It increases pressure inside the kidney’s filtering units, damaging the filtration barrier, increasing protein leakage into the urine, and accelerating the decline in kidney function.

High blood pressure can also damage the blood vessels in the kidneys and promote scarring, reducing the kidneys’ ability to filter waste. When high blood sugar and high blood pressure occur together, they trigger inflammation, oxidative stress, and hormonal changes that further accelerate kidney damage.

Therefore, controlling blood pressure is essential for protecting kidney function and slowing the progression of diabetic kidney disease. For people with diabetes and chronic kidney disease, blood pressure is often targeted below 130/80 mmHg, although the exact goal should be individualized by a healthcare professional. Medications such as ACE inhibitors or ARBs can lower blood pressure, reduce proteinuria, and provide additional kidney protection.

model: Doubao

**answer:**

Yes.

High blood pressure is one of the most critical risk factors that accelerates the progression of diabetic kidney disease (DKD).

Persistent high pressure damages the tiny blood vessels and filtration units in the kidneys, worsening protein leakage and kidney function decline.

Poorly controlled hypertension greatly increases the risk of developing end stage renal disease.

Strict blood pressure management is a core part of treating and slowing diabetic kidney damage.

model: Kimi

**answer:**

Yes. High blood pressure (hypertension) is one of the most significant factors that can worsen diabetic kidney disease (diabetic nephropathy).

Here's why:

The damaging cycle

Diabetes already damages the small blood vessels in the kidneys (the glomeruli), making them less effective at filtering waste

High blood pressure adds mechanical stress to these already weakened vessels, accelerating the damage

As kidney function declines, blood pressure often rises further, creating a vicious cycle

What actually happens

Increased pressure inside the kidney's filtering units causes them to leak more protein (albumin) into the urine a key marker of worsening kidney disease

Over time, this leads to scarring (fibrosis) and permanent loss of kidney function

Hypertension also promotes inflammation and oxidative stress in kidney tissue

The clinical reality

Most guidelines recommend stricter blood pressure targets for people with diabetes and kidney disease than for the general population

Controlling blood pressure is one of the most effective ways to slow progression of diabetic kidney disease

ACE inhibitors or ARBs are often preferred medications because they both lower blood pressure and provide specific kidney protection

Bottom line: If you have diabetes, monitoring and controlling blood pressure is just as important as controlling blood sugar for protecting your kidneys over the long term.

Note: This is general medical information. For personal health concerns or treatment decisions, please consult your healthcare provider.

model: Qwen

**answer:**

Yes, high blood pressure significantly worsens diabetic kidney disease. The two conditions create a vicious cycle that accelerates kidney damage.

How Hypertension Accelerates Kidney Damage

Increased Glomerular Pressure: High blood pressure transmits excessive force to the glomerular capillaries, keeping them in a state of hyperfiltration and high pressure. This directly damages the filtering units.

Vascular Hardening: Chronic hypertension causes thickening and narrowing of the small arteries supplying the kidneys. This reduces blood flow and oxygen, leading to ischemic injury and fibrosis.

The Vicious Cycle

Diabetes damages the kidney's microvessels, impairing the organ's ability to regulate blood pressure. In turn, elevated blood pressure worsens vascular injury and increases protein leakage. This feedback loop rapidly pushes the disease toward advanced stages.

Core Management Strategies

Strict Blood Pressure Control: The target for patients with diabetes and kidney involvement is generally less than 130/80 mmHg.

Kidney Protective Medications: ACE inhibitors (ACEI) or angiotensin receptor blockers (ARB) are the first line choice. They lower systemic pressure while specifically reducing intraglomerular pressure and urinary protein.

Regular Monitoring: Early detection is critical. Annual screening for urine albumin to creatinine ratio (UACR) and estimated glomerular filtration rate (eGFR) is essential to catch changes before they become irreversible.

Controlling blood pressure is arguably as important as managing blood sugar in slowing the progression of diabetic kidney disease. Consistent monitoring and adherence to prescribed therapy can significantly preserve long term kidney function.

**Q8. Does poor blood sugar control increase my chance of kidney damage?**

model: ChatGPT

**answer:**

Yes. Poor blood sugar control does increase the chance of kidney damage, especially when high glucose continues over months to years. High blood sugar can damage the small blood vessels in the kidneys, making them filter less effectively; high blood pressure often adds further damage.

The main warning signs doctors screen for are albumin in the urine and a reduced eGFR blood test result. Many people have no symptoms early on, so kidney checks are important even when you feel well. NIDDK notes yearly kidney testing for people with type 2 diabetes, and for people with type 1 diabetes after more than 5 years.

The good news is that kidney damage risk can be reduced or delayed by improving blood sugar control, controlling blood pressure, not smoking, limiting excess salt, staying active, and taking prescribed medicines. For many adults with diabetes, an A1C goal is around below 7%, but the right goal depends on age, kidney function, hypoglycemia risk, pregnancy status, and other conditions.

Ask your clinician about these two tests: urine albumin to creatinine ratio, or UACR, and serum creatinine with eGFR. A UACR above 30 mg/g can be a sign of kidney damage and should be interpreted with your full clinical picture.

model: Gemini

**answer:**

Yes, poor blood sugar control significantly increases your risk of kidney damage. In fact, diabetes is the leading cause of chronic kidney disease worldwide. When blood sugar levels remain consistently high, it triggers a chain reaction that physically damages the kidneys over time a condition known as diabetic nephropathy or diabetic kidney disease (DKD).

How High Blood Sugar Damages the KidneysYour kidneys contain millions of tiny blood vessel clusters called glomeruli that act as filters, removing waste from your blood while keeping valuable proteins and cells in your body. High blood sugar attacks this filtration system in three main ways:Hyperfiltration (The "Overwork" Phase): High blood sugar changes the osmotic pressure in your kidneys, forcing them to filter more blood than normal. This increased pressure is like running water too forcefully through a delicate sieve it causes physical stress and wear and tear on the filters. Vascular and Cellular Damage: Excess glucose in the bloodstream causes proteins to become "glycated" (sugar coated), which triggers inflammation and oxidative stress. This damages the endothelial cells lining the blood vessels and thickens the basement membrane, making the vessels stiff and narrow. Podocyte Injury: High sugar and pressure specifically damage podocytes specialized cells that wrap around the capillaries and help form the filtration barrier. When podocytes are damaged or die off, the filter becomes leaky. The Domino EffectAs the glomeruli become damaged and leaky, albumin (a vital protein) begins to spill out of the blood and into the urine. This condition, called microalbuminuria, is often the very first clinical sign of diabetic kidney disease. If blood sugar remains uncontrolled, the constant inflammation leads to scarring (fibrosis) within the kidney tissue. Over years or decades, this scarring destroys the kidney's ability to filter waste entirely, eventually leading to kidney failure.Because the kidneys and the cardiovascular system are closely linked, this damage is often accelerated if you also have high blood pressure a common complication for people with diabetes.

model: DeepSeek

**answer:**

Yes, poor blood sugar control significantly increases your chance of kidney damage. This condition is often called diabetic kidney disease (or diabetic nephropathy). Over time, high blood sugar levels can damage the tiny blood vessels in your kidneys (the glomeruli), which are responsible for filtering waste from your blood. Once these vessels are damaged, they become leaky and less efficient, potentially leading to chronic kidney disease or even kidney failure.

The risk rises with:

Consistently high HbA1c levels

Long duration of diabetes

Also having high blood pressure (which often coexists with poor sugar control)

The good news is that improving blood sugar control through medication, diet, exercise, and regular monitoring can significantly lower this risk and slow or prevent further kidney damage if caught early. Routine urine tests (like checking for albumin) and blood tests (like eGFR) help detect kidney damage at a reversible stage. If you have diabetes, talk to your doctor about your specific kidney risk and how to manage it.

model: Doubao

**answer:**

Yes, it definitely does. Long term high blood sugar is a major cause of kidney damage, especially diabetic kidney disease.

High blood sugar damages the tiny blood vessels and filtering units in the kidneys over time. Gradually, the kidneys lose their ability to filter waste from the blood, which may eventually lead to serious kidney problems or even kidney failure.

Keeping blood sugar within a healthy range is one of the most effective ways to protect your kidneys.

model: Kimi

**answer:**

Yes. Poor blood sugar control is one of the leading causes of diabetic kidney disease (also called diabetic nephropathy).

How it happens

When blood sugar stays high over time, it damages the tiny blood vessels (glomeruli) in your kidneys that filter waste from your blood. This can cause:

Protein leaking into urine (microalbuminuria, often the first sign)

Reduced kidney function over time

Chronic kidney disease (CKD) and, in severe cases, kidney failure requiring dialysis or transplant

What the research shows

Diabetes is the 1 cause of kidney failure in many countries.

The risk rises significantly with higher HbA1c levels (a measure of average blood sugar over 2 to 3 months).

Other factors that compound the risk include high blood pressure, smoking, and longer duration of diabetes.

The good news

Tight blood sugar control significantly lowers risk. Studies show that every 1% reduction in HbA1c reduces the risk of diabetic complications, including kidney disease. Blood pressure control (especially with ACE inhibitors or ARBs) and regular monitoring of urine albumin also help protect kidney function.

Important: If you have diabetes or prediabetes, regular screening (urine albumin and eGFR blood tests) is key to catching problems early. For personalized advice, please speak with your healthcare provider.

model: Qwen

**answer:**

Yes, poor blood sugar control significantly increases the risk of kidney damage. Chronic high blood sugar injures the kidneys' microscopic filtering vessels, leading to protein leakage and eventual decline in renal function.

How High Blood Sugar Damages the Kidneys

Kidney Overload and Vessel Damage: The kidneys act as the body's "filter." Long term high blood sugar forces them to work overtime. This sustained high pressure state enlarges the tiny filtering vessels, causing the "mesh" to develop leaks.

Protein Leakage (Proteinuria): When these filters are stretched, high quality proteins that should remain in the blood begin to leak into the urine.

Toxin Buildup and Decline: As vessel damage worsens, the kidneys gradually lose the ability to filter waste. Toxins accumulate in the blood, potentially progressing to kidney failure (uremia).

Early Warning Signs to Watch For

Changes in Urine: Noticeable foam or bubbles in the urine in the morning that do not dissipate quickly.

Swelling: Puffiness around the eyes in the morning or swelling in the legs and ankles during the day.

Other Symptoms: Unexplained fatigue, abnormal blood pressure spikes, or an increase in nighttime urination.

Specific Steps to Protect Kidney Health

Set Blood Sugar Targets: Keep Glycated Hemoglobin (HbA1c) below 7%. This is the key standard for preventing diabetic kidney disease.

Regular Screening: Conduct annual urine tests for microalbumin and blood tests for Glomerular Filtration Rate (GFR) to identify abnormalities early.

Comprehensive Management: Control blood pressure and blood lipids strictly. High blood pressure and lipids accelerate kidney damage exponentially and must be managed alongside blood sugar.

Reasonable Diet: Limit high protein intake appropriately, prioritizing high quality proteins like fish, eggs, and soybeans to reduce the filtration burden on the kidneys.

In summary, maintaining stable and well controlled blood sugar is the core defense against kidney damage. By managing blood sugar, blood pressure, and lipids effectively, early kidney injury can be significantly delayed or even reversed.

**Q9. If my parent had kidney disease, am I more likely to get kidney problems from diabetes?**

model: ChatGPT

**answer:**

Yes a family history of kidney disease can increase your risk, but it does not mean you will definitely get kidney problems from diabetes.

The risk depends on why your parent had kidney disease:

If your parent's kidney disease was from diabetes or high blood pressure, you may have a higher risk because diabetes, high blood pressure, and kidney disease can run in families and share lifestyle/genetic risk factors. Diabetes and high blood pressure are also major causes of chronic kidney disease.

If your parent had an inherited kidney disease, such as autosomal dominant polycystic kidney disease, your risk may be much higher; this condition can be inherited from one affected parent.

If you have diabetes, the most important things are regular kidney checks and risk control. Diabetic kidney disease often has no symptoms early on, so doctors check it with a urine albumin test and a blood test for eGFR. The American Diabetes Association recommends monitoring albuminuria and eGFR at least annually in people with diabetes.

What you can do now: ask your clinician for uACR and eGFR testing, keep blood sugar and blood pressure well controlled, avoid smoking, and tell your doctor clearly: "My parent had kidney disease should I be screened more often?"

model: Gemini

**answer:**

Yes. If your parent had kidney disease, you are at a higher risk of developing diabetic kidney disease (also known as diabetic nephropathy) if you have diabetes.

Wichita Nephrology Group

While diabetes is the leading cause of chronic kidney disease, not everyone with diabetes develops kidney problems.

Having a family history, however, significantly increases your susceptibility.

DaVita Kidney Care

plus 1

Why Family History Matters

The kidneys contain millions of tiny filters called nephrons.

Prolonged high blood sugar from diabetes can damage the blood vessels in these filters, but your genetics strongly influence how vulnerable your nephrons are to that damage.

CDC

plus 1

Inherited Susceptibility: Studies show that diabetic nephropathy tends to cluster in families.

If a parent or close relative had kidney disease especially if theirs was also caused by diabetes or high blood pressure your genetic risk is noticeably higher than a person with diabetes who has no family history.

PMC NIH

Shared Environment: Families do not just share genes; they often share similar diets, activity levels, and lifestyles. These shared habits can influence blood sugar control and overall kidney health.

Other Factors That Compound Risk

Family history is only one piece of the puzzle. The risk to your kidneys increases further if you experience any of the following:

Risk Factor How it Affects the Kidneys

High Blood Pressure Forces blood through the delicate kidney filters at high pressure, causing scarring.

Poor Blood Sugar Control Chronically high glucose levels damage the tiny blood vessels within the nephrons.

Smoking Constricts blood vessels and reduces vital blood flow to the kidneys, accelerating damage.

Duration of Diabetes The longer you have lived with diabetes, the longer your kidneys have been exposed to systemic stress.

How to Protect Your KidneysHaving a genetic predisposition does not guarantee that you will develop kidney disease. Knowing your family history gives you a major advantage because you can take proactive steps to protect your kidney function: Tell your doctor: Make sure your healthcare provider knows about your parent's kidney disease so they can monitor you more closely.Get screened annually: Kidney disease rarely shows symptoms in its early stages. If you have diabetes, you should get a yearly urinalysis (to check for albumin, a protein that leaks into urine when kidneys are damaged) and a blood test to check your GFR (glomerular filtration rate, which measures how well your kidneys filter waste). Manage your numbers: Keeping your blood sugar and blood pressure within your target ranges is the single most effective way to prevent or delay diabetic kidney disease.Protect your heart: The cardiovascular and renal systems are deeply connected. Eating a low sodium diet, staying active, and avoiding smoking protect both your heart and your kidneys.

model: DeepSeek

**answer:**

The short answer is yes, having a parent with kidney disease may increase your risk of developing kidney problems if you have diabetes but the reason is likely a combination of genetics and shared family factors, not a direct rule.

Here's a more detailed breakdown:

1. Genetics play a role in both diabetes and kidney disease

Family history of kidney disease (especially from causes like high blood pressure or diabetes) can mean you inherit genes that make your kidneys more vulnerable to injury over time.

Type 2 diabetes itself has a strong genetic component. If your parent had diabetes related kidney disease, you may also have a higher chance of developing diabetes, which then increases kidney risk.

2. Diabetes is the leading cause of kidney disease

In people with diabetes, high blood sugar damages the small blood vessels in the kidneys. This condition is called diabetic kidney disease (or diabetic nephropathy).

If you already have a family history of chronic kidney disease, that background risk adds to the risk from diabetes.

3. Shared family factors (not just genes)

Families often share lifestyle habits (diet, exercise patterns) and sometimes undiagnosed mild high blood pressure or obesity, all of which affect kidney health. These can make kidney problems from diabetes more likely.

4. What does the research say?

Studies show that people with diabetes who have a first degree relative (parent or sibling) with chronic kidney disease even without diabetes have a higher risk of developing kidney disease themselves. When the relative's kidney disease was caused by diabetes, the risk is even higher.

5. Important nuance: not inevitable

Having a parent with kidney disease does not mean you will get kidney problems if you have diabetes. Many people with a family history never develop significant kidney issues. The increased risk is moderate, not absolute.

What you can do to lower your risk

If you have diabetes (or prediabetes) and a family history of kidney disease:

Control blood sugar (HbA1c typically less than 7% or as targeted by your doctor).

Control blood pressure (aim for less than 130/80 mmHg often recommended in diabetes).

Ask your doctor about yearly urine albumin to creatinine ratio (checks for early kidney damage) and blood tests (eGFR).

Avoid NSAIDs (like ibuprofen, naproxen) unless absolutely needed, as they can stress kidneys.

Manage weight, diet (lower sodium, moderate protein if advised), and stop smoking if you smoke.

Bottom line

Yes, you are at somewhat higher risk, but it's not a guarantee. The most powerful influence is how well you manage your diabetes and blood pressure. If you have diabetes and a parent with kidney disease, talk to your doctor about a proactive kidney monitoring plan early detection makes a major difference.

Note: If your parent's kidney disease was from a specific inherited condition (like polycystic kidney disease), that's a different situation you would need genetic testing and specialized follow up.

model: Doubao

**answer:**

Having a parent with kidney disease does increase your risk of developing diabetic kidney disease, and the two factors combine to raise your chances further. Here's a clear breakdown:

1. Why family history matters

Genetic susceptibility: A family history of kidney disease means you may inherit genes that make your kidneys more vulnerable to damage. These genes can affect how your kidneys filter blood and resist stress.

Shared lifestyle habits (diet, activity, blood pressure tendencies) within families also add to the risk.

2. Diabetes plus family kidney history is higher combined risk

Diabetes is the leading cause of kidney disease worldwide. High blood sugar gradually damages the tiny blood vessels and filters in the kidneys.

If you have both:

Diabetes

A parent with kidney disease

Your risk of diabetic kidney disease is noticeably higher than someone with only one of these factors.

3. Key preventive steps (most important)

You can greatly lower risk with consistent management:

Keep blood sugar well controlled (follow your doctor's target levels).

Manage blood pressure strictly (high pressure worsens kidney damage; aim for normal ranges).

Limit salt, sugary foods, and processed meals; maintain a healthy weight.

Get regular checkups: Test urine for protein and blood work for kidney function yearly.

Avoid overusing painkillers (e.g, certain NSAIDs) that can harm kidneys.

Short summary

A family history of kidney disease makes you more prone to kidney complications if you have diabetes. But with proper disease management and routine screenings, you can effectively protect your kidneys.

model: Kimi

**answer:**

Yes having a parent with kidney disease does increase your likelihood of developing kidney problems from diabetes, though the reasons are a mix of genetics and shared risk factors rather than destiny.

Family history is a recognized risk factor for diabetic kidney disease. Research shows that a family history of kidney disease is specifically linked to an increased risk of diabetic nephropathy, independent of other factors like blood sugar control or blood pressure. Studies have also found familial clustering of diabetic kidney disease, providing evidence that genetic susceptibility plays a role in who develops kidney complications from diabetes.

However, it matters what caused your parent's kidney disease. If your parent had a hereditary kidney disorder like polycystic kidney disease (PKD) or Alport syndrome, your risk comes from a specific inherited gene mutation. But if their kidney disease was caused by diabetes or high blood pressure, your increased risk is partly because those conditions themselves tend to run in families, and partly because there appear to be inherited genetic factors that make some people's kidneys more vulnerable to damage from diabetes.

What this means practically:

Genes are not fate. Family history raises your risk, but it doesn't guarantee you'll develop kidney problems. Lifestyle and management play a huge role.

If you have diabetes or prediabetes, your family history means you should be extra vigilant about blood sugar and blood pressure control, as these are the strongest modifiable protectors of kidney function.

Consider earlier screening. Because of your family history, discussing kidney function monitoring (blood and urine tests) with your doctor is wise, even if you feel fine.

If you're concerned about your specific risk, a nephrologist or your primary care doctor can assess your family history alongside your current health to recommend a personalized monitoring plan.

model: Qwen

**answer:**

Having a parent with kidney disease does increase your risk, but it doesn't guarantee you will develop it. Controlling your blood sugar and blood pressure is the key to prevention.

The Impact of Genetics

Diabetic kidney disease does have a genetic component. If a parent has kidney disease, your risk is higher than average. However, genetics only make you susceptible; lifestyle and disease management are the real triggers.

Key Factors That Matter Most

Your future kidney health depends more on what you can control than your family history:

Blood Sugar Control: Long term high blood sugar damages kidney blood vessels. Keeping your glucose levels in the target range is the most important step.

Blood Pressure Management: High blood pressure puts extra strain on the kidneys. Keeping it below 130/80 mmHg significantly reduces damage.

Lifestyle Choices: Smoking, a high salt diet, and obesity accelerate kidney decline. Quitting smoking and maintaining a healthy weight are crucial.

Practical Prevention Steps

Since you know you have extra risk, you can take proactive steps:

Regular Screenings: Get checked at least once a year for urine albumin (UACR) and kidney function (blood creatinine/eGFR). This is the best way to catch early damage.

Watch for Signs: Be alert to persistent foamy urine or swelling in the face or ankles, as these can be early signs of kidney trouble.

Kidney Friendly Medications: If you have diabetes, certain glucose lowering and blood pressure medications offer direct protection to the kidneys.

In conclusion, a family history is just a warning signal. By strictly managing your blood sugar and blood pressure and getting regular checkups, you can protect your kidneys.

**Q10. Can smoking or drinking alcohol make diabetic kidney disease worse?**

model: ChatGPT

**answer:**

Yes. Smoking definitely can make diabetic kidney disease worse, and heavy or frequent alcohol use can also worsen kidney health indirectly.

Smoking: It raises blood pressure, damages blood vessels, reduces blood flow to the kidneys, and can make existing kidney disease progress faster. NIDDK specifically lists smoking as a risk factor for diabetic kidney disease and recommends stopping smoking to protect the kidneys.

Alcohol: Occasional light drinking is not always forbidden for everyone, but drinking too much can raise blood pressure, add extra calories/weight gain, cause dehydration, and make blood sugar control harder all of which can worsen diabetic kidney disease risk or progression. NIDDK advises limiting alcohol because too much alcohol can increase blood pressure.

For people with diabetes, alcohol also increases the risk of low blood sugar, especially when combined with insulin or sulfonylurea medicines; it is safer to drink with food and avoid drinking on an empty stomach.

A practical answer: quit smoking if possible, avoid binge drinking, and discuss any alcohol use with your diabetes/kidney doctor, especially if you have reduced eGFR, albuminuria, high blood pressure, liver disease, or take insulin.

model: Gemini

**answer:**

Yes, both smoking and heavy alcohol consumption can make diabetic kidney disease (DKD) worse, though smoking is widely considered to be far more aggressively damaging to the kidneys.Here is exactly how each habit impacts the progression of the disease.The Impact of SmokingSmoking is a potent, independent risk factor for accelerating kidney failure in people with diabetes. If a person has DKD, continuing to smoke dramatically speeds up the structural damage to the kidneys. Direct cell toxicity: Recent studies show that nicotine itself is directly toxic to podocytes specialized cells in the kidneys that prevent proteins from leaking into the urine. When these cells are damaged or die off, protein leakage (proteinuria) worsens, which is a primary driver of kidney failure. Vascular damage: Diabetes already damages the delicate blood vessels in the kidney's filtering system (the glomeruli). Smoking compounds this by stiffening blood vessels, introducing inflammatory toxins, and reducing overall blood flow to the kidneys. Blood pressure spikes: Smoking causes immediate surges in blood pressure. High blood pressure forces the already weakened kidneys to work much harder, accelerating scarring and filter damage. Because of this cascade of damage, smoking cessation is considered one of the single most critical interventions to delay the need for dialysis or a transplant in DKD patients.The Impact of AlcoholThe relationship between alcohol and diabetic kidney disease is slightly more nuanced and depends heavily on the amount consumed. Blood pressure elevation: Heavy drinking is a known trigger for chronic high blood pressure. Since controlling blood pressure is the second most important factor in slowing DKD (behind controlling blood sugar), heavy alcohol use actively destroys kidney function over time.Blood sugar instability: Alcohol can cause unpredictable swings in blood glucose sometimes causing severe drops, and other times causing spikes (especially if mixed with sugary beverages). Because DKD is fundamentally driven by high blood sugar damaging the kidney's filters, anything that disrupts control accelerates the disease.The moderation caveat: Some large scale studies suggest that light to moderate drinking (e.g, 1 to 2 drinks occasionally) does not significantly increase the risk of worsening kidney disease compared to heavy drinking. However, because alcohol adds empty calories, stresses the body's filtration systems, and interacts poorly with many diabetes and blood pressure medications, standard medical guidelines recommend strictly limiting intake. The Bottom Line: Quitting smoking is a non negotiable step for protecting remaining kidney function. Alcohol does not need to be entirely eliminated in every case, but it must be kept to a strict minimum to maintain stable blood pressure and blood sugar levels.

model: DeepSeek

**answer:**

Yes, both smoking and drinking alcohol can worsen diabetic kidney disease (DKD). Here's how:

Smoking:

Reduces blood flow to the kidneys Nicotine constricts blood vessels, impairing kidney function and accelerating damage.

Increases blood pressure and albuminuria Smoking raises blood pressure and protein leakage in urine, key drivers of DKD progression.

Promotes oxidative stress and inflammation This damages kidney tissues and reduces the effectiveness of diabetes treatment.

Alcohol:

Impairs blood sugar control Alcohol can cause hypo or hyperglycemia, and chronic heavy use worsens glycemic variability, accelerating DKD.

Raises blood pressure Heavy drinking is linked to hypertension, a major risk factor for kidney decline.

Interacts with medications Alcohol can interfere with diabetes and blood pressure drugs, potentially causing liver strain or dehydration, which harms kidneys.

Key clinical advice:

Smoking cessation is strongly recommended.

Moderate drinking ( less than or equal to 1 drink/day for women, less than or equal to 2 for men) may be acceptable for some stable patients, but heavy/binge drinking is dangerous. Many nephrologists advise avoiding alcohol entirely if kidney function is significantly reduced (e.g, eGFR less than 30 to 45 mL/min) or if blood pressure is poorly controlled.

Always consult your doctor for personalized guidance, as individual risks depend on medications, kidney function stage, and overall health.

model: Doubao

**answer:**

Yes, both smoking and drinking alcohol will significantly worsen diabetic kidney disease (DKD).

Harm of smoking

Smoking damages blood vessels, raises blood pressure, and reduces blood flow to the kidneys. It accelerates kidney function decline and greatly increases the risk of developing end stage renal disease.

Harm of drinking alcohol

Alcohol disrupts blood sugar control, increases the burden on the kidneys and liver. It may also cause dehydration, electrolyte disorders and elevated blood pressure, further damaging renal tissues.

Suggestion

Patients with diabetic kidney disease are advised to quit smoking completely and avoid all alcoholic drinks.

model: Kimi

**answer:**

Yes. Both smoking and excessive alcohol consumption can significantly worsen diabetic kidney disease (DKD). Here's how:

Smoking

Smoking is one of the strongest modifiable risk factors for DKD progression:

Accelerates kidney function decline Smokers with diabetes lose kidney function faster than non smokers

Increases albuminuria Raises protein levels in urine, an early marker of kidney damage

Worsens blood pressure Damages blood vessels and raises blood pressure, adding strain on kidneys

Promotes inflammation and oxidative stress Directly damages kidney tissue and filtering units

Increases cardiovascular risk Heart disease and kidney disease often worsen each other in diabetes

Even smoking a small number of cigarettes can be harmful. Quitting smoking is one of the most impactful steps someone with DKD can take to slow progression.

Alcohol

The relationship is more dose dependent, but excessive alcohol is clearly harmful:

Heavy drinking directly damages kidneys, raises blood pressure, and causes dehydration

Interferes with blood sugar control Can cause dangerous highs or lows, especially when combined with diabetes medications

Increases blood pressure A major driver of kidney damage

May interact with medications Some kidney or diabetes drugs don't mix well with alcohol

Moderate drinking (if any) may carry less risk, but guidelines generally recommend limiting or avoiding alcohol entirely if you have DKD. The safest approach is to discuss alcohol use with a doctor.

Bottom Line

If you have diabetic kidney disease, quitting smoking completely and limiting or avoiding alcohol are two of the most important lifestyle changes to protect your kidneys. These, combined with blood sugar and blood pressure control, can significantly slow disease progression.

This information is for educational purposes. Anyone with DKD should consult their healthcare provider for personalized advice on smoking cessation and alcohol use.

model: Qwen

**answer:**

Yes, smoking and excessive alcohol consumption significantly worsen diabetic kidney disease.

Direct Kidney Damage from Smoking

Accelerated Kidney Function Decline: Harmful substances in tobacco trigger oxidative stress and inflammation, directly damaging the tiny blood vessels in the kidneys. This increases protein leakage in urine and causes a faster drop in the glomerular filtration rate (GFR).

Worsened Blood Vessel Constriction: Smoking activates the sympathetic nervous system, causing blood vessels in the kidneys to constrict. This raises blood pressure and reduces blood supply to the kidneys, creating a vicious cycle.

Multiple Risks from Alcohol

Severe Blood Sugar Fluctuations: Alcohol interferes with glucose metabolism by the liver. For patients using insulin or oral medications, it can cause unpredictable, delayed hypoglycemia, which puts immense stress on vulnerable kidneys.

Increased Metabolic Burden: Alcohol and its byproducts must be excreted through the kidneys. Excessive intake overloads the kidneys and can directly damage the renal tubules.

Loss of Blood Pressure and Lipid Control: Drinking is often associated with high calorie intake, leading to spikes in blood pressure and lipids. These cardiovascular risk factors work alongside diabetes to accelerate kidney damage.

Daily Intervention Recommendations

Quit Smoking Immediately: There is no safe level of smoking. Complete cessation is the most effective non drug method to slow kidney disease progression.

Strictly Limit Alcohol: The medical community does not recommend starting to drink for health reasons. If you have a habit, limit alcohol intake to no more than 25 grams per day for men and 15 grams for women. Never drink on an empty stomach.

Quitting smoking and limiting alcohol are critical treatment steps for preserving kidney function. Adopting these lifestyle changes can significantly improve long term outcomes.

**Dimension 3-Screening and diagnosis**

**Q11. How often should people with diabetes have their kidneys checked?**

model: ChatGPT

**answer:**

Most people with diabetes should have their kidneys checked at least once every year.

This usually means two tests: a urine albumin to creatinine ratio, UACR, to look for protein leakage, and a blood eGFR test, to estimate kidney filtering function. The ADA's 2026 guidance says screening should be done annually for everyone with type 2 diabetes and for people with type 1 diabetes once they have had diabetes for 5 years or longer.

A practical schedule is:

Person with diabetes Kidney check frequency

Type 2 diabetes Every year starting at diagnosis

Type 1 diabetes Every year starting 5 years after diagnosis

Already has albuminuria, reduced eGFR, high blood pressure, or known CKD More often, commonly every 3 to 6 months depending on severity and treatment

More frequent monitoring is important because kidney disease can progress without symptoms, and both ADA and KDIGO guidance supports routine screening in diabetes to detect CKD early.

model: Gemini

**answer:**

If you have diabetes, the standard medical recommendation is to have your kidneys checked at least once a year. Because kidney damage from diabetes (diabetic nephropathy) often has no symptoms in its early stages, routine testing is the only way to catch it early when treatment is most effective. The timing of when these annual checks should begin depends on the type of diabetes you have:

Type of Diabetes When to Start Yearly Kidney Tests

Type 2 Diabetes Immediately upon diagnosis.

Type 1 Diabetes 5 years after diagnosis.

Childhood Diabetes Usually starting around age 10 to 12, after having the condition for 5 years.

What Tests Are Done?Your healthcare provider will typically order two simple tests to check your kidney health:A Blood Test (eGFR): This checks your estimated glomerular filtration rate, which measures how well your kidneys are filtering waste from your blood. A Urine Test (UACR): The urine albumin to creatinine ratio test checks for the presence of albumin (a protein). Healthy kidneys keep protein in the blood, so albumin in the urine is an early sign of kidney damage. Do Some People Need More Frequent Testing?Yes. While once a year is the baseline, your doctor may recommend checking your kidneys more often (such as every 3 to 6 months) if:Your test results show signs of kidney damage.Your blood pressure or blood sugar is consistently high and difficult to manage.You have other risk factors, like a family history of kidney failure or heart disease. If you haven't had your kidneys checked recently, it's a good idea to bring it up with your primary care provider or endocrinologist at your next visit.

model: DeepSeek

**answer:**

For people with diabetes, guidelines generally recommend kidney function checks at least once a year. This typically includes:

Urine test measuring albumin to creatinine ratio (UACR) to detect protein leakage.

Blood test estimating glomerular filtration rate (eGFR) to assess how well the kidneys filter waste.

Frequency may increase if:

Existing kidney disease is present (more frequent monitoring, e.g, every 3 to 6 months).

Urine albumin levels are rising or eGFR is declining rapidly.

Blood pressure or blood glucose control is poor.

Type 1 diabetes: Annual screening usually starts 5 years after diagnosis.

Type 2 diabetes: Annual screening begins at diagnosis (since onset may have preceded diagnosis by years).

Always follow your specific doctor's advice, as individual risk factors (e.g, hypertension, family history of kidney disease) may require more frequent checks.

model: Doubao

**answer:**

General standard guidelines (ADA and Chinese Diabetes Society)

1. Type 2 diabetes

Start screening immediately at diagnosis (many patients already have early kidney damage when found).

If kidney function is normal: test once a year.

If abnormal urine albumin or mild renal impairment: check every 3 to 6 months.

Moderate/severe diabetic kidney disease (DKD): monitor every 1 to 3 months.

2. Type 1 diabetes

Begin annual screening 5 years after diagnosis (kidney damage rarely occurs within the first 5 years).

Normal results: once per year.

Abnormal indicators: shorten interval to 3 to 6 months.

Key tests for diabetic kidney screening

Urine albumin to creatinine ratio (UACR) detects early microalbuminuria (the earliest sign of DKD).

Estimated glomerular filtration rate (eGFR) evaluates overall kidney filtering function.

Special high risk groups (more frequent checks)

Patients with any of the following need testing every 3 to 6 months:

High blood pressure

Poor long term blood sugar control

High blood lipid levels

History of smoking

Family history of kidney disease

Retinopathy (diabetic eye damage, closely linked to kidney injury)

Simple summary

Stable, normal kidney results: once a year

Early kidney damage signs: 3 to 6 months

Advanced diabetic kidney disease: 1 to 3 months

model: Kimi

**answer:**

For people with diabetes, kidney screening recommendations depend on the type of diabetes and whether chronic kidney disease (CKD) is already present. Current guidelines from the American Diabetes Association (ADA) and KDIGO recommend two key tests: urine albumin to creatinine ratio (uACR) to detect kidney damage, and estimated glomerular filtration rate (eGFR) to assess kidney function.

Baseline screening (no known kidney disease):

Type 2 diabetes: Start screening at the time of diagnosis, then at least annually thereafter.

Type 1 diabetes: Start screening 5 years after diagnosis, then at least annually thereafter.

If CKD is already present:

Once kidney disease is identified, monitoring should increase to 1 to 4 times per year depending on the stage and risk of progression. For example, mild disease may need annual checks, while more advanced disease may require monitoring every 1 to 3 months.

Why both tests matter:

Checking only eGFR or only uACR can miss cases of diabetic kidney disease. Some people develop reduced kidney function without albumin in their urine, while others show albumin leakage before eGFR drops. Using both tests together is the standard of care for proper CKD classification.

These are general guidelines your doctor may adjust the frequency based on your individual risk factors, medications, and test results.

model: Qwen

**answer:**

People with diabetes should generally have their kidneys checked at least once a year. 1 year type 1 diabetes should start screening 5 years after diagnosis, and type 2 diabetes should start screening right from diagnosis.

Core Screening Tests

Urinary Albumin to Creatinine Ratio (UACR): This is the most sensitive indicator for detecting early kidney damage, typically using a morning urine sample.

Blood Creatinine (eGFR): Used to assess the overall ability of the kidneys to filter waste from the blood.

Frequency Based on Risk

Standard Risk: For those with normal kidney function, an annual check up is sufficient.

High Risk or Confirmed Damage: If you have already been diagnosed with kidney issues, high blood pressure, or cardiovascular problems, the frequency increases to every 3 to 6 months to monitor progress and adjust treatment.

Warning Signs to Watch For

Although early kidney damage often has no symptoms, be alert to these "red flags":

Urinary Changes: Persistent small bubbles in the urine or a noticeable increase in nighttime urination.

Swelling: Puffiness in the ankles, legs, or around the eyes that leaves a dent when pressed.

Uncontrollable Blood Pressure: Sudden difficulty in keeping blood pressure within a normal range.

Regular screening is the most effective way to catch early signs of kidney trouble. By maintaining good control of blood sugar and blood pressure, and sticking to a regular check up schedule, you can significantly protect your kidney health and slow down any potential damage.

**Q12. What blood and urine tests are used to check for diabetic kidney disease?**

model: ChatGPT

**answer:**

The two main tests used to check for diabetic kidney disease are:

Test type Test What it checks

Blood test Serum creatinine with estimated glomerular filtration rate eGFR How well the kidneys filter blood. eGFR is calculated from creatinine and patient factors; it is more useful than creatinine alone.

Urine test Urine albumin to creatinine ratio UACR Whether small amounts of albumin/protein are leaking into the urine, an early sign of kidney damage.

Guidelines emphasize using both eGFR and urine albumin/UACR, because one can be abnormal while the other is still normal. NIDDK describes these as the two key CKD markers, and ADA 2026 recommends monitoring both albuminuria and eGFR at least annually in people with diabetes.

Urine tests commonly used:

Spot urine UACR preferred screening test. It compares urine albumin with urine creatinine, so a 24 hour urine collection is usually not needed. A UACR greater than 30 mg/g suggests albuminuria and should usually be repeated to confirm.

Urinalysis / dipstick may check protein, blood, glucose, ketones, and urine sediment. It is less sensitive than UACR for early diabetic kidney disease but helps look for other kidney or urinary problems.

Urine protein to creatinine ratio or 24 hour urine protein sometimes used when heavier protein loss or non diabetic kidney disease is suspected, but not the usual first line screening test.

Blood tests commonly used:

Serum creatinine with eGFR the main blood test for kidney function. An eGFR below 60 mL/min/1.73 m2 for more than 3 months may indicate CKD.

Cystatin C based eGFR sometimes used when creatinine based eGFR may be inaccurate or when a more precise estimate is needed; KDIGO 2024 notes that creatinine based eGFR is the first approach, with creatinine plus cystatin C eGFR promoted when more precision is required.

BUN, electrolytes, bicarbonate, potassium, calcium, phosphorus, albumin, CBC supportive tests to assess kidney related complications or alternative causes, not the primary screening tests. NIDDK lists these as part of initial CKD evaluation when clinically appropriate.

In practice, a typical diabetic kidney screening panel is: blood creatinine/eGFR plus spot urine UACR, usually checked once a year or more often if abnormal.

model: Gemini

**answer:**

Diabetic kidney disease (also known as diabetic nephropathy) rarely causes noticeable symptoms in its early stages.

Because of this, doctors rely on two routine lab tests to catch kidney damage early, when it is easiest to slow or stop.

Diagnosing and monitoring the disease requires a combination of one urine test and one blood test:

Test Type What it measures What it tells your doctor

UACR Urine Albumin (protein) leakage If the kidneys' physical filters are becoming "leaky" and damaged.

eGFR Blood Serum creatinine buildup How efficiently your kidneys are filtering waste from your blood.

Here is a breakdown of how each test works and what the numbers mean.The Urine Test: UACRThe Urine Albumin to Creatinine Ratio (UACR) is usually a simple "spot" urine test, meaning you just provide a single sample in a cup at the doctor's office. What it checks: It compares the amount of albumin (a protein normally kept in your blood) to creatinine (a normal waste product) in your urine. Why it matters: Healthy kidneys act like a coffee filter, keeping essential proteins in the bloodstream while letting waste pass into the urine. When high blood sugar damages these delicate filters, they become leaky, allowing albumin to slip through. Protein in the urine is often the very first sign of diabetic kidney disease. The numbers: A normal UACR is less than 30 mg/g. A result of 30 mg/g or higher indicates kidney stress. Because urine protein can temporarily spike from things like heavy exercise or a minor infection, doctors usually look for an elevated result in two out of three tests over a 3 to 6 month period to confirm a diagnosis. The Blood Test: eGFRThe estimated Glomerular Filtration Rate (eGFR) is derived from a standard blood draw. What it checks: The lab measures your blood for serum creatinine, a waste product generated naturally by your muscles. The lab then uses a formula factoring in your age, sex, and the creatinine level to calculate your eGFR. Why it matters: If your kidneys aren't filtering well, creatinine backs up and builds up in your blood. The eGFR gives your doctor a highly accurate estimate of exactly how many milliliters of blood your kidneys are cleaning per minute.The numbers: An eGFR of 60 or higher is generally considered normal. An eGFR below 60 that persists for three months or more indicates chronic kidney disease. As kidney function declines, the eGFR number drops. When Should You Be Tested?The American Diabetes Association (ADA) recommends routine screening because catching kidney stress early opens the door to highly effective medications and lifestyle adjustments that protect the kidneys. Type 2 Diabetes: You should be tested immediately upon diagnosis, and once a year after that. Because Type 2 diabetes can go unnoticed for years, kidney damage may already be present when diabetes is first found.

Type 1 Diabetes: You should be tested 5 years after your diagnosis, and once a year after that.

model: DeepSeek

**answer:**

To check for diabetic kidney disease (diabetic nephropathy), doctors typically use a combination of blood and urine tests that focus on kidney function and damage.

Urine Tests

Albumin to Creatinine Ratio (UACR) This is the primary urine test. It measures microalbumin (tiny amounts of protein) relative to creatinine. A result of 30 to 300 mg/g indicates microalbuminuria (early kidney disease), and greater than 300 mg/g indicates macroalbuminuria (more advanced disease).

Urine dipstick A less sensitive test for proteinuria, sometimes used for screening but not as reliable as UACR.

Blood Tests

Serum creatinine Measures waste product from muscle breakdown. Elevated levels suggest reduced kidney function.

Estimated Glomerular Filtration Rate (eGFR) Calculated from serum creatinine (and often age, sex, race). An eGFR less than 60 mL/min/1.73m2 for greater than or equal to 3 months indicates chronic kidney disease (CKD). Lower values (e.g, less than 30 or less than 15) indicate more severe stages.

Blood urea nitrogen (BUN) Less specific, but elevated BUN with normal creatinine may suggest dehydration; both elevated together often indicates kidney impairment.

Guidelines recommend:

Annual UACR and eGFR testing for all people with type 2 diabetes, and for those with type 1 diabetes 5 years after diagnosis.

Frequency: Repeat every 6 to 12 months if abnormalities are detected.

These two markers (UACR and eGFR) are the cornerstones for diagnosing, staging, and monitoring diabetic kidney disease.

model: Doubao

**answer:**

Diabetic kidney disease (diabetic nephropathy) screening and monitoring rely on urine tests (to detect kidney leakage of protein) and blood tests (to measure kidney filtration function).

1. Key Urine Tests (core screening for early DKD)

(1) Urine Albumin to Creatinine Ratio (UACR) Gold standard early marker

Purpose: Detects tiny amounts of albumin (a blood protein) leaking into urine, the earliest sign of DKD.

Specimen: Random spot urine (no 24 hour collection needed).

Cutoffs (mg/g creatinine):

Normal: less than 30

Microalbuminuria (early DKD): 30 to 299

Macroalbuminuria (overt kidney damage): greater than or equal to 300

Requires repeat testing 2 to 3 times over 3 to 6 months to confirm (transient elevation can happen from infection, exercise, high blood sugar).

(2) 24 hour Urine Albumin Excretion

Traditional confirmatory test for high UACR.

Measures total albumin lost in a full day's urine; reference normal: less than 30 mg/24h.

Less convenient than UACR, used for formal diagnosis or research.

(3) Routine Urinalysis

Checks for overt proteinuria, hematuria, infection, glycosuria.

Dipstick protein only picks up heavy albumin loss (misses mild microalbuminuria), so cannot replace UACR for early screening.

2. Key Blood Tests (assess glomerular filtration rate, kidney damage)

(1) Serum Creatinine

Waste product cleared by healthy kidneys. Rising levels mean reduced filtration.

Affected by muscle mass, race, age; not used alone.

(2) Estimated Glomerular Filtration Rate (eGFR)

Calculated automatically from serum creatinine, age, sex, ethnicity.

Measures how well kidneys filter blood:

greater than or equal to 90: Normal kidney function

60 to 89: Mild reduction

30 to 59: Moderate CKD

15 to 29: Severe CKD

less than 15: Kidney failure

DKD staging combines eGFR plus UACR.

(3) Blood Urea Nitrogen (BUN)

Urea waste filtered by kidneys. Elevates with impaired clearance, dehydration, high protein diet. Used alongside creatinine.

(4) Serum Electrolytes (renal panel)

Potassium: Kidneys excrete excess potassium; advanced DKD causes hyperkalemia (life threatening).

Phosphorus: Poor clearance leads to high phosphate, bone and vascular damage.

Bicarbonate: Low levels signal metabolic acidosis in late DKD.

(5) Additional blood tests for DKD complications/risk

Lipid panel (cholesterol, triglycerides): Accelerates kidney and cardiovascular damage.

Glycated hemoglobin (HbA1c): Tracks long term blood glucose control, main driver of DKD progression.

Serum albumin: Low in advanced DKD due to massive urinary protein loss.

3. Standard Screening Protocol for Diabetes

Annual UACR plus eGFR starting at diagnosis for type 2 diabetes; 5 years after diagnosis for type 1 diabetes.

If abnormal UACR/eGFR: repeat testing, plus full renal blood panel, lipid profile, HbA1c.

Summary Table

Test Type Tests Main Role

Urine screening UACR (first line), 24h urine albumin Detect early protein leakage (microalbuminuria)

Kidney function blood Creatinine, eGFR, BUN Measure glomerular filtration capacity

Metabolic and complication blood HbA1c, lipids, electrolytes, serum albumin Control diabetes and monitor DKD complications

model: Kimi

**answer:**

The main tests used to screen for and monitor diabetic kidney disease (DKD) are:

Urine Tests

Urine Albumin to Creatinine Ratio (UACR)

The primary screening test. It detects microalbuminuria (small amounts of albumin in urine) often the first sign of DKD.

A UACR greater than or equal to 30 mg/g on at least two of three tests over 3 to 6 months indicates persistent albuminuria.

A spot urine sample is usually sufficient; a 24 hour collection is rarely needed.

Urinalysis

A dipstick test may show protein, blood, or other abnormalities. However, standard dipsticks often miss small amounts of albumin, so UACR is preferred for early detection.

Blood Tests

Serum Creatinine and Estimated Glomerular Filtration Rate (eGFR)

Creatinine is a waste product filtered by the kidneys. Rising levels indicate reduced kidney function.

eGFR is calculated from creatinine (and sometimes cystatin C), age, sex, and race. It estimates how well kidneys filter blood.

eGFR staging:

G1: greater than or equal to 90 (normal or high)

G2: 60 to 89 (mildly decreased)

G3a: 45 to 59 (mildly to moderately decreased)

G3b: 30 to 44 (moderately to severely decreased)

G4: 15 to 29 (severely decreased)

G5: less than 15 (kidney failure)

Blood Urea Nitrogen (BUN)

Another waste product that rises when kidney function declines, though less specific than creatinine/eGFR.

Cystatin C

Sometimes used alongside or instead of creatinine to calculate eGFR, especially when creatinine based estimates are unreliable.

Supporting Tests

HbA1c

Measures average blood glucose over 2 to 3 months. Poor control accelerates kidney damage, so it is monitored closely in DKD management.

Lipid Panel

People with DKD often have dyslipidemia, and cardiovascular risk is high.

How They Work Together

DKD is typically diagnosed and staged using both albuminuria (from UACR) and eGFR. For example:

Persistent UACR greater than or equal to 30 mg/g plus eGFR less than 60 mL/min/1.73 m2 for greater than 3 months suggests DKD.

Diabetes is the leading cause of chronic kidney disease, but doctors may also rule out other causes (e.g, glomerulonephritis, drug toxicity) before confirming DKD.

Note: Current guidelines (e.g, KDIGO, ADA) recommend annual screening for albuminuria and eGFR in all people with type 1 diabetes ( greater than or equal to 5 years duration) and all people with type 2 diabetes, starting at diagnosis.

This information is for educational purposes and does not replace medical advice. If you have concerns about kidney health, consult a healthcare provider.

model: Qwen

**answer:**

Screening for diabetic kidney disease primarily relies on the urine albumin to creatinine ratio (UACR) and eGFR. UACR detects early kidney leakage, while eGFR evaluates the kidney's overall filtration capacity.

Urine Tests: Detecting Early Leakage

Urinary Albumin to Creatinine Ratio (UACR): This is the most critical early indicator. It measures tiny amounts of albumin (a type of protein) in the urine. A normal value is typically less than 30 mg/g. Values between 30 to 300 mg/g indicate microalbuminuria, while values over 300 mg/g suggest significant kidney damage.

Urinalysis: This checks for red blood cells or white blood cells to rule out infections or other issues. However, standard urinalysis is not sensitive enough to detect the tiny amounts of protein seen in early diabetic kidney disease.

24 Hour Urine Protein Quantification: If UACR is abnormal or the condition is more advanced, doctors may ask you to collect all urine produced in a 24 hour period to measure the total protein excreted.

Blood Tests: Assessing Filtration Capacity

Serum Creatinine and eGFR: Creatinine is a waste product from muscle metabolism. It often doesn't rise significantly until half of the kidney's function is lost. Therefore, it is combined with age, sex, and weight to calculate the estimated Glomerular Filtration Rate (eGFR). An eGFR below 60 mL/min indicates reduced kidney function.

Glucose and HbA1c: Long term high blood sugar is the root cause of kidney damage. Regular monitoring of fasting glucose, postprandial glucose, and glycated hemoglobin (HbA1c) helps assess overall glycemic control to slow down kidney damage.

Testing Frequency and Precautions

Frequency: People with Type 1 diabetes should be screened annually starting 5 years after diagnosis. People with Type 2 diabetes should be screened annually starting from the day of diagnosis.

Avoiding False Positives: Strenuous exercise, fever, urinary tract infections, or menstruation can temporarily raise urine indicators. If a random urine test is abnormal, doctors usually recommend a follow up test using a morning urine sample.

Early detection and prompt intervention can stop the progression of kidney damage. If your test results come back abnormal, please consult an endocrinologist or nephrologist to create a plan for blood sugar control and kidney protection.

**Q13. Should I get both an eGFR blood test and a urine albumin test?**

model: ChatGPT

**answer:**

Yes in many cases, getting both is the right approach, because they check different parts of kidney health.

An eGFR blood test estimates how well your kidneys filter blood, while a urine albumin to creatinine ratio test often called UACR/uACR checks whether protein is leaking into the urine, which can be an early sign of kidney damage even when eGFR is still normal. The National Kidney Foundation notes that CKD can be indicated by eGFR below 60 for 3 months or more, or by kidney damage such as high urine albumin even with eGFR above 60.

You should especially ask for both tests at least yearly if you have diabetes, high blood pressure, known kidney disease, cardiovascular disease, a family history of kidney disease, or prior acute kidney injury. CDC guidance focuses CKD screening on high risk patients, including those with diabetes, hypertension, cardiovascular disease, family history of kidney disease, or prior AKI. For people with diabetes, ADA Standards of Care state that both albuminuria and eGFR should be monitored annually.

A practical way to ask your clinician is:

"Can we check both my serum creatinine/eGFR and a spot urine albumin to creatinine ratio to screen for kidney disease?"

One important detail: a UACR of 30 mg/g or higher may suggest kidney disease, but temporary factors like exercise, infection, fever, menstruation, or very high blood pressure can affect results, so abnormal urine albumin usually needs confirmation. CDC also lists urine albumin results of 30 or above as potentially indicating kidney disease.

model: Gemini

**answer:**

Yes, getting both tests is highly recommended if you are evaluating your kidney health. While they both check your kidneys, they look for completely different things. Think of your kidneys like a high tech water filtration system: one test checks if the pump is running at full speed, while the other checks if the filters have holes in them. Here is why doctors look at both numbers to get a complete picture:1. The eGFR Blood Test (Checking the Pump Speed)What it is: Estimated Glomerular Filtration Rate. What it measures: How efficiently your kidneys are filtering waste out of your blood. The limitation: Your eGFR can often look completely normal (above 60) even if early kidney damage is already happening.2. The Urine Albumin Test (Checking for Filter Leaks)What it is: Usually ordered as a uACR (Urine Albumin to Creatinine Ratio). What it measures: Whether a protein called albumin is leaking into your urine. Why it matters: Healthy kidneys keep protein in the blood. If protein is leaking into your urine, it is often the very first warning sign of kidney stress or damage frequently showing up years before your eGFR blood test drops. The Takeaway: If you only get the eGFR blood test, you might miss early stage kidney changes. Getting both tests together allows your doctor to catch potential issues early, which is when they are the easiest to manage, protect, and reverse. This dual screening is especially critical if you have risk factors like high blood pressure, diabetes, or a family history of kidney issues.

model: DeepSeek

**answer:**

Yes getting both an eGFR blood test and a urine albumin test is often recommended, especially if you have risk factors for kidney disease (e.g, diabetes, high blood pressure, family history of kidney failure, or are over 60).

Here's why both matter:

eGFR (estimated Glomerular Filtration Rate) Measures how well your kidneys filter waste from your blood. It detects decreased kidney function but doesn't show early damage from diseases like diabetes or hypertension.

Urine albumin (e.g, urine ACR Albumin to Creatinine Ratio) Detects small amounts of protein (albumin) leaking into urine. This is an early sign of kidney damage, even when eGFR is still normal.

Together, they give a complete picture: kidney function (eGFR) plus kidney damage (urine albumin). Relying on just one can miss early, treatable kidney disease.

When you might skip one:

If you're young, healthy, and have no risk factors, a routine eGFR alone is often sufficient.

But if your doctor suspects early diabetic or hypertensive kidney disease, urine albumin is critical.

Practical takeaway:

Ask your doctor. For most people with risk factors, both tests together are the standard of care for early detection and prevention of chronic kidney disease.

model: Doubao

**answer:**

Yes, for complete kidney screening, you almost always need both tests they measure different kidney damage, and neither alone tells the full picture.

1. What each test checks (different roles)

eGFR blood test (estimated glomerular filtration rate)

From a creatinine blood draw.

Measures how well your kidneys filter waste overall.

Tells you kidney function level: normal, mild/moderate/severe loss of filtration ability.

Limitation: It cannot detect early, mild kidney damage when filtration is still normal. Many people with early kidney disease have a normal eGFR.

Urine albumin test (urine albumin to creatinine ratio, UACR)

Checks for albumin (a key protein) leaking into urine.

Detects kidney structural damage / tiny filter leaks (the earliest sign of diabetic kidney disease, hypertensive kidney injury).

Limitation: Normal urine albumin does not rule out reduced filtration later; advanced kidney failure can raise albumin and lower eGFR.

2. Who absolutely needs both tests together

These groups require dual testing for standard kidney screening guidelines (ADA, KDIGO, NHS):

People with type 1 / type 2 diabetes (annual combined screening mandatory)

People with long term high blood pressure

Family history of chronic kidney disease (CKD)

Obesity, metabolic syndrome, high cholesterol

History of heart disease, stroke

Taking long term NSAIDs (ibuprofen, naproxen), certain blood pressure meds, or nephrotoxic drugs

Pregnant people (routine prenatal kidney workup)

Anyone over 60 for routine organ screening

3. Why one test alone is insufficient

Scenario 1: Normal eGFR, high urine albumin

Early kidney damage (filters leak protein but still work well). Only caught by urine test. If you only did blood work, you'd miss this warning sign.

Scenario 2: Low eGFR, normal urine albumin

Slow loss of filtration function without major protein leakage (common in aging or vascular kidney disease). Only caught by blood eGFR.

Scenario 3: Abnormal both

Confirms established chronic kidney disease, guides staging and treatment.

4. When might a doctor skip one?

Rare exceptions only:

Very low risk young person with no diabetes/hypertension/other risk factors getting a basic wellness check (many providers still order both anyway for full screening).

Isolated follow up for a known single abnormality (e.g, repeat urine albumin only to monitor diabetes after prior normal eGFR).

Final recommendation

For baseline kidney screening, monitoring diabetes or high blood pressure, or any kidney related concern:

Get both eGFR blood creatinine test AND urine albumin (UACR) test at the same time.

Combined results let your provider accurately stage chronic kidney disease and catch early reversible kidney injury.

model: Kimi

**answer:**

These two tests measure different aspects of kidney health, and doctors often order them together for a more complete picture. Here is what each one does, and why they are complementary:

eGFR (blood test)

Estimates how well your kidneys filter waste from your blood.

It is a measure of function. A low number suggests reduced kidney function.

Urine albumin to creatinine ratio (uACR)

Detects small amounts of the protein albumin leaking into your urine.

It is a measure of damage. Albumin in the urine often appears before eGFR drops, making it an early warning sign.

Why they are often paired

eGFR can be normal even when early kidney damage is present.

uACR can catch that early damage.

Together, they help stage chronic kidney disease (CKD) and guide treatment.

General situations where both are commonly recommended

Diabetes (type 1 or 2)

High blood pressure

Cardiovascular disease

Family history of kidney failure

Age over 60

Obesity or metabolic syndrome

Certain medications that affect kidneys

The bottom line

I cannot tell you whether you personally need both tests right now. That depends on your symptoms, medical history, risk factors, and what your clinician is monitoring. If you have risk factors for kidney disease, asking your doctor about both is a reasonable discussion. If you already have a diagnosis that affects kidney health, your care plan may already include regular monitoring of both.

If you are unsure, the safest next step is to discuss your specific risk factors with your healthcare provider, who can advise whether one, both, or neither is appropriate at this time.

model: Qwen

**answer:**

Yes, it is highly recommended to get both tests. Combining eGFR and urine albumin testing provides a more complete picture of your kidney health than doing either one alone.

Why both tests are important

Think of these tests as checking the kidney's "function" and "structure."

eGFR (estimated Glomerular Filtration Rate): This is calculated from a blood creatinine test. It measures how efficiently your kidneys are filtering waste from your blood.

Urine Albumin Test: This checks if protein (albumin) is leaking into your urine. Early kidney damage often causes protein to leak before the overall filtration rate (eGFR) drops. Detecting this early is crucial for timely intervention.

Who should definitely get both tests

People with Diabetes: Both type 1 and type 2 diabetes can damage the kidneys over time.

People with Hypertension: High blood pressure is a leading cause of kidney disease.

Others at Risk: This includes people with a family history of kidney disease, obesity, or those over 60 years old.

In summary, these two tests work together to catch kidney issues early. If you have risk factors like diabetes or high blood pressure, ask your doctor to include both in your annual physical exam. If results are abnormal, a nephrologist can provide a clear treatment plan.

**Q14. If my eGFR is normal, do I still need a urine albumin test?**

model: ChatGPT

**answer:**

Yes—especially if you have diabetes, high blood pressure, cardiovascular disease, or a family history of kidney failure.

eGFR and urine albumin measure different things:

eGFR: how well your kidneys filter blood.

Urine albumin-to-creatinine ratio (UACR): whether the kidney filters are leaking protein—an early sign of damage that can occur while eGFR remains normal.

Current guidelines recommend assessing at-risk people with both tests. For diabetes, both are generally checked at least annually. A spot UACR of ≤30 mg/g is usually normal; an elevated result should be repeated because exercise, infection, fever, menstruation, or marked blood-glucose elevation can temporarily raise it. KDIGO 2024 guideline, ADA Standards of Care 2026, NIDDK

If you have no kidney-risk factors, routine screening may not be necessary; discuss it with your clinician based on your history.

model: Gemini

**answer:**

Yes, you still need a urine albumin test. While both look at your kidneys, an eGFR (estimated glomerular filtration rate) and a urine albumin test check entirely different things. A normal eGFR alone does not mean your kidneys are completely healthy.Here is why doctors look at both tests to get the full picture:1. They Measure Different Types of DamageTo understand the difference, it helps to look at how your kidneys filter waste:eGFR (The Filter Speed): Calculated from a blood test, your eGFR measures how fast and efficiently your kidneys are filtering waste out of your blood. A normal eGFR (typically 90 or higher) means your kidneys are filtering at a healthy speed.Urine Albumin (The Filter Quality): This urine test looks for albumin, a type of protein that should stay in your blood. If your kidney filters are physically damaged or "leaky," albumin slips through into your urine.Think of your kidney as a coffee filter. Your eGFR tells you how fast the water is flowing through the filter. The urine albumin test tells you if there are rips or holes in the paper letting the coffee grounds spill into the cup.2. Albuminuria is an "Early Warning" SignIn conditions like diabetes or high blood pressure, physical damage to the kidney filters usually happens long before the kidneys actually start slowing down.A urine albumin test can detect tiny amounts of protein leaking into the urine (a condition called albuminuria or a positive uACR test) while your eGFR is still perfectly normal. Catching kidney stress at this early stage allows your doctor to prescribe specific medications (like ACE inhibitors, ARBs, or SGLT2 inhibitors) that can protect your kidneys and potentially reverse the damage before your eGFR ever drops.What the Guidelines RecommendMajor medical organizations, including the American Diabetes Association (ADA) and Kidney Disease: Improving Global Outcomes (KDIGO), recommend that anyone at risk for chronic kidney disease especially individuals with diabetes or high blood pressure receive both an eGFR blood test and a urine albumin test at least once a year.

Test What it measures What normal looks like

eGFR (Blood) Efficiency/Filtering Speed greater than or equal to 60 (Ideally greater than or equal to 90)

Urine Albumin to Creatinine Ratio (uACR) (Urine) Structural Damage/Filter Leakage less than 30 mg/g

If you have a condition like diabetes or hypertension, your annual kidney checkup isn't complete without both parts of the puzzle.

model: DeepSeek

**answer:**

Yes, you likely still need a urine albumin test even if your eGFR is normal. These two tests measure different things and together provide a much more complete picture of your kidney health.

Here’s why the urine albumin test is still important:

eGFR and urine albumin are complementary markers. The eGFR (estimated Glomerular Filtration Rate) is a blood test that estimates how well your kidneys are filtering waste. A urine albumin test (specifically the Urine Albumin-to-Creatinine Ratio, or UACR) checks for leakage of a protein called albumin into your urine, which is one of the earliest signs of kidney damage. You can have kidney damage that causes albumin leakage even when your overall filtering function (eGFR) is still normal.

A normal eGFR with albumin in the urine changes your risk category. Kidney disease is classified using both eGFR and UACR together. An elevated UACR (≥30 mg/g) even with a normal eGFR (≥60) puts you in a higher risk category for kidney disease progression and other health problems. This classification can guide how often you need monitoring and what treatments might be helpful.

When is a UACR test most critical?

The need for a UACR test is especially important if you have any of these risk factors:

Diabetes: Annual testing is strongly recommended for everyone with type 2 diabetes and those with type 1 diabetes for five years or more.

High blood pressure (Hypertension): This is a leading cause of kidney damage, and screening is recommended.

Heart disease: Albuminuria is a marker for cardiovascular risk as well.

Family history of kidney disease or other risk factors like obesity, older age, or smoking.

In short: A normal eGFR is good news, but it doesn't rule out early kidney damage. The UACR test is a simple, inexpensive way to catch problems early. For people with diabetes or high blood pressure, it's considered a necessary part of routine care.

If you're unsure whether this test is right for you, it's best to discuss your personal risk factors with your healthcare provider. They can give you a recommendation tailored to your specific health history.

model: Doubao

**answer:**

Yes, many people with fully normal eGFR still need urine albumin (uACR/urine albumin creatinine ratio) testing. The two markers reflect different types of kidney damage and do not replace each other.

1. What eGFR and urine albumin measure separately

eGFR

Estimated glomerular filtration rate reflects kidney filtering capacity—how well the kidneys clear waste from blood.

Normal range: ≥90 mL/min/1.73m².

It only detects advanced kidney loss. Early kidney damage often leaves filtration function intact.

Urine albumin (uACR)

Detects glomerular leakage of protein, a sign of tiny damage to kidney filter membranes.

Normal: <30 mg/g

Mild albuminuria: 30–300 mg/g

Macroalbuminuria: >300 mg/g

Albumin leakage is the earliest marker of diabetic kidney disease, hypertensive kidney injury, even when eGFR stays normal for years.

2. Key groups who must test urine albumin despite normal eGFR

Type 1 / Type 2 diabetes

Diabetic nephropathy first presents with albuminuria; eGFR only declines in late stages. Annual uACR screening is standard guideline care regardless of eGFR.

Chronic hypertension

Long-standing high blood pressure damages glomeruli before filtration drops. Albuminuria predicts higher cardiovascular and kidney failure risk.

Cardiovascular disease, heart failure

Microalbuminuria signals generalized small-vessel damage, even with preserved eGFR.

Family history of chronic kidney disease (CKD)

Hereditary kidney disorders may cause protein leakage early without low eGFR.

Obesity, metabolic syndrome, dyslipidemia

These metabolic stressors trigger mild glomerular albumin loss before eGFR impairment.

Autoimmune disease (lupus, vasculitis)

Kidney inflammation leaks albumin long before filtration declines.

3. Clinical guideline rationale

CKD is defined by either of the following persisting for ≥3 months:

eGFR <60 mL/min/1.73m², OR

Urine albumin ≥30 mg/g (albuminuria)

A patient with eGFR = 95 but uACR = 45 mg/g already has stage A1 CKD and requires intervention to slow progression. If you only check eGFR, you will miss this early, reversible kidney injury.

4. Exception: Low-risk individuals

If you have none of the above risk factors (no diabetes, normal blood pressure, no heart disease, no family kidney history, healthy weight), routine urine albumin screening may not be required with normal eGFR. But your doctor may still order it as part of full renal workup for baseline reference.

Summary

Normal eGFR rules out severe loss of kidney filtering function, but it cannot rule out early glomerular damage detected by urine albumin. For all patients with diabetes, hypertension, or other kidney/cardiovascular risks, urine albumin testing remains essential even with a normal eGFR.

model: Kimi

**answer:**

Yes — even with a normal eGFR, a urine albumin test is still important. Here's why:

They measure different things

eGFR estimates how well your kidneys filter waste. It reflects function.

Urine albumin detects whether protein is leaking into your urine. It reflects damage to the kidney filters.

Albuminuria can appear before eGFR drops

In early kidney disease — especially from diabetes or hypertension — the kidneys may start leaking small amounts of albumin (microalbuminuria) while filtration still appears normal. Catching this early allows for intervention before function declines.

Clinical guidelines use both

Major guidelines (such as KDIGO) classify chronic kidney disease based on both eGFR and albuminuria levels. A normal eGFR with elevated urine albumin still warrants attention and monitoring.

Who especially benefits from both tests

People with diabetes

People with high blood pressure

Those with cardiovascular disease

Individuals with a family history of kidney disease

Older adults

Bottom line: A normal eGFR is reassuring, but it doesn't rule out early kidney damage. The urine albumin test provides complementary information. If your clinician recommended it, it's worth doing — and you should discuss your specific risk factors and results with them for personalized guidance.

model: Qwen

**answer:**

Yes, you still need the test. A normal eGFR only confirms your kidney filtration function is currently intact, but it cannot rule out early-stage kidney damage.

Different Roles of the Tests

As previously discussed, eGFR and urine albumin measure different aspects of kidney health. eGFR reflects the filtering power of the kidneys, while urine albumin detects leakage. These two indicators can exist independently.

Why the Test is Still Necessary

Early Detection: A normal eGFR does not rule out early-stage kidney damage. In fact, the first sign of kidney injury is often increased albumin leakage while eGFR remains normal.

High-Risk Screening: Given your history of diabetes and existing kidney issues, you are at high risk for Diabetic Kidney Disease (DKD). Clinical guidelines recommend that diabetic patients undergo at least annual screening with both urine albumin-to-creatinine ratio (UACR) and eGFR.

Treatment Guidance: Detecting albumin in urine allows for earlier intervention. This helps slow progression and significantly reduces the risk of future cardiovascular and renal complications.

In summary, a normal eGFR is a positive sign, but it does not replace the need for urine albumin testing. Continuing regular monitoring of both markers is the most effective way to protect your kidney health.

**Q15. Can one abnormal urine albumin test diagnose diabetic kidney disease?**

model: ChatGPT

**answer:**

No. One abnormal urine albumin result does not usually diagnose diabetic kidney disease (DKD).

Urine albumin can rise temporarily because of strenuous exercise, fever, infection, menstruation, severe hyperglycemia, uncontrolled hypertension, or heart failure. The preferred test is the urine albumin-to-creatinine ratio (UACR).

Generally:

UACR <30 mg/g: normal to mildly increased

UACR ≥30 mg/g: abnormal

At least 2 of 3 UACR samples collected over 3–6 months should be abnormal to confirm persistent albuminuria.

Kidney function should also be assessed using serum creatinine and estimated glomerular filtration rate (eGFR).

Chronic kidney disease requires kidney abnormalities—such as persistent albuminuria or eGFR below 60 mL/min/1.73 m²—to persist for at least 3 months.

Therefore, an abnormal result should prompt repeat testing and evaluation for temporary or non-diabetic causes, not an immediate DKD diagnosis.

model: Gemini

**answer:**

No, a single abnormal urine albumin test cannot diagnose diabetic kidney disease (DKD).

Because urine albumin levels can fluctuate easily due to temporary, everyday factors, medical guidelines require a pattern of consistency rather than a single snapshot.

Here is how the diagnosis actually works and why one test isn't enough.

The "2 out of 3" Rule

To officially diagnose diabetic kidney disease using urine testing, you generally need to have at least two abnormal urine albumin to creatinine ratio (UACR) tests within a 3 to 6 month period.

Doctors look for persistent microalbuminuria (small amounts of protein in the urine) or macroalbuminuria (larger amounts) over time.

Why a Single Test Can Be Misleading

Urine albumin can spike temporarily for reasons completely unrelated to permanent kidney damage. If you take a test during or shortly after any of the following, it can cause a "false positive" or a temporary high reading:

Intense exercise within the 24 hours before the test

A recent or current urinary tract infection (UTI)

A high fever or acute illness

Temporary, significant high blood pressure

Very high blood sugar levels at the time of the test

Menstruation

The Complete Diagnostic Picture

A urine test is only half of the equation. To get a clear picture of kidney health, doctors pair the UACR test with a blood test:

Urine Albumin to Creatinine Ratio (UACR): Measures how much protein is leaking into your urine. An abnormal result is typically a value of 30 mg/g or higher.

Estimated Glomerular Filtration Rate (eGFR): A blood test that measures how well your kidneys are filtering waste. A persistent score below 60 indicates reduced kidney function.

If you recently received an abnormal result, the next standard step is usually to manage blood sugar and blood pressure effectively and schedule a repeat test in a few weeks or months to see if the protein clearance returns to normal.

model: DeepSeek

**answer:**

No, a single abnormal urine albumin test cannot diagnose diabetic kidney disease (DKD).

A diagnosis requires persistent abnormalities on repeat testing, along with the exclusion of temporary interfering factors. Urine albumin levels are inherently variable and can be influenced by many transient conditions.

Diagnostic Criteria and Process

Clinically, DKD is diagnosed based on two key markers: Urine Albumin-to-Creatinine Ratio (UACR) and estimated Glomerular Filtration Rate (eGFR).

For urine albumin assessment, the standard diagnostic workflow is:

Initial screening: An abnormal UACR is detected (typically defined as ≥30 mg/g).

Confirmation: This initial result must be confirmed by repeat testing. Clinical guidelines generally recommend that at least 2 out of 3 tests performed over a period of 3 to 6 months should be abnormal to establish "persistent" albuminuria.

Excluding interfering factors: Before repeating the test, transient causes of elevated urine albumin must be ruled out, such as:

Fever

Strenuous exercise

Urinary tract infection

High-protein diet

Menstruation

Poorly controlled blood glucose or blood pressure

Importance of Comprehensive Assessment

Even if persistent albuminuria is confirmed, diagnosis does not rely solely on this single marker.

Integrated evaluation: Diagnosis requires combining UACR with eGFR. Some diabetic patients may have normal urine albumin but already show a decline in eGFR — relying only on urine protein would miss these cases. Thus, clinical staging integrates both UACR and eGFR (e.g., the KDIGO GA staging system).

Other clues: Physicians also consider the patient's duration of diabetes, presence of diabetic retinopathy, and other clinical features. If atypical findings are present (e.g., no retinopathy, rapid eGFR decline, or active urinary sediment), the possibility of non-diabetic kidney disease should be considered.

In summary: A single abnormal urine albumin test is an important warning sign that calls for a more thorough evaluation and repeat testing, but it is never sufficient for a definitive diagnosis of diabetic kidney disease.

model: Doubao

**answer:**

No, a single abnormal urine albumin result alone cannot confirm a diagnosis of diabetic kidney disease.

Detailed explanation

1. Why one abnormal value is insufficient

Urine albumin excretion is transiently elevated by many non-kidney-damage triggers unrelated to diabetic nephropathy:

Strenuous exercise

Fever, infection (urinary tract infection, systemic inflammation)

Uncontrolled high blood glucose / severe hyperglycemia

Hypertension crisis

Heart failure

Menstrual contamination, hematuria

Dehydration, heavy protein intake

These factors cause temporary albuminuria, which resolves after the stressor is removed. A single positive result may be reversible functional change, not permanent diabetic renal injury.

2. Standard diagnostic criteria for DKD albuminuria

International guidelines (ADA, KDIGO) require persistent albuminuria for diagnosis:

At least 2 out of 3 urine albumin tests (UACR, urinary albumin creatinine ratio) collected over a 3–6 month period must show elevated levels, after excluding confounding transient causes.

Normal UACR: <30 mg/g

Microalbuminuria (early DKD marker): 30–299 mg/g

Macroalbuminuria (overt DKD): ≥300 mg/g

3. Additional mandatory requirements to diagnose DKD

Even with persistent albuminuria, clinicians must verify all of the following to rule out non-diabetic kidney disease:

Confirmed long-standing diabetes (usually ≥5 years for type 1 diabetes; type 2 diabetes may present with DKD at diagnosis)

Presence of other diabetic microvascular complications (diabetic retinopathy is strongly correlated with DKD)

Absence of signs of other primary kidney diseases:

Absence of active urine sediment (red cell casts, dysmorphic red blood cells)

No rapid decline in eGFR over weeks/months

Negative autoimmune renal disease serology

No history of glomerulonephritis, polycystic kidney disease, toxic nephropathy

4. Exception: DKD without albuminuria

A subset of patients with diabetes develop reduced eGFR (declined glomerular filtration rate) with normal UACR—non-albuminuric DKD. These patients will have normal urine albumin tests but still have confirmed diabetic kidney damage. This further proves urine albumin testing alone cannot rule in or rule out DKD.

Summary

Isolated abnormal urine albumin: only a screening alert, not diagnostic.

Diagnosis needs: persistent elevated albuminuria (2/3 tests over months) + long diabetes duration + diabetic retinopathy + exclusion of other kidney disorders + consistent eGFR changes.

Normal urine albumin does not exclude DKD, as non-albuminuric DKD exists.

model: Kimi

**answer:**

No. A single abnormal urine albumin test cannot diagnose diabetic kidney disease (DKD). Here is why, and what the proper process involves.

Why one test is insufficient

Urine albumin can rise temporarily due to many non disease factors:

Exercise (especially vigorous activity within 24 to 48 hours)

Fever or acute illness

Urinary tract infection

Heart failure or uncontrolled hypertension

Dehydration

Menstrual contamination

Because of this, one elevated result only indicates possible albuminuria; it does not confirm persistent kidney damage.

The diagnostic standard

DKD is diagnosed by persistent albuminuria in a person with diabetes, after excluding other causes of kidney disease. The standard confirmation process is:

Initial abnormal result usually measured as urine albumin to creatinine ratio (ACR).

Repeat testing at least 2 out of 3 tests must be abnormal over a period of 3 to 6 months.

Exclusion of other causes rule out infections, hematuria, non diabetic kidney diseases, etc.

ACR categories (mg/g)

Category Range Interpretation

A1 less than 30 Normal to mildly increased

A2 30 to 300 Moderately increased (formerly "microalbuminuria")

A3 greater than 300 Severely increased (formerly "macroalbuminuria")

Bottom line

An abnormal albumin test is a screening red flag, not a diagnosis. If you or a patient has one abnormal result, the next step is repeat testing and clinical evaluation by a healthcare provider. If albuminuria is confirmed as persistent, then diabetic kidney disease can be diagnosed and staged (often alongside eGFR assessment).

model: Qwen

**answer:**

No, a single abnormal urine albumin test cannot diagnose diabetic kidney disease. A single abnormal result can be influenced by various temporary factors and requires confirmation through re-testing.

Why a Single Test Is Not Enough

Urine albumin excretion can be temporarily elevated by short-term factors such as recent vigorous exercise, fever, urinary tract infections, poor glycemic control, or blood pressure fluctuations. Therefore, a single high result does not necessarily indicate irreversible kidney damage.

According to clinical guidelines, diabetic kidney disease is typically diagnosed based on persistently elevated albuminuria and/or a decline in estimated glomerular filtration rate (eGFR). To make a definitive diagnosis, after excluding these temporary confounding factors, at least two out of three tests over a 3- to 6-month period must show albuminuria (UACR ≥ 30 mg/g) or a persistent decline in eGFR.

What Should Be Done Next?

1. Schedule Follow-Up Testing

It is recommended to re-test the urine albumin-to-creatinine ratio (UACR) or 24-hour urine albumin excretion rate within 3 to 6 months after avoiding potential interfering factors. If the follow-up results remain abnormal, they can serve as a critical basis for diagnosis.

2. Comprehensive Evaluation of Other Indicators

In addition to urine albumin, other factors must be evaluated, including the estimated glomerular filtration rate (eGFR), the presence of diabetic retinopathy, and blood pressure control. If diabetic retinopathy is present and other non-diabetic kidney diseases have been ruled out, the evidence for diagnosing diabetic kidney disease is much stronger.

3. Rule Out Other Kidney Diseases

Some patients with diabetes may have concurrent non-diabetic kidney diseases (such as primary glomerular diseases). If there is a sudden, dramatic increase in proteinuria, active urinary sediment (e.g., hematuria), or refractory hypertension, a physician may recommend a kidney biopsy to clarify the underlying cause.

**Dimension 4-Test interpretation**

**Q16. What does eGFR mean in my kidney blood test report?**

model: ChatGPT

**answer:**

eGFR means estimated glomerular filtration rate. It is an estimate of how well your kidneys are filtering waste from your blood, usually calculated from your blood creatinine level, age, and sex. It is reported as mL/min/1.73 m2.

A simple way to read it:

eGFR result What it often means

90 or higher Usually normal kidney filtration, unless urine tests show kidney damage

60 to 89 Mildly reduced; may be normal with aging, but urine albumin/protein matters

45 to 59 Mild to moderate reduction

30 to 44 Moderate to severe reduction

15 to 29 Severe reduction

Below 15 Kidney failure range; needs urgent specialist follow up

These ranges are commonly used for CKD staging, but one eGFR result alone does not necessarily mean chronic kidney disease. CKD is usually considered when eGFR is below 60 for at least 3 months, or when eGFR is above 60 but there are other signs of kidney damage, such as albumin/protein in the urine.

Also, eGFR is an estimate, not a perfect measurement. It can be affected by muscle mass, hydration, recent illness, pregnancy, and some medications. It normally tends to decline with age.

What to check next on your report: look for creatinine, urine albumin to creatinine ratio/uACR, urine protein, and whether previous eGFR results show the same pattern. If you share your eGFR number, age, and whether your urine protein/albumin was abnormal, I can help you interpret what the report is likely suggesting.

model: Gemini

**answer:**

eGFR stands for estimated Glomerular Filtration Rate.

In plain English, it is an estimate of how well your kidneys are filtering waste out of your blood. Instead of measuring your kidney function directly, labs calculate this number using a formula that looks at the amount of creatinine (a normal waste product from muscle breakdown) in your blood sample, along with your age and biological sex.

Think of it like a efficiency score for your kidneys' filtering system.

How to Read the Numbers

While you should always go over your specific results with your doctor, labs generally group eGFR results into a few main ranges:

eGFR Value What it Generally Means

90 or higher Normal / Optimal. Your kidneys are filtering at full or near full capacity.

60 to 89 Mild Decrease. This can be perfectly normal for older adults, or it might point to early stage kidney stress.

Below 60 Moderate to Severe Decrease. If this number stays below 60 for three months or more, it typically indicates chronic kidney disease (CKD), meaning the filters aren't working as well as they should.

Why it's "Estimated"

The "e" is crucial here. Because a true filtration test is complex and time consuming, doctors use your blood creatinine level as a proxy. However, because creatinine comes from your muscles, things like having a lot of muscle mass, being dehydrated, taking certain medications, or even eating a large amount of cooked meat before the test can temporarily throw the estimate off.

A single test is just a snapshot. Doctors rarely make conclusions based on one eGFR result alone. They look for trends over time and pair it with other tests, like a urine sample, to see if protein is leaking through the filters.

model: DeepSeek

**answer:**

eGFR stands for estimated Glomerular Filtration Rate.

In plain language, it is a calculation of how well your kidneys are filtering waste from your blood. It's the best overall measure of kidney function.

Here is the breakdown you need to read your report:

1. What it actually measures

Your kidneys have millions of tiny filters called glomeruli. The eGFR estimates how much blood (in milliliters) passes through those filters per minute. A higher number generally means better filtering.

2. The Standard Numbers (for adults)

Normal (No kidney disease): greater than or equal to 90 mL/min/1.73m2

Mildly reduced: 60 to 89 (May still be normal for some, like older adults)

Mild to moderate damage: 45 to 59

Moderate to severe damage: 30 to 44

Severe damage: 15 to 29

Kidney failure: less than 15

3. What affects the result

The lab calculates your eGFR using your blood creatinine level plus:

Age

Sex (assigned at birth)

Race (some newer labs have removed race)

(Sometimes) Body size

4. Important caveats (Don't panic yet)

One low result isn't a diagnosis: If you are dehydrated, ate a large cooked meat meal before the test, or took certain medications (like ibuprofen), your eGFR can temporarily drop.

Natural aging: It is normal for eGFR to drop slowly after age 40 to 50. A person who is 75 years old with an eGFR of 65 likely has normal kidneys for their age.

Lower in young adults: Some healthy young people naturally hover around 85 to 90.

5. When to be concerned

Call your doctor if your eGFR is:

less than 60 and has been dropping on multiple tests over months (not just one test).

less than 45 (Moderate to severe damage) your doctor will likely change your diet/medications.

less than 15 (Kidney failure you will likely need dialysis or transplant evaluation).

Bottom line for you:

Above 60 and no protein in urine? Usually fine.

Below 60 or dropping? Show the report to your doctor to look for a cause (diabetes, high BP, blockage, or medication side effects).

Disclaimer: I am an AI, not a doctor. Always discuss your specific lab results with the physician who ordered them, as they know your full medical history.

model: Doubao

**answer:**

eGFR is estimated Glomerular Filtration Rate

Breakdown

GFR: Glomerular Filtration Rate the core measurement of how well your kidneys filter waste from blood. Tiny filters (glomeruli) in kidneys clear toxins out into urine.

e: estimated doctors calculate this number using your blood creatinine level, age, gender, race (in some formulas), no direct kidney scan needed.

What the number means

Units: mL/min/1.73m2 (standard for adult body size)

eGFR value Kidney function stage Explanation

greater than or equal to 90 Stage 1 (normal/mild damage) Kidneys filter waste normally; minor damage possible

60 to 89 Stage 2 (mild decline) Slightly reduced filtration, often no symptoms

30 to 59 Stage 3 (moderate CKD) Noticeable kidney function loss; monitoring required

15 to 29 Stage 4 (severe decline) Serious kidney impairment; prepare for dialysis/transplant

less than 15 Stage 5 (kidney failure/ESRD) Kidneys barely work; dialysis or transplant necessary

Key notes

It's calculated from serum creatinine (the waste product measured on your kidney blood panel). Higher creatinine is lower eGFR.

Temporary low eGFR can be caused by dehydration, heavy exercise, certain medications, infection this is reversible, not permanent kidney disease.

Doctors pair eGFR with urine albumin/creatinine ratio to fully assess chronic kidney disease (CKD).

For people under 18, pregnancy, or extreme body size, eGFR formulas are less accurate and other tests are used.

Simple summary

Your eGFR number tells your doctor how efficiently your kidneys clean your blood. A higher number is healthier kidney filtration. A persistently low result means further kidney evaluation is needed.

model: Kimi

**answer:**

eGFR stands for estimated Glomerular Filtration Rate. It's one of the most important numbers on your kidney blood test report because it tells you how well your kidneys are filtering waste from your blood.

What it means in plain terms

Your kidneys act like a cleaning filter for your blood. eGFR is a calculated estimate of that filtering speed, expressed in mL/min/1.73m2. The lab calculates it mainly from your creatinine level, along with your age and sex.

How to read the number

eGFR Value What it generally indicates

90 or above Normal or near normal kidney function

60 to 89 Mildly decreased function (may be normal for elderly)

45 to 59 Mild to moderate decrease

30 to 44 Moderate to severe decrease

15 to 29 Severely decreased function

Below 15 Kidney failure / end stage renal disease

A few important caveats

One number isn't a diagnosis. A slightly low eGFR on a single test can be temporary (dehydration, certain medications, a recent illness). Doctors look at trends over time.

Age matters. eGFR naturally declines somewhat with age. A value of 70 in an 80 year old may not be alarming, while the same value in a 30 year old warrants investigation.

It's an estimate, not a direct measurement. Very high or low muscle mass, pregnancy, or extreme diets can skew the result.

What to do next

If your eGFR is flagged as low, your doctor will usually repeat the test and may add a urine albumin to creatinine ratio (uACR) check. Together, these two markers give the clearest picture of kidney health.

I'm an AI, not a doctor. If you're concerned about your result, the best person to interpret it in the context of your full medical history is your physician.

model: Qwen

**answer:**

eGFR stands for estimated Glomerular Filtration Rate. It is the core indicator for assessing kidney filtration function, representing the volume of blood the kidneys filter per minute.

What eGFR Measures

Think of your kidneys as a highly efficient filtration system. The eGFR measures the efficiency of this system. A higher value indicates a stronger ability to clear metabolic waste (such as creatinine) from the blood.

Understanding the Values

90 or above: This is within the normal range, indicating good kidney health.

60 to 89: This suggests mild kidney function decline. If there are no other abnormalities, regular monitoring is usually sufficient.

Below 60: This is a critical threshold. A value below 60 indicates kidney damage and usually requires further investigation to identify the underlying cause.

Why It Matters More Than Creatinine Alone

Your report likely includes "Serum Creatinine." While this measures waste products, it can be influenced by age, muscle mass, and diet. The eGFR uses a formula that factors in creatinine, age, and gender to provide a more accurate picture of your true kidney health.

In summary, an eGFR above 90 is ideal, while a value below 60 warrants a visit to a nephrologist. A specialist will combine this result with other tests, such as a urinalysis, to make a comprehensive assessment.

**Q17. What does urine albumin to creatinine ratio, or uACR, mean?**

model: ChatGPT

**answer:**

uACR stands for urine albumin to creatinine ratio. It is a urine test that compares:

Albumin a blood protein that should mostly stay in the bloodstream

Creatinine a waste product used to adjust for how concentrated or diluted the urine is

So, uACR tells you how much albumin is leaking into the urine after correcting for urine concentration. This makes it more reliable than just measuring urine protein alone. NIDDK notes that uACR is not affected by changes in urine concentration the way a simple albumin dipstick can be.

Why it matters: Albumin in urine can be an early sign of kidney damage, especially in people with diabetes, high blood pressure, cardiovascular risk, or chronic kidney disease. A uACR above 30 mg/g may suggest kidney disease even when eGFR is still normal.

Typical categories are:

uACR result Meaning

less than 30 mg/g Normal to mildly increased

30 to 300 mg/g Moderately increased albuminuria

greater than 300 mg/g Severely increased albuminuria

These categories are used in CKD classification systems such as KDIGO and the National Kidney Foundation.

A single high uACR does not automatically mean permanent kidney disease. It can rise temporarily from exercise, fever, infection, dehydration, very high blood sugar, uncontrolled blood pressure, or menstruation. Clinicians usually repeat the test, often with a first morning urine sample, and interpret it together with eGFR, blood pressure, diabetes status, medications, and overall risk.

model: Gemini

**answer:**

The urine albumin to creatinine ratio (uACR) is a simple urine test used to check for early signs of kidney damage. It is most commonly ordered for people with conditions that place stress on the kidneys, such as diabetes or high blood pressure.

To understand what it means, it helps to break down the two markers the test is measuring:

Albumin: This is a vital protein found in your blood. Healthy kidneys have microscopic filters (nephrons) that keep large, useful particles like albumin inside your bloodstream. If the kidney's filters are strained or damaged, albumin begins to leak through into your urine.

Creatinine: This is a normal chemical waste product generated by muscle breakdown. Healthy kidneys filter it out of the blood and remove it in urine at a relatively steady, consistent rate.

Why do doctors measure them as a ratio?

If a test only measured the total amount of albumin in your urine, the results would change dramatically depending on how much water you drank that day. If you are dehydrated, your urine is highly concentrated, making the albumin level look artificially high; if you drank a gallon of water, it would look artificially low.

Because your body excretes creatinine at a predictable rate, dividing the urine albumin concentration by the urine creatinine concentration eliminates this variable. The ratio gives your doctor an accurate, steady baseline regardless of your hydration level.

Understanding the Ranges

The test measures how many milligrams (mg) of albumin are present for every gram (g) of creatinine. Clinicians categorize the results into three main tiers:

uACR Result Category What it Means

Less than 30 mg/g Normal to mildly increased Your kidneys are filtering properly; no significant protein leakage.

30 to 300 mg/g Moderately increased Also known as microalbuminuria. This is an early sign of kidney stress or mild damage.

Greater than 300 mg/g Severely increased Also known as macroalbuminuria. This indicates more significant kidney damage or disease.

As shown in the clinical framework above, doctors evaluate your overall kidney health by pairing your uACR category (the columns, showing how much protein is leaking) with your GFR stage (the rows, measuring how fast your kidneys are filtering waste overall).

A quick note on variations: A single elevated uACR reading doesn't automatically mean you have chronic kidney disease. Dehydration, intense exercise right before the test, a high protein diet, urinary tract infections, or a high fever can temporarily cause protein to spike in your urine. Because of this, doctors typically like to confirm an elevated result with a follow up test.

model: DeepSeek

**answer:**

uACR (urine albumin to creatinine ratio) is a simple urine test that measures how much albumin (a type of protein) is leaking from your kidneys into your urine, while adjusting for how concentrated your urine is at that moment.

Here's what the result means in plain terms:

Normal: uACR less than 30 mg/g

to Little to no albumin leakage; healthy kidney function.

Moderately increased (formerly "microalbuminuria"): uACR 30 to 300 mg/g

to Early sign of kidney damage. This is often reversible or manageable with treatment (e.g, blood pressure control, diabetes management).

Severely increased (formerly "macroalbuminuria"): uACR greater than 300 mg/g

to More advanced kidney damage. Higher risk of kidney function decline and cardiovascular disease.

Why is creatinine included?

Creatinine is a waste product filtered out by the kidneys at a steady rate. By dividing albumin by creatinine, the test corrects for how diluted or concentrated your urine sample is so you don't need a 24 hour urine collection.

Who needs this test?

Most commonly people with:

Diabetes (type 1 or 2)

High blood pressure

Family history of kidney disease

Known chronic kidney disease

A single abnormal result should be repeated (typically twice over 3 to 6 months) to confirm. Always discuss your specific number with your doctor, as treatment targets may vary based on your overall health.

model: Doubao

**answer:**

1. Basic definition

uACR is Urine Albumin / Urine Creatinine

It is a standard urine test to quantify how much albumin leaks into urine, used to screen and monitor kidney damage, especially early stage chronic kidney disease (CKD).

Key substances involved

Albumin: The main protein in blood. Healthy kidneys almost block albumin from entering urine. When kidney filtering units (glomeruli) are damaged, albumin spills into urine (albuminuria).

Creatinine: A waste product made by muscle metabolism. It is excreted steadily by healthy kidneys, so its concentration in a single random urine sample stays consistent for an individual.

2. Why we use the ratio, not just random urine albumin

A random spot urine's albumin concentration alone is unreliable:

If you drink lots of water, urine is dilute to low albumin reading (false normal).

If dehydrated, urine is concentrated to high albumin reading (false abnormal).

Creatinine corrects for urine dilution/concentration automatically. The uACR result matches a 24 hour urine protein test (the historical gold standard) without collecting urine for a full day.

3. Clinical reference ranges (mg/g creatinine, standard unit)

Classification uACR value Meaning

Normal albumin excretion less than 30 mg/g Kidneys filter normally; no significant kidney leakage

Mild albuminuria (microalbuminuria) 30 to 299 mg/g Early kidney injury; common in uncontrolled diabetes, high blood pressure

Severe albuminuria (macroalbuminuria) greater than or equal to 300 mg/g Obvious kidney damage; advanced glomerular leakage

4. Main clinical uses

Diabetic kidney disease screening

The primary test to catch early diabetic nephropathy before kidney function declines. Guidelines recommend annual uACR for all people with type 1 and type 2 diabetes.

Hypertensive kidney damage monitoring

Persistent high blood pressure damages renal glomeruli; rising uACR signals worsening kidney harm.

Chronic kidney disease staging and progression tracking

Serial uACR tests show if kidney protein leakage is stable, improving or worsening.

Cardiovascular risk marker

Even mild albuminuria predicts higher risk of heart attack, stroke and heart failure, as it signals widespread blood vessel damage.

5. How the test works

A single random urine sample (no fasting required in most protocols) is analyzed for both albumin and creatinine. The lab divides albumin level by creatinine level to produce the uACR ratio.

6. Important notes

Transient high uACR can be caused by fever, heavy exercise, urinary tract infection, menstruation, or very high blood pressure readings. A single abnormal result usually requires repeat testing to confirm chronic kidney injury.

Treatment targets (tight blood sugar, controlled blood pressure, RAAS inhibitor medications) aim to lower uACR and slow permanent kidney loss.

model: Kimi

**answer:**

uACR stands for urine albumin to creatinine ratio. It's a simple urine test that checks how much albumin (a protein) is leaking into your urine, compared to creatinine (a waste product from muscle metabolism).

Why it matters

Healthy kidneys normally keep albumin in the blood. When kidneys are damaged especially from conditions like diabetes or high blood pressure tiny amounts of albumin can start slipping into the urine. This early leakage is called microalbuminuria, and it's often one of the first signs of kidney disease.

Why the ratio?

Urine concentration varies depending on how hydrated you are. Measuring albumin alone can be misleading. By comparing albumin to creatinine, the test normalizes for urine concentration, giving a more reliable result from a single urine sample (even a random spot urine).

How results are interpreted

uACR Result What it generally means

Less than 30 mg/g Normal

30 to 300 mg/g Moderately increased (microalbuminuria) early kidney damage

Over 300 mg/g Severely increased (macroalbuminuria) more significant kidney disease

When is it used?

Diabetes screening (Type 1 and Type 2): Recommended at least annually

High blood pressure monitoring

Tracking existing kidney disease

Assessing cardiovascular risk (elevated uACR is also linked to higher heart disease risk)

A few practical notes

A single elevated result isn't always definitive dehydration, fever, urinary tract infections, or strenuous exercise can temporarily raise albumin levels. Doctors usually repeat the test to confirm.

The test can be done with a spot urine sample (any time of day) or a 24 hour collection, though the spot test with the ratio is most common.

Lowering uACR through blood pressure control (especially with ACE inhibitors or ARBs), blood sugar management, and lifestyle changes can help protect kidney function.

If you've received a uACR result and are unsure what it means for you specifically, it's worth discussing with your healthcare provider in the context of your overall health and other lab values.

model: Qwen

**answer:**

The Urine Albumin to Creatinine Ratio (uACR) is a sensitive indicator for screening early kidney damage.

What the Test Measures

The kidneys act as a sophisticated filter, removing waste from the blood while retaining essential proteins like albumin. If the kidneys are damaged early on, albumin leaks into the urine. The uACR test measures the amount of albumin relative to creatinine (a waste product) to identify this leakage without needing a 24 hour urine collection.

Interpreting the Results

Less than 30 mg/g: Normal. This indicates healthy kidney filtration.

30 to 300 mg/g: Moderately increased (microalbuminuria). This suggests early kidney damage, which is often reversible with proper intervention.

Greater than 300 mg/g: Severely increased (severe albuminuria). This indicates significant kidney injury and requires prompt medical evaluation.

Who Should Get Tested

People with diabetes, high blood pressure, cardiovascular disease, or obesity are at higher risk for kidney damage. Regular uACR testing is strongly recommended for these high risk groups to monitor kidney health.

Factors That Affect Accuracy

Temporary spikes in uACR can occur due to fever, urinary tract infections, intense exercise, or uncontrolled blood sugar. If the initial result is elevated, doctors usually recommend repeating the test after 3 to 6 months to confirm the finding.

In summary, uACR is a convenient and effective tool for early kidney disease detection. Normal results are reassuring, while elevated results should be reviewed by a doctor to determine the underlying cause and necessary treatment.

**Q18. My uACR is above 30. Should I be worried?**

model: ChatGPT

**answer:**

A uACR above 30 mg/g is worth taking seriously, but it is not a reason to panic. It usually means there is albuminuria albumin leaking into the urine which can be an early sign of kidney stress or kidney damage. The National Kidney Foundation notes that uACR greater than or equal to 30 mg/g is above the ideal range and is linked with higher kidney and cardiovascular risk; NIDDK also describes UACR greater than 30 mg/g as a marker used to diagnose and monitor kidney disease.

The usual categories are:

uACR result Meaning

less than 30 mg/g Normal to mildly increased

30 to 300 mg/g Moderately increased albuminuria

greater than 300 mg/g Severely increased albuminuria

A single elevated result does not automatically mean chronic kidney disease. CKD generally requires kidney abnormalities to be present for at least 3 months, and uACR can rise temporarily from dehydration, intense exercise, fever/infection, urinary tract infection, heart failure flare, or sometimes menstruation.

What I'd do next: ask your clinician about repeating uACR, ideally with a first morning urine sample, and check it together with eGFR/creatinine, blood pressure, HbA1c if you have diabetes, and a urinalysis. If you have diabetes or high blood pressure, this result matters more because treatment such as optimizing blood pressure/glucose control and sometimes ACE inhibitor/ARB or kidney protective diabetes medications can reduce kidney and heart risk. ADA guidance emphasizes uACR plus eGFR screening and kidney risk management in diabetes.

Seek prompt medical advice sooner if you also have blood in urine, new swelling, very high blood pressure, shortness of breath, much less urine, or uACR greater than 300.

So: be concerned enough to follow up and repeat/confirm it, but don't assume the worst from one uACR just over 30.

model: Gemini

**answer:**

It is completely understandable to feel a bit anxious when a lab result comes back outside the normal range, but a uACR slightly above 30 is best viewed as an early warning signal rather than a reason to panic.

uACR stands for Urine Albumin to Creatinine Ratio. It measures whether a protein called albumin is leaking into your urine. In healthy kidneys, the filters are tight enough to keep important proteins in your blood while filtering out waste. If the filters are slightly stressed, small amounts of protein can slip through.

Here is how doctors generally break down the numbers:

uACR Level (mg/g) Category What it Means

Less than 30 Normal Excellent kidney filtration.

30 to 300 Moderately Increased Often called microalbuminuria. An early sign of kidney stress.

Greater than 300 Severely Increased Macroalbuminuria. Indicates more significant kidney strain.

Why a single reading isn't a diagnosis

A uACR above 30 means you are just crossing into the "moderately increased" zone, but a single test does not mean you have permanent kidney damage. Kidney protein levels can temporarily spike for completely benign, everyday reasons, including:

Recent intense exercise

High fever or a temporary illness

Dehydration

Urinary tract infections (UTIs)

What you should do next

Because of these temporary spikes, medical guidelines usually require two or three elevated tests over a period of a few months before confirming a trend.

The best next step is to share this result with your doctor. They will likely want to retest you in a few weeks to see if it was a temporary fluctuation. If it does stay consistently above 30, it is usually managed very effectively by checking on your blood pressure and blood sugar, or using common medications that protect the kidneys from further strain.

model: DeepSeek

**answer:**

It's understandable to be concerned, but rather than panic, think of a uACR above 30 mg/g as an early warning signal one that gives you a chance to protect your kidneys if you act wisely.

Here's what you need to know:

What uACR greater than 30 means:

Normal uACR is less than 30. A value of 30 to 300 is called moderately increased albuminuria (formerly "microalbuminuria"). It means your kidneys are leaking a small amount of protein, which can be an early sign of kidney damage especially from diabetes, high blood pressure, or other kidney stressors.

Should you be worried?

Worried enough to take it seriously, but not terrified. Many people with uACR 30 to 300 never progress to kidney failure if they manage the underlying cause. However, ignoring it raises the risk of kidney function decline over years.

Next steps (what to do now):

Confirm it A single high value can happen with fever, heavy exercise, UTI, or dehydration. Your doctor will likely repeat the test 2 to 3 times over 3 to 6 months.

Check your blood pressure and blood sugar These are the top causes. If you have diabetes or hypertension, control is critical.

Ask your doctor about an ACE inhibitor or ARB These blood pressure meds reduce albuminuria even if your BP is normal.

Lifestyle changes that help:

Low salt diet (helps BP)

Avoid NSAIDs (ibuprofen, naproxen) they can worsen kidney leakage

Control protein intake? Not yet needed wait for doctor's advice

Red flags that mean see a doctor sooner:

uACR greater than 300 (severely increased)

Rising creatinine or falling eGFR

Blood in urine, swelling in legs/face, foamy urine

Bottom line: You don't need to panic, but you do need to act. Make an appointment with your primary care doctor or a nephrologist. Most people with uACR 30 to 300 can stabilize or even reverse it with proper treatment. This is likely manageable not a crisis.

model: Doubao

**answer:**

1. Basic definition of uACR

uACR is Urinary Albumin to Creatinine Ratio, the gold standard to detect early kidney damage (albumin leakage into urine).

Normal: less than 30 mg/g

Mild albuminuria (early kidney injury): 30 to 300 mg/g

Severe albuminuria (obvious kidney damage): greater than 300 mg/g

A value above 30 means you have mild albuminuria, a warning sign not permanent kidney failure yet. There is no need for extreme panic, but you cannot ignore it.

2. Why uACR rises above 30 (most common causes)

Uncontrolled high blood pressure (top cause of kidney microdamage)

Diabetes (type 1/2) high blood sugar damages kidney filtering units

Obesity, metabolic syndrome, high blood lipids

Dehydration, heavy exercise, high protein meals, urinary tract infection (temporary mild elevation)

Chronic glomerular disease, autoimmune kidney issues

Long term use of NSAIDs (ibuprofen, naproxen), certain herbal supplements

3. Is it reversible? Critical takeaway

Mild albuminuria (30 to 300) is often reversible or controllable with early intervention.

If you take measures promptly, you can stop or slow kidney damage before permanent scarring occurs. This is why it's a warning marker, not a diagnosis of severe kidney disease.

4. Steps you must take immediately

Medical follow up

Repeat the test

One single elevated result can be false (due to exercise, infection, dehydration). Ask your doctor for a second morning spot urine uACR (first void urine is most accurate).

Check root risk factors

Blood pressure monitoring (target usually less than 130/80 mmHg for kidney protection)

Blood sugar / HbA1c if diabetic

Blood creatinine, eGFR (to check overall kidney filtering function)

Lipid panel, urine routine to rule out infection

Medication adjustment (prescription only)

If hypertension/diabetes is the cause, doctors often prescribe ACE inhibitors or ARBs these drugs lower urine albumin and protect kidneys. Do not start/stop them without medical advice.

Lifestyle changes (core treatment)

Limit sodium: less than 5g salt daily; avoid processed food, pickles, fast food

Moderate protein intake: Do not overload on high protein supplements/red meat

Lose weight if overweight; regular gentle cardio (walking, cycling, avoid extreme intense workouts)

Quit smoking; limit alcohol

Avoid over the counter painkillers (ibuprofen, diclofenac) unless approved by your doctor

Stay well hydrated daily

5. When to feel more concerned

Seek urgent nephrology referral if you have any of these together with uACR greater than 30:

eGFR below 60

Persistent uACR greater than 300 on repeated tests

Persistent high blood pressure / uncontrolled diabetes

Swelling of legs/face, foamy urine, fatigue, elevated blood creatinine

Summary

A uACR above 30 is an early warning signal, not a crisis. You don't need severe anxiety, but this requires consistent medical checkups and lifestyle adjustments to protect your kidneys long term. Most people can stabilize or lower their uACR with proper management.

model: Kimi

**answer:**

A uACR (urine albumin to creatinine ratio) above 30 mg/g indicates albuminuria, which is a sign of kidney damage. Here's what you should know:

What the numbers mean

uACR Range Interpretation

less than 30 mg/g Normal

30 to 300 mg/g Moderately increased (formerly "microalbuminuria")

greater than 300 mg/g Severely increased (formerly "macroalbuminuria")

Not panicked, but you should take it seriously and follow up. Albuminuria is an early marker of kidney damage, often before other symptoms appear. It's commonly associated with:

Diabetes (diabetic kidney disease)

High blood pressure

Cardiovascular disease risk albuminuria is also an independent risk factor for heart disease and stroke

What to do next

Confirm the result Repeat the test (uACR can vary with exercise, fever, UTI, or dehydration). A first morning urine sample is most reliable.

See your doctor They'll likely check:

eGFR (kidney function via blood test)

Blood pressure

Blood glucose/A1c (if diabetes status is unclear)

Monitor trends A single elevated value needs confirmation; persistent elevation ( greater than 3 out of 3 tests over 3 to 6 months) is clinically significant.

Risk reduction steps

Keep blood pressure and blood sugar well controlled

Avoid NSAIDs when possible (they stress kidneys)

Don't smoke

Maintain a healthy weight

Bottom line: uACR greater than 30 is a warning sign, not a diagnosis. With proper management, progression can often be slowed or prevented. Schedule that follow up appointment.

model: Qwen

**answer:**

Don't worry too much. A UACR above 30 is currently classified as microalbuminuria. It indicates very early kidney stress but is usually manageable.

What the Number Means

Normally, your kidneys act as filters, keeping protein in your blood. A UACR (Urine Albumin to Creatinine Ratio) above 30 mg/g means tiny amounts of albumin are leaking into your urine. It is often just a minor leak, not a broken filter.

Common Causes

Temporary Fluctuations: Fever, heavy exercise, or urinary tract infections can cause the number to go up temporarily.

Long term Conditions: The most common underlying reasons are uncontrolled diabetes or high blood pressure.

Next Steps

Recheck the Result: One test isn't enough to make a diagnosis. You should repeat the test in 3 to 6 months, preferably using your first morning urine.

See a Doctor: A nephrologist (kidney specialist) can check your blood work and urine to find the root cause.

Manage Health: Keeping your blood pressure and blood sugar under control is the best way to protect your kidneys.

Overall, this number is a gentle warning to start protecting your kidneys, not a sign of serious failure. Early intervention is highly effective.

**Q19. My eGFR dropped a little after starting a new kidney protective medicine. Is that dangerous?**

model: ChatGPT

**answer:**

Usually, a small eGFR drop soon after starting a kidney protective medicine is not automatically dangerous. For several kidney protective drugs, it can be an expected "hemodynamic dip," meaning the medicine lowers pressure inside the kidney filters. That can make eGFR look a bit lower at first, while still protecting the kidneys long term.

This is especially common with:

ACE inhibitors / ARBs such as lisinopril, losartan, valsartan. The National Kidney Foundation notes these can slightly lower eGFR because they reduce pressure in the kidney filters, and that this is not usually a sign that kidney disease is worsening. KDIGO recommends checking blood pressure, creatinine/eGFR, and potassium within 2 to 4 weeks after starting or increasing these medicines, and generally continuing unless creatinine rises by more than 30% within 4 weeks.

SGLT2 inhibitors such as empagliflozin/Jardiance, dapagliflozin/Farxiga, canagliflozin/Invokana. KDIGO states the initial reversible eGFR decrease after starting an SGLT2 inhibitor is generally not a reason to stop the medicine, and recommends SGLT2 inhibitors for many people with CKD down to eGFR 20 when appropriate.

It becomes more concerning if the drop is large, keeps falling on repeat tests, is accompanied by high potassium, very low blood pressure, dehydration, vomiting/diarrhea, use of NSAIDs like ibuprofen/naproxen, or symptoms such as severe dizziness, fainting, swelling, shortness of breath, much less urine, confusion, or chest pain. NKF also notes dehydration, NSAIDs, and diuretics can raise AKI risk when combined with ACE inhibitors/ARBs.

A practical way to frame it: a small early dip can be expected; a large or symptomatic drop needs prompt review. Do not stop the medicine on your own unless you are having emergency symptoms such as mouth/tongue/throat swelling or trouble breathing seek emergency care for those.

The most helpful details to interpret your situation are: the medicine name, your eGFR before and after, how many days/weeks after starting it the test was done, your potassium level, blood pressure, and whether you felt dehydrated or unwell.

model: Gemini

**answer:**

It is completely normal to feel a bit alarmed when you see a lab number go in the wrong direction, especially when you are taking something explicitly meant to protect that very organ. However, what you are experiencing is a very well known and expected medical phenomenon.In most cases, a small drop in eGFR (estimated Glomerular Filtration Rate) right after starting a kidney protective medication is not dangerous. In fact, it is often a sign that the medication is working exactly the way it is supposed to.Here is what is happening under the hood:The "Hemodynamic Dip" ExplainedKidney protective medications such as ACE inhibitors (like lisinopril), ARBs (like losartan), and SGLT2 inhibitors (like empagliflozin or dapagliflozin) work by changing the fluid pressure inside your kidney's filtering units (the glomeruli).Before you started the medication, the pressure inside those filters was likely too high. High pressure causes "hyperfiltration," which wears out the kidneys over time much like high water pressure straining a delicate pipe system.The Medication's Job: It opens up the vessels leading away from the filters or adjusts fluid balance to lower that intense internal pressure.The Result on Your Labs: Because the pressure drops, the kidneys filter slightly less fluid in the short term. Since eGFR measures how fast your kidneys filter, your eGFR number "dips."Think of it like taking your foot off the gas pedal to save the engine: your speed drops slightly right away, but you avoid a major breakdown down the road. Clinical studies consistently show that patients who experience this initial dip actually see their kidney function stabilize and are much better protected against long term decline over the lifespan.When does it become a concern?While a mild drop is expected, there are safety thresholds that doctors look out for:Expected Dip: A decline of 10% to 25% from your baseline value is standard and usually stabilizes within 2 to 4 weeks. Doctors typically do not stop the medication for this.Concerning Drop: A sudden drop of 30% or more can indicate that the pressure dropped too much, which can happen if you are dehydrated, taking NSAID painkillers (like ibuprofen), or have a specific narrowing of the kidney arteries.What You Should Do NextCritical Safety Note: Never stop or adjust your dose of a kidney medication without talking to your doctor first. Stopping it suddenly can cause your blood pressure or kidney pressure to spike.Check your exact numbers: Look at your lab results to see what your baseline eGFR was versus your new number to see if it falls within that normal 10 to 25% dip.Stay hydrated: Dehydration can amplify the eGFR drop when starting these medications, so make sure you are drinking enough fluids unless your doctor has given you a specific fluid restriction.Review your other pills: Ensure you aren't taking over the counter NSAID pain relievers (ibuprofen, naproxen), as these can interact with your new medication and cause eGFR to drop further.

Follow up: Your doctor will likely want to recheck your blood work (both eGFR and potassium levels) in a few weeks to confirm that the number has stabilized.

model: DeepSeek

**answer:**

This is a very common and often expected finding, not a sign of danger. In fact, a small, early drop in eGFR after starting a kidney protective medication (like an ACE inhibitor, ARB, SGLT2 inhibitor, or certain blood pressure pills) is usually a good sign that the medicine is working as intended.

Here's why it happens:

These medications lower pressure inside the kidney's filtering units (glomeruli). This reduces stress on the filters, protecting them long term. The immediate trade off is a slight, stable dip in eGFR (typically 10 to 20%, or 10 to 15 mL/min), which is reversible if you stop the drug.

When it's not dangerous:

The drop occurs within the first 2 to 4 weeks of starting or increasing the dose.

It stabilizes at a new, lower level (e.g, from 60 to 52) and doesn't keep falling.

Your potassium level stays normal, and you don't retain fluid.

When it could be a problem (and you should contact your doctor promptly):

The drop is large: eGFR falls by greater than 30% or greater than 20 mL/min.

It keeps declining week after week.

You develop signs of kidney injury: low urine output, swelling in legs/ankles, shortness of breath, or nausea.

What you should do right now:

Do not stop the medication doing so removes the long term protection.

Call your prescribing doctor and ask: "Was this drop within the expected range for this drug?" They can check your exact numbers.

Confirm timing if the drop happened within 4 weeks of starting, it's highly likely expected.

Request a repeat eGFR in 2 to 4 weeks stability is more important than the absolute number.

In short: A small, one time dip early on is expected drug effect, not damage. But always confirm with your doctor, as they know your baseline and other health conditions.

model: Doubao

**answer:**

1. Common mechanism (Not usually dangerous for most people)

The main kidney protective drugs causing mild eGFR decline are SGLT 2 inhibitors (e.g, dapagliflozin, empagliflozin) and RAAS blockers (ACEI/ARB: lisinopril, losartan, etc.), the first line medicines to slow chronic kidney disease (CKD) progression.

Why eGFR falls slightly:

These drugs dilate the kidney's efferent arteriole (the small blood vessel leaving the filtering unit). This lowers high pressure inside the glomerulus (kidney filter).

Lower glomerular pressure reduces scarring and long term kidney damage this is the therapeutic goal.

Less filtration pressure temporarily lowers the calculated eGFR value, which looks like kidney function worsened on lab tests, but the kidneys are actually being protected.

2. What counts as a normal, harmless drop

Typical mild change: 10% 30% reduction in eGFR within 1 to 4 weeks of starting or up titrating the drug.

No accompanying dangerous signs (listed below) is almost always an expected, benign response.

This slight decline usually stabilizes after 2 to 4 weeks; eGFR rarely keeps falling long term if the drug is tolerated.

3. Red flags that mean it IS dangerous (contact your doctor immediately if you have any)

Stop self judging seek urgent medical review if you have:

eGFR drops more than 30% and keeps falling week after week

Sharp rise in serum creatinine (a key marker paired with eGFR)

High potassium blood level ( greater than 5.0 mmol/L): symptoms like muscle weakness, numbness, palpitations

Signs of acute kidney injury: little/no urine output, swelling all over, fatigue, nausea/vomiting, confusion

Severe low blood pressure: dizziness when standing, fainting, cold clammy skin

Dehydration (vomiting, diarrhea, not drinking enough water) at the same time you started the drug

4. What you should do next

Do NOT stop the medicine on your own: Sudden withdrawal can spike glomerular pressure and accelerate permanent kidney damage.

Repeat kidney blood work (creatinine, eGFR, electrolytes, potassium) 1 to 2 weeks after the initial drop to check stability.

Rule out reversible triggers: dehydration, recent NSAID painkillers (ibuprofen, naproxen), infection, low blood pressure, or heart failure.

Share all lab trends with your nephrologist: They will compare your baseline eGFR vs. the new value to judge if the drop is acceptable.

Summary

A small, stable eGFR drop shortly after starting standard kidney protective medication is a normal therapeutic effect and not dangerous in isolation. Risk only exists if the drop is large, progressive, or paired with high potassium, dehydration, low urine output, or severe hypotension. Always coordinate dose adjustments with your kidney specialist.

model: Kimi

**answer:**

A small drop in eGFR after starting certain kidney protective medications can actually be expected and is not always dangerous but the details matter, and you should definitely tell your prescribing doctor.

Why it can happen:

ACE inhibitors or ARBs (like lisinopril, losartan): These dilate the blood vessels leaving the kidney, which can reduce the measured filtration rate early on, even while they are protecting your kidneys long term. A drop of up to 20 to 30% in the first 1 to 2 months is sometimes expected.

SGLT2 inhibitors (like empagliflozin, dapagliflozin): These can cause a small initial dip in eGFR (often 3 to 5 mL/min/1.73m2) that usually stabilizes or recovers somewhat over time.

NSAIDs, diuretics, or contrast dye (if any were involved): These can also affect eGFR.

When to be concerned:

A drop greater than roughly 30% from baseline

A continued downward trend over several readings

New symptoms like swelling, decreased urine output, fatigue, or nausea

If your eGFR fell below a threshold your doctor had warned you about

What you should do:

Contact your doctor they need to know, and they may want to recheck labs soon.

Ask specifically: "Is this initial drop expected with this medication, or should we adjust the dose or stop it?"

Don't stop the medication on your own unless your doctor tells you to some kidney protective drugs need to be tapered.

Important: I can't tell you whether your specific drop is safe or not. That depends on how large the drop is, what medication it is, your baseline kidney function, and other health conditions. Your doctor has that full picture.

If you want, you can share the medication name and how much your eGFR changed, and I can explain what is typically seen with that specific drug but again, that won't replace your doctor's judgment.

model: Qwen

**answer:**

Don't worry. A slight drop in eGFR after starting a kidney protective medicine is usually not dangerous; it's often a sign that the medication is working.

Why eGFR Might Drop Initially

Common kidney protective medicines, such as ACE inhibitors, ARBs, or SGLT2 inhibitors, adjust blood flow dynamics within the kidneys when you first take them. This process lowers internal kidney pressure and reduces protein leakage. This temporary dip is a sign of the drug's therapeutic effect, not kidney damage.

Differentiating Normal Response from Kidney Injury

Check the Magnitude: A drop of less than 30% is generally considered a normal reaction to the medication.

Observe the Trend: This initial decrease typically stabilizes within a few weeks of starting the medication and won't continue to drop indefinitely.

Watch for Symptoms: Actual kidney injury would typically present with a significant spike in serum creatinine, abnormal blood potassium levels, or severe symptoms like extreme fatigue and swelling.

Next Steps

Continue Medication: Do not stop taking the medication on your own. Stopping abruptly can cause your condition to rebound or worsen.

Follow up Testing: Schedule a follow up to check your kidney function again, usually 2 to 4 weeks after starting the medication, so your doctor can compare the trends.

Stay Hydrated: Ensure you drink enough water daily to prevent any temporary fluctuations in your results due to dehydration.

In summary, a slight decrease in eGFR is often a sign of the drug working. As long as the drop is small and you have no discomfort, continue taking your medication as prescribed and attend your scheduled check up.

**Q20. What do CKD stages 1 to 5 mean for someone with diabetes?**

model: ChatGPT

**answer:**

For someone with diabetes, CKD stages 1 to 5 describe how well the kidneys are filtering blood, usually measured by eGFR. Doctors should also check urine albumin to creatinine ratio, uACR, because diabetes related kidney disease may show up first as albumin/protein in the urine, even when eGFR is still normal. CKD is usually confirmed only when abnormalities last 3 months or more.

CKD stage eGFR level What it generally means in diabetes

Stage 1 greater than or equal to 90 Kidney filtering is normal or high, but there is evidence of kidney damage, often albumin in urine. This is an early warning stage.

Stage 2 60 to 89 Mild decrease in kidney function, plus evidence of kidney damage. Often no symptoms.

Stage 3a 45 to 59 Mild to moderate loss of kidney function. Medication doses, blood pressure, uACR, potassium, anemia, and heart risk need closer monitoring.

Stage 3b 30 to 44 Moderate to severe loss of kidney function. Complication risk rises; kidney specialist involvement is often considered.

Stage 4 15 to 29 Severe loss of kidney function. Planning for advanced kidney care, possible dialysis/transplant evaluation, or conservative kidney care may begin.

Stage 5 less than 15 Kidney failure. Dialysis, transplant, or conservative kidney management may be needed depending on symptoms, overall health, and patient preference.

The key point: Stage 1 to 2 are not "normal" if uACR is high, and stage 3 to 5 are based mainly on reduced eGFR. In CKD classification, both GFR stage and albuminuria category matter: A1 is uACR less than 30 mg/g, A2 is 30 to 300 mg/g, and A3 is greater than 300 mg/g; higher albuminuria means higher kidney and cardiovascular risk.

For people with diabetes, guidelines recommend regular monitoring with both eGFR and uACR, because these tests guide diagnosis, progression monitoring, medication dosing, and decisions about nephrology referral.

A reassuring note: CKD progression is not inevitable. Good glucose control, blood pressure control, avoiding kidney harming medicines when possible, and kidney protective diabetes medicines when appropriate can slow progression. Your clinician can interpret the stage together with uACR, age, blood pressure, diabetes type, medications, and other conditions.

model: Gemini

**answer:**

For someone managing diabetes, understanding Chronic Kidney Disease (CKD) stages is all about tracking how effectively your kidneys filter waste. High blood sugar can damage the tiny blood vessels (nephrons) inside your kidneys over time.

Doctors determine your stage using eGFR (estimated Glomerular Filtration Rate), which acts like a percentage score of your kidney function. They also test for albuminuria (protein leaking into your urine), which is often the earliest sign of diabetic kidney disease.

The staging system below breaks down exactly what is happening at each milestone, ranging from early, manageable changes to advanced kidney care.

Stage 1: Kidney Damage with Normal Function (eGFR 90 or higher)

What it means: Your kidneys are still filtering waste perfectly, but there are early signs of physical damage. For people with diabetes, this is almost always discovered because a routine test detected small amounts of protein (albumin) in the urine.

The Goal: Protect your kidneys early. This means tight blood sugar control and managing blood pressure, often with specific medications like ACE inhibitors or ARBs, which double as kidney protectors.

Stage 2: Mild Loss of Function (eGFR 60 to 89)

What it means: There is a slight drop in filtering efficiency, but your kidneys are still working well enough that you likely won't feel any physical symptoms.

The Goal: Keep it from moving forward. In addition to blood sugar and blood pressure management, doctors frequently look at newer diabetes medications (like SGLT2 inhibitors) that have been proven to slow down kidney decline significantly.

Stage 3: Moderate Loss of Function (eGFR 30 to 59)

Doctors often split this stage into 3a (45 to 59) and 3b (30 to 44).

What it means: Your kidneys are noticeably struggling. Waste products and extra fluid may start building up in your blood. You might begin to experience symptoms like mild swelling in your ankles or feet, fatigue, or changes in how often you urinate.

The Goal: Complication management. Your doctor will carefully monitor you for related issues like anemia or bone health changes. You may also need to adjust dosages for certain diabetes medications (like metformin), as your kidneys take longer to clear them from your system.

Stage 4: Severe Loss of Function (eGFR 15 to 29)

What it means: Your kidneys are significantly compromised. This is a crucial transitional phase where symptoms like fatigue, swelling, loss of appetite, and nausea become much more pronounced.

The Goal: Active preparation. At this point, you will work closely with a nephrologist (kidney specialist) to map out the future. This involves discussing options like a kidney transplant or setting up access for dialysis, ensuring you are prepared before reaching a medical emergency.

Stage 5: Kidney Failure (eGFR less than 15)

What it means: Your kidneys have either stopped working completely or are on the verge of it. Life threatening waste and fluid buildup will happen without medical intervention.

The Goal: Renal Replacement Therapy. To stay healthy, you will require dialysis treatments to clear your blood or a kidney transplant.

A Quick Reminder: A CKD diagnosis is a map, not a fixed destination. Many individuals with diabetes stay in Stages 1, 2, or 3 for decades without ever progressing to kidney failure, especially with early and consistent medical management.

model: DeepSeek

**answer:**

This is an excellent and important question. For someone with diabetes, understanding Chronic Kidney Disease (CKD) stages 1 through 5 is critical for preventing or delaying kidney failure and managing overall health.

In short, the stages are a way to measure how well your kidneys are filtering waste from your blood. They are primarily defined by your eGFR (estimated Glomerular Filtration Rate) and the amount of albumin (a protein) in your urine.

For a person with diabetes, these stages take on a special urgency. High blood sugar damages the tiny blood vessels in the kidneys (a condition called diabetic nephropathy), and high blood pressure (common in diabetes) accelerates this damage.

Here's what each stage means, specifically for someone with diabetes.

Quick Reference: The 5 Stages of CKD (by eGFR)

Stage 1: Kidney damage (e.g, protein in urine) but normal eGFR (90 plus ).

Stage 2: Mild loss of function, eGFR 60 to 89.

Stage 3: Moderate loss of function, eGFR 30 to 59 (often split into 3a and 3b).

Stage 4: Severe loss of function, eGFR 15 to 29.

Stage 5: Kidney failure, eGFR under 15.

(Note: Normal eGFR for a young adult is about 120 and naturally declines with age.)

Stage 1: Kidney Damage with Normal Function (eGFR 90 plus )

What it means: Your kidneys are still filtering blood perfectly. However, there are signs of early damage. The most common sign is microalbuminuria small amounts of a protein called albumin in your urine. Your blood pressure may also be creeping up.

What it means for you with diabetes: This is a critical warning and a golden opportunity. The damage is happening at a microscopic level, but your overall kidney function is still excellent.

Your goals at Stage 1:

Tight Glucose Control: Keep your HbA1c as close to your target (often less than 7%) as possible. This is the 1 way to stop further damage.

Control Blood Pressure: Target is usually less than 130/80 mmHg.

Medications: Your doctor will likely start an ACE inhibitor (like lisinopril) or an ARB (like losartan). These blood pressure meds are especially good at protecting diabetic kidneys, even if your blood pressure is normal.

Lifestyle: Healthy diet, exercise, no smoking.

Stage 2: Mild Loss of Function (eGFR 60 to 89)

What it means: You still have evidence of kidney damage (protein in urine), and your eGFR has dropped a bit, though it might still be in the "normal" range for an older person.

What it means for you with diabetes: The damage is progressing. This is the last stage where you have a very high chance of slowing or halting progression to more advanced stages. Many people live for decades in Stage 2 if managed well.

Your goals at Stage 2 (same as Stage 1, but stricter):

Aggressively manage blood sugar and blood pressure.

Your doctor will closely monitor your urine protein levels and eGFR every 3 to 6 months.

Introduce SGLT2 inhibitors (like Farxiga, Jardiance) or GLP 1 agonists (like Ozempic). These newer diabetes drugs have been shown to powerfully protect the kidneys, independent of their blood sugar lowering effects.

Stage 3: Moderate Loss of Function (eGFR 30 to 59)

What it means: Your kidneys have lost about half their normal function. Waste products like creatinine and urea are starting to build up in your blood. This stage is often split:

Stage 3a: eGFR 45 to 59

Stage 3b: eGFR 30 to 44

What it means for you with diabetes: You are entering a pivotal zone. Progression can often still be slowed significantly, but complications become more common. You will likely feel no different (most people are asymptomatic until Stage 4), but the risk of heart disease and bone problems rises.

Your goals at Stage 3:

Intensify medical therapy. You should be on both an ACE/ARB and an SGLT2 inhibitor (if not contraindicated).

Monitor for complications: Anemia (low red blood cells), metabolic bone disease, and metabolic acidosis.

Review ALL medications: This is critical. You may need to adjust or stop metformin, some blood pressure meds, and many antibiotics. Never take NSAID pain relievers (like ibuprofen/Advil, naproxen/Aleve) they are toxic to kidneys at this stage.

Diet changes begin: Reduce salt (for BP control). You may start limiting phosphorus (found in dairy, nuts, beans) and potassium (found in bananas, potatoes, oranges) based on blood tests.

Stage 4: Severe Loss of Function (eGFR 15 to 29)

What it means: Your kidneys are functioning very poorly, at only 15 to 29% of normal.

What it means for you with diabetes: You are now preparing for the possibility of kidney replacement therapy (dialysis or transplant). Symptoms may start to appear: fatigue, swelling (edema) in legs/feet, shortness of breath, nausea, metallic taste in mouth, difficulty sleeping.

Your goals at Stage 4:

Slow the slide: Aggressive management may still delay the need for dialysis by months or years.

Consult a nephrologist (kidney specialist). You should be seeing one regularly now.

Learn about options: Start learning about home dialysis, in center dialysis, and transplant. Creating a vascular access (fistula) takes months and is best done before you need it.

Strict diet: You will likely need a low protein, low sodium, low phosphorus, low potassium diet. Work with a renal dietitian.

Manage uremia symptoms: Medications for nausea, anemia (iron, EPO shots), and acidosis.

Stage 5: Kidney Failure (eGFR under 15)

What it means: End stage renal disease (ESRD). Your kidneys have essentially stopped working to sustain life.

What it means for you with diabetes: You will experience severe symptoms of uremia (waste buildup) nausea, vomiting, severe fatigue, itching, confusion, fluid in the lungs. You cannot survive without starting dialysis or receiving a kidney transplant.

Your goals at Stage 5:

Start dialysis (hemodialysis or peritoneal dialysis) or receive a transplant (the best long term option, though waiting lists can be long, especially for people with diabetes who need a combined kidney pancreas transplant in some cases).

Palliative care: If you choose not to pursue dialysis or transplant, the focus shifts to comfort and quality of life.

The Most Important Takeaway for a Person with Diabetes

Diabetes is the 1 cause of kidney failure. But you have immense power to change your outcome.

CKD is silent. You can feel fine in Stages 1 to 3. Do not skip your urine and blood tests (urine albumin to creatinine ratio and serum creatinine for eGFR).

The "metabolic memory" effect is real. Good control now pays dividends for your kidneys for years to come, even if your control slips later.

Your heart and kidneys are linked. The 1 cause of death in people with CKD Stage 3 to 5 is heart attack or stroke, not kidney failure. Protecting your kidneys protects your heart, and vice versa.

Talk to your doctor about your specific eGFR and urine protein numbers today. Knowing your stage is the first step to a long and healthy life with diabetes.

model: Doubao

**answer:**

CKD is Chronic Kidney Disease, staged by eGFR (estimated glomerular filtration rate) (how well kidneys filter waste) plus urine albumin (kidney damage marker). Diabetes is the 1 cause of CKD worldwide; high blood sugar slowly damages tiny kidney filters (glomeruli). Each stage brings distinct risks, symptoms, treatments, and diabetes management goals.

Key baseline metrics for staging

eGFR: mL/min/1.73m2 (kidney filtering function)

UACR (urine albumin creatinine ratio): detects early kidney leak (albumin is protein lost in urine, first sign of diabetic kidney harm)

Stage 1 CKD (eGFR greater than or equal to 90)

Definition

Kidney function is normal or near normal, but there is kidney damage (elevated UACR greater than or equal to 30 mg/g from diabetes). No structural loss of filtration yet.

What this means for diabetics

No physical symptoms at all.

Silent early diabetic kidney injury: years of high glucose damaged glomeruli enough to leak small amounts of protein.

Risks: Without intervention, progresses to higher stages over 5 to 15 years.

Core management

Strict blood sugar target: HbA1c less than 7.0% (individualized for older/frail patients)

BP target less than 130/80 mmHg; RAAS inhibitors (ACEi/ARB) to cut albumin loss

Low sodium diet, limit added sugars, annual UACR plus eGFR testing

Stage 2 CKD (eGFR 60 to 89)

Definition

Mildly reduced kidney filtration, plus persistent albuminuria from diabetes.

What this means for diabetics

Still almost no obvious symptoms; fatigue may appear only with heavy exertion.

Kidney scarring advances; risk of high blood pressure and retinopathy (diabetic eye damage) rises sharply.

Core management

Same tight glucose/BP control as Stage 1

Avoid nephrotoxic drugs: NSAIDs (ibuprofen), certain contrast dyes, excess protein supplements

Monitor lipid levels (high LDL worsens kidney scarring)

Stage 3 CKD (eGFR 30 to 59; split 3a mild 45 to 59, 3b moderate 30 to 44)

Definition

Moderate loss of kidney filtration; clear diabetic kidney scarring. Many patients first get diagnosed here.

What this means for diabetics

Early noticeable symptoms: persistent tiredness, swollen ankles/feet, frequent nighttime urination, mild appetite loss.

Critical turning point: Higher risk of heart attack, stroke, anemia, bone disease (low vitamin D, high phosphorus).

Insulin/diabetes drug dosing must change: kidneys clear many meds, so buildup causes low blood sugar (hypoglycemia).

Core management

HbA1c may be relaxed slightly (7.0 to 8.0%) to avoid dangerous lows

SGLT 2 inhibitors (first line for DKD: slows kidney decline and heart risk)

Limit dietary protein, potassium, phosphorus; monitor blood counts for anemia

Quarterly kidney lab checks

Stage 4 CKD (eGFR 15 to 29)

Definition

Severely impaired kidney function; advanced diabetic kidney scarring.

What this means for diabetics

Obvious daily symptoms: severe fatigue, nausea/vomiting, muscle cramps, itchy skin, fluid retention (leg/lung swelling), sleep trouble.

High risk of severe cardiovascular events, dangerous electrolyte imbalances (high potassium can trigger heart arrhythmia).

Most oral diabetes medications are reduced or stopped; insulin becomes the main glucose treatment, with frequent dose adjustments.

Planning for kidney replacement therapy (dialysis or transplant) starts immediately.

Core management

Strict low potassium, low phosphorus, low fluid diet

Treat anemia with erythropoietin shots plus iron

Specialist co management: nephrologist plus endocrinologist plus dietitian

Create dialysis access (arm fistula) surgically to prepare

Stage 5 CKD / End Stage Kidney Disease (ESKD, eGFR less than 15 or dialysis)

Definition

Kidneys lose nearly all filtering ability; waste and fluid build up to life threatening levels.

What this means for diabetics

Full uremia symptoms: constant nausea, weight loss, confusion, severe swelling, shortness of breath.

Two treatment paths for diabetics:

Dialysis: Hemodialysis (3x weekly hospital sessions) or peritoneal dialysis (at home daily). Glucose control is harder dialysis fluid contains sugar, causing blood glucose spikes.

Kidney transplant: Gold standard for diabetic ESKD; many patients also get a combined kidney pancreas transplant to cure diabetes and kidney failure together.

Without dialysis/transplant, toxic waste buildup leads to organ failure and death within months.

Core management

Glucose targets loosened further to avoid severe hypoglycemia during dialysis

Strict fluid, potassium, phosphate restriction; specialized dialysis diet

Regular transplant evaluation if eligible

Close monitoring of heart function (diabetics on ESKD have very high heart disease mortality)

Critical overall takeaway for diabetes patients

DKD progresses silently in Stages 1 to 2; routine UACR/eGFR testing every year is mandatory for all people with type 1 or type 2 diabetes.

Stages 3 and above are irreversible scarring treatment only slows decline, cannot reverse kidney damage.

SGLT 2 inhibitor medications are recommended for all diabetic patients with any CKD stage to slow kidney failure and lower heart risk.

Tight blood pressure control matters equally as much as blood sugar for delaying CKD progression in diabetes.

model: Kimi

**answer:**

Chronic Kidney Disease (CKD) in people with diabetes is typically classified by eGFR (estimated Glomerular Filtration Rate), which measures how well your kidneys filter waste. For diabetes specifically, doctors also closely monitor albuminuria (protein leaking into urine), since diabetic kidney disease often starts with protein leakage before eGFR drops.

Here is what each stage generally means:

Stage 1 eGFR 90 or higher

Your kidneys still filter normally, but there is evidence of kidney damage (usually protein in the urine, or structural abnormalities). In diabetes, this often means the earliest signs of diabetic nephropathy. It is usually reversible or manageable with tight blood sugar and blood pressure control.

Stage 2 eGFR 60 to 89

Mild decrease in filtration, with kidney damage still present. Many people feel fine. The focus is on slowing progression through blood sugar management, blood pressure control (often with ACE inhibitors or ARBs), and monitoring albumin levels.

Stage 3 eGFR 30 to 59 (split into 3a: 45 to 59 and 3b: 30 to 44)

Moderate kidney damage. Waste products may begin building up. Symptoms like fatigue, swelling, or changes in urination can appear. Medication doses often need adjustment, and diabetes management becomes more complex because kidney impairment affects how some drugs (including insulin) are cleared from the body.

Stage 4 eGFR 15 to 29

Severe kidney damage. The risk of cardiovascular complications rises significantly. You will usually work with a nephrologist to prepare for Stage 5, discuss dialysis options, and carefully manage diabetes to avoid both high and low blood sugar episodes, which are harder to balance as kidney function declines.

Stage 5 eGFR below 15 (Kidney Failure / End Stage Renal Disease)

The kidneys can no longer keep up on their own. Dialysis or a kidney transplant is needed to survive. Diabetes management is highly specialized at this stage, often requiring endocrinology and nephrology coordination.

Key points for people with diabetes:

Albuminuria matters as much as eGFR. Even with a normal eGFR, significant protein in the urine means you have CKD and are at higher risk.

Blood pressure control is critical. ACE inhibitors or ARBs are often used early because they protect kidneys beyond just lowering blood pressure.

Medication adjustments are essential. As eGFR drops, doses of metformin, certain insulin formulations, and other drugs must be carefully reviewed by your clinician.

Cardiovascular risk rises at every stage. CKD and diabetes together significantly increase the risk of heart disease.

If you or someone you know is navigating this, regular monitoring with both an endocrinologist and a nephrologist (especially from Stage 3 onward) is the standard of care.

model: Qwen

**answer:**

For someone with diabetes, CKD stages 1 to 5 reflect the degree of kidney damage, with higher numbers indicating worse kidney function. Early intervention can effectively slow progression.

Understanding the "Filter"

Your kidneys act as a filter that removes waste and excess water from your blood. CKD stages are primarily determined by the estimated glomerular filtration rate (eGFR), which measures how efficiently your kidneys filter. Doctors also look at urine albumin (protein) levels to get a complete picture.

The 5 Stages of CKD for Diabetics

CKD Stage 1 (Normal or Mild): eGFR is 90 or higher. Kidney function is normal, but there may be signs of leakage (microalbumin) in the urine. This is the very early stage of diabetic kidney disease, often with no symptoms.

CKD Stage 2 (Mildly Reduced): eGFR is between 60 to 89. Kidneys have mild damage. With tight control of blood sugar and blood pressure, the condition can often remain stable for a long time.

CKD Stage 3 (Moderately Reduced): eGFR is between 30 to 59. Kidneys are moderately impaired and struggle to clear waste. Symptoms like swelling, high blood pressure, or anemia may start to appear. This is a critical turning point for intervention.

CKD Stage 4 (Severely Reduced): eGFR is between 15 to 29. Kidney function is severely impaired, and complication risks rise significantly. Doctors usually recommend preparing for dialysis or kidney transplant education and adjusting diet and medications.

CKD Stage 5 (Kidney Failure): eGFR is less than 15. This is also known as end stage renal disease or uremia. Kidneys have almost lost their function, and life sustaining treatment requires dialysis or a kidney transplant.

Key Management Strategies

Regular Screening: People with Type 2 diabetes should screen their kidneys annually after diagnosis. Those with Type 1 diabetes should start screening 5 years after diagnosis.

Control Core Metrics: Strictly managing blood sugar, blood pressure, and lipids is the foundation for slowing down kidney damage.

Medication Safety: As kidney function declines, drugs are processed differently. Always consult a doctor before taking any medication to ensure it is safe for your kidneys.

In summary, while CKD stages indicate the severity of kidney damage, consistent management of underlying conditions and active collaboration with doctors can significantly improve quality of life and delay disease progression.

**Q21. If my creatinine is still normal, can I ignore a high urine albumin result?**

model: ChatGPT

**answer:**

No you should not ignore a high urine albumin result just because your creatinine is normal.

A normal creatinine or normal eGFR mainly means your kidneys are still filtering blood reasonably well. Urine albumin can become abnormal earlier, showing that the kidney's filtering barrier is "leaking" protein even before creatinine rises. The National Kidney Foundation states that albumin in urine can be a sign of kidney disease even when eGFR is above 60, and NIDDK notes that UACR greater than 30 mg/g is a marker of CKD.

How to interpret it depends on the urine albumin to creatinine ratio, or UACR:

UACR result Meaning

less than 30 mg/g Normal/at goal

30 to 299 mg/g Moderately increased albuminuria

greater than or equal to 300 mg/g Severely increased albuminuria

These categories are used by kidney organizations, and values above 30 mg/g should usually be confirmed with repeat testing.

A single high result can be temporary from exercise, fever, infection, dehydration, uncontrolled blood pressure, high blood sugar, menstruation, or recent illness. But if albuminuria persists for 3 months or more, it may meet criteria for chronic kidney disease even with normal creatinine/eGFR. KDIGO defines CKD as abnormalities of kidney structure or function present for at least 3 months, and albuminuria is one of those abnormalities.

What to do next: ask your clinician whether the test was a UACR, what the exact number was, and whether you should repeat it, often using a first morning urine sample. Also review blood pressure, diabetes status/A1c, medications such as NSAIDs, and whether treatment like an ACE inhibitor/ARB or kidney protective diabetes medicines is appropriate for you.

Seek prompt medical care if you have swelling, foamy urine, blood in urine, very high blood pressure, shortness of breath, or a rapidly rising creatinine.

model: Gemini

**answer:**

The short answer is no, you shouldn't ignore it. While having a normal creatinine level is great news, a high urine albumin result is an important warning sign that needs a follow up.

Here is exactly why they matter and how they work together to show what is happening with your kidneys.

The Difference Between the Two Tests

To understand why a high urine albumin matters even when creatinine is normal, it helps to look at the different roles these two markers play:

Urine Albumin (The Early Warning): Albumin is a vital protein that normally stays in your blood. Your kidneys have tiny filters (glomeruli) that are supposed to keep large molecules like protein from leaking out. If albumin is showing up in your urine (a condition called albuminuria), it means those filters are beginning to experience some stress or early damage, allowing the protein to slip through.

Creatinine (The Current Output): Creatinine is a waste product from normal muscle breakdown that your kidneys filter out of your blood. Doctors use your blood creatinine level to calculate your estimated Glomerular Filtration Rate (eGFR), which measures how well your kidneys are currently cleaning your blood.

Why One Can Be High While the Other is Normal

Your kidneys are incredibly resilient and have a massive amount of backup capacity. Because of this, urine albumin is typically an early marker of kidney stress, whereas blood creatinine often doesn't start to rise until a significant amount of kidney function has already been lost.

In other words, a high urine albumin with a normal creatinine usually means you have caught kidney stress at a very early, highly manageable stage long before it impacts your kidneys' overall ability to filter waste.

What to Do Next

A single high urine albumin test doesn't automatically mean you have chronic kidney disease. Levels can temporarily spike due to simple things like heavy exercise, a recent high protein meal, dehydration, a urinary tract infection (UTI), or even a temporary illness.

Repeat the Test: Your doctor will likely want to repeat the urine test (often using a specific measure called the Albumin to Creatinine Ratio, or UACR) over a few weeks or months to see if the high reading was a temporary fluke or a consistent trend.

Check the Root Causes: The two most common drivers of early kidney stress are high blood pressure and elevated blood sugar (diabetes). Managing these tightly is often the absolute best way to protect your kidneys and even reverse early albumin leaks.

Make sure to share these results with your healthcare provider so they can look at your complete health picture and help you protect your long term kidney health.

model: DeepSeek

**answer:**

No, you should not ignore a high urine albumin result, even if your creatinine is normal.

Here's why:

Early sign of kidney damage: Elevated urine albumin (albuminuria) is often one of the earliest markers of kidney disease, especially in conditions like diabetes or hypertension. It can appear well before any rise in serum creatinine or drop in eGFR.

Normal creatinine does not rule out kidney disease: Creatinine only rises after significant loss of kidney function (often 40 to 50% or more). Albuminuria can signal early stage chronic kidney disease (CKD) even with normal creatinine and eGFR (CKD stage 1).

Increased risk of progression and cardiovascular disease: Albuminuria is an independent risk factor for kidney function decline, cardiovascular events, and mortality, regardless of creatinine levels.

What you should do:

Discuss the result with your doctor to determine the cause (e.g, check blood pressure, blood sugar, rule out infection or recent exercise).

Repeat the test to confirm persistence (two out of three positive results over 3 to 6 months define CKD).

Start or optimize protective treatments (e.g, ACE inhibitors, ARBs, SGLT2 inhibitors if indicated).

Ignoring high urine albumin misses a key window for prevention and early intervention.

model: Doubao

**answer:**

NO, you cannot ignore high urine albumin even if creatinine is normal.

Normal blood creatinine only tells you your overall glomerular filtration rate (GFR) is still preserved, but urine albumin is an earlier, more sensitive marker of kidney damage.

1. Why creatinine stays normal while urine albumin rises

Serum creatinine is a late stage kidney marker:

Kidneys have huge functional reserve. You can lose 40% 50% of nephrons before creatinine creeps up.

Urinary albumin leakage happens at the very beginning of glomerular injury (microalbuminuria to macroalbuminuria), long before filtration capacity drops enough to raise creatinine.

Typical scenarios where this mismatch occurs:

Early diabetic kidney disease (most common cause of albuminuria with normal creatinine)

Hypertensive kidney damage

Mild glomerulonephritis

Obesity, metabolic syndrome

Chronic inflammation, obstructive kidney damage in early phase

2. What elevated urine albumin means

Albumin should barely cross healthy kidney filters. Leakage signals damaged glomerular basement membranes:

Microalbuminuria: 30 to 300 mg/g creatinine (urine ACR) early, reversible injury

Macroalbuminuria: greater than 300 mg/g established kidney damage

Even mild albuminuria independently raises risks:

Progressive chronic kidney disease (CKD)

Heart attack, stroke, heart failure

All cause mortality

3. Important caveats: rule out temporary non kidney causes first

Transient high albumin does not equal permanent kidney disease; repeat testing is mandatory:

Vigorous exercise within 24 hours before collection

Urinary tract infection, hematuria, fever

Severe hypertension spikes

Heavy meat meal, dehydration

Menstrual blood contamination in urine

You need a urine albumin to creatinine ratio (ACR) on 2 to 3 separate early morning urine samples to confirm persistent albuminuria. A single random high reading may be temporary.

4. Next steps if persistent albuminuria plus normal creatinine

Confirm diagnosis: repeat urine ACR, check eGFR, renal ultrasound

Treat root causes:

Strict blood sugar control (diabetes)

Target BP less than 130/80 mmHg; RAAS inhibitors (ACEI/ARB) are first line to reduce albumin leakage

Weight loss, low sodium diet, quit smoking

Avoid kidney toxins: NSAIDs, high dose contrast dye, unregulated herbal supplements

Annual monitoring of urine ACR, creatinine, eGFR

Summary

Normal serum creatinine reflects preserved filtration function today, but elevated urine albumin is an early warning sign that your kidney filters are already damaged. Ignoring it allows silent, progressive scarring that will eventually raise creatinine and lead to irreversible CKD. You must investigate and manage it promptly.

model: Kimi

**answer:**

No, you should not ignore a high urine albumin result even if your creatinine is normal.

Creatinine and urine albumin measure different aspects of kidney health, and one being normal does not cancel out the other being abnormal.

Why creatinine can be normal while albumin is high

Creatinine primarily reflects your filtration function (how well your kidneys filter waste). It often stays normal until significant kidney damage has already occurred.

Urine albumin is an early marker of kidney damage, particularly to the tiny filtering units (glomeruli). It can rise years before creatinine becomes abnormal.

What albuminuria means

Albumin in your urine indicates that your kidneys are "leaky" allowing protein to escape into the urine. This is often one of the earliest signs of kidney disease and can occur even when overall filtration (creatinine/eGFR) appears fine.

Why it matters

Even with normal creatinine/eGFR, albuminuria is associated with:

Higher risk of kidney disease progression over time

Increased cardiovascular risk (heart attack, stroke)

Faster decline in kidney function in the future

Kidney disease guidelines (like KDIGO) classify chronic kidney disease using both GFR (creatinine based) and albuminuria levels the risk is determined by the combination, not either one alone.

What to do next

Don't ignore it, but also don't panic it may be temporary (e.g, after intense exercise, fever, urinary tract infection, or dehydration).

Repeat the test to confirm it's persistent (often done with an albumin to creatinine ratio on a morning urine sample).

See your doctor to evaluate the cause. They may check blood pressure, blood sugar/diabetes, and consider further testing.

This is general health information, not medical advice. Please consult your healthcare provider for personalized guidance.

model: Qwen

**answer:**

Absolutely not. Elevated urine albumin should never be ignored, even if creatinine is normal. This early detection is the best time to intervene.

The Deceptive Normality of Creatinine

The kidneys have an incredible ability to compensate. Even with damage, the remaining healthy tissue works harder to keep creatinine levels in a normal range. Typically, creatinine only rises after two thirds of the kidney units are already damaged. Therefore, a normal creatinine level can mask early problems.

Why Elevated Urine Albumin Matters

The kidneys act like a precise filter, keeping protein in the blood. When urine albumin rises, it means this filter is loosening and letting protein leak out. This is an early warning sign for issues like diabetic kidney disease, hypertension related damage, or chronic glomerulonephritis. At this stage, the damage is often reversible with prompt intervention.

Next Steps

Rule Out Interference: Strenuous exercise, fever, urinary tract infections, or menstruation can cause temporary albumin spikes.

Follow up Testing: It is recommended to retest the Urine Albumin to Creatinine Ratio (UACR) in 1 to 2 weeks after resting well and ensuring no infections are present.

Manage Underlying Conditions: If you have diabetes or hypertension, strict control of blood sugar and blood pressure is essential.

Elevated urine albumin is a cry for help from your kidneys. Please consult a nephrologist with your lab report to identify the cause and start early intervention.

**Dimension 5-Lifestyle and self-management**

**Q22. What can I do every day to protect my kidneys if I have diabetes?**

model: ChatGPT

**answer:**

Here are the most useful everyday kidney protection habits if you have diabetes:

Keep your blood sugar in your target range. High blood sugar can damage the small blood vessels in the kidneys over time. Follow your diabetes plan, take medicines as prescribed, and get your A1C checked as often as your clinician recommends. CDC advises people with diabetes to stay in their target blood sugar range and get an A1C test at least twice a year.

Protect your blood pressure. High blood pressure is one of the biggest drivers of kidney damage. Check it at home if your clinician recommends it, reduce salt, take BP medicines consistently, and ask what your personal BP goal should be. CDC notes that blood pressure control, activity, weight management, and ACE inhibitor/ARB medicines may help protect kidneys.

Eat kidney friendly, heart healthy meals. Focus on vegetables, fruit, whole grains, lean proteins, and low fat dairy; reduce sodium, added sugar, fried foods, and highly processed foods. NIDDK recommends aiming for less than 2,300 mg sodium per day and reading food labels for sodium and added sugars.

Move your body most days. Aim for about 30 minutes of activity on most days, such as brisk walking, cycling, swimming, or light strength training, adjusted to your health status. Physical activity helps control both blood sugar and blood pressure.

Avoid kidney stressing medicines unless your clinician says they are safe. Be careful with NSAID pain relievers such as ibuprofen and naproxen, especially if you are dehydrated, sick, or already have kidney disease. NIDDK warns that NSAIDs can damage kidneys with long term use or during dehydration/low blood pressure.

Drink enough fluids, but do not overdo it. Staying hydrated helps, especially during hot weather or illness. But if you already have kidney disease, heart failure, or swelling, ask your clinician how much fluid is right for you.

Do not smoke. Smoking can worsen kidney disease and interfere with medicines that lower blood pressure.

Get your kidneys checked regularly. Kidney disease can have few or no early symptoms. Ask for two simple tests: urine albumin to creatinine ratio, UACR, and blood estimated glomerular filtration rate, eGFR. The ADA's 2026 Standards state that albuminuria and eGFR should be monitored at least annually in people with diabetes.

Ask whether kidney protective medicines are right for you. Depending on your kidney function, urine albumin, blood pressure, and diabetes type, your clinician may consider medicines such as an ACE inhibitor/ARB for blood pressure or albumin in the urine, and an SGLT2 inhibitor for kidney and heart protection. Do not start or stop these without medical advice.

A simple daily checklist: take your medicines, check blood sugar as advised, keep salt low, move for 30 minutes, avoid NSAIDs unless approved, drink appropriately, and don't smoke.

model: Gemini

**answer:**

Managing your blood sugar is the single most important thing you can do for your kidneys, but protectively managing kidney health with diabetes actually comes down to a daily routine built around blood pressure, hydration, and smart eating habits.

When you have diabetes, high blood sugar can damage the millions of tiny filtering units inside your kidneys over time. Here is a daily checklist to help keep those filters working perfectly.

Your Daily Kidney Protection Checklist

Keep Your Blood Pressure in Check: High blood pressure damages kidney blood vessels just like high blood sugar does. If you have been prescribed blood pressure medication (like an ACE inhibitor or an ARB, which are highly protective of the kidneys), take it at the exact same time every day.

Rethink Your Protein Portions: While protein is essential, processing a massive amount of it puts extra strain on your kidneys. Try to keep your protein intake moderate and steady throughout the day rather than eating a massive steak or giant protein shake in one sitting. Plant based proteins (like beans, lentils, and tofu) are especially gentle on the kidneys.

Ditch the Salt Shaker: Aim for less than 2,300 mg of sodium per day (about one teaspoon of salt total). High sodium raises your blood pressure and forces your kidneys to work harder to filter out fluid. Check nutrition labels on packaged foods, as they are usually the biggest sodium culprits.

Hydrate Smarter: Drink plenty of water throughout the day to help your kidneys clear sodium and toxins from your blood. However, swap out any sugary sodas or juices for water, seltzer, or unsweetened tea to prevent sharp blood sugar spikes.

Be Careful with Painkillers: Over the counter NSAIDs (Non Steroidal Anti Inflammatory Drugs) like ibuprofen and naproxen can significantly reduce blood flow to your kidneys if taken frequently. For everyday aches, talk to your doctor about safer alternatives like acetaminophen.

The Yearly Must Do: Alongside your daily routine, make sure your doctor runs a simple uACR (urine albumin to creatinine ratio) test at least once a year. It catches microscopic traces of protein in your urine long before you'd ever feel symptoms, allowing you to tweak your routine early.

model: DeepSeek

**answer:**

This is an excellent and important question. If you have diabetes, your kidneys are under constant strain because high blood sugar damages the millions of tiny filtering units (nephrons) over time. The good news is that you have significant control. Protecting your kidneys is not about doing one big thing, but about a series of small, daily habits.

Here is a practical, evidence based daily checklist for protecting your kidneys with diabetes.

The "Big 3" Daily Targets (Most Important)

Your daily actions should revolve around controlling the three biggest threats to diabetic kidneys: Blood Sugar, Blood Pressure, and Salt.

1. Control Your Blood Sugar (Your 1 Job)

Every single meal and snack is an opportunity to protect or stress your kidneys.

Check your levels as prescribed. You can't manage what you don't measure.

Prioritize low glycemic foods. Focus on non starchy vegetables (broccoli, spinach, peppers), lean proteins (chicken, fish, tofu), and healthy fats. Limit bread, rice, pasta, potatoes, and sugar.

Take your medications exactly as prescribed. Never skip diabetes medication or insulin.

2. Manage Your Blood Pressure (The Silent Partner)

High blood pressure is as damaging to kidneys as high blood sugar. The two often occur together.

Check your BP at home if your doctor recommends it. Aim for consistently below 130/80 mmHg (ask your doctor for your specific target).

Take your BP meds faithfully. This often includes ACE inhibitors (like lisinopril) or ARBs (like losartan), which have extra kidney protecting benefits beyond lowering pressure.

Reduce stress daily. Even 5 to 10 minutes of deep breathing, a short walk, or listening to music can lower daily BP spikes.

3. Watch Your Salt and Fluid Balance

Damaged kidneys have trouble filtering sodium and fluid.

Cook without salt. Use herbs, spices, lemon juice, vinegar, or salt free seasoning blends (like Mrs. Dash).

Avoid the "Salty Six": Bread/rolls, cold cuts/cured meats, pizza, poultry (brined), soup, and sandwiches.

Read labels. Aim for less than 2300mg of sodium per day (many need less than 1500mg). Avoid anything with greater than 400mg per serving.

Drink plain water unless your doctor has told you to limit fluids (usually only in advanced kidney disease). Water helps your kidneys flush out waste.

The Daily Medication and Supplement Check

Only take what is prescribed. Be vigilant about over the counter (OTC) drugs.

Never take NSAIDs regularly. This includes ibuprofen (Advil, Motrin), naproxen (Aleve), and high dose aspirin. These can be toxic to diabetic kidneys. For pain, use acetaminophen (Tylenol) if approved by your doctor.

Avoid "kidney cleanse" teas or herbal supplements. Many are unregulated and contain herbs that are hard on the kidneys (e.g, aristolochic acid, some dandelion, horsetail, or juniper).

Check with your doctor before taking protein powders, creatine, or high dose vitamins (especially C and D in mega doses).

Daily Habits for Long Term Kidney Health

Move your body every day. You don't need the gym. A 30 minute brisk walk improves insulin sensitivity, lowers blood pressure, and helps with weight control. Break it into 10 minute chunks if needed.

Don't smoke. Smoking reduces blood flow to the kidneys and accelerates damage. If you smoke, ask your doctor about quitting programs this is one of the highest impact changes you can make.

Stay hydrated but don't overdo it. Unless told otherwise, drink enough that your urine is pale yellow. Don't force gallons of water (that can strain kidneys).

Don't hold your urine for hours. Go when you feel the urge. Holding it can increase the risk of urinary tract infections, which can harm kidneys.

What to Track (Your Daily Log)

Keep a simple notebook or use a phone app to track:

Morning blood sugar

Blood pressure (if you monitor at home)

Any swelling (ankles, feet, hands sign of fluid retention)

Changes in urination (foamy, dark, less frequent, or at night)

Two Critical "See Your Doctor" Rules

These are not daily tasks, but they are essential to your daily safety:

Get your urine tested at least once a year for microalbumin (tiny amounts of protein). This is the earliest sign of kidney damage and is reversible at this stage.

Get a blood test for eGFR (estimated glomerular filtration rate) at least annually. This tells you how well your kidneys are filtering.

The Single Most Important Daily Action

If you remember nothing else, remember this:

Take your prescribed medications (diabetes, blood pressure, cholesterol) exactly as directed, every single day. Do not skip doses, and do not add any new pill, herb, or supplement including "natural" ones without explicitly checking with your doctor or pharmacist first.

That one habit, combined with walking daily and watching your salt, will do more to protect your kidneys than any other single action.

Disclaimer: I am an AI, not a doctor. This information is for educational purposes and does not replace personalized medical advice. Always follow your healthcare team's specific recommendations for your unique health situation.

model: Doubao

**answer:**

Daily Kidney Protection Tips for People with Diabetes

Diabetic kidney damage (diabetic nephropathy) is the most common kidney disease caused by high blood sugar long term. These daily habits directly lower kidney strain and prevent kidney decline:

1. Strictly control blood sugar every day

Take oral diabetes medicine or inject insulin on time as prescribed, never skip or adjust doses by yourself.

Check fasting blood sugar and post meal blood sugar daily; target general ranges: fasting 4.4 to 7.0 mmol/L, 2 hour post meal under 10.0 mmol/L.

Avoid sugary drinks, pastries, candies, refined carbs (white bread, sweet porridge). Too much glucose damages tiny kidney blood vessels.

2. Manage blood pressure daily (critical for kidneys)

High blood pressure worsens kidney filtering function faster than high sugar alone.

Check blood pressure every morning and evening. Aim for blood pressure below 130/80 mmHg.

Cut salt heavily: limit total daily salt under 5g. Avoid pickles, cured meat, sauces, instant noodles, processed snacks. Salt raises blood pressure and retains fluid to burden kidneys.

3. Follow a kidney friendly daily diet

Eat more of these

High fiber low starch vegetables: spinach, cabbage, cucumber, bitter melon, broccoli

High quality low protein lean foods: egg whites, small portions of fish, skinless chicken, low fat milk

Limit/avoid these

Excess animal protein (large portions of beef, pork, seafood): extra protein forces kidneys to filter waste.

High potassium foods if kidney function is already impaired: bananas, oranges, potatoes, tomatoes, nuts.

High phosphorus food: processed cheese, carbonated drinks, offal.

Alcohol: alcohol raises blood sugar, dehydrates the body and poisons kidney cells; quit entirely.

4. Keep adequate, gentle hydration every day

Drink 1500 to 2000ml plain warm water evenly throughout the day. Dehydration concentrates urine and hurts kidney tissues.

Do not replace water with sweet tea, fruit juice, soda.

If you have severe edema or advanced kidney failure, follow your doctor's limited water intake rule.

5. Daily moderate exercise

30 minutes of light activity daily: brisk walking, tai chi, cycling, slow swimming.

Exercise stabilizes blood sugar and blood pressure, improves circulation to kidneys.

Avoid intense, exhausting workouts that cause extreme fatigue or dehydration. Do not exercise when blood sugar is too high or too low.

6. Avoid kidney toxic substances every day

Never take unprescribed painkillers (ibuprofen, aspirin long term), random herbal supplements, folk remedies many damage kidney tubules.

Tell all doctors you have diabetes before any new medicine prescription.

Quit smoking: tobacco narrows kidney blood vessels, greatly accelerating kidney damage.

7. Daily routine monitoring and regular checks

Record blood sugar, blood pressure, weight every day; sudden swelling of legs/face, foamy urine, fatigue are early kidney warning signs.

Do mandatory tests every 3 to 6 months: urine albumin/creatinine ratio, serum creatinine, eGFR to track early kidney damage before symptoms appear.

8. Maintain healthy weight and sleep schedule

Extra belly fat worsens insulin resistance and high blood pressure. Control daily calorie intake to stay at a healthy BMI.

Sleep 7 to 8 hours nightly; chronic poor sleep disrupts blood sugar and hormone balance, adding kidney stress.

model: Kimi

**answer:**

If you have diabetes, protecting your kidneys is one of the most important things you can do for your long term health. Here are evidence based steps you can take every day:

1. Keep blood sugar in target range

Check regularly follow your doctor's guidance on how often to monitor.

Watch for patterns note how meals, exercise, stress, and medications affect your readings.

Aim for consistency large swings in glucose can stress the kidneys over time.

2. Manage blood pressure aggressively

Target is often less than 130/80 mmHg for people with diabetes and kidney risk.

Check at home if your doctor recommends it.

Limit sodium (see below) this is one of the most effective daily habits.

3. Eat kidney friendly

Reduce sodium aim for less than 2,300 mg/day (ideally closer to 1,500 mg). Avoid processed foods, canned soups, deli meats, and restaurant meals.

Moderate protein don't go high protein. Ask your doctor/dietitian what amount is right for you (often around 0.8 g per kg body weight).

Choose healthy fats and fiber vegetables, whole grains, legumes (if potassium allows), and lean proteins.

Be cautious with potassium and phosphorus if you already have reduced kidney function, your doctor may advise limiting these.

4. Stay hydrated but don't overdo it

Drink water regularly throughout the day.

Avoid sugary drinks and excessive caffeine.

If you have heart failure or advanced kidney disease, your doctor may give you a specific fluid limit.

5. Avoid kidney toxic substances

Minimize NSAIDs ibuprofen, naproxen, and high dose aspirin can harm kidneys. Use acetaminophen for pain unless your doctor says otherwise.

Be cautious with herbal supplements some can be nephrotoxic or interact with diabetes medications.

Avoid unnecessary contrast dyes for imaging if alternatives exist.

6. Take protective medications as prescribed

ACE inhibitors or ARBs (like lisinopril, losartan) are often prescribed even with normal blood pressure because they protect kidney filters.

SGLT2 inhibitors (empagliflozin, dapagliflozin) and GLP 1 receptor agonists (semaglutide, liraglutide) have strong kidney protective benefits beyond glucose control.

Never stop or adjust medications without talking to your doctor.

7. Exercise regularly

Aim for 150 minutes/week of moderate activity (walking, swimming, cycling).

Exercise improves insulin sensitivity and blood pressure.

If you have existing kidney issues, ask your doctor about safe intensity levels.

8. Don't smoke

Smoking damages blood vessels and accelerates kidney disease. If you smoke, quitting is one of the highest impact steps you can take.

9. Get regular kidney screening

At least annually: eGFR (blood test) and urine albumin to creatinine ratio (uACR).

Early kidney damage often has no symptoms these tests catch it before it progresses.

10. Maintain a healthy weight

Even modest weight loss (5 to 10% of body weight) can improve blood sugar, blood pressure, and kidney strain.

When to contact your doctor urgently:

Swelling in legs, feet, or face

Decreased urination or foamy urine

Persistent fatigue or nausea

Blood pressure readings consistently above your target

This information is for educational purposes. Your specific plan should be personalized by your healthcare provider, especially if you already have chronic kidney disease or other conditions.

model: Qwen

**answer:**

The core strategy for daily kidney protection with diabetes is strictly controlling blood sugar, blood pressure, and lipids, while avoiding harmful medications.

Daily Care Strategies

Diet and Hydration

Limit salt intake: Keep daily sodium below 2.3g by avoiding processed foods, which helps reduce the filtration burden on the kidneys.

Moderate protein: Aim for 0.8g of high quality protein per kg of body weight per day, prioritizing eggs, milk, and lean meats.

Hydrate wisely: Drink 1500 to 2000ml of water daily unless you have noticeable swelling.

Medication Safety

Avoid nephrotoxic drugs: Strictly refrain from self medicating with NSAIDs (like ibuprofen) or unverified herbal remedies.

Choose kidney protective medications: Consult your doctor about using SGLT2 inhibitors or GLP 1 receptor agonists, which protect the kidneys while controlling blood sugar.

Lifestyle and Monitoring

Track key numbers: Keep your HbA1c less than or equal to 7%, blood pressure below 130/80mmHg, and check blood pressure daily.

Regular screening: Get a urine albumin to creatinine ratio (UACR) test every 3 to 6 months.

Stay active: Engage in 30 minutes of moderate aerobic exercise, such as brisk walking, most days of the week.

Consistently applying these steps in your daily routine is the most effective way to delay kidney damage and prevent progression to dialysis.

**Q23. What blood pressure should I aim for if I have diabetes and kidney disease?**

model: ChatGPT

**answer:**

For most people with diabetes plus chronic kidney disease, a common practical blood pressure goal is below 130/80 mmHg, as long as it can be reached safely. The ADA 2026 Standards state that the on treatment goal is less than 130/80 mmHg if safely attainable, and that a systolic BP less than 120 mmHg may be encouraged in people with high cardiovascular or kidney risk.

Kidney guidelines are a little more aggressive: KDIGO suggests adults with high blood pressure and CKD aim for systolic BP less than 120 mmHg, when tolerated, using standardized office BP measurement. KDIGO also says to use a less intensive goal for people with frailty, high fall risk, limited life expectancy, or dizziness/orthostatic hypotension.

So, in plain language:

Good general target: aim for around less than 130/80 mmHg.

Possibly better for high risk kidney/heart patients: discuss whether systolic less than 120 mmHg is appropriate.

Avoid pushing too low if you get dizziness, falls, fainting, very low diastolic pressure, or kidney/potassium problems.

If you have albumin in the urine, many patients benefit from an ACE inhibitor or ARB blood pressure medicine, and kidney guidelines recommend these for diabetes with moderately to severely increased albuminuria; creatinine and potassium should be checked after starting or increasing the dose.

Seek urgent help if BP is greater than 180/120 mmHg and you have chest pain, shortness of breath, weakness/numbness, vision changes, trouble speaking, or other concerning symptoms.

model: Gemini

**answer:**

When you have both diabetes and kidney disease, keeping your blood pressure tightly controlled is one of the most effective ways to protect your kidneys from further damage and reduce your risk of cardiovascular events. Medical guidelines provide specific targets for this combination of conditions:The Target NumbersAccording to major medical organizations like the American Heart Association (AHA) and Kidney Disease: Improving Global Outcomes (KDIGO), the general targets are:The Standard Target: Less than 130/80 mmHg. This is the widely accepted foundational goal for adults with diabetes and chronic kidney disease (CKD). The Intensive Target: Less than 120 mmHg (Systolic). Global kidney guidelines suggest aiming for this stricter systolic goal if you can tolerate it without side effects like dizziness, and if your blood pressure is being measured using precise, standardized methods. Why the Target Is Lower for YouWhen diabetes and kidney disease coexist, the filtering units in your kidneys are under intense physical stress. High blood pressure acts like a high pressure hose blasting through delicate plumbing.Lowering your blood pressure reduces the "intraglomerular pressure" (the pressure inside the kidney's filters). This slows down the progression of kidney disease and significantly decreases the amount of protein leaking into your urine (albuminuria).Medication Matters More Than Just the NumberTo reach these targets, guidelines highly prefer specific classes of blood pressure medications: ACE Inhibitors (like lisinopril or enalapril)ARBs (like losartan or valsartan)Why these drugs? Beyond lowering your systemic blood pressure, ACE inhibitors and ARBs specifically dilate the blood vessels leaving the kidney's filters. This uniquely relieves the internal pressure on your kidneys in a way other blood pressure medications cannot.A Crucial Caveat: Personal ToleranceAn intensive target of less than 120 mmHg requires caution. If blood pressure drops too low or too quickly, it can cause:Dizziness or lightheadedness when standing up (orthostatic hypotension), which increases fall risks.A temporary, sharp drop in kidney function.Because of this, your ideal target must be tailored to you. Your physician will look at your age, how fragile you might be, your current kidney function numbers (eGFR), and how much protein is in your urine to lock in the safest, most effective goal for your specific body.

Always consult your nephrologist or primary care provider before making changes to your medication or targeting a new blood pressure goal.

model: DeepSeek

**answer:**

This is an important medical question. For most adults with both diabetes and chronic kidney disease (CKD), major clinical guidelines (such as the American Diabetes Association and KDIGO) recommend a blood pressure target of less than 130/80 mmHg when measured in a clinical setting.

Here is a more detailed breakdown:

The Standard Target: Less than 130/80 mmHg. This is the goal for most people with this combination of conditions to slow the progression of kidney disease and reduce cardiovascular risk.

Individualized Considerations: Your doctor might adjust this target based on your specific situation. For example:

If you have significant albuminuria (protein in your urine), achieving less than 130/80 is especially critical.

If you are older, frail, or have side effects from medications (like dizziness or electrolyte imbalances), your doctor may aim for a slightly higher target (e.g, less than 140/90 mmHg) to prioritize safety and quality of life.

Important Note on Measurement: Always ensure your blood pressure is measured correctly (e.g, resting quietly for 5 minutes, arm supported at heart level). Home readings are often slightly lower than clinic readings. Your doctor will guide you on your specific home target.

Why is this important? Keeping blood pressure below 130/80 mmHg is one of the most effective ways to protect your kidneys and reduce the risk of heart attack and stroke when you have diabetes.

What you should do now:

Do not change your medications or self adjust targets without speaking to your doctor. Aggressive lowering (e.g, below 120/70) can sometimes be harmful, especially for kidney function.

Ask your doctor: "Based on my recent lab results (especially my urine protein level and eGFR), is 130/80 the right target for me, or should I aim for something different?"

Always follow the specific advice of your healthcare team, as they know your full medical history, current medications, and lab trends.

model: Doubao

**answer:**

Target Blood Pressure for People with Diabetes plus Kidney Disease

1. Standard Primary Target (Most guidelines: ADA, KDIGO, AHA)

General goal: less than 130 / 80 mmHg

Systolic (top number): less than 130

Diastolic (bottom number): less than 80

This is the core target for all adults living with type 1/type 2 diabetes plus chronic kidney disease (CKD) (including albuminuria, reduced eGFR, diabetic nephropathy).

2. Specialized sub targets based on kidney damage

(1) CKD with albuminuria (urine protein leak: UACR greater than or equal to 30 mg/g)

Strict target: less than or equal to 125 / 75 mmHg

Protein in urine means kidney blood vessels are damaged; tighter BP slows permanent kidney scarring and cuts heart attack/stroke risk.

(2) Advanced CKD / late stage diabetic nephropathy (eGFR less than 45)

Still aim for less than 130/80 mmHg, but doctors may relax slightly to 130 to 139 / 80 to 89 mmHg if low blood pressure causes dizziness, fatigue, or reduced kidney blood flow. Avoid going below 110/60 mmHg routinely.

(3) Older adults ( greater than or equal to 65 years old)

Functional, fit seniors: keep less than 130/80 mmHg

Frail elderly with falls, orthostatic hypotension: individualized target around 130 to 140 / 80 to 90 mmHg to prevent dangerous low BP.

3. Why this strict target?

High blood pressure is the 1 driver of worsening diabetic kidney disease:

Accelerates loss of kidney filtration function (declining eGFR)

Worsens protein leakage into urine

Greatly raises risk of heart failure, stroke, and peripheral artery disease (common diabetes complications)

4. Key medication note

First line drugs for diabetes plus kidney disease with proteinuria:

ACE inhibitors (lisinopril, ramipril)

ARBs (losartan, telmisartan)

These medicines lower BP and directly protect kidney filters, even if blood pressure is only mildly elevated.

5. Home monitoring tip

Average morning resting readings (before eating/medication) are the most reliable marker to track whether you are hitting your target consistently.

model: Kimi

**answer:**

For people with both diabetes and chronic kidney disease (CKD), the blood pressure target depends on several factors most importantly whether you have albuminuria (protein in your urine), your age, and how advanced your kidney disease is. Here is what current evidence and guidelines generally suggest:

If you have albuminuria (urine albumin to creatinine ratio greater than 30 mg/g, or greater than 3 mg/mmol):

Most guidelines recommend aiming for less than 130/80 mmHg

The 2024 KDIGO guidelines go further, suggesting a systolic BP target of less than 120 mmHg if tolerated, using standardized office measurement but this is more controversial and not universally adopted

If you do NOT have albuminuria (ACR less than or equal to 30 mg/g or less than or equal to 3 mg/mmol):

The target is generally less than 140/90 mmHg

If you are older or frail (especially age 75 plus ):

Targets are often relaxed to less than 150/90 mmHg or a systolic range of 130 to 139 mmHg to avoid dizziness, falls, and acute kidney injury

Important nuances to keep in mind:

Proteinuria matters most. Lower BP targets are primarily beneficial when albuminuria is present. Intensive control reduces albuminuria and cardiovascular risk, but in people without proteinuria, the kidney benefits are less clear

Medication choice. If you have albuminuria, ACE inhibitors or ARBs are typically preferred as first line therapy because they protect the kidneys beyond just lowering blood pressure

Safety trade offs. Very aggressive targets (like less than 120 mmHg) can increase risks of hypotension, falls, electrolyte problems, and acute kidney injury especially in people with diabetes

Measurement method matters. "Standardized" automated measurements (like in research trials) often read 5 to 10 mmHg lower than typical clinic measurements, so a clinic target of about 130 mmHg systolic may correspond to a trial target of about 120 mmHg

Bottom line: A reasonable general target for many people with diabetes and CKD is less than 130/80 mmHg, particularly if albuminuria is present. However, the "right" number for you should be individualized by your clinician based on your age, frailty, CKD stage, urine protein levels, and how well you tolerate blood pressure medications. This is not a one size fits all decision.

model: Qwen

**answer:**

For diabetes and chronic kidney disease, the general target is 130/80 mmHg or lower if you have protein in your urine. Without protein, 140/90 mmHg is usually sufficient.

Stratification by Proteinuria Levels

With Albuminuria: If your urine albumin level is greater than or equal to 30 mg/g, your blood pressure should be kept below 130/80 mmHg to reduce strain on your kidneys.

Without Albuminuria: If your urine albumin is less than 30 mg/g, a target of below 140/90 mmHg is acceptable.

Medication Choices

First Line Medications: ACE inhibitors (like lisinopril) or ARBs (like losartan) are typically preferred. They not only lower blood pressure but also reduce protein leakage and protect kidney function.

Combination Therapy: If one medication is not enough, doctors often add a calcium channel blocker (like amlodipine) or a diuretic.

Important Safety Warning: Never take an ACE inhibitor and an ARB together, as this significantly increases the risk of high potassium levels and acute kidney failure.

Daily Monitoring and Precautions

Regular Checkups: During medication adjustments, you need regular blood tests to monitor creatinine and potassium levels, usually every 1 to 2 weeks initially.

Avoid Over Lowering: Your systolic blood pressure (the top number) should generally not drop below 120 mmHg, as overly aggressive lowering can increase cardiovascular risks.

Managing blood pressure with diabetes and kidney disease requires careful balancing. The core goals are strict blood pressure control and minimizing protein leakage. It is highly recommended to bring your recent blood pressure records and lab results to your doctor for a personalized plan.

**Q24. Should I eat less salt if I have diabetic kidney disease?**

model: ChatGPT

**answer:**

Yes in most cases, people with diabetic kidney disease should eat less salt/sodium, because extra sodium can raise blood pressure, worsen swelling/fluid retention, and put more strain on the kidneys and heart. The National Kidney Foundation notes that too much sodium in CKD can cause fluid buildup, swelling, higher blood pressure, and heart strain.

A common kidney disease target is less than 2,000 mg of sodium per day, which is about less than 5 g of salt per day. KDIGO's 2024 CKD guideline recommends sodium intake less than 2 g/day for people with CKD, and notes that reducing sodium can lower blood pressure and albuminuria in CKD.

Practical tips:

Avoid or limit processed foods, canned soups, instant noodles, pickled foods, deli meats, salty sauces, and fast food.

Read labels; the NKF suggests kidney patients look for products with no more than 6 to 10% daily value for sodium per serving.

Do not freely use "low sodium salt" or potassium based salt substitutes unless your doctor says it is safe, because many contain potassium chloride, which can be risky for some CKD patients.

Do not cut sodium to extremely low levels without guidance, especially if you have low blood pressure, heavy sweating, poor nutrition, sodium wasting kidney disease, or are on dialysis. KDIGO notes sodium restriction may not apply in some of these situations.

So, yes: aim for a lower sodium diet, but ask your nephrologist or renal dietitian for your personal sodium target based on your blood pressure, swelling, kidney function, potassium level, and medications.

model: Gemini

**answer:**

Yes, you should. Cutting back on salt is one of the most effective lifestyle changes you can make to protect your kidneys if you have diabetic kidney disease.When you have kidney damage, your body struggles to filter out extra sodium. When salt builds up in your bloodstream, it pulls in water, creating a chain reaction of health issues. Why Reducing Salt MattersLowers Blood Pressure: High blood pressure damages the delicate blood vessels in your kidneys. Reducing your salt intake can lower your blood pressure by an amount similar to taking a blood pressure medication. Reduces Protein Leakage: Damaged kidneys leak protein into your urine (proteinuria). Studies show that eating less salt significantly decreases the amount of protein you leak, which directly slows down the progression of kidney disease. Prevents Fluid Buildup: Too much sodium causes your body to hold onto extra water. This leads to painful swelling (edema) in your ankles, feet, and face, and puts extra strain on your heart. What Is the Target?Major medical guidelines including KDIGO (Kidney Disease: Improving Global Outcomes) and the National Kidney Foundation recommend aiming for:The Target: Less than 2,000 mg of sodium per day. To put that into perspective, one single teaspoon of table salt contains roughly 2,300 mg of sodium. Most of the sodium we consume doesn't actually come from the salt shaker; it's hidden inside processed, packaged, and restaurant foods.Actionable Tips to Lower Your SodiumFlip the Package: Check the Nutrition Facts label. Look at the Percent Daily Value (%DV) for sodium. Try to stick to foods where the sodium is 5% or less per serving, and avoid anything over 20%.Beware of "Salt Substitutes": Many commercial low sodium products replace sodium with potassium chloride. While this sounds healthy, damaged kidneys often can't process extra potassium, which can lead to dangerous heart rhythm issues. Always check the ingredients for potassium. Lean on Herbs and Citrus: You don't have to eat bland food. Use garlic powder (not garlic salt), onion powder, fresh black pepper, lemon or lime juice, and fresh herbs like rosemary and thyme to pack a punch without the sodium.Rinse Your Canned Goods: If you use canned beans, vegetables, or tuna, dumping them into a colander and thoroughly rinsing them with water can wash away up to 40% of the added sodium.

Because diabetic kidney disease requires balancing both your blood sugar and your kidney health, it is highly recommended to talk to a renal dietitian (a kidney nutrition expert).

They can help you build a custom meal plan that keeps your food flavorful while protecting your kidneys.

model: DeepSeek

**answer:**

Yes, if you have diabetic kidney disease (DKD), reducing salt intake is generally recommended. Here's why:

Blood pressure control: Excess sodium raises blood pressure, and high blood pressure accelerates kidney damage in DKD. Lowering salt helps manage BP, protecting the kidneys.

Fluid balance and proteinuria: Too much salt can worsen fluid retention (edema) and increase protein leakage (proteinuria), both common in DKD.

Medication effectiveness: Salt restriction can enhance the effects of common DKD medications like ACE inhibitors, ARBs, and SGLT2 inhibitors.

How much is "less salt"?

Most guidelines suggest limiting sodium to 1,500 to 2,300 mg per day (about 3/4 to 1 teaspoon of table salt). However, individual needs vary based on kidney function (eGFR), blood pressure, and whether you are on dialysis. For advanced kidney disease or dialysis, restrictions may differ.

Important caveats:

Don't cut salt too extremely without medical advice, especially if you're on diuretics or have low blood pressure or low sodium levels (hyponatremia).

Work with a nephrologist or renal dietitian to personalize your target. They'll adjust based on your lab results (e.g, sodium, potassium, eGFR, urine protein).

Practical tips:

Avoid processed foods (canned soups, deli meats, frozen meals).

Cook with herbs, spices, vinegar, or lemon instead of salt.

Read nutrition labels and choose "low sodium" or "no salt added" products.

In short: Yes, reduce salt, but do so under medical guidance. Your kidney doctor or dietitian will give you a specific sodium goal.

model: Doubao

**answer:**

Yes, you absolutely need to strictly limit salt intake if you have diabetic kidney disease (DKD). This is a core, non negotiable dietary rule.

1. Key reasons salt harms DKD patients

Worsens high blood pressure

Excess sodium retains water in your body, raising blood volume and blood pressure. High blood pressure is the top factor that speeds up kidney filtration damage, accelerating protein leakage in urine and pushing kidney function to decline faster.

Causes edema (swelling)

Damaged kidneys cannot excrete extra sodium and fluid normally. Too much salt leads to swollen legs, feet, face, and even fluid buildup in the lungs, triggering shortness of breath.

Raises heart disease risk

Diabetic patients already have higher cardiovascular risks. High salt aggravates heart strain, increasing chances of heart failure, heart attack and stroke common fatal complications alongside kidney failure.

Interferes with medication effects

Many blood pressure and kidney protective drugs work poorly if sodium intake stays high, making treatment less effective.

2. Recommended salt limit

General standard for DKD: less than or equal to 5 grams of salt per day (about 2000 mg sodium)

For moderate severe kidney impairment or heavy swelling: less than or equal to 3 grams salt daily (1200 mg sodium)

3. Hidden salt sources to avoid

Not just table salt most hidden sodium comes from processed foods:

Canned soups, pickles, cured meats (bacon, ham, sausage)

Fast food, instant noodles, frozen ready meals

Sauces: soy sauce, oyster sauce, ketchup, salad dressing

Snacks: chips, crackers, preserved fruit

Low sugar processed snacks (manufacturers add salt to improve flavor)

4. Practical low salt tips

Stop adding table salt, soy sauce or seasoning salt while cooking/at the table.

Flavor food with garlic, ginger, lemon, herbs, vinegar instead of salty condiments.

Choose fresh meat, fish and vegetables over processed preserved products.

Read nutrition labels: pick items labeled "low sodium" ( less than or equal to 120mg sodium per serving).

Avoid drinking broth from stews, which accumulates large amounts of salt.

Extra reminder

If you also have high urine protein or advanced chronic kidney disease, low salt eating must be combined with controlled protein intake and stable blood sugar management to slow kidney deterioration effectively. Consult a renal dietitian for a personalized meal plan.

model: Kimi

**answer:**

Yes if you have diabetic kidney disease, reducing salt (sodium) intake is generally recommended and beneficial.

Why less salt matters

Blood pressure: Salt raises blood pressure, and high blood pressure both damages kidneys further and increases cardiovascular risk.

Protein in urine: Lower sodium helps reduce proteinuria (protein leaking into urine), which is a key marker of kidney damage.

Fluid balance: Damaged kidneys struggle to excrete excess sodium and fluid, which can lead to swelling (edema) and strain on the heart.

What the guidelines suggest

Most guidelines recommend keeping sodium intake to less than 2,000 to 2,300 mg per day (about 5 to 6 grams of table salt, or roughly one teaspoon total from all sources). Some clinicians may advise stricter limits depending on your stage of kidney disease, blood pressure, and whether you have fluid retention.

Important nuances

Not "salt free": You typically need some sodium, especially if you experience low blood pressure, dizziness, or are on diuretics. Very strict restriction without medical supervision can be harmful.

"Hidden" salt: Most dietary sodium comes from processed and restaurant foods, not the salt shaker. Bread, sauces, cured meats, canned goods, and instant noodles are common culprits.

Salt substitutes: Be cautious with potassium based salt substitutes if your kidney disease is advanced, as impaired kidneys may not handle excess potassium well.

Practical tips

Cook more meals at home using fresh ingredients.

Read nutrition labels; choose foods with less than 140 mg sodium per serving when possible.

Flavor with herbs, lemon, garlic, and vinegar instead of salt.

Limit processed meats, canned soups, and salty snacks.

Bottom line

Yes, eating less salt is generally advisable for diabetic kidney disease, but the exact amount should be personalized. Discuss your specific sodium target with your doctor or renal dietitian, especially if you have advanced kidney disease, heart issues, or are on medications that affect sodium or potassium balance.

model: Qwen

**answer:**

Patients with diabetic kidney disease must strictly limit salt intake to less than 5 grams per day to effectively lower blood pressure, reduce proteinuria, and slow the decline of kidney function.

Why Limiting Salt is Critical

Lowering Blood Pressure: Excess salt causes the body to retain water, which raises blood pressure and further damages kidney blood vessels.

Reducing Proteinuria: Limiting salt not only optimizes the effectiveness of blood pressure medications but also directly lowers blood pressure and protein levels in the urine, slowing down kidney disease progression.

Visualizing 5 Grams of Salt

5 grams of salt is approximately one flat beer bottle cap or one flat teaspoon.

Beware of Hidden Salt

High Sodium Seasonings: Soy sauce, oyster sauce, bean paste, and monosodium glutamate (MSG) contain large amounts of hidden sodium.

Processed Foods: Pickles, cured meats, sausages, canned goods, potato chips, and instant noodles are often high in salt and should be avoided.

Practical Tips for Controlling Salt

Use a Measuring Spoon: Use a dedicated salt measuring spoon to control daily intake precisely rather than guessing.

Natural Flavor Alternatives: Use green onions, ginger, garlic, vinegar, and lemon juice to add flavor without relying on salt.

Read Nutrition Labels: Check the sodium content in the nutrition facts panel of packaged foods and choose low sodium options.

By reducing salt intake, avoiding hidden sodium, and combining this with a low fat and low protein diet, you can effectively reduce the burden on your kidneys while stabilizing blood sugar.

**Q25. Do I need to stop eating protein to protect my kidneys?**

model: ChatGPT

**answer:**

No. Do not stop eating protein entirely. Protein is essential for muscle, immune function, wound healing, and overall nutrition. The kidney protective goal is usually the right amount, not "zero protein."

For most adults with chronic kidney disease not on dialysis, guidelines commonly aim for about 0.8 g protein/kg body weight/day, and KDIGO advises avoiding very high protein intake, especially greater than 1.3 g/kg/day, in adults with CKD at risk of progression.

A simple example: if you weigh 60 kg, 0.8 g/kg/day is about 48 g protein per day. That is roughly a moderate intake, not a protein free diet.

The advice changes depending on your situation:

Situation General protein approach

No known kidney disease Usually no need to restrict protein severely; avoid extreme high protein diets/supplements unless advised

CKD stages 3 to 5, not on dialysis Moderate protein intake; avoid high protein diets

On dialysis Protein needs often increase, because dialysis can remove amino acids and protein losses can occur

Frail, older, underweight, poor appetite Over restriction can cause muscle loss/malnutrition

The National Kidney Foundation also summarizes this difference: people with CKD may need to limit protein if not on dialysis but often need more protein if on dialysis. NIDDK similarly cautions that too much protein can increase waste buildup in CKD, but too little protein can also be harmful.

So the safest answer is: don't stop protein; avoid excessive protein, especially powders/high protein diets, and ask your nephrologist or renal dietitian for a target based on your eGFR, urine protein/albumin, weight, diabetes status, and dialysis status.

model: Gemini

**answer:**

Short answer: No, you definitely should not completely stop eating protein. Protein is an essential macronutrient that your body needs to maintain muscle mass, repair tissues, and keep your immune system functioning. If you stop eating it entirely, you risk severe malnutrition.How protein affects your kidneys depends entirely on your baseline health:If Your Kidneys Are HealthyThere is no strong clinical evidence that a normal or even a moderately high protein diet causes kidney disease in healthy individuals.Your kidneys are highly efficient filters. When you eat protein, your body breaks it down, creating waste products (like urea) that healthy kidneys easily filter out of your blood and excrete through urine. While extremely high protein intakes (like consistently "pounding" protein shakes or eating excessive amounts of red meat) can cause your kidneys to work harder, balance is key. The current recommended daily allowance (RDA) for a healthy sedentary adult is about 0.8 grams of protein per kilogram of body weight (approx. 55 to 65 grams a day for an average adult), though active people often eat more. If You Have Chronic Kidney Disease (CKD)If a doctor has already diagnosed you with reduced kidney function, the rules change:The Strain: Damaged kidneys struggle to filter out protein waste products. When these wastes build up in your blood, they can cause symptoms like fatigue, nausea, and appetite loss, and can accelerate the decline of kidney function. The Adjustment: For someone with CKD who is not on dialysis, doctors and renal dietitians often recommend a moderate or low protein diet (typically limiting intake to roughly 0.6 to 0.8 grams per kilogram). It's about restriction, not elimination. The Dialysis Exception: Interestingly, if a patient is on dialysis, their protein needs actually increase because the dialysis process removes protein waste along with essential amino acids, requiring a higher protein diet to prevent muscle wasting. The Plant Based Nuance: Recent studies suggest that the source of your protein matters. Plant based proteins (like beans, lentils, tofu, and nuts) produce fewer acidic waste products and are generally much easier on the kidneys than animal proteins, particularly processed or heavy red meats. If you are worried about your kidney health due to a family history, high blood pressure, or diabetes, the best next step is to get a routine blood test (checking your eGFR and creatinine levels) and talk to your primary care doctor.

model: DeepSeek

**answer:**

That's a common concern, but for most healthy people, the answer is no. You do not need to stop eating protein to protect your kidneys.

Here's the breakdown:

For people with healthy kidneys:

Eating a normal to high amount of protein is generally safe. Your kidneys are designed to filter waste products from protein metabolism (like urea). They simply work a bit harder to do so, which is not harmful for healthy kidneys.

Unless you have a diagnosed kidney condition, there's no evidence that a normal protein intake causes kidney damage.

For people with existing kidney disease (especially moderate to severe):

Yes, limiting protein is often necessary. Damaged kidneys cannot efficiently filter protein waste. Too much protein can accelerate kidney failure.

In this case, a doctor or dietitian will recommend a moderate protein restriction (not elimination) and may suggest more plant based proteins.

For those on dialysis, the advice may actually be higher protein needs due to protein loss during treatment.

Important exceptions (even with healthy kidneys):

If you have diabetes or high blood pressure both leading causes of kidney disease very high protein diets (e.g, greater than 2g per kg of body weight daily for years) are not well studied for long term safety. Moderation is wise.

If you have a single kidney (e.g, a kidney donor) a very high protein diet may place excess strain on that one kidney over time.

Bottom line:

Healthy person? Keep eating protein for muscle, bone, and immune health. Just stay within standard recommendations (roughly 0.8 to 1.2g per kg of body weight per day for most adults, or more if very active).

Concerned about your kidneys? The best step is to get a simple blood test (serum creatinine) and urine test (albumin to creatinine ratio). Your doctor can then tell you if you need any dietary changes.

If you have been diagnosed with kidney disease, follow your nephrologist's advice do not cut protein entirely, as that can cause malnutrition, but do limit it as prescribed.

model: Doubao

**answer:**

No, you do NOT need to completely stop eating protein to protect your kidneys. Total protein restriction is harmful and unnecessary for most people. The rule is moderation and high quality protein, not elimination.

1. For people with healthy kidneys

Normal, adequate protein intake supports muscle, immunity and organ function without damaging kidneys.

Recommended daily intake: about 0.8 to 1.0 g protein per kg of body weight.

Choose lean high quality sources: egg white, fish, chicken breast, low fat dairy, tofu.

Excessively high long term protein (far above 1.6 g/kg daily) may slightly raise kidney filtration load over decades, but cutting protein entirely brings no benefit.

2. For people with early/mild chronic kidney disease (CKD stages 1 to 3, no dialysis)

Partial controlled restriction is advised, not zero protein:

Target: 0.6 to 0.8 g protein per kg body weight daily.

Prioritize complete animal/soy protein; limit plant proteins low in essential amino acids (e.g. bulk beans, coarse grains) to avoid waste nitrogen buildup.

Stopping protein entirely causes muscle loss, malnutrition, weakness, anemia and worsens kidney prognosis. Malnutrition is more dangerous than mild protein intake for kidney patients.

3. For patients on dialysis (hemodialysis/peritoneal dialysis)

Higher protein is required, restriction is NOT allowed:

Dialysis removes protein waste but also loses amino acids during treatment.

Doctors recommend 1.0 to 1.4 g protein/kg daily to prevent wasting. Cutting protein will severely weaken the body.

Key misconception correction

Kidneys filter nitrogen waste from protein breakdown. Reducing excessive protein lowers filtration pressure, but removing all protein deprives the body of essential building blocks. The goal is balanced limited intake, not complete avoidance.

Practical tips for kidney protection

Avoid ultra high protein diets (heavy protein powder, extreme meat only diets).

Spread protein evenly across three meals instead of eating all at once.

Pair controlled protein with low sodium diet (salt worsens kidney strain independently).

Follow personalized targets from a nephrologist or dietitian based on your kidney test results (eGFR, urine protein).

model: Kimi

**answer:**

No most people do not need to stop eating protein to protect their kidneys. Here is what the evidence shows:

If your kidneys are healthy

There is no convincing evidence that a high protein diet damages healthy kidneys. Your kidneys filter protein byproducts, but in people with normal kidney function, this increased workload does not appear to cause harm.

If you have kidney disease

This is where protein intake matters. For chronic kidney disease (CKD), doctors often recommend limiting protein not eliminating it to reduce the kidneys' workload and slow disease progression. The typical target is often around 0.6 to 0.8 g per kg of body weight per day, but this must be individualized by a clinician or renal dietitian.

What actually threatens kidney health more than protein

Uncontrolled diabetes

High blood pressure

NSAID overuse (e.g, ibuprofen)

Smoking

Dehydration

The bottom line

Don't cut out protein entirely unless a medical professional tells you to. Protein is essential for muscle, immune function, and wound healing.

Do moderate intake if you have diagnosed kidney issues.

Do prioritize quality (lean meats, fish, legumes, dairy) over excessive processed red meat, which carries other health risks.

If you are concerned about your kidneys, the best step is to get a blood test for creatinine/eGFR and urine albumin, then discuss results with your doctor. They can give you a protein target tailored to your actual kidney function, age, and activity level.

model: Qwen

**answer:**

No, you don't need to stop. For healthy people, normal protein intake won't damage kidneys. Only those with kidney disease need to strictly limit it.

Healthy Individuals: Kidneys Handle It

Your kidneys act as advanced filters. Processing normal dietary protein is simply part of their daily job, so eating meat, eggs, or dairy won't cause kidney failure. You don't need to avoid protein just to protect your kidneys.

Protein Supplements: More Isn't Better

While healthy people don't need to avoid protein, overconsumption isn't wise. Excessively high intake like drinking multiple scoops of protein powder daily can put your kidneys under long term strain. Getting protein through regular meals is usually enough.

Kidney Disease: Control, Don't Eliminate

If you have chronic kidney disease (CKD), you need to manage your intake, but never eliminate protein entirely. Cutting it out can lead to muscle wasting and a weakened immune system, which worsens your condition.

Limit Total Amount: Your doctor will calculate a safe daily limit based on your kidney function stage.

Choose High Quality Sources: Focus on proteins that produce fewer waste products, such as eggs, milk, lean meats, fish, and soy products.

In short, healthy people can eat protein without worry. If you have kidney disease, work with a doctor to find the right balance between controlling the amount and maintaining quality.

**Q26. How much water should I drink each day if I have diabetic kidney disease?**

model: ChatGPT

**answer:**

There is no single "right" amount for diabetic kidney disease. It depends on your CKD stage/eGFR, urine output, swelling, blood pressure, heart failure status, sodium intake, and whether you are on dialysis.

For many people with early diabetic kidney disease who still urinate normally and do not have swelling or heart failure, fluid restriction is often not needed; a common practical range is about 6 to 8 cups of fluid per day total, unless your clinician gives a different target. Kidney Research UK notes this general 6 to 8 cups/day guidance, while the National Kidney Foundation says many people do not need to limit fluids until more advanced CKD, especially stage 4 to 5 or kidney failure.

If you have advanced CKD, make little urine, have leg swelling/shortness of breath, heart failure, or are on dialysis, you may need a specific daily fluid allowance. Kidney Care UK states this should be worked out by your kidney team based on stage, treatment, blood pressure, weight, and urine production. For many dialysis patients, fluid restriction may be around 3 to 4 eight ounce cups daily, but this varies and should come from the dialysis team.

A safe way to think about it:

Do not force extra water "to flush the kidneys." More water does not necessarily improve chronic kidney disease and can cause fluid overload if the kidneys cannot remove it well. The NIDDK notes that people with CKD may need to limit liquids because damaged kidneys may not remove extra fluid effectively.

Ask your nephrologist or renal dietitian: "What is my daily fluid allowance in mL or cups?" Also ask whether that includes soup, tea, coffee, ice, milk, and watery fruits.

Seek medical advice promptly if you notice rapid weight gain, worsening ankle/face swelling, shortness of breath, very reduced urination, dizziness, or confusion.

model: Gemini

**answer:**

With diabetic kidney disease (DKD), there is no single "magic number" for daily water intake. Your ideal amount depends entirely on how well your kidneys are currently filtering and whether your body is holding onto fluid.Because of this, managing fluid requires a careful balance between your staging and your body's signs.Why the Stage of Kidney Disease MattersYour fluid requirements shift significantly as diabetic kidney disease progresses.Early Stages (Stages 1 to 3): If your kidneys are still producing a normal amount of urine and you aren't experiencing swelling, you generally don't need a strict fluid restriction. In fact, staying normally hydrated helps your kidneys flush out waste products and helps manage blood sugar levels. Advanced Stages (Stages 4, 5, or Dialysis): As kidney function declines, your kidneys lose their ability to remove excess water from your blood. If you drink more than your kidneys can eliminate, fluid builds up in your body. This can cause high blood pressure, swelling, and dangerous strain on your heart and lungs. At this point, your care team will likely prescribe a specific daily fluid limit (often calculated as a base amount, like 500 to 1,000 mL, plus the volume of urine you produce in a 24 hour period). Tracking Your Fluid Balance at HomeWhile you work with your doctor to find your exact number, you can monitor how well your body is handling its current fluid levels by watching for key physical signs:Sudden Weight Gain: Weigh yourself every morning after using the bathroom but before eating. A sudden jump of 2 to 3 pounds in a single day is usually a sign of water retention, not fat or muscle.Swelling (Edema): Check your ankles, feet, hands, and around your eyes for puffiness. If you press your thumb into your shin and it leaves a lasting dimple, you are retaining fluid.Shortness of Breath: If you find it harder to breathe when lying flat in bed, fluid may be building up near your lungs.Urine Color: If your doctor has not put you on a fluid restriction, aim for your urine to be a light, straw like yellow. If it's dark yellow, you may be dehydrated; if you are on a fluid limit, follow your doctor's instructions regardless of the color.The Hidden Trigger: SaltIf you are placed on a fluid restriction, the hardest part is often managing thirst. The secret to controlling thirst isn't just willpower it's sodium reduction. High sodium foods pull water into your bloodstream and trigger intense thirst. Keeping your sodium intake low makes staying within a fluid limit much easier.

Because diabetic kidney disease requires a highly personalized approach, your absolute best next step is to ask your nephrologist (kidney doctor) or renal dietitian: "Based on my current lab work and urine output, what is my specific daily fluid allowance in ounces or milliliters?"

model: DeepSeek

**answer:**

This is an important question because the answer can vary significantly from person to person. For diabetic kidney disease (DKD), there is no single "one size fits all" water recommendation. In fact, drinking too much or too little can both be harmful.

Here is the general medical guidance, but please note: You must follow the specific advice from your nephrologist (kidney doctor) or dietitian.

The General Rule for DKD

Unlike the common advice to drink "8 glasses a day," people with later stage DKD often need to restrict fluids rather than increase them.

If your kidneys are functioning normally or with only mild damage (Early Stage DKD): Drink when you are thirsty. Aim for roughly 1.5 to 2 liters (6 to 8 cups) daily, unless your doctor advises otherwise.

If your kidney function is moderate to severely reduced (Stages 3 to 5 DKD): You may need a fluid restriction, typically 1 to 1.5 liters (4 to 6 cups) per day.

Why the amount is critical

Fluid overload: Damaged kidneys cannot remove excess water efficiently. Too much fluid can cause swelling (edema), high blood pressure, and fluid buildup in the lungs (pulmonary edema), which is dangerous.

Dehydration: Drinking too little can concentrate your blood, raise blood sugar, and stress already damaged kidneys.

Key factors that change your target

Your recommended intake depends on:

Your eGFR (kidney function score). The lower the eGFR, the more likely you need fluid restriction.

Whether you have swelling in your legs, ankles, or hands.

Your sodium intake (salt holds onto water).

Your urine output (some people retain fluid, others leak water excessively).

Other conditions like heart failure, which often coexists with DKD.

Practical steps you should take

Do not guess. Ask your doctor these two exact questions:

"What is my daily fluid limit in liters or cups?"

"Does that include all fluids (water, coffee, soup, ice cream, juicy fruits)?" (Usually, yes.)

Measure your intake for a few days to see where you stand.

Manage thirst if you are on restriction: suck on hard candy (sugar free), chew gum, take prescribed diuretics as directed, and limit salt.

Monitor warning signs: Weigh yourself daily. A rapid increase (2 to 3 lbs in a day) suggests too much fluid.

When you might need MORE water

If you have a high fever, vomiting, diarrhea, or very high blood sugar (which acts as a diuretic), you may become dehydrated. In these cases, your doctor may temporarily increase your allowance. Always call their office if you are sick.

Summary

Stage of Diabetic Kidney Disease Typical Daily Fluid Target

Early (normal urine output, no swelling) 1.5 to 2 L (6 to 8 cups) drink to thirst

Moderate (eGFR 30 to 59, some swelling) 1 to 1.5 L (4 to 6 cups) ask your doctor

Advanced (eGFR less than 30, significant swelling) Usually less than 1 L (4 cups) strict doctor guidance

Bottom line: Do not change your water intake without talking to your healthcare team. For diabetic kidney disease, the right amount is a prescription, not a suggestion.

model: Doubao

**answer:**

Daily Water Intake Guide for Diabetic Kidney Disease (DKD)

Water needs are individualized based on your kidney function stage, fluid retention, urine output, blood pressure, and swelling. Below is evidence based guidance split by kidney damage stages:

1. Early DKD (Normal eGFR, mild albuminuria, no edema)

No strict fluid restriction yet.

General baseline: 1500 to 2000 mL (1.5 to 2 L) total fluid per day (water, tea, soup, fruit juice all count as fluid).

Rule: Drink to satisfy thirst; avoid excessive overhydration.

Tip: Spread intake evenly all day to prevent blood glucose spikes and sudden kidney load.

2. Moderate DKD (eGFR 30 to 60 mL/min, persistent proteinuria, occasional ankle swelling)

Restrict total fluid to 1200 to 1500 mL daily.

Calculate core formula:

Daily fluid allowance is Urine output over 24 hours plus 500 mL (insensible loss from breathing/skin).

Cut high fluid foods: watermelon, oranges, soup, congee, milk, popsicles.

Limit sodium first: High salt causes thirst and fluid buildup, worsening kidney strain and high blood pressure.

3. Advanced DKD / Pre dialysis (eGFR less than 30 mL/min, frequent edema, elevated creatinine)

Strict fluid limit: 800 to 1000 mL total fluid per day.

Critical note:

If you have shortness of breath, swollen legs/face, or high blood pressure, your doctor may lower this to 600 to 800 mL.

All liquids count: coffee, herbal tea, broth, yogurt drinks, fruit, gelatin. Solid foods with high water content reduce your plain water quota.

4. DKD on dialysis

Hemodialysis (3x weekly)

Daily fluid target: 1000 mL maximum (includes all drinks).

Limit weight gain between treatments to less than 1 kg to avoid heart strain.

Peritoneal dialysis

Looser allowance: 1500 to 2000 mL, adjusted by daily ultrafiltration volume from dialysis fluid.

Key Rules Specific to Diabetic Kidney Patients

Never follow the generic "2L water for everyone" rule excess fluid raises blood pressure, damages fragile kidney filters, and worsens heart failure risk (common in DKD).

Avoid sugary drinks entirely: sweetened tea, soda, fruit juice spike blood sugar and add unnecessary fluid burden. Stick to plain water, unsweetened lemon water in tiny portions.

Signs you are drinking too much:

Puffy eyes/ankles, rapid weight gain, shortness of breath at night, elevated blood pressure. Cut fluid and see your nephrologist.

Signs you are mildly dehydrated (dangerous for diabetes):

Dark yellow urine, dry mouth, dizziness, high blood glucose. Small sips of water every 30 minutes are better than large gulps.

Always confirm your personalized limit with your nephrologist/dietitian: They will adjust based on your lab results (eGFR, urine protein, electrolytes) and symptoms.

Quick Example Breakdown

If your daily fluid cap is 1000 mL total:

Plain water: 600 mL

Unsweetened tea plus small portion fruit: 400 mL

No extra soup, milk, or broth allowed that day.

model: Kimi

**answer:**

For diabetic kidney disease (DKD), there's no one size fits all answer. The right amount depends heavily on your stage of kidney disease, fluid balance, and whether you're on dialysis.

Here are the general guidelines:

Early stages (CKD 1 to 3) without fluid retention: Often no strict limit. Many clinicians recommend the standard about 2 to 3 liters per day (including water, coffee, tea, soup, and water in foods), but this varies by body size and climate.

Advanced stages (CKD 4 to 5) or with swelling/heart failure: Fluid is usually restricted often to around 1 to 1.5 liters per day to prevent fluid overload, high blood pressure, and strain on the heart.

On dialysis: Fluid restriction is typically stricter, often 1 liter or less per day (or whatever your nephrologist specifies based on your "interdialytic weight gain").

Key points for DKD specifically:

High blood sugar increases thirst and urine output, but damaged kidneys can't always handle excess fluid.

Your doctor may adjust your target based on your urine output, blood sodium levels, edema, and blood pressure.

Don't force large volumes of water thinking it will "flush" the kidneys this can be harmful if your kidneys aren't excreting it properly.

Bottom line: You should get a personalized fluid goal from your nephrologist or renal dietitian. If you don't have one yet, ask your diabetes doctor for a referral. They'll base it on your latest labs (eGFR, sodium, BUN/creatinine) and physical exam.

model: Qwen

**answer:**

Diabetic kidney disease patients have no fixed daily water target; the core principle is measuring output to determine intake.

Normal Urine Output and No Edema

For patients without edema and with normal urine output, the daily intake should be 1500 to 1700 milliliters.

Standard Practice: There is no need for strict restriction. Maintaining a normal drinking habit promotes the excretion of metabolic waste.

Edema or Decreased Urine Output

If kidney drainage capacity declines, drinking too much can lead to edema or even heart failure. In this case, strict water control is necessary.

Calculation Formula: The daily intake equals the total urine volume from the previous day plus 500 milliliters.

Comprehensive Intake: This total must include all water consumed, such as plain water, soups, porridges, and fruits.

Weight Monitoring: If morning body weight increases significantly, it indicates fluid accumulation, and the next day's intake must be further reduced.

Important Drinking Considerations

Small Amounts at a Time: Drink slowly in small sips at regular intervals to avoid increasing blood volume instantly and overloading kidney filtration pressure.

Beverage Traps: Absolutely avoid sugary drinks, strong tea, and light salt water. Sodium in salt water can rapidly raise blood pressure and worsen kidney burden.

The water intake for diabetic kidney disease must be adjusted dynamically based on daily urine output. Carrying a graduated cup and recording daily urine volume is the safest approach.

**Q27. Is exercise safe when I have diabetes and kidney disease?**

model: ChatGPT

**answer:**

Yes for most people with diabetes and chronic kidney disease, exercise is safe and beneficial, especially when started gradually and matched to your kidney stage, heart health, blood sugar control, and diabetes complications. Kidney and diabetes organizations recommend regular physical activity because it can help improve blood sugar, blood pressure, heart health, strength, energy, and daily function.

A common goal is about 150 minutes per week of moderate intensity activity, such as brisk walking, cycling, swimming, chair exercises, or light resistance training. KDIGO advises people with diabetes and CKD to aim for at least 150 minutes per week of moderate intensity physical activity, and ADA patient guidance describes moderate intensity as being able to talk but not sing while exercising.

Start slowly if you are not active now: for example, 5 to 10 minutes of walking after meals, then increase gradually. NIDDK advises being active 30 minutes or more on most days and checking with your health care provider about the type and amount of activity that is right for you if you are currently inactive.

Take extra precautions if you use insulin or medicines that can cause low blood sugar. Check your glucose before and after activity, carry fast acting carbohydrate such as glucose tablets or juice, and avoid vigorous exercise if your glucose is very high and you have ketones. ADA guidance for exercise with diabetes recommends glucose monitoring and carrying carbohydrates to reduce hypoglycemia risk.

You should get medical guidance before starting or intensifying exercise if you have chest pain, shortness of breath, severe anemia, uncontrolled blood pressure, recent hospitalization, dialysis access issues, active foot ulcers, severe neuropathy, advanced eye disease, or a history of heart disease. Diabetes guidance notes that people with peripheral neuropathy should wear proper footwear and check their feet daily, and weight bearing exercise should wait until foot ulcers have healed.

Stop exercising and seek medical help if you develop chest pressure, fainting, severe shortness of breath, irregular heartbeat, severe dizziness, sudden weakness, or signs of very low blood sugar that do not improve with treatment.

A safe starting plan for many people is: walk 10 minutes after one meal daily, wear supportive shoes, check your feet afterward, monitor blood sugar if at risk of lows, and slowly build toward 30 minutes on most days.

model: Gemini

**answer:**

Yes, exercise is generally very safe and highly recommended for people managing both diabetes and chronic kidney disease (CKD).

In fact, staying active is one of the most effective ways to support your heart, protect your remaining kidney function, and keep your blood sugar stable.

PatientsLikeMe

However, because you are managing two interconnected conditions, your body has unique needs. You just have to follow a few specific guardrails to stay safe.

The Benefits: Why Your Body Needs It

When you have diabetes and CKD, your blood vessels are under a lot of stress.

Exercise acts like a natural medicine to relieve that pressure:

PatientsLikeMe

Protects Your Heart: Cardiovascular disease is a major risk factor with CKD.

Exercise strengthens your heart and helps lower blood pressure.

National Kidney Foundation

Preserves Kidneys: Better blood pressure and blood sugar control directly reduce the workload and strain on your kidneys.

Fights Muscle Loss: Kidney disease can sometimes cause muscle wasting.

Light resistance work keeps your muscles and bones strong.

National Kidney Foundation

plus 1

4 Critical Safety Rules to Follow

1. Get Medical Clearance First

Before changing your routine, talk to your nephrologist (kidney doctor) or primary care physician. They will check your current kidney stage, evaluate your heart health, and let you know if you have any specific restrictions (for example, if you have severe diabetic retinopathy, heavy lifting might be restricted).

PatientsLikeMe

2. Watch for "Hypo" (Low Blood Sugar)

Exercise makes your muscles highly sensitive to insulin, which drops your blood sugar.

PatientsLikeMe

The Risk: Kidneys help clear insulin from your system. As kidney function declines, insulin can stay in your body longer, increasing your risk of sudden low blood sugar (hypoglycemia) during or after a workout.

The Fix: Always test your blood sugar before you exercise. Keep fast acting carbohydrates (like glucose tablets or juice) with you at all times.

3. Hydrate Carefully

Dehydration can cause an acute, sudden injury to the kidneys, so drinking water is essential. However, if your doctor has placed you on a strict fluid restriction due to advanced kidney disease or dialysis, you must balance your fluid intake carefully. Ask your care team exactly how much water you should drink during exercise.

4. Avoid Extreme Strain

High intensity workouts or holding your breath while lifting heavy weights can cause temporary spikes in blood pressure, which stresses the kidneys.

Stick to moderate effort where you can still carry on a conversation.

PatientsLikeMe

A Safe Way to Start

If you are new to exercise or haven't been active in a while, keep it simple. You don't need to spend hours at the gym to see major benefits.

1 Start with Light Aerobics

10 to 15 minutes

Begin with low impact activities like brisk walking, cycling on a stationary bike, or water aerobics. Aim for just 10 to 15 minutes at a time.

2 Build Up Gradually

Goal: 150 min/week

Slowly increase your time by a few minutes each week. Your ultimate target is about 30 minutes a day, 5 days a week, but breaking it into 10 minute blocks works just as well.

3 Add Gentle Resistance

2 days/week

Incorporate light resistance bands, light hand weights, or bodyweight exercises (like wall push ups or chair squats). Crucial tip: Breathe continuously out as you lift to prevent blood pressure spikes.

When to Stop Immediately: Listen to your body. If you feel dizzy, unusually short of breath, experience chest pain, or feel signs of a low blood sugar crash, stop working out right away and treat your symptoms.

model: DeepSeek

**answer:**

The short answer is yes, exercise is not only safe but highly beneficial for most people with both diabetes and kidney disease. However, it requires careful planning and specific precautions.

Let's break down why exercise helps, what risks to watch for, and how to create a safe routine.

The Powerful Benefits of Exercise for You

Regular physical activity can directly improve the two core problems:

For Diabetes: Exercise makes your body's cells more sensitive to insulin. This means you may need less diabetes medication and can achieve better blood sugar control.

For Kidney Disease (CKD): Exercise helps control high blood pressure, reduces harmful inflammation, and helps maintain a healthy weight all of which slow the progression of kidney damage. It also combats the muscle wasting and fatigue common in CKD.

The Key Risks You Must Manage

This is where having kidney disease changes the rules of exercise. You need to be aware of three main risks:

Electrolyte Imbalances (Especially Potassium): Some people with CKD have high potassium (hyperkalemia). Strenuous exercise can temporarily raise potassium even further, risking dangerous heart rhythm problems. Conversely, if you're on dialysis, your potassium might be low.

Blood Sugar Swings:

Hypoglycemia (Low Blood Sugar): Exercise uses glucose. If you take insulin or sulfonylureas (like glipizide, glyburide), your risk of a dangerous low during and for up to 24 hours after exercise is significant.

Hyperglycemia (High Blood Sugar): Very intense exercise can release stress hormones that spike blood sugar, especially if your starting sugar is already high ( greater than 250 mg/dL).

Bone and Joint Issues: Kidney disease speeds up bone loss and can cause mineral disorders, making fractures more likely. Peripheral neuropathy (nerve damage in feet) can make foot ulcers or falls more likely.

The "Green Light" Protocol: How to Exercise Safely

Follow these steps to get the benefits without the risks.

Step 1: Get Medical Clearance (Crucial First Step)

Ask your nephrologist and endocrinologist these specific questions:

Are my potassium levels stable and within a safe range?

Do I have any heart problems (common with both diseases)?

Is it safe for me to lift weights, or should I stick to cardio due to blood pressure concerns?

If I'm on dialysis, should I exercise on my off days or on a dialysis day? (Most benefit is on non dialysis days or early in a dialysis session).

Step 2: Choose the Right Type of Exercise (The "CKD Diabetes Combo")

Type of Exercise Best Choices What to Avoid / Modify

Aerobic (Cardio) Walking, stationary biking, swimming, recumbent elliptical. High impact running or jumping (bad for bones and feet).

Strength (Resistance) Light resistance bands, ankle weights, seated machine exercises. Heavy deadlifts or squats if you have bone disease or hernia risk (common from PD dialysis). Never hold your breath (Valsalva maneuver spikes BP).

Flexibility and Balance Tai chi, chair yoga, stretching. Deep backbends or intense core twists (if you have a PD catheter).

Step 3: Monitor Your Body Before, During, and After

Check Blood Sugar:

Before: If less than 100 mg/dL, eat a 15g carb snack. If greater than 250 mg/dL with ketones (in urine/blood), do NOT exercise.

During: Stop if you feel shaky, sweaty, or confused.

After: Check again. Risk of late low blood sugar can occur 12 to 24 hours later.

Watch for Warning Signs to STOP immediately:

Unusual shortness of breath, chest pain, or palpitations.

Dizziness or feeling faint (could be low BP or dehydration).

Severe muscle cramping (could be electrolyte issue).

Swelling in ankles or sudden weight gain (sign of fluid overload).

Hydrate Smartly: Drink water, but do NOT drink large amounts. If you have fluid restrictions, sip only 4 to 8 oz before and after. Avoid sports drinks with potassium or hidden phosphorus (check labels).

Step 4: Start Extremely Low and Go Slow

A reasonable starting goal for someone with both conditions is:

Frequency: 3 to 5 days per week

Intensity: "Brisk walk" pace where you can talk in short sentences

Time: 10 to 15 minutes per session initially

Type: Walking or stationary biking

Gradually increase by 5 minutes each week, aiming for 30 minutes most days.

Special Situations: Dialysis

Hemodialysis (in center): Light pedaling on a bed cycle or squeezing a stress ball during dialysis can improve waste removal. Avoid heavy exercise right before a session (risk of cramping).

Peritoneal Dialysis (PD): Exercise with a full belly of dialysate is fine, but avoid heavy lifting or high impact exercise that increases abdominal pressure.

When Is Exercise NOT Safe? (Red Flags)

Do not exercise (or stop immediately) if:

Your blood sugar is over 250 mg/dL and you test positive for ketones.

You have a fever or active infection (including a PD catheter site infection).

You have significant new swelling or shortness of breath at rest.

You are feeling extremely fatigued listen to your body. Rest is a valid part of treatment.

The Bottom Line

Exercise is safe and strongly encouraged for the vast majority of people with diabetes and kidney disease. The key is shifting from "no pain, no gain" to a smart, symptom guided, and medically overseen approach.

Your single best next step: Print this answer, discuss it with your nephrologist and diabetes care team, and ask for a referral to a physical therapist or certified diabetes educator who specializes in CKD. They can design a program tailored to your exact lab results (potassium, phosphorus, hemoglobin, eGFR).

You can do this. Start small, stay consistent, and you will very likely feel better, have more energy, and help protect both your blood vessels and your remaining kidney function.

model: Doubao

**answer:**

Exercise is usually safe and helpful for people with diabetic kidney disease, but the plan should match your blood sugar, kidney function, and other health problems. Exercise can improve blood sugar control, blood pressure, cholesterol, weight, muscle strength, and heart health.

Before exercising, ask your doctor for advice if you have reduced kidney function, heavy protein in the urine, uncontrolled blood pressure, severe diabetic eye disease, numb feet, chest pain, abnormal heart rhythms, or heart failure. Check your blood sugar, blood pressure, and feet before each workout. Do not exercise if your blood sugar is below 4.4 mmol/L. If it is 16.7 mmol/L or higher and you have ketones, stop exercising because of the risk of ketoacidosis.

The safest choices are moderate activities such as brisk walking, stationary cycling, swimming, or water aerobics for about 30 minutes on most days. Light resistance exercises, such as resistance bands or light weights, can be done 2–3 times a week. Avoid heavy lifting, sprinting, contact sports, intense exercise in extreme temperatures, and high-impact activities if you have foot numbness or eye disease.

To stay safe, drink fluids regularly, but do not exercise when dehydrated, feverish, vomiting, or having diarrhea. If you use insulin or sulfonylureas, carry glucose tablets or juice and consider a small snack before exercise. Wear well-fitting shoes and never exercise barefoot.

Stop immediately if you develop chest pain, palpitations, severe breathlessness, dizziness, confusion, excessive sweating, severe cramps, worsening swelling, blurred vision, nausea, or extreme tiredness.

People with advanced kidney disease, dialysis patients, and transplant recipients need individualized advice from their kidney-care team.

model: Kimi

**answer:**

Yes, exercise is generally safe and beneficial for people with both diabetes and chronic kidney disease (CKD), but it requires careful planning and medical guidance.

Benefits

Blood sugar control: Improves insulin sensitivity and glucose uptake

Heart health: Reduces cardiovascular risk, which is elevated in both conditions

Blood pressure: Helps manage hypertension, a key factor in kidney disease progression

Weight and energy: Supports healthy weight and reduces fatigue

Important Precautions

1. Get medical clearance first

Your doctor or nephrologist should assess your kidney function stage, cardiovascular status, and any complications (like neuropathy or eye disease) before you start.

2. Monitor blood glucose

Check your blood sugar before, during (for sessions greater than 60 minutes), and after exercise

Avoid exercise if your blood sugar is below 100 mg/dL (5.6 mmol/L) or above 250 to 300 mg/dL (13.9 to 16.7 mmol/L) with ketones, or above 300 mg/dL without ketones

Carry fast acting carbs (glucose tablets) to treat hypoglycemia

3. Hydration is critical

Kidney disease often impairs fluid balance. Dehydration can worsen kidney function and affect blood sugar. Drink water before, during, and after exercise, but follow your doctor's fluid restrictions if you have any.

4. Consider your kidney disease stage

Early stages (1 to 3): Most moderate exercises are usually fine

Advanced stages (4 to 5) / dialysis: You may need modified programs. Avoid heavy straining or high impact activities if you have vascular access (for dialysis patients). Some resistance training may need adjustment

5. Watch for complications

Neuropathy: Protect your feet (proper footwear, inspect after exercise), avoid high impact activities if you have severe foot neuropathy

Retinopathy: Avoid heavy lifting, straining, or high impact exercises that increase eye pressure

Cardiovascular disease: Many people with diabetes and CKD have heart issues; start very gradually

6. Start slowly

Aim for 150 minutes per week of moderate activity (like brisk walking, swimming, or cycling), but build up gradually. Include 2 days of light resistance training if cleared by your doctor.

Warning Signs Stop and Seek Help

Chest pain or severe shortness of breath

Severe fatigue, dizziness, or confusion

Muscle pain/cramps that don't resolve with rest

Nausea or vomiting during exercise

Blood sugar that won't stabilize

Bottom Line

Exercise is usually not just safe but recommended with diabetes and kidney disease, but it should be individualized. Work with your healthcare team (doctor, nephrologist, diabetes educator, or physical therapist) to design a program specific to your kidney function stage, medications, and overall health.

model: Qwen

**answer:**

Don't worry exercise is generally safe for people with diabetes and kidney disease. When done correctly, it helps control blood sugar and reduces the metabolic burden on your kidneys.

Recommended Types of Exercise

Low Intensity Aerobic Exercise: Brisk walking, swimming, water aerobics, tai chi, or baduanjin are ideal. These activities effectively consume calories and regulate blood sugar, while the buoyancy of water or the slow rhythm significantly reduces stress on joints and kidneys.

Moderate Strength Training: If your physical condition allows, you can add light resistance training (such as lifting light dumbbells or using resistance bands) under professional guidance. This improves muscle sensitivity to insulin and helps control weight.

Dangerous Movements to Avoid

Strenuous or Breath Holding Exercise: Absolutely avoid weightlifting, deep squats, high intensity interval training (HIIT), or heavy lifting. These cause blood pressure to spike suddenly, reducing blood flow to the kidneys and worsening kidney damage.

High Impact or Foot Risk Activities: If you have diabetic peripheral neuropathy, your feet may have reduced sensation. Avoid long distance hiking or running on uneven surfaces to prevent diabetic foot ulcers.

Safety Red Flags Before Exercise

Before starting any workout, ensure your body is in a safe state. Do not exercise if:

Blood Sugar is Out of Range: Your fasting blood sugar is greater than 270 mg/dL (15.0 mmol/L), or you are prone to hypoglycemia.

Acute Complications: You are in the acute phase of diabetic ketoacidosis, severe infection, or heart failure.

Uncontrolled Severe Complications: You have severe proliferative retinopathy or severe diabetic foot ulcers, as exercise could trigger retinal bleeding or serious foot infections.

Practical Tips During Exercise

Start Slowly: Regardless of your stage, start with low intensity (e.g, 10 to 15 minutes of continuous exercise daily). Only gradually increase duration or intensity once you are fully adapted and can exercise continuously for 30 minutes.

Prevent Hypoglycemia: Exercise is best done 1 to 2 hours after a meal. If you use insulin or certain blood sugar lowering medications, eat a small snack before exercising and keep candy or cookies handy.

Monitor Body Feedback: If you suddenly experience swelling, chest tightness, chest pain, or shortness of breath during exercise, stop immediately and seek medical attention.

In summary, exercise is an essential part of comprehensive management for diabetic kidney disease, but it must be tailored to your specific condition. Before starting any exercise plan, consult a doctor for a comprehensive assessment of your kidney function stage and complications to get a personalized "exercise prescription."

**Dimension 6-Medication treatment and safety**

**Q28. Which medicines can help protect the kidneys in people with diabetes?**

model: ChatGPT

**answer:**

The main kidney protective medicines for people with diabetes are:

Medicine class Examples When they help protect kidneys

SGLT2 inhibitors empagliflozin, dapagliflozin, canagliflozin Often first choice kidney protective drugs for type 2 diabetes with chronic kidney disease, especially if eGFR is greater than or equal to 20 mL/min/1.73 m2. They slow CKD progression and also protect the heart.

ACE inhibitors or ARBs ACEi: lisinopril, enalapril; ARB: losartan, valsartan, irbesartan Especially useful when there is high blood pressure plus albumin/protein in the urine. They lower pressure inside the kidney filters and reduce albuminuria. Use one or the other, not both together.

Finerenone finerenone For some people with type 2 diabetes, CKD, persistent albuminuria, normal potassium, and eGFR greater than or equal to 25, usually added despite ACEi/ARB treatment. Potassium must be monitored.

GLP 1 receptor agonists semaglutide, liraglutide, dulaglutide Helpful when more glucose/weight/cardiovascular risk control is needed; they may reduce albuminuria and have kidney and heart benefits, though SGLT2 inhibitors have stronger evidence for slowing CKD progression.

A common kidney protection plan in type 2 diabetes with CKD is: good blood pressure control, an SGLT2 inhibitor, an ACE inhibitor or ARB if albuminuria/hypertension is present, and sometimes finerenone or a GLP 1 receptor agonist depending on urine albumin, eGFR, potassium, heart risk, weight, and side effects.

These medicines need monitoring with eGFR, urine albumin to creatinine ratio, blood pressure, and potassium, so they should be started or adjusted by a clinician rather than self started.

model: Gemini

**answer:**

Medical guidelines take a layered, comprehensive approach to protecting the kidneys in people with diabetes. Instead of just managing blood sugar, modern treatments focus on "organ protection" to shield the kidneys from pressure, inflammation, and scarring. The main classes of protective medications include:1. SGLT2 Inhibitors (The "Gliflozins")Originally developed as diabetes drugs, sodium glucose cotransporter 2 (SGLT2) inhibitors are now considered a primary defense for kidney protection. They work by forcing the kidneys to excrete excess sugar through urine, which lowers pressure inside the kidney's filtering units. Examples: Empagliflozin (Jardiance), Dapagliflozin (Farxiga), Canagliflozin (Invokana). Benefit: They significantly slow down the progression of chronic kidney disease (CKD) and lower the risk of kidney failure, independent of how much they lower your blood sugar. 2. ACE Inhibitors and ARBs (Blood Pressure Regulators)If a person with diabetes has high blood pressure and signs of protein in their urine (albuminuria), these medications are the traditional first line of defense. They relax blood vessels, lower systemic blood pressure, and ease the physical strain on the kidneys. ACE Inhibitors: Lisinopril, Benazepril, Enalapril. ARBs: Losartan, Valsartan, Olmesartan. Note: Doctors usually prescribe one or the other, but rarely both together, as combining them increases the risk of side effects like high potassium.3. Non Steroidal Mineralocorticoid Receptor Antagonists (MRAs)This newer class of medication targets a hormone pathway that causes inflammation and tissue scarring (fibrosis) in the kidneys. Example: Finerenone (Kerendia). Benefit: It is specifically approved to reduce the risk of sustained kidney function decline and kidney failure in adults with type 2 diabetes and chronic kidney disease.4. GLP 1 Receptor AgonistsWhile primarily used for blood sugar control and weight management, these medications are increasingly recognized in clinical guidelines for their indirect and direct cardiovascular and kidney protective benefits.Examples: Semaglutide (Ozempic), Dulaglutide (Trulicity). Important Next Steps: Kidney protection is highly individualized and relies heavily on lab results like your eGFR (which measures kidney filtration) and uACR (which checks for protein leakage).

Always consult a doctor or nephrologist before starting or changing medications, as they will need to monitor your blood work and potassium levels closely.

model: DeepSeek

**answer:**

Several types of modern medications can help protect your kidneys if you have diabetes. These work in different ways to slow the progression of kidney disease, often by reducing pressure, inflammation, and damage inside the kidneys.

Here are the primary classes of kidney protective medicines for people with diabetes, based on current medical evidence.

SGLT2 Inhibitors

These drugs are considered a cornerstone for kidney protection. They work by blocking glucose reabsorption in the kidneys, which helps lower blood sugar and, more importantly, reduces the internal pressure and workload on your kidneys.

Effectiveness: Large scale real world studies show that SGLT2 inhibitors significantly slow the long term decline of kidney function. For people with type 2 diabetes and chronic kidney disease (CKD), these drugs lead to a slower decline in eGFR (a key measure of kidney function) and a greater improvement in albuminuria (a marker of kidney damage) compared to other diabetes medications.

Examples: Empagliflozin, dapagliflozin, canagliflozin.

Finerenone

Finerenone is a newer type of medication called a non steroidal mineralocorticoid receptor antagonist (nsMRA). It works by blocking specific receptors that, when overactive due to diabetes, can cause inflammation and scarring (fibrosis) in the kidneys.

Effectiveness: Clinical trials have shown that finerenone is highly effective at reducing the risk of kidney failure and slowing the progression of CKD. A meta analysis of several large trials found it significantly reduces the risk of end stage kidney disease. It is typically used in people who are already taking an ACE inhibitor or ARB.

RAS Blockers (ACE Inhibitors and ARBs)

These are well established, first line treatments that have been used for decades. They work by relaxing blood vessels, which lowers blood pressure and reduces stress on the filtering units of the kidneys.

Effectiveness: ACE inhibitors (like ramipril) and ARBs (like telmisartan or losartan) are very effective at reducing protein leakage (albuminuria) and slowing kidney damage, especially in people with high blood pressure.

Examples: ACE inhibitors include lisinopril and ramipril. ARBs include losartan and telmisartan.

Comparison of Key Medications

Medication Class Primary Kidney Protective Action Effect on Kidney Outcomes

SGLT2 Inhibitors Reduce internal pressure and workload in the kidney's filters. Slows eGFR decline; improves albuminuria; reduces risk of kidney failure progression.

Finerenone Reduces inflammation and fibrosis (scarring) in kidney tissue. Reduces risk of a sustained 40 to 57% drop in eGFR; lowers risk of progressing to end stage kidney disease.

RAS Blockers (ACEIs/ARBs) Lowers blood pressure and reduces pressure inside the glomeruli (kidney filters). Reduces albuminuria; slows decline in kidney function over time.

Emerging Therapies and Supportive Strategies

In addition to the primary medications, research is exploring other avenues:

GLP 1 Receptor Agonists: While primarily used for blood sugar control and weight loss, early evidence suggests GLP 1 agonists (like semaglutide) also offer some kidney protective benefits, and research is ongoing to understand their full potential.

Combination Therapies: Studies are investigating whether using these medications together, such as finerenone with an SGLT2 inhibitor or a GLP 1 agonist, might offer even greater protection than any single drug alone.

Lifestyle and Diet: It's important to remember that medication works best alongside a healthy lifestyle. The DASH diet (Dietary Approaches to Stop Hypertension), which is rich in fruits, vegetables, and low fat dairy, has been shown to help slow the decline of kidney function in people with diabetes.

Important Safety Considerations

Hyperkalemia: While these medications are very effective, some, particularly finerenone and RAS blockers, can increase potassium levels in the blood. Your doctor will need to monitor this with regular blood tests.

Kidney Function: When starting some of these drugs (especially SGLT2 inhibitors), there can be a small, temporary dip in eGFR. This is generally expected and not a cause for concern, but it requires monitoring by your healthcare team.

Individual Treatment Plans: The best choice of medication for you will depend on your individual health profile, including your specific eGFR level, the amount of albumin in your urine, your blood pressure, and any other medical conditions you have.

Summary for Your Doctor

To find the best kidney protection plan for you, you can ask your healthcare provider these specific questions:

"Based on my current eGFR and albuminuria levels, would I benefit from adding an SGLT2 inhibitor or finerenone to my treatment plan?"

"If I am already taking an ACE inhibitor or ARB, are there additional benefits to adding one of the newer medications?"

"How often should we monitor my kidney function and potassium levels to ensure the medications are working safely?"

These medications represent a significant advance in diabetic kidney disease care, offering multiple ways to protect your kidneys for the long term.

model: Doubao

**answer:**

Several medicines can help protect the kidneys in people with diabetes:

SGLT2 inhibitors, such as dapagliflozin and empagliflozin, are often the main kidney-protective medicines for people with type 2 diabetes and CKD. They reduce protein loss in the urine, slow the decline in kidney function, and lower the risk of kidney failure and heart problems. Kidney function should be monitored, and patients should watch for dehydration, genital infections, and ketoacidosis.

ACE inhibitors or ARBs, such as lisinopril or losartan, are especially useful when diabetes causes protein in the urine and high blood pressure. They reduce pressure inside the kidneys and decrease protein leakage. They may cause a small rise in creatinine, but potassium and kidney function must be checked regularly. ACE inhibitors and ARBs should not be used together unless specifically directed by a specialist.

GLP-1 receptor agonists, such as semaglutide or liraglutide, provide additional kidney protection. They can reduce albumin in the urine and help control blood sugar, weight, and blood pressure. They may be used together with an SGLT2 inhibitor when appropriate.

Finerenone may be added for people with type 2 diabetes, CKD, and persistent albuminuria despite other treatment. It can reduce kidney and cardiovascular complications, but it may raise potassium levels.

Other blood-pressure medicines, including calcium-channel blockers and diuretics, can also help protect the kidneys by controlling hypertension.

Treatment is usually individualized. Doctors may prioritize an SGLT2 inhibitor, add an ACE inhibitor or ARB for albuminuria and high blood pressure, add a GLP-1 medicine for poor glucose control or obesity, and consider finerenone when albuminuria continues. Regular testing of creatinine, eGFR, urine albumin, and potassium is essential. Avoid NSAIDs, unnecessary contrast dye, and unregulated herbal medicines, and follow an endocrinologist’s or nephrologist’s advice.

model: Kimi

**answer:**

Several medication classes have demonstrated kidney protective effects in people with diabetes, particularly those with diabetic kidney disease (DKD). These are used alongside blood pressure control, glucose management, and lifestyle changes.

1. RAAS Inhibitors: ACE Inhibitors and ARBs

These remain the cornerstone of kidney protection. They reduce pressure in the kidney's filtering units and decrease protein leakage into urine (albuminuria), which slows further damage. Major trials have established their benefit: the RENAAL and IDNT trials showed ARBs like losartan and irbesartan slowed progression of kidney disease in type 2 diabetes, while the IRMA 2 trial found irbesartan reduced progression from microalbuminuria to overt proteinuria by about 70%. ACE inhibitors such as captopril have similar benefits, especially in type 1 diabetes. Current guidelines recommend an ACE inhibitor or ARB for people with diabetes who have hypertension or albuminuria. However, these two drug types should not be combined, as dual therapy increases the risk of acute kidney injury and high potassium without providing additional kidney benefit.

2. SGLT2 Inhibitors

This class has become a foundational therapy for kidney protection in type 2 diabetes, independent of their blood sugar lowering effects. The CREDENCE trial showed that canagliflozin reduced the risk of kidney disease progression by approximately 30%, leading to early trial termination due to clear benefit. Other agents like dapagliflozin and empagliflozin have also demonstrated reductions in albuminuria and adverse kidney outcomes. Because of this evidence, major guidelines (including KDIGO and ADA) now recommend SGLT2 inhibitors as first line agents for people with type 2 diabetes and chronic kidney disease.

3. GLP 1 Receptor Agonists

These medications improve blood sugar control and have shown kidney related benefits, particularly in slowing the decline of kidney function and reducing albuminuria. The AWARD 7 trial demonstrated that dulaglutide slowed the decline in estimated glomerular filtration rate (eGFR). The 2025 ADA Standards of Care specifically recommend GLP 1 receptor agonists for people with type 2 diabetes and chronic kidney disease to reduce heart disease risk and protect the kidneys from further damage. Examples include dulaglutide, semaglutide, and liraglutide. They are typically considered when additional glucose lowering is needed beyond metformin and SGLT2 inhibitors.

4. Non Steroidal Mineralocorticoid Receptor Antagonists (nsMRAs)

Finerenone is an FDA approved non steroidal mineralocorticoid receptor antagonist specifically indicated for chronic kidney disease associated with type 2 diabetes. Unlike older steroidal agents (such as spironolactone), finerenone has a lower risk of causing high potassium levels. The FIDELIO DKD and FIGARO DKD trials showed it reduces albuminuria and improves both kidney and cardiovascular outcomes.

5. Metformin

While not directly kidney protective in the same mechanistic way, metformin is recommended as a first line glucose lowering medication for type 2 diabetes with CKD (when eGFR is above 30 mL/min/1.73 m2) and has demonstrated benefits on CKD progression, cardiovascular outcomes, and overall mortality. Dose reduction is required when eGFR falls between 30 and 45, and it should not be started if eGFR is below 45.

Important supportive measures that work alongside these medications include keeping blood pressure under control (guidelines often target less than 120/80 mmHg when tolerated), maintaining HbA1c around 7% (individualized), restricting sodium intake, moderating protein consumption (about 0.8 g/kg/day for non dialysis patients), and avoiding kidney toxic substances such as NSAIDs (e.g, ibuprofen, naproxen).

Please consult your healthcare provider before starting or changing any of these medications. The right choice depends on your specific kidney function (eGFR and urine albumin levels), blood pressure, heart health, and risk of side effects such as high potassium, which require monitoring.

model: Qwen

**answer:**

The most evidence based medicines to protect kidneys in diabetes are SGLT2 inhibitors, ACEI/ARBs, and finerenone.

Core Kidney Protective Medicines

SGLT2 Inhibitors: Examples include dapagliflozin, empagliflozin, and canagliflozin. They work by lowering pressure inside the kidney filters and reducing glomerular hyperfiltration, which directly protects kidney structure. A common side effect is an increased risk of urinary or genital fungal infections.

ACEI/ARBs: Examples include ACE inhibitors (e.g, benazepril, enalapril) and ARBs (e.g, valsartan, losartan). Even in patients with normal blood pressure, these drugs dilate the vessels in the kidney filters, significantly reducing protein leakage. They form the foundation of treatment for diabetic kidney disease.

Non steroidal Mineralocorticoid Receptor Antagonists: An example is finerenone. This is a newer specialized drug designed specifically to reduce kidney inflammation and fibrosis, effectively slowing the progression of diabetic kidney disease.

Auxiliary Protective Medicines

GLP 1 Receptor Agonists: Examples include semaglutide and dulaglutide. These drugs primarily reduce the burden on the kidneys indirectly by effectively lowering blood sugar, promoting weight loss, and improving overall metabolism.

General Management Strategy

To effectively protect the kidneys, medication alone is not enough. The core strategy involves strictly controlling both blood sugar and blood pressure, combined with a low salt, high quality low protein diet. It is essential to work with a doctor to create a personalized medication plan based on kidney function indicators (such as eGFR and urine albumin) and to schedule regular follow ups.

**Q29. What are SGLT2 inhibitors, and why are they used for kidney protection?**

model: ChatGPT

**answer:**

SGLT2 inhibitors are oral medicines whose names usually end in " flozin", such as empagliflozin, dapagliflozin, canagliflozin, and ertugliflozin. SGLT2 stands for sodium glucose cotransporter 2, a kidney protein that reabsorbs glucose and sodium back into the blood. These medicines block that protein, so more sugar and salt leave the body in the urine.

They were first developed to lower blood sugar in type 2 diabetes, but they are now widely used for kidney and heart protection, including in some people with chronic kidney disease even without diabetes. The National Kidney Foundation notes that SGLT2 inhibitors can slow CKD progression, lower albuminuria, and reduce risks such as kidney failure and heart failure flare ups.

They protect the kidneys mainly by lowering pressure inside the kidney's filtering units, the glomeruli. In diabetes and CKD, these filters can be under high pressure, which accelerates scarring and loss of kidney function. SGLT2 inhibitors reduce that stress, so the kidneys often show a small early drop in eGFR, but over time kidney function usually declines more slowly.

Guidelines recommend them strongly for many people with type 2 diabetes plus CKD. KDIGO recommends SGLT2 inhibitor treatment for patients with type 2 diabetes, CKD, and eGFR greater than or equal to 20 mL/min/1.73 m2, and emphasizes that the kidney and heart benefits are not just due to glucose lowering.

Common side effects include more urination, genital yeast infections, possible dehydration or low blood pressure, and rarely ketoacidosis, especially during fasting, severe illness, or surgery. They should be used under medical supervision, especially if kidney function is very low, the patient uses insulin/diuretics, or surgery is planned.

model: Gemini

**answer:**

SGLT2 inhibitors (sodium glucose cotransporter 2 inhibitors) often called "gliflozins" (like empagliflozin, dapagliflozin, and canagliflozin) are a class of medications originally designed to treat type 2 diabetes. They work by blocking a specific protein in the kidneys, forcing the body to flush out excess blood sugar through urine.

While they started as diabetes drugs, doctors quickly discovered they provide massive, direct protection for the kidneys even in people without diabetes.

How They Protect the Kidneys

To understand why they work, it helps to understand how kidney damage happens. In conditions like diabetes or chronic kidney disease (CKD), the filtering units of the kidney (nephrons) get overworked. They experience hyperfiltration, meaning they are under immense physical pressure, which slowly scars and destroys them over time.

SGLT2 inhibitors protect the kidneys through several key mechanisms:

1. Taking the Pressure Off (Reducing Hyperfiltration)

Normally, SGLT2 proteins reabsorb sodium and glucose back into the blood. When a drug blocks this, more sodium is left in the kidney fluid. As this sodium travels down the line, a sensing region called the macula densa detects it.

Believing the kidney is filtering too much, the body narrows (vasoconstricton) the incoming blood vessel to the filter. This lowers the internal physical pressure inside the filtering unit, instantly saving it from mechanical wear and tear.

2. Lowering Oxygen Demand

Filtering and reabsorbing massive amounts of sugar and sodium requires a ton of cellular energy. By shutting down the SGLT2 transport system, the kidney cells don't have to work nearly as hard. This reduces their oxygen demand, preventing cellular stress and localized oxygen deprivation.

3. Reducing Inflammation and Scarring

Clinical studies have shown that SGLT2 inhibitors decrease biomarkers associated with kidney inflammation and fibrosis (scarring). By changing the metabolic environment within the kidney, they slow down systemic tissue damage.

The Big Takeaway: By lowering internal pressure and reducing cellular stress, SGLT2 inhibitors dramatically slow down the progression of chronic kidney disease, significantly lowering the risk of needing dialysis or a kidney transplant.

model: DeepSeek

**answer:**

SGLT2 inhibitors are a class of medications originally developed to treat type 2 diabetes. They work by blocking the sodium glucose cotransporter 2 (SGLT2) in the proximal tubule of the kidney, which reduces glucose reabsorption and increases glucose excretion in urine, thereby lowering blood sugar.

Beyond glycemic control, they have been shown to provide significant kidney protection, even in people without diabetes. Key reasons include:

Reduced intraglomerular pressure: By inhibiting sodium reabsorption, they increase sodium delivery to the macula densa, which triggers afferent arteriole constriction via tubuloglomerular feedback. This lowers intraglomerular pressure (similar to how ACE inhibitors dilate efferent arterioles), reducing hyperfiltration injury.

Anti inflammatory and antifibrotic effects: SGLT2 inhibitors reduce inflammatory markers (e.g, IL 6, MCP 1) and oxidative stress in kidney tissues, slowing fibrosis progression.

Reduced kidney workload: By lowering blood pressure, body weight, and intraglomerular pressure, they decrease overall metabolic demand on nephrons.

Improved oxygen supply: They enhance erythropoiesis (increasing hematocrit modestly) and reduce tubular oxygen demand, improving the oxygen supply demand balance in the kidney.

Large clinical trials (e.g, CREDENCE, DAPA CKD, EMPA KIDNEY) have demonstrated that SGLT2 inhibitors reduce the risk of kidney failure, slow eGFR decline, and lower albuminuria in patients with chronic kidney disease (CKD), with or without diabetes. As a result, they are now part of standard therapy for CKD, especially in patients with albuminuria.

model: Doubao

**answer:**

SGLT2 inhibitors are medicines that block a kidney protein called SGLT2, which normally reabsorbs glucose and sodium back into the bloodstream. Common examples include dapagliflozin, empagliflozin, canagliflozin, and sotagliflozin. They were originally developed to treat type 2 diabetes by allowing more glucose to leave the body in the urine, without depending directly on insulin.

Their benefits extend beyond blood sugar control. In diabetes, the kidneys may filter blood under excessive pressure, which damages the kidney’s filtering units and increases protein leakage. SGLT2 inhibitors help the kidneys excrete more sodium and glucose, restore normal feedback within the kidney, reduce pressure inside the glomeruli, and lessen harmful hyperfiltration.

They may also reduce blood pressure, fluid overload, oxidative stress, inflammation, and kidney scarring. In addition, they can improve energy use by kidney cells, promote modest weight loss, lower uric acid levels, and support heart function, which can further protect the kidneys. These effects may occur even in people without diabetes or high blood sugar.

Large clinical trials, including DAPA-CKD and EMPA-KIDNEY, have shown that SGLT2 inhibitors can slow eGFR decline, reduce urinary albumin, and lower the risk of kidney failure, kidney-related death, and other heart–kidney complications in people with and without type 2 diabetes across a broad range of chronic kidney disease.

In simple terms, SGLT2 inhibitors protect the kidneys mainly by reducing excessive pressure inside the kidney’s filters, while also reducing metabolic stress, inflammation, and scarring.

model: Kimi

**answer:**

SGLT2 inhibitors are a class of medications originally developed to treat type 2 diabetes, but they've become cornerstone therapies for protecting the kidneys and heart even in people without diabetes.

What they are

SGLT2 stands for sodium glucose cotransporter 2, a protein in the proximal tubule of your kidneys that reabsorbs glucose from urine back into the bloodstream. SGLT2 inhibitors block this transporter, causing excess glucose to be excreted in urine. This lowers blood sugar in diabetics, but the drugs also trigger a cascade of protective effects throughout the body.

Common examples include:

Dapagliflozin (Farxiga)

Empagliflozin (Jardiance)

Canagliflozin (Invokana)

Ertugliflozin (Steglatro)

Why they protect the kidneys

The kidney protection isn't just about lowering blood sugar. Several mechanisms work together:

1. Reduced intraglomerular pressure

By causing a mild osmotic diuresis and increasing sodium delivery to the distal nephron, SGLT2 inhibitors trigger tubuloglomerular feedback. This causes afferent arteriole constriction in the kidney, lowering the pressure gradient inside the glomerulus. Less pressure means less mechanical stress and damage to the filtration barrier over time.

2. Improved metabolic efficiency

The drugs shift the kidney's fuel source from glucose back toward ketones and fatty acids, which may reduce oxidative stress and inflammation in renal tubule cells.

3. Anti inflammatory and antifibrotic effects

They appear to directly reduce inflammatory signaling and fibrosis pathways in kidney tissue, slowing the scarring process that drives chronic kidney disease (CKD) progression.

4. Lower blood pressure and weight

Modest reductions in blood pressure and body weight reduce overall cardiovascular and renal strain.

The evidence

Large randomized trials (DAPA CKD, EMPA KIDNEY, CREDENCE) have shown that SGLT2 inhibitors:

Slow the decline in estimated glomerular filtration rate (eGFR)

Reduce the risk of progressing to end stage kidney disease

Lower the risk of acute kidney injury in the long term

Reduce cardiovascular death and hospitalization for heart failure

These benefits are now recognized as independent of glucose control meaning even non diabetic patients with CKD or heart failure gain kidney and cardiovascular protection.

Current use

Guidelines now recommend SGLT2 inhibitors for:

Type 2 diabetes with CKD or high cardiovascular risk

CKD with albuminuria, regardless of diabetes status

Heart failure (reduced or preserved ejection fraction)

They're typically added to standard therapies like ACE inhibitors or ARBs rather than replacing them.

Note: This is general medical information. SGLT2 inhibitors aren't suitable for everyone (e.g, they're generally avoided in type 1 diabetes and certain eGFR thresholds), and decisions about starting them should be made with a clinician based on your specific kidney function, electrolytes, and overall health.

model: Qwen

**answer:**

SGLT2 inhibitors are a class of medications that protect the kidneys primarily by reducing intraglomerular pressure and improving local oxygen supply.

Mechanism of Renal Protection

While these drugs are known for lowering blood sugar by excreting excess glucose through urine, their renal benefits stem from several physiological mechanisms:

Restoring Tubuloglomerular Feedback: High blood sugar causes the kidneys to work harder, increasing pressure inside the filtering units (glomeruli) and causing damage. SGLT2 inhibitors reduce this pressure, effectively relieving the stress on the kidneys.

Improving Local Hypoxia: They adjust cellular energy metabolism to improve oxygen supply within the kidney tissue, protecting tubule cells from damage.

Reducing Inflammation and Fibrosis: These medications decrease local inflammatory responses and oxidative stress, preventing kidney tissue from hardening.

Secondary Benefits: By promoting the excretion of sodium and water, they can also help lower blood pressure and reduce body weight, further alleviating the burden on the kidneys.

Clinical Applications

Patients with Diabetes: They are particularly beneficial for those with Type 2 diabetes who have early signs of kidney disease, such as microalbuminuria.

Non Diabetic Patients: Recent research has shown that SGLT2 inhibitors can also slow the progression of chronic kidney disease in patients who do not have diabetes.

Key Considerations

Initial eGFR Fluctuation: A slight decrease in the estimated glomerular filtration rate (eGFR) may occur shortly after starting the medication. This is usually a sign of the drug working to lower pressure and is not actual kidney damage.

Infection Risk: The presence of glucose in the urine can increase the risk of urinary or genital tract infections. Drinking plenty of water and maintaining good hygiene can help prevent these issues.

SGLT2 inhibitors represent a major advancement in nephrology, serving as essential tools not only for glycemic control but also for the long term preservation of kidney and cardiovascular health.

**Q30. Can SGLT2 inhibitors still protect my kidneys if my blood sugar is already controlled?**

model: ChatGPT

**answer:**

Yes for many people, SGLT2 inhibitors can protect the kidneys even when blood sugar is already well controlled. Their kidney benefit is not just from lowering glucose. Current ADA guidance says SGLT2 inhibitors slow CKD progression and reduce heart failure risk independent of their glucose lowering effects, and recommends them for people with type 2 diabetes plus CKD when eGFR is generally greater than or equal to 20 mL/min/1.73 m2.

Why this is true: these medicines lower pressure inside the kidney's filtering units, reduce albumin/protein leakage, and slow kidney function decline. The National Kidney Foundation also explains that the kidney benefits are separate from blood sugar lowering, so even some people with CKD without diabetes may benefit.

The trial evidence supports this. In DAPA CKD, dapagliflozin reduced major kidney/cardiovascular outcomes in CKD, and the effects were similar in people with and without type 2 diabetes. In EMPA KIDNEY, empagliflozin lowered the risk of kidney disease progression or cardiovascular death across a broad CKD population.

The key is whether you have an indication, such as CKD, albuminuria/protein in urine, type 2 diabetes with kidney risk, or heart failure not whether your HbA1c is high. If your kidney function is normal, urine albumin is normal, and you have no heart failure or CKD risk, the benefit may be smaller and it may not be needed.

Discuss with your clinician using your eGFR and urine albumin to creatinine ratio (uACR). Also ask about risks: genital yeast/urinary infections, dehydration or low blood pressure, a small initial eGFR "dip," and rare ketoacidosis especially around surgery, prolonged fasting, or severe illness, when SGLT2 inhibitors are often held.

model: Gemini

**answer:**

Yes, absolutely. In fact, major medical guidelines now recommend SGLT2 inhibitors (like Jardiance, Farxiga, or Invokana) specifically for kidney and heart protection, regardless of what your blood sugar levels are.While these medications were originally created to treat type 2 diabetes, large clinical trials discovered that their ability to protect the kidneys is a standalone benefit. It works independently of their blood sugar lowering effects. Here is why they still protect your kidneys even if your A1C is already perfect:1. They Lower the "Physical Stress" Inside Your KidneysWhen you have diabetes or chronic kidney disease, the filtering units of your kidneys (called glomeruli) are often forced to work in overdrive. This hyperfiltration creates high blood pressure inside the kidney's delicate filters, wearing them out over time. SGLT2 inhibitors change how sodium is processed, which naturally triggers the blood vessels entering the kidney to constrict slightly. This reduces that internal high pressure, giving your kidney filters a chance to rest and preventing long term "burnout." 2. They Reduce Protein Leaking (Albuminuria)By easing that internal fluid pressure, SGLT2 inhibitors significantly reduce the amount of protein that leaks into your urine. Less protein leaking means less inflammation and scarring in the kidney tissue over time. 3. They Improve Local Oxygen DeliveryBy shifting how the kidney reabsorbs nutrients, these medications decrease the heavy workload on the early parts of the kidney tubules. This lowers the kidney's overall oxygen demand and improves oxygen delivery throughout the organ, keeping the tissue healthier. What to expect when you start: Because SGLT2 inhibitors lower the internal filtering pressure to protect the kidney long term, it is completely normal to see a slight, initial drop in your eGFR (kidney function number) when you first start taking them. Think of it like taking your foot off the gas pedal of a car that's been redlining the immediate slowdown is actually saving the engine from burning out. Because they work so effectively through these mechanical and structural pathways, they are even prescribed to patients with chronic kidney disease who don't have diabetes at all. If your blood sugar is already well controlled, the medication simply adapts it will still protect your organs, but it won't force your blood sugar down into dangerously low (hypoglycemic) ranges.

model: DeepSeek

**answer:**

That's an excellent question, and the answer is yes, absolutely. In fact, for many people, the kidney protective benefits of SGLT2 inhibitors are now considered independent of their blood sugar lowering effects.

Here's the breakdown of why they work, even when your A1C is already at goal.

The Short Answer

Modern research clearly shows that SGLT2 inhibitors (like empagliflozin, dapagliflozin, canagliflozin) protect the kidneys through several mechanisms that have nothing to do with lowering blood sugar. Therefore, even if your glucose is perfectly controlled with diet, exercise, or other medications, adding an SGLT2 inhibitor can provide significant additional protection for your kidneys.

How They Protect Kidneys Beyond Blood Sugar

Think of SGLT2 inhibitors as having two separate jobs: a glucose job and a hemodynamic job (affecting pressure and flow within the kidney). It's the second job that matters most for kidney protection.

Restoring Tubuloglomerular Feedback (The Key Mechanism): High blood sugar and other factors can cause the tiny filtering units in your kidneys (nephrons) to work in overdrive. This internal "high pressure" slowly destroys them. SGLT2 inhibitors reduce the workload by telling the nephron to relax, lowering this harmful internal pressure. This protects the filters even if your blood sugar is normal.

Reducing Intraglomerular Pressure: By relaxing the artery that brings blood into the filter (the afferent arteriole), these drugs directly lower the pressure inside the glomerulus. This is a unique effect, similar to how an ACE inhibitor or ARB works, but through a different pathway.

Reducing Inflammation and Fibrosis: High pressure and metabolic stress can trigger inflammation and scarring (fibrosis) in the kidney, even with normal blood sugar. SGLT2 inhibitors have direct anti inflammatory and anti fibrotic effects on kidney cells.

What the Clinical Trials Show

Large, landmark trials have proven this beyond doubt:

EMPA KIDNEY and DAPA CKD trials: These studies included thousands of people with chronic kidney disease (CKD). A large percentage of them did not have type 2 diabetes (they had normal blood sugar). In both groups those with and without diabetes the SGLT2 inhibitor significantly slowed the progression of kidney disease, reduced the risk of kidney failure, and lowered the risk of death.

For people with diabetes and already well controlled blood sugar, the benefit was just as strong as for those with poor control.

When Is It Most Beneficial for You?

Your doctor would most likely consider adding an SGLT2 inhibitor specifically for kidney protection if you have:

Chronic Kidney Disease (CKD): Especially if your eGFR (estimated glomerular filtration rate) is between 25 and 90, and you have albuminuria (protein in your urine, like a UACR greater than 30 mg/g).

Type 2 Diabetes with risk factors: Even with a good A1C, if you have microalbuminuria or early CKD, you are a prime candidate.

Heart Failure: These drugs are also powerful for heart failure, and kidney protection comes along for the ride.

Important Considerations

They are not for everyone: SGLT2 inhibitors are generally not started if your eGFR is very low (e.g, below 20 to 25 mL/min/1.73m2, depending on the drug), if you have a history of frequent urinary tract infections or genital yeast infections, or if you are prone to dehydration or low blood pressure.

Risk of Euglycemic DKA: This is a rare but serious side effect. Even with normal blood sugar levels, the drug can, in certain circumstances (like severe illness, fasting for surgery, very low carb diets, or heavy alcohol use), cause a form of diabetic ketoacidosis without high sugar. Your doctor will advise on "sick day rules" (stopping the drug during vomiting, diarrhea, or before major procedures).

They are an add on therapy: You would take this alongside your current kidney protective medications (like an ACE inhibitor or ARB), not instead of them.

The Bottom Line

Do not think of SGLT2 inhibitors as just "diabetes drugs." They are now considered fundamental kidney and heart protective drugs that happen to also lower blood sugar.

If your blood sugar is well controlled, you are already doing great. But you may still have silent, ongoing pressure and inflammation inside your kidneys that an SGLT2 inhibitor could help. Have a direct conversation with your nephrologist or endocrinologist: "My A1C is good, but I want to know if adding an SGLT2 inhibitor would give me additional long term protection for my kidneys."

They can check your urine protein (UACR) and eGFR to see if you are a candidate. For many people with even mild kidney risk, the answer is a clear yes.

model: Doubao

**answer:**

Yes, SGLT2 inhibitors exert independent kidney protection that is NOT dependent on blood sugar levels. Even if your HbA1c and daily glucose are perfectly controlled, these drugs still deliver meaningful renal benefits for people with diabetic kidney disease (DKD) or chronic kidney disease (CKD).

1. Mechanisms separate from glucose lowering

The kidney protective effects come from multiple pathways unrelated to blood sugar reduction:

Lower intraglomerular hypertension (core mechanism)

SGLT2 inhibitors reduce sodium and glucose reabsorption in the proximal tubule, which dilates the afferent renal arteriole and lowers pressure inside the glomerulus. High glomerular pressure is the main driver of progressive kidney scarring, proteinuria and eGFR decline this effect occurs regardless of how low your blood sugar is.

Reduction of albuminuria / urinary protein loss

They cut urinary albumin excretion significantly, a key marker of kidney damage progression, independent of glycemia.

Anti inflammatory and anti fibrotic effects in renal tissue

They suppress renal oxidative stress, inflammatory cytokines, and collagen deposition that causes permanent kidney scarring. These tissue level protective actions do not rely on glucose lowering.

Lower systemic blood pressure and weight loss

Mild diuresis and calorie loss reduce volume overload and blood pressure, easing kidney burden separately from glycemic control.

2. Clinical trial evidence

Major landmark trials confirm renal benefit persists in well controlled glycemia groups:

DAPA CKD, EMPA KIDNEY: These trials included participants with type 2 diabetes and non diabetic CKD, many with near target HbA1c. SGLT2 inhibitors slowed eGFR decline, reduced kidney failure risk, and cut renal hospitalization/death even in subgroups with optimal glucose control.

Subgroup analyses: The magnitude of kidney protection did not weaken in patients with HbA1c less than 7.0% (tight glycemic targets). The renal risk reduction was consistent across all glucose strata.

For patients already on insulin/GLP 1 agonists with stable normal blood sugar: Adding an SGLT2 inhibitor still slows kidney disease progression.

3. Practical clinical implication

If you have:

Type 2 diabetes plus CKD (elevated urine albumin, reduced eGFR)

OR

CKD without diabetes

SGLT2 inhibitor is indicated for kidney protection regardless of whether blood glucose is already well managed.

Your clinician may even keep you on a low dose SGLT2 inhibitor solely for renal/cardiac protection, without expecting further glucose lowering.

4. Important caveats

You still need standard glycemic targets to avoid additional glucose mediated kidney damage; SGLT2i kidney protection complements not replaces good blood sugar control.

Monitor for side effects regardless of glucose levels: genital mycotic infections, volume depletion, acute kidney injury risk at initiation, euglycemic diabetic ketoacidosis (rare but possible even with normal blood sugar).

Dose adjustment is required based on eGFR thresholds per drug labeling.

Summary

Blood glucose control and SGLT2 inhibitor kidney protection work through distinct pathways. Tight glycemia does not negate the renal protective effects of SGLT2 inhibitors, and they remain indicated to slow chronic kidney disease progression even when sugars are already well controlled.

model: Kimi

**answer:**

Yes. SGLT2 inhibitors can protect your kidneys even if your blood sugar is already well controlled. In fact, a large body of evidence shows that their kidney protective effects are largely independent of glucose lowering.

Key Evidence

Protection without diabetes: The DAPA CKD trial tested dapagliflozin in people with chronic kidney disease including one third who did not have diabetes at all. Dapagliflozin reduced the risk of kidney decline, end stage kidney disease, and cardiovascular death by 50% in the non diabetic group and 36% in the diabetic group. Since people without diabetes have no excess blood sugar to correct, this demonstrates that the benefit cannot be explained by glucose control alone.

Benefits independent of baseline HbA1c: In the CREDENCE trial (canagliflozin), the drug slowed kidney function decline and reduced albuminuria progression regardless of the patient's starting blood sugar level, type of blood pressure medication, or cardiovascular disease status.

Low dose preclinical studies: Animal research has shown that very low doses of SGLT2 inhibitors can protect the kidneys without producing enough glucosuria to lower blood glucose, suggesting direct renal tissue effects.

How They Protect Kidneys (Beyond Glucose)

SGLT2 inhibitors protect kidneys through several non glycemic mechanisms:

Tubuloglomerular feedback: By causing more sodium to reach the macula densa in the kidney, SGLT2 inhibitors trigger constriction of the afferent arteriole in the glomerulus. This lowers pressure inside the glomerular filter, reducing hyperfiltration damage a mechanism that works regardless of blood sugar.

Anti inflammatory and anti fibrotic effects: They reduce inflammatory signaling and scarring in kidney tissue.

Improved energy metabolism: They enhance mitochondrial function and shift renal cells toward more efficient fuel use (ketone bodies), reducing oxidative stress.

Blood pressure and volume: A modest, sustained reduction in blood pressure and improved fluid distribution also contribute.

Direct effects on kidney cells: Some evidence suggests SGLT2 inhibitors act directly on mesangial cells in the kidney to reduce damage pathways, independent of systemic glucose changes.

Clinical Takeaway

Major guidelines now recommend SGLT2 inhibitors for kidney protection and heart failure regardless of whether the patient has diabetes, precisely because the cardiorenal benefits are separate from the glucose lowering effect.

Important caveat: This is general medical information. Whether an SGLT2 inhibitor is appropriate for you depends on your specific kidney function (eGFR), albuminuria levels, other medications, and risk factors. You should discuss this with your doctor or nephrologist, who can evaluate if the benefits outweigh any risks in your individual case.

model: Qwen

**answer:**

Yes, they can still protect your kidneys. SGLT2 inhibitors provide renal benefits through mechanisms that function independently of glucose control.

Mechanisms of Kidney Protection

SGLT2 inhibitors offer more than just blood sugar management. Their protective effects work independently of blood sugar levels through several pathways:

Reducing Kidney Stress: They improve blood flow within the kidneys, lowering the internal pressure on the filtering units (glomeruli). This reduces the risk of kidney damage caused by high pressure.

Anti inflammatory Effects: They help decrease inflammation and tissue scarring (fibrosis) within the kidneys, helping to preserve kidney function for the long term.

Energy Metabolism: They improve how kidney cells use energy and reduce oxidative stress, protecting cells even in challenging environments.

Who Can Benefit

Because these mechanisms are independent of blood sugar, SGLT2 inhibitors are proven to protect kidney function in people with diabetes, as well as in those with chronic kidney disease who do not have diabetes.

Important Considerations

Even though they offer significant benefits, there are practical things to keep in mind:

Hygiene: Since the drug causes sugar to be excreted in urine, it can increase the risk of urinary tract infections. Staying well hydrated and maintaining good hygiene is important.

Dehydration Risk: These medications have a mild diuretic effect. During illnesses involving fever, vomiting, or diarrhea, you need to be careful not to become dehydrated, as this can strain kidney function.

Regular Monitoring: It is essential to have regular check ups to monitor kidney function markers, such as blood creatinine and urine albumin.

In summary, SGLT2 inhibitors remain a powerful tool for protecting your kidneys even when your blood sugar is well controlled. You should continue taking them as directed by your doctor and attend regular check ups to monitor your kidney health.

**Q31. What side effects of SGLT2 inhibitors should I watch for?**

model: ChatGPT

**answer:**

SGLT2 inhibitors include empagliflozin, dapagliflozin, canagliflozin, and ertugliflozin. They are often used for type 2 diabetes, chronic kidney disease, and heart failure. Common side effects are usually manageable, but a few rare effects need urgent attention.

Common side effects to watch for

1. Genital yeast/fungal infections

Watch for itching, redness, soreness, unusual discharge, burning, or irritation around the vagina or penis. These are among the most common side effects and are usually treatable.

2. Urinary tract infection symptoms

Watch for burning when urinating, frequent or urgent urination, lower abdominal pain, fever, flank/back pain, or feeling generally unwell. Serious UTIs such as pyelonephritis or urosepsis have been reported, so worsening symptoms should be treated promptly.

3. More urination, thirst, dehydration, dizziness, or low blood pressure

These medicines make you pass more glucose and fluid in the urine. Be alert for lightheadedness, fainting, very dry mouth, unusual weakness, or reduced urination especially if you are older, taking diuretics/water pills, have kidney disease, or are not eating/drinking well.

4. Low blood sugar mainly if combined with insulin or sulfonylureas

SGLT2 inhibitors alone rarely cause hypoglycemia, but risk increases if you also take insulin or drugs such as gliclazide, glimepiride, or glipizide. Watch for sweating, shaking, hunger, confusion, palpitations, or dizziness.

Rare but urgent warning signs

Seek urgent medical care now if you have symptoms of diabetic ketoacidosis (DKA): nausea/vomiting, abdominal pain, rapid or deep breathing, severe fatigue, confusion, dehydration, or fruity/acetone smelling breath. With SGLT2 inhibitors, DKA can occur even when blood sugar is not very high, so normal glucose does not fully rule it out.

Also seek urgent care for possible Fournier's gangrene, a very rare but life threatening infection: severe pain, tenderness, redness, swelling, fever, or feeling very unwell around the genitals, anus, or area between them.

Situations where you should ask about pausing it

Contact your prescriber about "sick day rules" if you are vomiting, have diarrhea, are dehydrated, fasting, eating very little, on a very low carb/keto diet, or preparing for surgery/procedures. Many patient guidance sheets advise temporarily holding SGLT2 inhibitors during significant illness or fasting and restarting after you are eating and drinking normally, but follow your clinician's specific instructions.

Also tell your clinician promptly about foot ulcers, wounds, discoloration, circulation problems, or foot pain, especially if you have diabetes, neuropathy, peripheral vascular disease, or a prior foot ulcer/amputation.

model: Gemini

**answer:**

SGLT2 inhibitors (like Jardiance, Farxiga, and Invokana) work by causing your kidneys to flush excess glucose out of your body through your urine. Because of how they work, the side effects are usually tied to that extra sugar and fluid leaving your system.

Here is what you should watch for, categorized by how common or urgent they are:

Common Side Effects (Monitor closely)

Genital Yeast Infections: This is the most common side effect. Because there is more sugar in your urine, it creates an environment where yeast can thrive. Watch for itching, redness, or soreness. It affects both women and men.

Urinary Tract Infections (UTIs): For the same reason, bacteria can grow more easily. Watch for a burning sensation when urinating, an urgent need to go, or cloudy urine.

Increased Urination and Dehydration: You will likely find yourself using the bathroom more frequently. This can lead to mild dehydration, causing a dry mouth, increased thirst, or lightheadedness when you stand up too quickly (due to a slight drop in blood pressure).

Rare but Serious Side Effects (Seek immediate medical care)

Diabetic Ketoacidosis (DKA): This is a serious condition where the body builds up too much acid (ketones). With SGLT2 inhibitors, DKA can happen even if your blood sugar looks completely normal (called euglycemic DKA). Go to an emergency room immediately if you experience:

Nausea, vomiting, or severe abdominal pain

Shortness of breath or rapid breathing

Confusion, unusual fatigue, or a sweet/fruity odor to your breath

Fournier's Gangrene: This is an incredibly rare but life threatening bacterial infection of the perineal area (the genitals and the area between the genitals and anus). Seek emergency care if you develop pain, tenderness, redness, or swelling in that area accompanied by a fever or a general feeling of being unwell.

Staying Hydrated: To minimize the risk of dehydration and dizziness, make sure you are drinking plenty of water throughout the day, especially in hot weather or if you are exercising.

Disclaimer: I am an AI, not a doctor. If you are experiencing uncomfortable side effects or suspect something serious like DKA, contact your healthcare provider or seek emergency medical care immediately.

model: DeepSeek

**answer:**

This is an excellent question, as SGLT2 inhibitors (such as empagliflozin, dapagliflozin, canagliflozin, and ertugliflozin) are effective but have unique risks.

You should seek medical attention immediately if you experience any of the following serious side effects.

1. Life Threatening but Rare Side Effects

Euglycemic Diabetic Ketoacidosis (DKA): Unlike typical DKA, your blood sugar may be only slightly elevated or even normal. Watch for: nausea, vomiting, abdominal pain, extreme fatigue, trouble breathing, or confusion. Stop the medication and seek emergency care if these occur, especially if you are sick, reducing insulin, fasting, or drinking heavily.

Fournier's Gangrene (Necrotizing Fasciitis of the Perineum): A rare but rapidly progressive soft tissue infection. Watch for: tenderness, redness, or swelling in the genital or rectal area, along with fever or feeling unwell. Do not wait this requires urgent surgery and antibiotics.

2. Common Side Effects You'll Likely Notice

Genital Mycotic Infections: Yeast infections (balanitis in men, vulvovaginitis in women) are very common. Watch for: itching, redness, discharge, or pain in the genital area. Over the counter antifungals often work, but see a doctor if recurrent.

Urinary Tract Infections (UTIs): Increased risk, though less common than genital infections. Watch for: painful/burning urination, frequent urge, cloudy or bloody urine, or lower abdominal pain. Some UTIs can progress to pyelonephritis (kidney infection) with fever and back pain.

Increased Urination and Thirst (Polyuria/Polydipsia): Due to osmotic diuresis. Usually mild, but can lead to dehydration or low blood pressure if you don't drink enough fluids.

3. Important Metabolic and Kidney Effects

Hypotension (Low Blood Pressure): Particularly risky if you already take diuretics, ACE inhibitors, or ARBs. Watch for: dizziness, lightheadedness, or fainting upon standing. Ensure adequate salt/fluid intake and monitor your BP.

Dehydration and Acute Kidney Injury (AKI): Risk rises with use of diuretics, NSAIDs (ibuprofen, naproxen), or during illness. Watch for: markedly reduced urination, dry mouth, rapid heartbeat, or confusion.

Hypoglycemia: Not common alone, but risk increases dramatically if you also take insulin or sulfonylureas (glipizide, glyburide). Watch for: shakiness, sweating, confusion, or hunger.

4. Other Side Effects to Monitor

Hyperkalemia (High Potassium): More likely if you have chronic kidney disease or take ACE inhibitors/ARBs. Usually asymptomatic but can cause muscle weakness or dangerous heart rhythms.

Bone Fractures and Leg/Foot Amputations: Primarily linked to canagliflozin. Increased risk of lower limb amputations (toe, foot) and reduced bone density. Notify your doctor of any foot pain, ulcers, or new bone pain.

Acute Pancreatitis: Rare. Watch for: severe upper abdominal pain radiating to the back, with nausea/vomiting.

When to Call Your Doctor (Non Emergency)

First yeast infection or UTI (to confirm diagnosis)

Persistent dizziness or fatigue (may need dose adjustment or fluid advice)

Mild rash or allergic reaction

Any unexpected muscle pain or weakness

Key Preventive Actions

Stay hydrated drink enough fluid to avoid dehydration.

Practice good hygiene to reduce genital infection risk.

Stop temporarily during "sick days" (vomiting, diarrhea, unable to eat/drink) ask your doctor for sick day rules.

Inspect your feet daily (especially with canagliflozin) for any sores or color changes.

Disclaimer: This is not a substitute for professional medical advice. Always report new or worsening symptoms to your prescribing physician, and read your medication guide carefully.

model: Doubao

**answer:**

SGLT2 inhibitors (e.g, dapagliflozin, empagliflozin, canagliflozin, ertugliflozin) lower blood sugar by excreting glucose through urine. Their adverse effects are mostly linked to glycosuria and metabolic shifts, divided into common mild effects and rare but serious life threatening complications.

1. Common, Mild Side Effects (Most frequent)

Genital and Urinary Tract Infections

Mechanism: Extra glucose in urine feeds bacteria/fungi.

Signs:

Women: Vaginal yeast infection (itching, white discharge, burning)

Men: Balanitis (red, itchy foreskin/glans)

Urinary tract infection (UTI): frequent painful urination, suprapubic pain, cloudy urine

Risk tips: Drink enough water, maintain genital hygiene.

Polyuria, Dehydration and Orthostatic Hypotension

Increased urine output leads to fluid loss.

Symptoms: frequent urination, thirst, dry mouth, dizziness, lightheadedness when standing, low blood pressure, fatigue.

High risk groups: elderly patients, diuretic users, low fluid intake, renal impairment.

Mild Dyslipidemia

Small rises in LDL cholesterol, usually clinically insignificant.

2. Less Common Metabolic Side Effects

Weight loss

Desirable for overweight diabetics but may cause unwanted thinness in underweight patients.

Mild Ketoacidosis (euglycemic DKA, critical to recognize)

Unique to SGLT2 inhibitors: normal or only slightly elevated blood glucose (often less than 13.9 mmol/L / 250 mg/dL), so easy to misdiagnose.

Warning symptoms: persistent nausea, vomiting, abdominal pain, deep rapid breathing, fatigue, confusion, sweet/fruity breath odor.

Triggers: fasting, surgery, infection, reduced insulin intake, heavy exercise, alcohol use.

Emergency: Stop drug immediately and seek hospital care.

3. Rare Severe Adverse Reactions (Require immediate medical attention)

Fournier's Gangrene (Necrotizing Fasciitis of the Perineum)

Rare but life threatening bacterial soft tissue infection of genital/perineal area.

Red flags: severe groin/scrotal/vulvar pain, swelling, fever, skin discoloration (purple/black), foul discharge. Requires urgent surgical debridement plus IV antibiotics.

Acute Kidney Injury

Fluid depletion, reduced renal perfusion can trigger acute kidney damage.

Warning signs: sharp drop in urine volume, swelling, fatigue, elevated creatinine. Avoid concurrent NSAIDs and limit diuretics.

Lower Limb Amputation Risk (Canagliflozin specific)

Clinical trials showed slightly higher risk of foot/toe amputations with canagliflozin. Monitor foot ulcers, peripheral artery disease closely; prioritize foot self care.

Bone Fractures (Canagliflozin)

Increased fracture risk due to calcium/phosphate urinary loss; avoid in patients with osteoporosis.

Hyperkalemia

Mild risk of elevated blood potassium, especially in CKD, ACEI/ARB users. Monitor serum potassium regularly.

4. Other Minor Effects

Constipation, nausea

Joint pain, back pain

Increased thirst

Key Monitoring Advice

Stay well hydrated daily to reduce dehydration and infection risk.

Stop the medication and contact a doctor before surgery, prolonged fasting, or severe illness.

Learn euglycemic DKA symptoms do not dismiss nausea/vomiting as simple stomach upset.

Check renal function, electrolytes, and LDL at follow up visits.

Report any genital pain, perineal swelling, or persistent dizziness promptly.

model: Kimi

**answer:**

SGLT2 inhibitors can protect the heart and kidneys, but they may cause side effects.

Common problems include genital yeast infections, urinary tract infections, increased urination, thirst, dizziness, and dehydration. Low blood sugar is more likely when these medicines are taken with insulin or sulfonylureas.

Rare but serious problems include diabetic ketoacidosis, severe genital infections, acute kidney injury, and severe allergic reactions. Seek medical care immediately if you have vomiting, stomach pain, confusion, rapid breathing, severe genital pain or swelling, very little urine, facial swelling, or difficulty breathing.

Canagliflozin may also be linked to a higher risk of bone fractures and lower-limb amputation. Drink enough fluids, maintain good genital hygiene, and ask your clinician whether to stop the medicine temporarily during fasting, severe illness, vomiting, diarrhea, or before surgery.

model: Qwen

**answer:**

SGLT 2 inhibitors most commonly cause genitourinary tract infections. The rarest but most serious risk is diabetic ketoacidosis (DKA).

Common Side Effects to Watch For

Genitourinary Tract Infections: Since these drugs remove excess sugar through urine, the increased sugar in the urine can promote bacterial growth. Women are more prone to yeast infections (itching/discharge), while both men and women may experience urinary tract infections (burning/frequent urination). Staying hydrated and keeping the area clean can help prevent this.

Dehydration and Low Blood Pressure: By excreting sugar, the body also loses water, which can lower blood volume. You might feel dizzy or lightheaded when standing up, especially when you first start the medication or if you are already taking diuretics.

Rare but Serious Warning Signs

Diabetic Ketoacidosis (DKA): This is a dangerous condition that can occur even when blood sugar is normal. It is often triggered by surgery, severe infection, fasting, or extreme low carb diets. Watch for unexplained nausea, vomiting, abdominal pain, deep rapid breathing, and extreme weakness. Seek emergency care immediately if these occur.

Fournier's Gangrene: This extremely rare but severe bacterial infection affects the genitals or perineum. Seek immediate emergency care if you experience severe pain, redness, swelling, or fever in the genital area.

Practical Tips for Safe Use

Drink Plenty of Water: Stay hydrated, especially during hot weather, exercise, or illness.

Monitor for Low Blood Sugar: If you are also taking insulin or sulfonylureas, your risk of hypoglycemia increases.

Stop Before Surgery: You usually need to temporarily stop this medication before elective surgeries, during severe acute illness, or when food intake is severely restricted.

SGLT 2 inhibitors offer great heart and kidney protection, but staying vigilant about these symptoms ensures you can use them safely.

**Q32. What are ACE inhibitors or ARBs, and why are they used when there is protein in the urine?**

model: ChatGPT

**answer:**

ACE inhibitors and ARBs are two related groups of blood pressure medicines that also protect the kidneys.

ACE inhibitors include medicines such as lisinopril, enalapril, ramipril.

ARBs include medicines such as losartan, valsartan, irbesartan. They work on the renin angiotensin system, a hormone system that can tighten blood vessels and raise pressure inside the kidney's tiny filters. ACE inhibitors reduce production of angiotensin II, while ARBs block its action. The end result is relaxed blood vessels and lower pressure on the kidneys.

When there is protein or albumin in the urine, it usually means the kidney filters are "leaky" or under stress. These medicines are used because they can reduce albumin/protein leakage, lower pressure inside the kidney filters, and slow worsening of chronic kidney disease, especially in people with diabetes, high blood pressure, or chronic kidney disease. The National Kidney Foundation describes them as kidney protective medicines that lower uACR, a common urine albumin test.

Current KDIGO kidney guidelines recommend starting an ACE inhibitor or ARB for people with CKD and moderately or severely increased albuminuria, including those with diabetes. Albuminuria categories are commonly defined as ACR 30 to 300 mg/g for moderately increased and greater than 300 mg/g for severely increased.

A few important cautions: patients usually take either an ACE inhibitor or an ARB, not both together. After starting or increasing the dose, clinicians typically check blood pressure, blood creatinine/eGFR, and potassium within 2 to 4 weeks, because these medicines can sometimes cause low blood pressure, a rise in creatinine, or high potassium.

In simple terms: protein in the urine is a warning sign that the kidney filters are being damaged; ACE inhibitors and ARBs help lower the stress on those filters and reduce leakage, which can protect kidney function over time.

model: Gemini

**answer:**

Both ACE inhibitors (Angiotensin Converting Enzyme inhibitors) and ARBs (Angiotensin II Receptor Blockers) are classes of medications primarily known for lowering blood pressure. However, doctors frequently prescribe them for a completely different superpower: protecting the kidneys.

When a lab test shows protein in your urine (a condition called proteinuria), it is a major warning sign that the kidneys' filtration systems are under too much stress.

The Coffee Filter Analogy

To understand why these medications help, it helps to imagine how the kidneys filter blood.

Inside your kidneys are millions of tiny filtering units called glomeruli. Think of each glomerulus as a delicate coffee filter or a fine mesh strainer.

Normal function: The mesh is tight enough to let waste and extra water pass through into your urine, while keeping large, valuable things like proteins and red blood cells in your bloodstream.

The problem: If the blood pressure pushing into that tiny filter is too high, it forcefully stretches the mesh. Over time, holes form, and protein starts leaking out into the urine.

How ACE Inhibitors and ARBs Fix It

Every microscopic kidney filter has an "inlet" pipe bringing blood in, and an "outlet" pipe taking blood away.

ACE inhibitors and ARBs block a hormone called Angiotensin II, which normally causes blood vessels to constrict (narrow). By blocking this hormone, these medications specifically dilate (widen) the outlet pipe leaving the kidney filter.

Relieving the backpressure: Widening the exit pipe is like opening a second lane on a congested highway. Blood flows out of the filter much more easily, which instantly drops the intraglomerular pressure (the physical pressure inside the filter itself).

Stopping the leak: With the intense pressure relieved, the mechanical force tearing at the filter's mesh stops. This slows down or completely halts the leaking of protein.

Preventing scarring: Protein itself is actually irritating to the kidney tubules when it leaks through. By stopping the leak, these drugs prevent long term inflammation and scarring (kidney damage).

Who benefits from this?

Because of this protective mechanism, doctors will often prescribe an ACE inhibitor (like lisinopril or enalapril) or an ARB (like losartan or valsartan) to patients who have protein in their urine, even if their overall blood pressure is completely normal.

This treatment is a cornerstone of medical care for people with diabetes, chronic kidney disease (CKD), or hypertension, as it is one of the most effective ways to prevent kidney damage from progressing over time.

model: DeepSeek

**answer:**

This is an excellent question that sits at the intersection of cardiology and nephrology.

Here's a breakdown of what ACE inhibitors and ARBs are, and the specific reason they are the go to drugs for protein in the urine (proteinuria).

What are ACE Inhibitors and ARBs?

Both are classes of blood pressure medications, but they work on the same biological system (the Renin Angiotensin Aldosterone System, or RAAS) in slightly different ways.

ACE Inhibitors (Angiotensin Converting Enzyme Inhibitors): Drugs like lisinopril, enalapril, and ramipril. They block the enzyme that converts Angiotensin I to Angiotensin II (a very potent vasoconstrictor).

ARBs (Angiotensin II Receptor Blockers): Drugs like losartan, valsartan, and irbesartan. They block the receptor site where Angiotensin II exerts its effects.

The final result is the same for both: they lower blood pressure by relaxing blood vessels and reduce the effect of a powerful hormone called Angiotensin II.

Why are they used when there is protein in the urine?

Protein in the urine is a sign that the filtering units of the kidney (glomeruli) are damaged. Normally, these filters act like a fine sieve, keeping large molecules like protein (especially albumin) in the blood. When damaged, the "holes" in the sieve get bigger, and protein leaks through.

ACE inhibitors and ARBs are used to protect the kidneys for two critical reasons, independent of their blood pressure lowering effect:

1. They Lower Pressure Inside the Kidney Filter (Intraglomerular Pressure)

Think of the kidney filter like a strainer for pasta. If you push water through the strainer with very high pressure, it will force more pasta (protein) through and eventually damage the strainer further.

Angiotensin II constricts the blood vessel leaving the kidney filter (the efferent arteriole). This creates a "traffic jam" of blood behind it, which increases the pressure inside the delicate filter.

ACE inhibitors and ARBs block this constriction. They allow the outflow vessel to relax and open up, reducing the internal pressure on the damaged filter.

Result: Less physical stress on the filter means less protein is forced out, and the rate of further damage slows down significantly.

2. They Directly Protect the Filter's Structure (Anti fibrotic Effect)

Chronic exposure to Angiotensin II doesn't just raise pressure; it also acts as a growth factor that promotes scarring and fibrosis (hardening) of the kidney tissue. It directly damages the barrier cells of the filter (podocytes).

By blocking Angiotensin II, ACE inhibitors and ARBs reduce this direct chemical injury.

Result: They help preserve the long term structural integrity of the filter, slowing the progression toward chronic kidney disease (CKD) and kidney failure.

The Key Clinical Point

In patients with proteinuria (e.g, from diabetes, hypertension, or glomerulonephritis), doctors will prescribe an ACE inhibitor or ARB even if their blood pressure is normal.

The goal is not just to lower systemic blood pressure but to specifically lower the intraglomerular pressure and reduce protein leakage to preserve kidney function for as long as possible.

Important Note: They Are Not Used Together

You will usually be prescribed either an ACE inhibitor or an ARB, but never both at the same time.

A landmark study (the ONTARGET trial) showed that combining them caused more harm (dangerously high potassium, sudden kidney failure) without providing extra benefit compared to using one alone.

Simple Analogy

Damaged kidney filter is A hole in a garden hose.

Angiotensin II is Someone turning up the water pressure at the spigot, making the hole shoot out water and get larger.

ACE inhibitor/ARB is Someone turning the water pressure down at the spigot. The leak slows down, and the hose is protected from further tearing.

In summary: ACE inhibitors and ARBs are the most kidney protective medications available for proteinuria because they lower the damaging high pressure inside the kidney's filters and directly reduce scarring, slowing the progression of kidney disease.

model: Doubao

**answer:**

ACE Inhibitors and ARBs: Definition plus Mechanism for Urinary Proteinuria

1. What are ACE inhibitors?

ACE is Angiotensin Converting Enzyme

ACE inhibitors block the enzyme ACE, which converts angiotensin I to angiotensin II (a powerful vasoconstrictor hormone).

Representative drugs: Lisinopril, Enalapril, Ramipril, Captopril

Core effects:

Dilate blood vessels to lower systemic blood pressure

Reduce aldosterone secretion to cut sodium/water retention

Suppress excessive renin angiotensin aldosterone system (RAAS) activation

2. What are ARBs?

ARB is Angiotensin II Receptor Blockers

Instead of blocking ACE, ARBs directly bind and block angiotensin II type 1 (AT1) receptors on blood vessels, kidneys and heart, stopping angiotensin II from acting.

Representative drugs: Losartan, Valsartan, Irbesartan, Telmisartan

Similar overall effects to ACE inhibitors, with fewer dry cough side effects (a common adverse reaction of ACE inhibitors).

3. Why they treat urine protein (proteinuria)

Proteinuria means albumin/protein leaks through damaged kidney glomeruli (the tiny filtering units of kidneys). The RAAS system damages glomeruli in two key ways:

Increases intraglomerular pressure

Angiotensin II constricts the efferent arteriole (the small blood vessel leaving the glomerulus) more strongly than the afferent arteriole entering it. This traps high pressure inside the glomerulus, stretching and rupturing the glomerular filtration barrier, letting protein leak into urine.

ACEi/ARBs dilate the efferent arteriole, lowering pressure inside the glomerulus directly this is their most critical kidney protective action.

Reduces glomerular barrier inflammation and scarring

Overactive angiotensin II triggers inflammation, fibrosis (scar tissue buildup), and podocyte damage (podocytes are the filter cells that stop protein loss). By inhibiting RAAS, these drugs protect podocytes, stabilize the filtration membrane, and slow irreversible kidney scarring.

Lowers systemic blood pressure indirectly

High systemic hypertension worsens glomerular damage. Lowering blood pressure further reduces mechanical stress on kidney filters.

4. Clinical benefits in proteinuria patients

Reduce urinary albumin/protein excretion significantly

Slow decline of kidney function (lower rate of eGFR drop)

Delay progression of chronic kidney disease (CKD), diabetic nephropathy, hypertensive nephropathy

Reduce risk of end stage renal disease (ESRD)

Key note

Even in patients with normal blood pressure but significant proteinuria, ACE inhibitors or ARBs are routinely prescribed solely for their renoprotective, anti proteinuric effects. Dose adjustment and monitoring of serum creatinine and potassium are required during treatment.

model: Kimi

**answer:**

ACE inhibitors and ARBs are two classes of blood pressure medications that also have special protective effects on the kidneys. Here's what they are and why they're used when protein appears in the urine.

What They Are

ACE inhibitors (Angiotensin Converting Enzyme inhibitors) such as lisinopril, enalapril, and ramipril block the production of angiotensin II, a hormone that narrows blood vessels and raises blood pressure.

ARBs (Angiotensin II Receptor Blockers) such as losartan, valsartan, and irbesartan work one step downstream: they block angiotensin II from attaching to its receptors, achieving a similar blood pressure lowering effect.

Both classes target the renin angiotensin aldosterone system (RAAS), a key hormonal system that regulates blood pressure and kidney function.

Why They're Used When There's Protein in the Urine

Protein in the urine (proteinuria) usually signals that the kidney's filtering units (glomeruli) are damaged or under excessive stress. Under normal conditions, the glomeruli are selective barriers that keep large proteins like albumin inside the bloodstream. When these barriers are compromised, protein leaks into the urine and this leakage itself is both a sign of damage and a cause of further injury.

ACE inhibitors and ARBs help in three main ways:

1. They reduce pressure inside the glomeruli

Angiotensin II causes constriction of the efferent arteriole in the kidney, which raises the pressure gradient across the glomerulus and forces more fluid (and protein) through the filter. By inhibiting this constriction, ACE inhibitors and ARBs lower the pressure inside the glomerular capillaries. This reduces protein leakage and eases the mechanical stress on the filtering barrier.

2. They protect the kidney structure

High glomerular pressure and protein trafficking through the tubules are toxic to kidney cells over time. By reducing both, these medications slow the progression of chronic kidney disease (CKD) and help preserve remaining kidney function.

3. They lower systemic blood pressure

High blood pressure is both a cause and consequence of kidney damage. By controlling hypertension, these drugs reduce the overall workload on the cardiovascular system and the kidneys.

Key Clinical Points

Renoprotection independent of blood pressure: Even in patients whose blood pressure is already well controlled, ACE inhibitors and ARBs can still reduce proteinuria and slow kidney damage. This is why kidney guidelines often recommend them specifically for proteinuric kidney disease, not just for hypertension.

They are not used together routinely: Combining an ACE inhibitor with an ARB generally isn't recommended due to increased risks (such as high potassium levels and acute kidney injury) without proven additional benefit in most patients.

Monitoring is essential: When starting these medications, doctors typically check kidney function and potassium levels, because they can affect both.

In short, ACE inhibitors and ARBs are used in proteinuria because they lower the damaging pressure inside the kidney's filters, reduce protein leakage, and slow the progression of kidney disease effects that go beyond simply controlling blood pressure.

model: Qwen

**answer:**

ACE inhibitors (ACEIs) and ARBs are medications used to reduce intraglomerular pressure, which helps seal leaky filters to decrease proteinuria and protect kidney function.

The Mechanism of Proteinuria

The kidneys act as a precise filtration system. The glomeruli are tiny filters that remove waste from the blood while keeping essential proteins in the bloodstream. When the kidneys are damaged or blood pressure is high, the pressure inside the glomeruli increases. This high pressure forces proteins to leak through the filter and into the urine, a condition known as proteinuria.

How ACEIs and ARBs Work

Both drug classes target the Renin Angiotensin Aldosterone System (RAAS) to lower kidney pressure:

Lowering Kidney Pressure: They specifically dilate the small arteries that carry blood away from the glomerulus (efferent arterioles). This reduces the internal pressure within the kidney filter.

Reducing Protein Leakage: With lower internal pressure, the permeability of the filtration membrane decreases, significantly reducing the amount of protein leaking into the urine.

Slowing Kidney Damage: Long term high pressure causes glomerular scarring (sclerosis). By blocking the hormones that cause this damage, these medications slow down the progression of chronic kidney disease.

Key Differences and Similarities

Mechanism: ACEIs block the production of angiotensin II (a substance that narrows blood vessels), while ARBs directly block angiotensin II from binding to its receptors.

Side Effects: A common side effect of ACEIs is a persistent dry cough. ARBs rarely cause coughing and are generally better tolerated. If a patient develops a cough on an ACEI, doctors often switch them to an ARB.

Clinical Considerations

Do Not Combine: These two drug classes should never be taken together. Their mechanisms overlap, and combining them significantly increases the risk of dangerous side effects like hyperkalemia (high blood potassium).

Contraindications: They are strictly contraindicated in pregnant women, patients with bilateral renal artery stenosis, and those with severe hyperkalemia.

Monitoring: Blood tests for kidney function (creatinine) and electrolytes (potassium) are necessary, especially when starting medication or adjusting doses.

In summary, ACE inhibitors and ARBs are the cornerstone therapy for reducing proteinuria. By effectively lowering intraglomerular pressure, they not only reduce protein loss but also provide critical long term protection against kidney failure.

**Q33. Should I stop my blood pressure medicine if my creatinine rises slightly?**

model: ChatGPT

**answer:**

In most cases, do not stop your blood pressure medicine on your own just because creatinine rises slightly. For ACE inhibitors or ARBs medicines like lisinopril, benazepril, losartan, valsartan, irbesartan a small creatinine rise or small eGFR drop can be expected because these drugs lower pressure inside the kidney filters, and they often protect the kidneys long term. The National Kidney Foundation specifically says ACE inhibitors/ARBs may slightly lower eGFR at first and that this is not necessarily worsening kidney disease.

A common clinical rule is: continue ACEi/ARB unless creatinine rises more than about 30% within 4 weeks of starting or increasing the dose, while also checking potassium and blood pressure. KDIGO 2024 recommends checking BP, serum creatinine, and potassium within 2 to 4 weeks after starting or increasing a renin angiotensin system inhibitor, and continuing unless creatinine rises by more than 30%.

You should contact your clinician promptly rather than stopping it yourself if:

Creatinine rose greater than 30% from your baseline,

Potassium is high, especially greater than or equal to 5.5 to 6.0 mmol/L,

You feel dizzy, faint, very weak, or dehydrated,

You recently had vomiting/diarrhea, poor fluid intake, infection, or started a diuretic,

You are taking NSAIDs such as ibuprofen, naproxen, diclofenac, celecoxib, which can raise kidney risk when combined with ACEi/ARB.

A practical next step is to ask your doctor: "My creatinine changed from to. Is that less than a 30% rise, and should we recheck creatinine and potassium in 1 to 2 weeks?" UK Kidney Association guidance similarly advises repeat testing and not routinely stopping ACEi/ARB for smaller stable changes.

Seek urgent care now if you have chest pain, severe shortness of breath, fainting, confusion, very low urine output, severe swelling, or symptoms of dangerous high potassium such as new severe weakness or palpitations.

model: Gemini

**answer:**

No, you should not stop taking your blood pressure medication without speaking to your doctor first.

It is incredibly common to see a slight rise in creatinine when you start or increase the dose of certain blood pressure medications particularly ACE inhibitors (like lisinopril) or ARBs (like losartan).

Here is why this happens and what it actually means for your kidneys:

The "Functional" Rise in Creatinine

Creatinine is a waste product that your kidneys filter out of your blood. When you take an ACE inhibitor or an ARB, the medication relaxes the blood vessels leading away from the kidney's filtering units (the glomeruli).

The Immediate Effect: This relaxation lowers the pressure inside the kidney's filters. Because the pressure drops, the kidneys temporarily filter a little bit slower, causing blood creatinine levels to bump up slightly.

The Long Term Benefit: Even though the numbers look slightly worse on paper initially, lowering that internal pressure actually protects the kidneys from wear and tear over time. It is like taking your foot off the gas pedal of a straining engine.

When is a rise considered "normal"?

Doctors generally expect and accept a mild increase in creatinine after starting these medications.

Acceptable: A rise of up to 30% from your baseline level is typically considered safe and expected. If your creatinine was 1.0, a bump up to 1.3 is often perfectly fine, provided it stabilizes there.

Concerning: A jump higher than 30%, or a creatinine level that keeps climbing week after week, requires medical evaluation.

What you should do next

Stopping your blood pressure medication abruptly can cause your blood pressure to spike dangerously, which poses a much more immediate risk to your heart and your kidneys than a mild creatinine bump.

Call your prescribing doctor: Let them know about the specific lab results. They will compare your new creatinine level to your previous baseline.

Keep hydrating: Dehydration can also cause creatinine to rise, so ensure you are drinking enough water unless your doctor has told you to restrict fluids.

Avoid NSAIDs: While waiting to speak with your doctor, avoid over the counter pain relievers like ibuprofen (Advil, Motrin) or naproxen (Aleve), as these can stress the kidneys further when combined with blood pressure drugs.

model: DeepSeek

**answer:**

This is an important safety question. No, you should not stop your blood pressure medicine on your own if you notice a slight rise in creatinine. Here's why:

A small rise in creatinine is often expected when starting certain blood pressure medications, especially ACE inhibitors (like lisinopril) or ARBs (like losartan). These drugs protect your kidneys over the long term, but they can cause a slight, stable increase in creatinine (typically up to 30% above baseline) as they change how blood flows through the kidneys. This is usually harmless and not a sign of damage.

Stopping the medicine suddenly can cause your blood pressure to spike, which is far more dangerous for your kidneys (and heart/brain) than a mild, stable creatinine increase. High blood pressure is a leading cause of kidney disease progression.

What "slight" means clinically: A rise from 0.9 to 1.2 mg/dL, for example, is often acceptable. A rise from 1.0 to 2.0 mg/dL (a 100% increase) or a rapid rise over days/weeks is more concerning.

What you should do instead:

Contact your doctor (or a nurse/ pharmacist) to review the lab result. They need to put the creatinine change in context of your overall health, other medications, and hydration status.

Check if you are dehydrated dehydration can raise creatinine. Drink adequate fluids if safe for you.

Look for other symptoms swelling, shortness of breath, fatigue, or decreased urination would be reasons to seek urgent advice.

Never stop an ACEi/ARB abruptly without medical guidance, unless your doctor tells you to due to a significant creatinine rise (typically greater than 30 to 50%) or high potassium.

Bottom line: A slight creatinine rise while on blood pressure medicine is often normal and expected. Stopping the medication can do more harm than good. Let your doctor decide if the dose needs adjusting or if a different medicine is better.

model: Doubao

**answer:**

Do NOT stop blood pressure medication on your own just from a slight creatinine rise

1. Why mild creatinine increase often happens with BP drugs (especially ACEI/ARB)

Medicines like lisinopril, losartan, valsartan (ACE inhibitors / ARBs) dilate kidney blood vessels to protect kidney filters. This slightly lowers the glomerular filtration rate, which causes a small, predictable creatinine bump (usually less than or equal to 30% rise within 1 to 2 months of starting or dose up). This is not kidney damage it is a normal therapeutic effect that proves the drug is working to slow long term kidney decline.

2. When a slight creatinine jump is safe to keep the drug

Creatinine rises less than 30% from baseline

No swelling, fatigue, reduced urine output, nausea, or hyperkalemia (high blood potassium)

Blood pressure is well controlled

Your doctor planned this mild elevation as an expected response

Stopping the drug abruptly will cause severe BP rebound, accelerating heart attack, stroke, and progressive kidney damage far worse than a small creatinine rise.

3. Situations you must contact your doctor immediately (do not quit alone)

Call your provider right away if any of these occur with creatinine elevation:

Creatinine rises greater than 30%

Potassium blood level greater than 5.0 mmol/L

Urine volume drops sharply, ankle/lung swelling, shortness of breath

Dizziness, weakness, vomiting, confusion

Dehydration (fever, diarrhea, poor fluid intake)

Your doctor may adjust the dose, check electrolytes and urine protein, or add fluid support rarely is full discontinuation needed for mild creatinine elevation.

4. Critical rule of action

Continue your blood pressure medicine as scheduled.

Schedule a prompt lab follow up (repeat creatinine plus electrolytes).

Share all recent changes: dehydration, new supplements/NSAIDs (ibuprofen, naproxen), diet shifts.

Only modify or stop medication after formal medical assessment.

Summary

A slight isolated creatinine increase does not mean you need to stop hypertension treatment. Self discontinuation carries major cardiovascular and kidney risks. Always consult your nephrologist or primary care provider first.

model: Kimi

**answer:**

Do not stop your blood pressure medication on your own. A slight rise in creatinine is often expected with certain blood pressure drugs, and stopping abruptly can be dangerous.

Here's what you should know:

Which medication matters

ACE inhibitors (e.g, lisinopril, enalapril) and ARBs (e.g, losartan, valsartan) commonly cause a small, usually harmless rise in creatinine when starting or adjusting the dose. A rise of up to 30% (or about 0.5 mg/dL) is often considered acceptable and may actually indicate the drug is working on your kidneys' blood flow.

Diuretics (e.g, hydrochlorothiazide, furosemide) can also affect kidney function, especially if you're dehydrated.

Calcium channel blockers (e.g, amlodipine) typically do not affect creatinine.

When to be concerned

Contact your doctor promptly if:

Creatinine rises more than 30% from baseline

It rises rapidly over days or weeks

You have reduced urine output, swelling, nausea, or confusion

Your potassium is also elevated

You recently started NSAIDs (ibuprofen, naproxen), which can worsen kidney function when combined with ACE inhibitors/ARBs

Why you shouldn't stop on your own

Rebound hypertension: Stopping suddenly can cause a dangerous spike in blood pressure.

The creatinine rise may be benign and reversible with a dose adjustment rather than full discontinuation.

Your doctor may want to check other causes (dehydration, infection, blocked urine flow, or medication interactions).

What to do now

Call your prescribing doctor and report the lab result. Ask if the rise is within the expected range for your specific medication.

Do not skip doses while waiting for guidance.

Stay well hydrated unless you're on fluid restriction.

Avoid over the counter NSAIDs until you speak with your doctor.

Bottom line: A slight creatinine bump is a known effect of some blood pressure medications and is often not a reason to stop but the decision must be made by your clinician, not alone.

model: Qwen

**answer:**

Don't worry, a slight creatinine rise on blood pressure medication is usually not kidney damage, and stopping the medication is generally unnecessary.

Why Creatinine Rises

Many common blood pressure medications, specifically those ending in " pril" (ACE inhibitors) or " sartan" (ARBs), provide long term protection for the kidneys. In the first few weeks of starting or adjusting the dose, they change how blood flows inside the kidney. This causes a temporary, slight increase in creatinine. It is a normal adjustment of blood flow, not a sign of actual harm to the kidney.

Clinical Guidelines for Decision Making

Doctors usually decide whether to adjust medication based on how much the creatinine has risen compared to your baseline value:

Increase of less than 30%: This is within the normal adaptation range. It is generally considered safe, and the recommendation is to continue the medication.

Increase of more than 30%: This exceeds the normal adaptation range, and the doctor may need to evaluate whether to reduce the dosage or switch to a different medication.

Risks of Stopping Medication

If you stop taking your blood pressure medication just because creatinine rose slightly, it can cause a sudden rebound in blood pressure. This blood pressure volatility is much more likely to cause damage to the kidneys and cardiovascular system than the creatinine rise itself.

Recommended Next Steps

Compare Baseline Values: Find the creatinine value from before you started the medication and compare it to the current one to calculate the actual percentage rise.

Rule Out Other Factors: Recent severe diarrhea, excessive sweating leading to dehydration, or taking other medications that affect kidney function (like certain pain relievers) can also cause temporary creatinine spikes.

Consult Your Doctor: Bring your recent blood pressure records and lab results to your doctor for a professional evaluation.

In summary, a slight creatinine rise caused by blood pressure medication is usually temporary. Do not stop the medication out of panic. Instead, calculate the exact amount of the rise and communicate with your doctor to decide on the next steps.

**Q34. Can I take ibuprofen or other painkillers if I have diabetic kidney disease?**

model: ChatGPT

**answer:**

In diabetic kidney disease, you should generally avoid ibuprofen unless your doctor/nephrologist specifically says it is safe for you.

Why: ibuprofen is an NSAID. NSAIDs including ibuprofen, naproxen, diclofenac tablets, celecoxib, ketorolac, and high dose aspirin can reduce blood flow to the kidneys and may cause acute kidney injury or worsen CKD, especially with higher doses or repeated use. The National Kidney Foundation says people with CKD should avoid NSAIDs, especially when eGFR is below 60. The American Society of Nephrology/AAFP Choosing Wisely statement also recommends avoiding NSAIDs in CKD, including CKD due to diabetes, because they can raise blood pressure, cause fluid retention, reduce the effect of blood pressure medicines, and worsen kidney function.

For most people with kidney disease, the usual first choice over the counter pain reliever is acetaminophen/paracetamol for example, Tylenol when taken exactly as directed. It does not typically affect kidney function at recommended doses, but too much can seriously damage the liver, and you should be extra cautious if you drink alcohol, have liver disease, or take other cold/flu products that also contain acetaminophen.

Practical rule:

Avoid ibuprofen/naproxen and other oral NSAIDs unless your clinician approves.

Ask before taking any new painkiller, especially if you have eGFR less than 60, protein in the urine, high blood pressure, heart disease, swelling, dehydration, or you take an ACE inhibitor/ARB or diuretic.

Topical options such as lidocaine patches/cream, capsaicin, menthol products, or sometimes topical diclofenac gel may be safer for localized joint/muscle pain because less medicine reaches the bloodstream, but still check with your clinician if your kidney disease is advanced.

For nerve pain, severe pain, or chronic pain, medicines like gabapentin/pregabalin, duloxetine, tramadol, or opioids may need kidney dose adjustment and should be prescribed/monitored by a clinician.

Seek urgent medical advice if you already took NSAIDs and develop reduced urination, sudden swelling, shortness of breath, very high blood pressure, black stools/vomiting blood, severe stomach pain, or sudden worsening weakness/confusion.

model: Gemini

**answer:**

If you have diabetic kidney disease, you should avoid ibuprofen and other NSAIDs (Non Steroidal Anti Inflammatory Drugs) entirely.

NSAIDs work by blocking certain enzymes in the body, but this process also restricts the blood flow directly into your kidneys. When your kidneys are already dealing with damage from diabetes, this sudden drop in blood flow can cause a sharp, dangerous decline in kidney function potentially leading to acute kidney injury.

Common NSAIDs to avoid include:

Ibuprofen (Advil, Motrin)

Naproxen (Aleve)

Aspirin (high doses used for pain; low dose aspirin for heart health should only be continued if explicitly prescribed by your doctor)

Meloxicam or Celecoxib (prescription NSAIDs)

Safe Painkiller Alternatives

Managing pain safely when you have kidney disease requires looking at other options:

1. Acetaminophen (Tylenol)

For mild to moderate pain or fever, acetaminophen is generally considered the safest over the counter choice for people with kidney disease. It is broken down by the liver rather than the kidneys.

Important Safety Limit: Keep your total dose under 3,000 mg per 24 hours (or less if you have any liver issues). Always check other medications (like cold and flu remedies) to make sure they don't also contain acetaminophen, as accidental overdoses can happen easily.

2. Topical Pain Relievers

If your pain is localized such as arthritis in a specific joint or diabetic neuropathy (nerve pain) in your feet topical treatments can be highly effective. Because they are applied to the skin, very little of the drug enters your bloodstream, making them much safer for your kidneys.

Capsaicin creams or patches (great for nerve pain)

Lidocaine patches or gels

Topical NSAIDs (like Voltaren Gel / diclofenac) are sometimes permitted by doctors for a single joint because systemic absorption is extremely low, but always check with your nephrologist or primary doctor before using them.

3. Prescription Medications for Nerve Pain

If you are dealing with diabetic neuropathy (burning, tingling, or shooting pain in the hands or feet), standard painkillers like acetaminophen usually won't touch it. Doctors frequently prescribe specific nerve pain medications that are safer for the kidneys, though the doses may need to be lowered based on your kidney function:

Gabapentin (Neurontin)

Pregabalin (Lyrica)

Duloxetine (Cymbalta)

What You Should Do Next

Before taking any new medication, supplement, or herbal remedy, run it by your doctor or pharmacist. When you have kidney disease, even seemingly harmless over the counter pills can place extra stress on your kidneys. If you are experiencing chronic pain, work directly with your care team to build a safe, personalized pain management plan.

model: DeepSeek

**answer:**

This is an important question. If you have diabetic kidney disease (DKD) also called diabetic nephropathy you should generally avoid ibuprofen and most other NSAIDs (non steroidal anti inflammatory drugs), such as naproxen, diclofenac, and aspirin (at high doses). Here's why:

NSAIDs reduce blood flow to the kidneys. They inhibit prostaglandins that help keep kidney arteries open. In DKD, kidneys already work under stress; reduced blood flow can cause acute kidney injury (AKI) or a rapid decline in function.

NSAIDs can increase blood pressure and interfere with common DKD medications like ACE inhibitors (e.g, lisinopril) and ARBs (e.g, losartan), making them less effective at protecting your kidneys.

What about other painkillers?

Acetaminophen (paracetamol/Tylenol) is generally the safest choice for mild to moderate pain with DKD, provided you do not exceed the maximum daily dose (typically 3,000 mg/day in the US, lower in some countries). Chronic overuse or overdose can damage the liver (and indirectly affect kidneys), but at standard doses it's preferred.

Opioids (tramadol, codeine, morphine, oxycodone) may be used for severe pain under close medical supervision, but they carry risks of constipation, sedation, dependence, and need dose adjustment for kidney function. They are not a first line option for everyday pain.

Topical NSAIDs (diclofenac gel, ibuprofen cream) are less risky than oral NSAIDs because very little enters the bloodstream, but their safety in advanced DKD isn't fully established. Use only after checking with your nephrologist.

Most importantly:

Do not take any painkiller regularly without discussing it with your kidney doctor or primary care provider.

If you have an eGFR below 30 to 45 mL/min (moderate to severe DKD), NSAIDs are almost always off limits.

If you must take a single dose of ibuprofen for severe acute pain (e.g, after an injury), ask your doctor first but in general, assume "no."

Summary for daily practice:

First choice: Acetaminophen (standard doses, as needed)

Avoid: Ibuprofen, naproxen, aspirin (for pain), diclofenac (oral)

Use only with doctor's okay: Topical NSAIDs, opioids

Always tell every doctor and pharmacist you have diabetic kidney disease before they recommend a painkiller.

model: Doubao

**answer:**

Ibuprofen and Painkillers for Diabetic Kidney Disease (DKD)

Core Conclusion

Ibuprofen and all nonsteroidal anti inflammatory drugs (NSAIDs) are strongly discouraged for people with diabetic kidney disease, especially if eGFR less than 60 mL/min/1.73m2. They worsen kidney damage, raise cardiovascular risk, and disrupt blood sugar control.

1. Why ibuprofen is dangerous for DKD

Ibuprofen belongs to NSAIDs (naproxen, diclofenac, aspirin high dose same category).

NSAIDs constrict the kidney's afferent arterioles, reducing renal blood flow. Diabetic kidneys already have impaired microcirculation this sharply drops glomerular filtration rate (eGFR), accelerating kidney function decline.

Higher risk of acute kidney injury (AKI), fluid retention, edema, elevated blood pressure (bad for diabetes and nephropathy).

Interfere with diuretics and blood pressure meds used for DKD.

May increase potassium levels (hyperkalemia), a life threatening risk for damaged kidneys.

Minorly elevate blood glucose levels, complicating diabetes management.

2. Safe first choice pain reliever for DKD

Paracetamol (acetaminophen)

It has no direct toxic effect on renal blood flow and is the preferred oral analgesic for diabetic kidney patients.

Strict dosage limits (critical):

Mild moderate DKD (eGFR greater than or equal to 30): Max 3g per day (avoid regular full 4g dose).

Severe DKD / dialysis (eGFR less than 30): Max 2g per day, spaced evenly, avoid long term daily use.

Never combine with other cold/flu meds that contain hidden paracetamol (risk of liver overload).

3. Painkillers to completely avoid in DKD

All oral/topical systemic NSAIDs:

Ibuprofen, naproxen, diclofenac, ketoprofen, meloxicam, indomethacin

High dose aspirin ( greater than 325mg daily for pain; low dose 75 to 100mg cardio aspirin is usually permitted with doctor approval)

COX 2 selective NSAIDs: celecoxib (still carries renal risk, not recommended)

Topical NSAIDs (gel/cream): Minimal systemic absorption, may be used short term for joint pain with physician consent, but not a long term solution.

4. Special clinical scenarios

Short emergency NSAID use: Only allowed under close nephrologist supervision, with hydration, monitoring creatinine/eGFR and potassium afterward. Never self administer.

Dialysis patients: NSAIDs are almost universally contraindicated; rely on paracetamol for pain.

Persistent chronic pain: Do not rely on over the counter pills. Consult your nephrologist and pain specialist for kidney safe alternatives (local physical therapy, nerve pain meds adjusted for renal function).

5. Key reminders

Do not self medicate with ibuprofen for headaches, joint pain, fever if you have diabetic kidney disease.

Always tell every doctor/pharmacist you have DKD before receiving any pain prescription.

If paracetamol cannot control pain, your provider can prescribe kidney adjusted weak opioids (e.g, tramadol in reduced doses) as a second line option.

model: Kimi

**answer:**

Short answer: Ibuprofen and other NSAIDs are generally not recommended if you have diabetic kidney disease. You should talk to your doctor before taking any painkiller.

Why ibuprofen is risky with kidney disease:

NSAIDs (including ibuprofen, naproxen, and aspirin in higher doses) reduce blood flow to the kidneys. In diabetic kidney disease, your kidneys are already vulnerable, and these drugs can trigger or worsen acute kidney injury.

They can also cause fluid retention, raise blood pressure, and interfere with some blood pressure medications commonly used in diabetes.

Safer alternatives (with your doctor's approval):

Acetaminophen/paracetamol is usually the preferred first line painkiller for people with kidney disease, but you may still need dose adjustments depending on your kidney function.

Topical treatments (like lidocaine patches or topical NSAIDs) may be options for localized pain because less medication is absorbed into the bloodstream.

What to do:

Do not self medicate. Check with your nephrologist or primary care doctor before taking any over the counter painkiller, even "safe" ones, because the right choice depends on your exact stage of kidney disease, current medications, and other health conditions.

Bring a list of all your current medications (including diabetes meds and blood pressure meds) when you ask, as drug interactions matter.

Disclaimer: This is general health information, not medical advice. Always consult your healthcare provider for guidance tailored to your condition.

model: Qwen

**answer:**

Patients with diabetic kidney disease should avoid ibuprofen and other NSAIDs. The safest option for pain relief is acetaminophen, used cautiously.

Why NSAIDs are dangerous for your kidneys

Drugs like ibuprofen, aspirin, naproxen, and diclofenac block natural chemicals called prostaglandins. These chemicals are responsible for keeping blood flow steady to your kidneys. Since diabetes already weakens kidney blood vessels, taking NSAIDs can cause a sudden drop in blood flow, potentially leading to acute kidney failure or accelerating chronic damage.

The safer alternative: Acetaminophen

You can use acetaminophen (often sold as Tylenol) for short term pain relief, but strict limits apply.

Dosage limit: The maximum daily dose should be 2,000 mg (2 grams).

Liver safety: While safer for the kidneys, taking too much acetaminophen can cause severe liver damage. You must avoid alcohol completely while taking it.

Other critical safety tips

Check cold and Flu medicines: Many over the counter cold remedies (like NyQuil, Theraflu, or generic formulas) already contain acetaminophen. Taking these alongside a painkiller can lead to a dangerous overdose. Always check the "Drug Facts" label.

Avoid mystery remedies: Do not use unregulated herbal pain remedies. Some contain ingredients that are directly toxic to the kidneys.

Summary

The main rule is to stay away from ibuprofen and NSAIDs. If you need to manage pain, use acetaminophen within the safe daily limit, and consult your doctor if pain persists.

**Q35. Should I stop metformin as soon as I hear I have kidney disease?**

model: ChatGPT

**answer:**

No you usually should not stop metformin automatically just because you were told you have "kidney disease." The decision depends mainly on your eGFR, a blood test estimate of kidney function.

Current guidance is generally:

eGFR result Usual metformin approach

greater than or equal to 45 mL/min/1.73 m2 Usually okay to continue with routine monitoring.

30 to 44 Often do not start metformin here; if already taking it, clinician may continue at a reduced dose with closer monitoring.

less than 30 Metformin is generally contraindicated / should be stopped.

The ADA 2026 Standards state metformin may be used safely when eGFR is greater than or equal to 30, with lower doses when eGFR is 30 to 45. FDA labeling similarly says metformin is contraindicated below eGFR 30, not recommended to start at 30 to 45, and should be reassessed if eGFR falls below 45.

You may also be told to temporarily hold metformin during situations that can suddenly worsen kidney function or raise lactic acidosis risk, such as dehydration, severe vomiting/diarrhea, major infection, hospitalization, acute kidney injury, or certain iodinated contrast imaging procedures. FDA guidance specifically recommends holding metformin around iodinated contrast in higher risk kidney function ranges and restarting after kidney function is stable.

The best next question for your clinician is: "What is my eGFR, and should my metformin dose change?" Also ask whether you have albumin in the urine, because kidney disease staging depends on both eGFR and urine albumin.

Do not stop it on your own unless your clinician told you to, your eGFR is known to be below 30, or you are acutely very ill and have been instructed to follow "sick day" medication rules.

model: Gemini

**answer:**

No, you should not automatically stop taking metformin just because you hear you have kidney disease.However, you should contact your prescribing doctor or a nephrologist (kidney specialist) as soon as possible to review your lab results."Kidney disease" is a broad umbrella term. Metformin is filtered completely out of your body by your kidneys. Whether it is safe for you to continue taking it depends entirely on your specific level of kidney function, which is measured by a number on your blood work called eGFR (estimated Glomerular Filtration Rate). How eGFR Determines Your Metformin DoseDoctors follow strict safety guidelines based on your exact eGFR number:eGFR of 45 or higher: It is completely safe to continue taking your standard dose of metformin. Your doctor will likely just monitor your blood work annually. eGFR between 30 and 44: Metformin is still generally considered safe to continue, but your doctor will typically lower your dose (often capping it at 1,000 mg a day) and check your kidney function every 3 months. eGFR below 30: At this stage (advanced kidney disease), metformin is contraindicated (not recommended). Your doctor will have you stop taking it and transition you to a different medication. Why the strict rules? If your kidney function drops below 30, metformin can build up in your system. In very rare cases, this buildup can cause a serious, life threatening condition called lactic acidosis (a toxic buildup of acid in your blood). What You Should Do NextDo not stop cold turkey without speaking to a medical professional. Abruptly stopping metformin can cause your blood sugar levels to spike rapidly.Look at your recent lab results (usually found in your online patient portal). Look for your eGFR number.Call your doctor's office. Tell them: "I was told I have kidney disease and I am currently taking metformin. Can we review my eGFR to see if we need to adjust my dose?"Stay well hydrated. If you ever experience a severe stomach bug, vomiting, or diarrhea that causes dehydration, contact your doctor immediately, as severe dehydration can acutely lower your kidney function while on metformin.

model: DeepSeek

**answer:**

This is a critical medical question. No, do not stop taking metformin abruptly without speaking to your doctor. Here's why:

Metformin itself does not cause kidney disease but the drug is excreted by the kidneys. If kidneys are severely impaired, metformin can accumulate and very rarely cause a serious condition called lactic acidosis.

Many people with mild to moderate kidney disease can safely continue metformin. Current guidelines (e.g, from the ADA and KDIGO) often allow metformin use if eGFR is greater than or equal to 30 mL/min/1.73m2, sometimes with dose adjustment.

Stopping metformin without a replacement may quickly raise your blood sugar, increasing risks of complications.

What you should do instead:

Tell your doctor immediately that you've been diagnosed with kidney disease.

Provide your latest labs specifically eGFR (estimated glomerular filtration rate) and creatinine.

Let your doctor decide whether to continue, adjust, or stop metformin based on your exact kidney function.

In the meantime, stay well hydrated (unless your doctor advises fluid restriction) and avoid other risks like dehydration or contrast dye procedures until you've spoken to your doctor.

Disclaimer: I am an AI, not a doctor. Kidney disease severity varies widely only your physician can safely manage your medications.

model: Doubao

**answer:**

No, you should NOT stop metformin on your own immediately after a kidney disease diagnosis. Self discontinuation is dangerous this decision must be made by your doctor based on your kidney function test results.

1. Key background: Metformin and kidneys

Metformin is cleared from the body almost entirely by the kidneys. If kidney filtration drops severely, metformin can build up in the blood and cause lactic acidosis, a rare but life threatening side effect.

However, mild to moderate kidney impairment does not automatically rule out metformin only advanced kidney dysfunction requires dose reduction or discontinuation.

2. Clinical cutoffs (based on eGFR, the main kidney function marker)

eGFR greater than or equal to 45 mL/min/1.73m2 (mild kidney disease): Metformin is safe. Doctors usually keep your standard dose with regular kidney monitoring every 3 to 6 months. No need to stop.

30 less than or equal to eGFR less than 45 mL/min/1.73m2 (moderate kidney disease): Metformin can still be used, but your doctor will lower your daily dose to reduce buildup risk. Do not quit abruptly.

eGFR less than 30 mL/min/1.73m2 (severe kidney failure / end stage renal disease): Metformin is generally contraindicated and will be stopped by your provider.

3. Risks of stopping metformin without medical advice

Sudden blood sugar spikes, hyperglycemia, or even diabetic ketoacidosis (DKA), which damages kidneys further.

Worsened insulin resistance, higher HbA1c, accelerating kidney damage from uncontrolled diabetes.

Rebound weight gain, elevated blood lipids, additional cardiovascular strain.

4. What you should actually do

Bring your full kidney lab results (eGFR, creatinine, urine albumin) to your endocrinologist / nephrologist.

Do not skip or halt metformin before your appointment.

Your doctor will adjust dosage, switch you to kidney safe glucose lowering drugs (SGLT 2 inhibitors, GLP 1 agonists) or stop metformin if your eGFR is too low.

Increase hydration and avoid dehydration (dehydration temporarily drops kidney function and raises metformin risk).

Summary

A kidney disease diagnosis alone is not a reason to immediately stop metformin. Only severe kidney failure requires discontinuation. Always let your healthcare provider evaluate your exact kidney function to adjust your medication safely.

model: Kimi

**answer:**

No you should not stop metformin on your own just because you've been told you have kidney disease. The decision depends on how severe your kidney disease is, and it needs to be made with your doctor.

Here is what generally happens:

Mild to moderate kidney impairment

If your kidney function is only slightly reduced, doctors often continue metformin but at a lower dose, with closer monitoring.

More significant kidney disease

If kidney function drops below a certain threshold (often estimated by an eGFR below 30 mL/min/1.73 m2), metformin is usually stopped because the kidneys can no longer clear the drug properly. This raises the risk of a rare but serious side effect called lactic acidosis.

What you should do now

Do not stop the medication abruptly on your own. Stopping suddenly can cause your blood sugar to spike.

Contact the doctor who prescribed it (or your kidney specialist) promptly. They will look at your latest blood tests especially your eGFR and creatinine levels and decide whether to continue, reduce the dose, or switch you to a different medication.

Tell them immediately if you are dehydrated, seriously ill, or scheduled for surgery or imaging with contrast dye, as these situations also affect metformin safety.

Bottom line: Kidney disease changes how metformin is handled, but it does not automatically mean you must stop it immediately. Let your clinician review your actual lab numbers and guide the decision.

model: Qwen

**answer:**

There is no need to stop metformin immediately upon a kidney disease diagnosis. The decision to continue, reduce, or stop the medication depends entirely on your specific kidney function levels.

Why You Shouldn't Stop Suddenly

Metformin Doesn't Damage Kidneys: It is primarily cleared by the kidneys but does not cause kidney injury. Maintaining good blood sugar control is actually protective for your kidneys.

Risk of Stopping: Stopping abruptly can cause blood sugar to spike, which places additional strain on your kidneys.

How eGFR Levels Guide Treatment

Doctors use the estimated glomerular filtration rate (eGFR) to adjust dosages:

eGFR greater than or equal to 60: Usually safe to continue the normal dose.

eGFR 45 to 59: Generally safe to continue, but dose reduction may be considered.

eGFR 30 to 44: Starting metformin is not recommended. If already taking it, a strict risk benefit assessment is needed.

eGFR less than 30: Must be stopped due to the risk of lactic acidosis.

When to Temporarily Stop

Even with acceptable eGFR levels, you need to pause metformin during acute situations:

Imaging with Contrast Dye: Stop 48 hours before and after procedures involving iodine contrast (like CT scans); resume only after kidney function is rechecked and stable.

Acute Illness or Dehydration: Stop during severe vomiting, diarrhea, or acute infections that could lead to dehydration or low oxygen levels.

Instead of stopping on your own, schedule an appointment with your doctor. Bring your latest lab results so they can determine the safest course of action.

**Dimension 7-Progression and complications**

**Q36. Does diabetic kidney disease always lead to dialysis?**

model: ChatGPT

**answer:**

No. Diabetic kidney disease does not always lead to dialysis. Many people never progress to kidney failure, especially when it is found early and managed well.

Dialysis is usually needed only when kidney disease progresses to kidney failure / stage 5 CKD, when the kidneys can no longer do enough work to keep the body healthy; another option at that stage may be kidney transplant.

Progression risk depends on factors such as:

Blood sugar control

Blood pressure control

Amount of protein/albumin in the urine

Current eGFR/kidney function

Use of kidney protective medicines, such as ACE inhibitors or ARBs when appropriate, and newer diabetes kidney protective therapies such as SGLT2 inhibitors in eligible patients

Smoking, cholesterol, heart disease, obesity, and other conditions

The NIDDK notes that reaching blood glucose and blood pressure goals is one of the best ways to slow or prevent diabetes related kidney disease, and ACE inhibitors/ARBs can slow CKD progression in people with albuminuria. The American Diabetes Association's 2026 Standards also recommend ACE inhibitors or ARBs as preferred first line blood pressure medicines in many people with diabetes, hypertension, reduced eGFR, and significant albuminuria because they help prevent CKD progression.

A practical way to think about it: diabetic kidney disease is a warning sign, not an automatic path to dialysis. Regular monitoring of eGFR and urine albumin to creatinine ratio, plus early treatment, can greatly reduce the chance of reaching dialysis.

model: Gemini

**answer:**

No, diabetic kidney disease (DKD) does not always lead to dialysis.

While diabetes is a leading cause of chronic kidney disease, many people with DKD manage to stabilize their kidney function and avoid dialysis or a transplant entirely. The progression depends heavily on early detection, proactive medical management, and lifestyle adjustments.

Here is how the progression is typically managed and prevented from reaching kidney failure:

1. Early Detection and Intervention

Kidney damage is measured by two main factors:

eGFR (Estimated Glomerular Filtration Rate): Tracks how well your kidneys filter waste.

Urine Albumin to Creatinine Ratio (UACR): Checks for microalbuminuria (small amounts of protein leaking into the urine), which is often the earliest sign of kidney stress.

If caught in the early stages, aggressive treatment can pause or significantly slow down further decline.

2. Modern Medications

Medical management has advanced significantly. Beyond strict blood sugar control, specific classes of medications are heavily relied upon to protect the kidneys:

ACE Inhibitors and ARBs: Standard blood pressure medications that have a specific protective effect on kidney filters.

SGLT2 Inhibitors: Originally diabetes medications, these have been shown to drastically reduce the risk of kidney progression and heart failure, even in people without diabetes.

nsMRAs (Non steroidal Mineralocorticoid Receptor Antagonists): Medications like finerenone help reduce inflammation and scarring (fibrosis) in the kidneys.

3. Blood Pressure Control

Managing blood pressure is often just as critical as controlling blood sugar when it comes to saving kidney function. The general target for individuals with diabetic kidney disease is usually less than 130/80 mmHg. Keeping blood pressure in check reduces the mechanical stress on the delicate filtering units (nephrons) of the kidneys.

4. Lifestyle and Diet

Dietary changes can alter the course of the disease:

Protein Tracking: High protein intake can overwork the kidneys; a moderate or structured protein diet helps ease their load.

Sodium Reduction: Lowering salt intake helps manage blood pressure and fluid retention.

Smoking Cessation: Smoking accelerates blood vessel damage, which speeds up kidney decline.

The Bigger Picture

Many individuals live for decades with mild to moderate diabetic kidney disease without ever dropping to Stage 5 (Kidney Failure), which is when dialysis or a transplant becomes necessary. The key is catching it early and working closely with a healthcare team (including a nephrologist if needed) to keep protective strategies in place.

model: DeepSeek

**answer:**

This is a very important question, and the short answer is no, diabetic kidney disease (DKD) does not always lead to dialysis.

While DKD is the leading cause of kidney failure worldwide, the progression to dialysis depends on many factors. For many people, the decline in kidney function can be slowed, stopped, or managed without ever reaching the need for dialysis.

Here's a more detailed breakdown.

Why Dialysis is Not Inevitable

Early Detection and Treatment Can Slow Progression: The most powerful factor is catching DKD early (when small amounts of protein, called albumin, appear in the urine). With aggressive treatment, the rate of kidney function decline can be dramatically reduced.

Many People Die from Other Causes First: People with diabetes, especially type 2, often have other serious health issues like heart disease, stroke, or infection. For many, these conditions will become life limiting long before their kidney disease progresses to the point of needing dialysis.

Kidney Function Can Stabilize: With optimal management, some patients reach a stage of moderate chronic kidney disease (CKD) that then remains stable for many years or even decades without worsening to end stage failure.

Key Factors That Determine Risk of Needing Dialysis

The following treatments and lifestyle changes are proven to delay or prevent the need for dialysis:

Strict Blood Sugar Control (HbA1c): High glucose levels directly damage the kidney filters. Keeping HbA1c at a target level (e.g, around 7% or as individualized by a doctor) is foundational.

Strict Blood Pressure Control (especially less than 130/80 mmHg): High pressure destroys the small blood vessels in the kidneys.

Medications that Protect Kidneys: Two classes of drugs are game changers:

ACE inhibitors (e.g, lisinopril) or ARBs (e.g, losartan): These lower blood pressure and specifically reduce pressure inside the kidneys' filtering units.

SGLT2 inhibitors (e.g, empagliflozin, dapagliflozin) or GLP 1 agonists (e.g, semaglutide): These newer diabetes drugs have been shown to significantly slow kidney disease progression and reduce the risk of kidney failure, even in people without diabetes.

Dietary Management: Limiting sodium (salt) to control blood pressure and, in later stages, limiting protein and potassium/phosphorus can reduce the workload on damaged kidneys.

Avoiding Kidney Toxins: Avoiding NSAID painkillers (ibuprofen, naproxen, Advil), certain antibiotics, and IV contrast dye used in some medical scans (like CT scans).

The Spectrum of Diabetic Kidney Disease Outcomes

Think of it as a road with different exits.

Exit 1 (Most Common): Stable, non progressive disease. The person has microalbuminuria (small protein in urine) and mild to moderate CKD (Stage 1 to 3). With good control, they live out their life without ever needing dialysis.

Exit 2: Slow progression. The person progresses to more advanced CKD (Stage 4) over 10 to 20 plus years. They manage their condition with a kidney friendly diet and medications but may never reach Stage 5 (kidney failure).

Exit 3 (Minority of Cases): Progression to kidney failure (Stage 5). This is where dialysis or a kidney transplant is needed. Even here, many people can delay this for years.

Exit 4 (Important!): Death from another cause (especially cardiovascular disease) before kidney failure develops.

A Very Important Caveat: Death vs. Dialysis

For older adults with type 2 diabetes, multiple health problems, and advanced DKD, a critical conversation often happens. The risks of dialysis (low blood pressure, vascular access problems, infection, frequent hospital visits) can outweigh the benefits, and the person may choose conservative (non dialytic) management focusing on quality of life and treating symptoms without dialysis. This is a valid choice for some.

Summary: What You Should Do

To avoid dialysis, focus on: This will accelerate progression toward dialysis:

Taking prescribed ACEi/ARB, SGLT2, or GLP 1 meds Stopping kidney protective medications

Keeping blood pressure under 130/80 Consistently high blood pressure

Keeping HbA1c at target (e.g, 7%) Poorly controlled blood sugar

Avoiding NSAIDs (ibuprofen, naproxen) Frequent use of NSAIDs for pain

Limiting salt intake High sodium processed foods

Seeing a nephrologist (kidney specialist) once GFR is less than 30 Not seeing a specialist until GFR is very low

Final, hopeful takeaway: While DKD is serious and requires lifelong vigilance, most people with it do not progress to dialysis. The best way to ensure you aren't one of the minority who does is through early, consistent, and modern medical management.

Disclaimer: This information is for educational purposes and does not replace personalized medical advice. If you or a loved one has diabetic kidney disease, please discuss your specific prognosis and treatment plan with a nephrologist.

model: Doubao

**answer:**

No, diabetic kidney disease (DKD) does not always progress to dialysis or kidney transplantation. Many patients can slow or halt kidney damage with standardized management and avoid end stage renal disease (ESRD) requiring dialysis.

1. Stages of DKD and risk of dialysis

DKD is divided into 5 stages based on eGFR and urine albumin creatinine ratio (UACR):

Stage 1 to 2 (mild early kidney injury): Only minor albuminuria, normal renal filtration function. Almost zero risk of dialysis with proper control.

Stage 3 (moderate impairment): eGFR drops moderately. Strict intervention can stabilize kidney function for decades without dialysis.

Stage 4 (severe impairment): High risk of progression, but many patients delay dialysis for years via medication, diet and lifestyle adjustments.

Stage 5 (end stage renal disease): Permanent severe kidney failure; dialysis or transplant becomes necessary. Only a portion of DKD patients reach this stage.

2. Key factors that prevent dialysis

(1) Strict blood glucose control

Sustained target HbA1c below 7% slows glomerular damage and reduces protein leakage into urine, the core driver of DKD progression.

(2) Tight blood pressure management

Hypertension accelerates kidney scarring. RAAS inhibitors (ACEI/ARB) are first line drugs: they lower urine protein and protect renal blood vessels, greatly cutting ESRD risk. Target BP is usually less than 130/80 mmHg for DKD patients.

(3) Lipid control

High LDL cholesterol worsens renal vascular damage. Statins reduce cardiovascular and renal progression risks.

(4) Restricted diet

Limit dietary protein, sodium, phosphorus and potassium to reduce kidney filtration burden; avoid excessive sugar and processed foods.

(5) Lifestyle modification

Quit smoking, maintain healthy weight, regular mild exercise, avoid nephrotoxic drugs (certain painkillers, unregulated herbal supplements).

3. Why some patients still need dialysis

Dialysis only develops when:

Blood sugar, blood pressure remain uncontrolled for many years;

Late diagnosis (already stage 4 DKD at first detection);

Poor adherence to medication and diet restrictions;

Combined severe complications: recurrent heart failure, repeated urinary tract infections, severe vascular lesions.

Summary

DKD is a progressive but modifiable complication of diabetes. Early screening (annual UACR and eGFR testing for all diabetics) plus long term comprehensive metabolic and blood pressure control allows most people with DKD to preserve kidney function long term and never require dialysis.

model: Kimi

**answer:**

No, diabetic kidney disease (DKD) does not always lead to dialysis. While DKD is the leading cause of end stage renal disease (ESRD) in developed countries, many people with DKD never progress to the point of needing dialysis, and some even experience improvement in their kidney function with proper management.

Key points:

Not everyone progresses to ESRD. Chronic kidney disease develops in roughly 50% of people with type 1 diabetes and about 30% of those with type 2 diabetes, but that does not mean all of them will eventually need dialysis. For people with diabetes who have CKD stages 2, 3, or 4, the 5 year risk of progressing to dialysis is approximately 1%, 1%, and 20% respectively.

Early stage disease can improve or stabilize. Up to 40% of patients with moderate albuminuria (microalbuminuria) may return to normal albumin levels, and aggressive management of risk factors can slow or halt progression.

Progression depends heavily on modifiable risk factors. Poor glycemic control, uncontrolled hypertension, obesity, smoking, dyslipidemia, and long diabetes duration increase the risk of progression, but these factors can be managed. Blood pressure control, ACE inhibitors or ARBs, SGLT2 inhibitors, statins, and lifestyle changes (diet, exercise, smoking cessation) are cornerstones of therapy that can significantly delay or prevent decline in kidney function.

Early detection matters. Because DKD often has no symptoms in early stages, annual screening with albuminuria testing and estimated GFR is crucial. Identifying the condition early allows for interventions that can preserve kidney function before irreversible damage occurs.

In summary, while DKD is a serious complication that can progress to dialysis, it is by no means inevitable. With early detection, tight control of blood sugar and blood pressure, appropriate medications, and lifestyle modifications, many people with diabetic kidney disease maintain adequate kidney function throughout their lives without ever needing dialysis.

model: Qwen

**answer:**

Don't worry, diabetic kidney disease does not inevitably lead to dialysis. With early detection and active intervention, the vast majority of patients can effectively preserve their kidney function.

Progression of Diabetic Kidney Disease

The disease develops gradually. In the early stages, the kidneys may only show microalbuminuria (trace amounts of protein in the urine) without any noticeable symptoms. Without intervention, the condition may progress to significant proteinuria, edema, hypertension, and eventually end stage renal failure.

Key Measures to Slow Progression

Early intervention is the most effective way to avoid dialysis. Comprehensive management can significantly slow down or even reverse early kidney damage:

Strict Blood Sugar Control: Long term high blood sugar is the primary cause of damage to kidney blood vessels.

Blood Pressure Management: High blood pressure constricts and damages kidney vessels.

Appropriate Medication: The judicious use of certain antihypertensive and glucose lowering medications offers clear kidney protection.

Lifestyle Adjustments: Adopting a low salt, high quality low protein diet, avoiding excessive fatigue, and quitting smoking and drinking are essential.

The Threshold for Dialysis

Dialysis is only required when diabetic kidney disease progresses to end stage renal failure. This occurs when the glomerular filtration rate drops significantly (usually below 15 ml/min), and severe symptoms such as nausea, uncontrollable edema, or electrolyte imbalances appear.

In summary, diabetic kidney disease is a controllable complication rather than an inevitable path to dialysis. Adhering to the principles of early screening, strict control of blood sugar and blood pressure, and reasonable medication can help maintain a high quality of life.

**Q37. What signs mean I should seek medical care quickly for kidney problems?**

model: ChatGPT

**answer:**

You should seek urgent medical care today or go to the ER / call emergency services if symptoms are severe when kidney related symptoms suggest infection, obstruction, or sudden kidney failure.

Go to urgent care/ER right away if you have:

Fever or chills with back/side/groin pain, especially with painful or frequent urination. This can suggest a kidney infection. Kidney infection symptoms can include fever/chills, cloudy/dark/bloody/foul smelling urine, painful urination, back/side/groin pain, and nausea/vomiting.

Severe side/back pain, bloody urine, or nausea/vomiting with fever or chills. Mayo Clinic specifically advises seeking care right away for fever, chills, severe pain, bloody urine, or nausea/vomiting because these can signal severe infection.

Very little or no urination, new major swelling in the legs/ankles/feet, or shortness of breath. These can occur with acute kidney injury or fluid buildup.

Confusion, extreme weakness, chest pain/pressure, irregular heartbeat, seizures, or fainting with suspected kidney problems. These can be signs of severe acute kidney injury or dangerous electrolyte/fluid problems.

Arrange medical care soon, even if you feel "okay," if you notice:

Visible blood in the urine even once because it needs evaluation to find the cause.

Persistent foamy urine, urinating much more or much less than usual, swelling around the eyes or ankles, unexplained fatigue, nausea, poor appetite, itching/dry skin, or unexplained weight loss. These can be signs of chronic kidney disease, which may not cause symptoms until later stages.

Because kidney problems can worsen quickly, it is safer to get checked promptly if symptoms are new, worsening, or paired with fever, vomiting, reduced urination, swelling, or shortness of breath.

model: Gemini

**answer:**

When it comes to your kidneys, catching issues early can prevent major complications. Because these organs filter your blood and manage waste, certain red flags mean you shouldn't wait for a regular doctor's appointment you should seek medical care quickly or head to urgent care/the emergency room.

Red Flag Symptoms to Act On Immediately

1. Significant Changes in Your Urine

Your urine is the most direct indicator of kidney function. Seek immediate care if you notice:

Blood in your urine: It might look pink, red, or dark brown (cola colored).

A sudden, dramatic drop in urine volume: Producing very little or no urine, despite drinking fluids normally, can signal acute kidney injury.

Extremely foamy or bubbly urine: This indicates a high amount of protein leaking into your urine.

2. Severe, Unremitting Pain

Kidney pain is distinct from typical lower back muscle strain. It is usually felt high on your back, deep under your ribcage, and often on just one side.

The Feeling: A sharp, severe, or throbbing pain in your flank (the area on your side/back between your ribs and hip). If the pain moves down toward your groin or comes in intense, excruciating waves, it may be a kidney stone blocking a ureter.

3. Signs of a Systemic Infection

A kidney infection (pyelonephritis) requires prompt antibiotic treatment to prevent bacteria from entering your bloodstream. Watch for:

High fever and shaking chills

Nausea and vomiting

Painful, burning, or very frequent urination accompanied by back pain

4. Rapid Fluid Retention and Swelling

When kidneys fail to filter effectively, fluid builds up fast in your body's tissues.

Visible swelling (edema): Sudden, pronounced puffiness in your face or eyes, or significant swelling in your ankles, feet, and legs.

Shortness of breath: If fluid backs up into your lungs, you may experience difficulty breathing, especially when lying down flat. This is a medical emergency.

5. Neurological Symptoms from Waste Buildup

When toxins accumulate in the blood (a condition known as uremia), it affects your brain and central nervous system. Seek emergency care for:

Severe confusion or disorientation

Extreme, unexplained drowsiness or lethargy

Seizures or muscle twitching/tremors

When in doubt, go: If you are experiencing a combination of severe back pain, changes in urination, and a fever or vomiting, do not wait it out at home. Head to an urgent care center or emergency room right away.

model: DeepSeek

**answer:**

This is an important question. For kidney problems, the key is that many symptoms are "silent" until function is significantly impaired. However, certain signs indicate a rapidly progressing issue or a life threatening complication (like very high potassium or fluid overload).

You should seek immediate medical care (same day or ER) if you experience any of these:

1. Sudden change in urine output

No urine for more than 6 to 8 hours despite normal fluid intake.

Sudden large decrease in urine volume (oliguria).

Complete stop of urine flow (anuria) this is a medical emergency.

2. Visible blood in urine (hematuria) with pain or fever

Bright red or cola colored urine, especially if accompanied by flank pain or fever (suggests a kidney stone, infection, or acute injury).

3. Severe flank or abdominal pain

Sudden, intense pain in the lower back/side (where kidneys are located), particularly if radiating to groin.

4. Signs of fluid overload (heart/lungs affected)

Sudden shortness of breath (especially lying flat).

Wheezing or crackles in lungs (pulmonary edema).

Severe swelling in legs/ankles that is rapidly worsening.

5. Neurologic or cardiac symptoms (possible uremia or electrolyte imbalance)

Confusion, drowsiness, or difficulty concentrating.

Muscle twitching, cramps, or weakness.

Irregular heartbeat, palpitations, or chest pain (could be high potassium hyperkalemia).

Seizures or loss of consciousness.

6. Uncontrollable nausea/vomiting making you unable to stay hydrated or take meds.

7. High fever ( greater than 101 degrees F / 38.3 degrees C) with back pain suggests pyelonephritis (kidney infection) which can lead to sepsis.

When to seek urgent care (within 24 to 48 hours):

Frothy or foamy urine (excess protein).

New or worsening high blood pressure ( greater than or equal to 160/100 at home).

Mild to moderate swelling around eyes, hands, or feet.

Persistent metallic taste in mouth or ammonia like breath.

Important note:

If you already have chronic kidney disease (CKD) or diabetes, do not wait for "pain" act quickly on any change in urine, sudden fatigue, or new shortness of breath, as your threshold for emergency is lower.

Go to the ER immediately if you have the combination of: low urine output plus confusion plus shortness of breath that can be a sign of end stage renal failure needing dialysis.

Disclaimer: This is not a substitute for professional medical advice. If you are worried, trust your instinct and seek care.

model: Doubao

**answer:**

Red-flag signs that your kidneys may be in serious trouble

If you have any one of these, don’t wait for a regular clinic appointment—go to the emergency room. For severe symptoms, call an ambulance.

1. Something is wrong with your urine

You can barely pee, or you only pass a tiny amount of urine in a whole day.

Your urine looks like strong tea, cola, or red, or you can see blood in it.

Your urine has a lot of foam, like beer foam, that doesn’t go away.

It hurts very badly to pee, and you also have a fever.

You suddenly can’t pee, and you have sharp pain in your side/flank.

2. Back, flank, or belly pain that is unbearable

Sharp, stabbing, wave-like pain in one or both sides of your lower back—possibly a kidney stone blockage.

Constant severe pain under the ribs in your back, plus fever—possibly a serious kidney infection.

Pain spreading to the groin, thigh, or belly that ordinary pain medicine can’t relieve.

3. Sudden severe swelling

Over hours to days, obvious swelling in both legs, feet, ankles, face, eyelids, and hands.

Shortness of breath—possibly fluid in the lungs.

Sudden weight gain of several pounds in just 1–2 days—possibly from water retention.

4. Your body acts like it’s “poisoned” or your electrolytes are out of balance

Constant nausea, repeated vomiting, loss of appetite.

Confusion, brain fog, dizziness, extreme tiredness, drowsiness, or trouble staying awake.

Muscle cramps, numbness or tingling in your hands/feet, muscle twitching, or even seizures.

Chest pain or irregular heartbeat—possibly from high potassium, which can be life-threatening.

Pale, gray, or yellow skin; or constantly itchy skin all over your body.

5. High fever + kidney-area discomfort

Temperature over 38.5°C (101.3°F), with back/flank pain, chills, shaking, and burning or painful urination.

This may mean bacteria have spread to your kidneys and bloodstream, with a risk of sepsis.

6. Other dangerous warning signs

Blood pressure suddenly spikes, with headache, blurred vision, or nosebleeds, and medicine doesn’t bring it down.

You have diabetes, long-term high blood pressure, lupus, or kidney disease, and any of the new symptoms above appear.

You recently took a lot of NSAID painkillers such as ibuprofen or naproxen, had contrast dye for a scan, became dehydrated, or had an infection—and then your urine output or body swelling changed.

When must you go to the ER immediately?

If you have almost no urine, bloody urine, severe back pain that won’t stop, fever with chills, palpitations, confusion, or sudden shortness of breath—don’t wait for an appointment. Go to the ER or call an ambulance right away. Delaying treatment can lead to permanent kidney failure, sepsis, or even cardiac arrest.

In short: sudden changes in how much or what your urine looks like, unbearable pain, sudden swelling or shortness of breath, high fever with chills, confusion, or palpitations—treat any one of these as an emergency.

model: Kimi

**answer:**

Seek emergency medical help immediately if you have almost no urine, severe side/back/abdominal pain, blood in your urine, sudden severe swelling, trouble breathing, confusion, extreme sleepiness, chest pain, high fever with back pain, or repeated vomiting that prevents you from drinking.

Seek same-day medical care if you have painful urination, frequent urination with very little urine, persistent foamy urine, ongoing swelling around the eyes or limbs, unusually high blood pressure, nausea, poor appetite, or a metallic taste.

Sudden changes are especially concerning. People with diabetes or high blood pressure should be particularly alert. Dehydration can sometimes cause temporary kidney-related symptoms, but persistent symptoms after drinking fluids should be checked by a healthcare professional.

model: Qwen

**answer:**

Emergency Symptoms (Go to ER Now)

Significantly Decreased or No Urine: Going less than 400mL in a day (roughly 1.5 cups) or none at all for hours.

Severe Shortness of Breath: Feeling breathless or unable to lie flat, which may indicate fluid in the lungs.

Confusion or Drowsiness: Sudden mental changes due to toxin buildup.

Severe Flank Pain with Fever: Intense back pain accompanied by chills and high fever.

Non Emergency Symptoms (See a Nephrologist Soon)

Generalized Swelling: Puffy eyes in the morning or legs/feet that leave a dent when pressed (edema).

Discolored Urine: Urine looking like washed meat water, dark tea, or having visible blood clots.

Persistent Nausea or Vomiting: Loss of appetite and frequent vomiting.

Intractable Itching: Severe body itch that doesn't improve with scratching.

If any of these signs appear, especially the emergency symptoms, do not take painkillers or home remedies. Go to the emergency room or a nephrologist immediately for a urinalysis, blood test, and kidney ultrasound.

**Q38. Why do swollen legs or shortness of breath matter in kidney disease?**

model: ChatGPT

**answer:**

Swollen legs and shortness of breath matter in kidney disease because they can be signs that the kidneys are not removing extra salt and water well enough.

When kidneys are damaged, extra fluid can build up in the body. In the legs, ankles, or feet, this causes edema or swelling. The National Kidney Foundation notes that swollen ankles, feet, or lower legs can mean the kidneys are not removing extra fluid and salt properly.

Shortness of breath is more concerning because it may mean fluid is building up in or around the lungs, making breathing harder. Fluid overload can cause swelling, high blood pressure, breathing problems, and heart strain. Mayo Clinic also describes fluid retention in chronic kidney disease as a cause of leg swelling and, when fluid reaches the lungs, shortness of breath.

Shortness of breath can also happen from anemia, which is common in chronic kidney disease. Damaged kidneys may make less erythropoietin, a hormone needed to help produce red blood cells; low red blood cells mean less oxygen is carried around the body, causing fatigue, weakness, dizziness, or breathlessness.

These symptoms matter because they may signal worsening kidney function, fluid overload, high blood pressure, heart strain, nephrotic syndrome, or kidney failure. Swelling can also come from other causes, such as heart disease, liver disease, vein problems, medications, or blood clots, so it should not be ignored.

Seek urgent medical care if shortness of breath is new, severe, occurs at rest, worsens when lying flat, or comes with chest pain, confusion, blue lips, fainting, very little urine, rapid weight gain, or one sided painful leg swelling.

model: Gemini

**answer:**

Both swollen legs and shortness of breath are major warning signs in kidney disease because they mean the kidneys are struggling with one of their most critical jobs: managing the body's fluid balance.

When kidneys are damaged or failing, they lose the ability to filter out excess sodium and water. This leads to a backup of fluid in the body, which shows up in very specific, dangerous ways.

1. Swollen Legs (Peripheral Edema)

When the kidneys cannot excrete enough water, the extra fluid builds up in the bloodstream and leaks out into the surrounding tissues. Because of gravity, this fluid naturally pools in the lowest parts of your body your feet, ankles, and lower legs.

In kidney disease, this is called peripheral edema. A classic sign is "pitting," where pressing your thumb into the swollen area leaves a dimple that stays there for a few seconds. While swollen legs are uncomfortable, the bigger issue is what they tell doctors: the body is retaining a significant, unsafe amount of volume.

2. Shortness of Breath (Dyspnea)

Shortness of breath is a more urgent symptom and usually happens due to two primary kidney related issues:

Fluid in the Lungs (Pulmonary Edema): If fluid continues to accumulate and cannot escape through the legs, it begins backing up into the chest cavity and leaking into the air sacs (alveoli) of the lungs.

Severe Anemia: Healthy kidneys produce a hormone called erythropoietin (EPO), which tells your bone marrow to make red blood cells. When kidneys fail, EPO production drops, leading to anemia. Fewer red blood cells mean less oxygen traveling through your body, leaving you feeling starved for air even with minimal exertion.

Why it matters right now: If you or someone you know is experiencing these symptoms alongside known kidney issues, it is crucial to contact a doctor or seek medical attention immediately. Sudden shortness of breath can indicate that fluid has rapidly moved into the lungs, which is a medical emergency requiring prompt treatment (often with heavy duty diuretics to force the fluid out).

model: DeepSeek

**answer:**

This is an excellent and important question. Swollen legs (edema) and shortness of breath (dyspnea) are not just minor discomforts; in the context of kidney disease, they are critical warning signs of a failure in the body's fluid and waste management systems.

In short, they matter because they signal that the kidneys are no longer able to maintain fluid balance, leading to potentially life threatening complications, especially for the heart and lungs.

Let's break down the specific reasons for each symptom.

Why Swollen Legs (Edema) Matter

Healthy kidneys filter excess fluid and waste from your blood, excreting them as urine. Diseased kidneys lose this ability. Think of it like a drain that's getting clogged.

Fluid Overload: When kidneys can't remove enough fluid, that fluid stays in your body. Gravity pulls this excess fluid down to the lowest points, which, when standing or sitting, are the feet, ankles, and lower legs.

Sodium Retention: Damaged kidneys also struggle to excrete sodium (salt). Sodium acts like a magnet for water, holding onto fluid in your tissues instead of letting it stay in the bloodstream, where it belongs. This worsens the leakage of fluid into surrounding tissues (a process called peripheral edema).

Protein Loss (Nephrotic Syndrome): In some types of kidney disease (like minimal change disease or diabetic nephropathy), the kidney's filters (glomeruli) become leaky. They spill a crucial protein called albumin into the urine. Albumin normally acts like a sponge in your blood vessels, holding fluid inside them. When albumin levels drop too low, fluid leaks out of the blood vessels and into the surrounding tissues, causing dramatic swelling.

Why this matters clinically: Severe leg swelling isn't just uncomfortable or heavy. It can stretch and break the skin, leading to open wounds (ulcers) that heal poorly, increase the risk of severe skin infections (cellulitis), and can become a portal for sepsis. It's a direct sign that the kidneys are failing a core job.

Why Shortness of Breath (Dyspnea) Matters

Shortness of breath is an even more urgent and dangerous sign. It directly connects kidney failure to the heart and lungs.

Fluid in the Lungs (Pulmonary Edema): This is the single most critical reason. The excess fluid the kidneys can't remove doesn't just go to the legs. When you lie down at night, that fluid from your legs gets reabsorbed into your bloodstream. A weakened or overworked heart (common with kidney disease) may not be able to pump this increased blood volume effectively. As a result, pressure backs up into the blood vessels of the lungs, forcing fluid out of the tiny vessels (capillaries) and into the air sacs (alveoli). This is like lungs drowning from the inside out. The result is:

Difficulty breathing (feeling of suffocation or "air hunger")

Wheezing (sometimes called "cardiac asthma")

Cough with frothy, pink tinged sputum

A feeling of being unable to lie flat (orthopnea), needing to prop up on several pillows to breathe.

Anemia (Low Red Blood Cells): Healthy kidneys produce a hormone called erythropoietin (EPO), which tells your bone marrow to make red blood cells. Diseased kidneys produce less EPO, leading to anemia. Red blood cells carry oxygen. With fewer red blood cells, your body is starved of oxygen, and even minimal exertion can leave you severely short of breath. This is a different mechanism than pulmonary edema, but equally distressing.

Metabolic Acidosis: Kidneys also remove acid from the blood. When they fail, acid builds up. To compensate, your brain instinctively tries to blow off this acid by making you breathe faster and deeper (Kussmaul breathing). This rapid breathing is often perceived as shortness of breath.

Why this matters clinically: Shortness of breath from pulmonary edema is a medical emergency. It can rapidly progress to respiratory failure, requiring immediate hospitalization, oxygen, and emergency dialysis to pull the fluid off the lungs. It is one of the leading causes of hospitalization and death in advanced kidney disease (Stage 5 / End Stage Renal Disease).

The "Heart Kidney Connection" (Cardiorenal Syndrome)

These two symptoms perfectly illustrate the vicious cycle of cardiorenal syndrome.

Bad kidneys cause fluid overload.

Fluid overload puts immense strain on the heart.

Heart failure makes it harder for the kidneys to be perfused with blood, worsening kidney failure.

Kidney failure leads to more fluid overload.

Swollen legs are the early warning. Shortness of breath is the alarm bell.

In Summary:

Symptom What it Signals in Kidney Disease Why it Matters

Swollen Legs The kidneys cannot remove enough fluid and sodium. (Or, they are leaking protein). Indicates fluid overload. Leads to skin breakdown, infection, immobility, and is a precursor to pulmonary edema.

Shortness of Breath Fluid is backing up into the lungs (pulmonary edema), OR severe anemia, OR acid build up in the blood. This is a medical emergency. Pulmonary edema can cause respiratory failure and death. Requires urgent treatment (dialysis, oxygen).

If you or someone you know has kidney disease and develops new or worsening shortness of breath, especially with the inability to lie flat, seek emergency medical attention immediately. If you notice worsening leg swelling, contact your nephrologist promptly it may indicate a need to adjust dialysis, fluids, or medications (like diuretics).

model: Doubao

**answer:**

Kidneys filter waste and excess fluid from blood, regulate blood volume, and balance electrolytes. When kidney function declines, two core problems trigger these symptoms, and they signal dangerous complications:

1. Swollen legs (peripheral edema)

Root cause

Damaged kidneys lose the ability to excrete extra sodium and water. Fluid builds up in the bloodstream, and excess fluid leaks out into soft body tissues. Gravity pulls this fluid down to the lower legs, ankles and feet, causing swelling.

Why it matters

Marker of fluid overload: Edema means the body is retaining dangerous amounts of fluid, not just a cosmetic issue.

Worsens heart strain: Extra blood volume forces the heart to pump harder, quickly leading to heart failure.

Sign of advanced kidney damage: Persistent leg swelling often means glomerular filtration rate (GFR) has dropped significantly.

Secondary risks: Severe edema raises risk of skin breakdown, infection, deep vein thrombosis (DVT).

Linked to protein loss: Many kidney diseases damage glomeruli, leaking albumin into urine (proteinuria). Low blood albumin pulls fluid out of blood vessels into tissues, worsening swelling.

2. Shortness of breath (dyspnea)

This is a medical red flag, far more urgent than leg swelling alone, with two main kidney related causes:

A. Pulmonary edema (fluid in the lungs)

Excess retained fluid moves from blood vessels into lung air sacs. Oxygen cannot cross into the blood normally, causing breathlessness, especially when lying flat, nighttime breathlessness, or frothy cough. This is life threatening acute heart/lung congestion.

B. Anemia from chronic kidney disease

Healthy kidneys make erythropoietin (EPO), a hormone that stimulates red blood cell production. Failed kidneys stop making enough EPO to severe anemia. Low red blood cells reduce oxygen delivery to all organs, creating constant fatigue and shortness of breath with mild activity.

C. Electrolyte imbalances

Advanced kidney failure causes high potassium or acid buildup, which weakens heart muscle, reduces cardiac output, and triggers breathlessness.

Combined danger: kidney heart connection (cardiorenal syndrome)

Leg swelling plus shortness of breath together prove cardiorenal syndrome: kidney dysfunction damages the heart, and heart failure further worsens kidney function in a vicious cycle.

Untreated fluid overload can lead to acute heart failure, pulmonary edema, arrhythmia, or sudden cardiac death the top cause of death in chronic kidney disease patients.

These symptoms mean treatment adjustments are urgently needed: diuretics to remove fluid, blood pressure control, protein restricted diet, EPO therapy for anemia, or dialysis in late stage kidney failure.

Summary

Swollen legs signal dangerous fluid retention and protein loss; shortness of breath points to fluid flooding the lungs, severe anemia, or heart strain. Both are not minor discomfort they are early warning signs of life threatening heart and organ complications caused by failing kidney filtration and hormone function.

model: Kimi

**answer:**

Swollen legs and shortness of breath are two of the most clinically significant symptoms in kidney disease because they usually signal that the kidneys are failing to regulate fluid balance, electrolytes, or blood composition and they can rapidly become life threatening.

Here is why each matters:

Swollen Legs (Peripheral Edema)

In healthy kidneys, excess sodium and water are filtered out and sent to the bladder. When kidney function declines:

Sodium and water retention: Damaged nephrons lose the ability to excrete sodium, so water follows by osmosis and accumulates in tissues.

Low albumin (nephrotic syndrome): Damaged kidneys leak protein into the urine. Low blood albumin reduces the osmotic pressure that keeps fluid inside blood vessels, so fluid leaks into tissues often starting in the legs due to gravity.

Why it matters: Persistent edema stretches the skin, causes discomfort and impaired mobility, and indicates advancing fluid overload. It also raises blood pressure, which further damages the kidneys and cardiovascular system.

Shortness of Breath (Dyspnea)

This is often more urgent than leg swelling because it can reflect several dangerous processes:

Pulmonary edema: When fluid overload becomes severe, excess fluid backs up into the lungs. This impairs gas exchange and can cause oxygen deprivation. It is a medical emergency.

Anemia of chronic kidney disease (CKD): Diseased kidneys produce less erythropoietin (EPO), the hormone that stimulates red blood cell production. Fewer red blood cells mean less oxygen delivery to tissues, so the body compensates by increasing breathing rate.

Metabolic acidosis: Failing kidneys cannot excrete acid effectively. The blood becomes acidic, and the lungs compensate by hyperventilating (Kussmaul breathing) to blow off carbon dioxide.

Uremic pericarditis/pleuritis: In advanced disease, uremic toxins can inflame the lining around the heart or lungs, causing pain and restricted breathing.

Why These Symptoms Are Red Flags

Together, leg swelling and shortness of breath suggest the body is accumulating fluid faster than it can be removed. This state of volume overload increases the risk of:

Hypertensive crisis and heart failure

Pulmonary edema requiring emergency dialysis

Accelerated cardiovascular damage (the leading cause of death in CKD patients)

In clinical practice, the appearance or worsening of these symptoms often triggers immediate evaluation of dialysis needs, diuretic adjustments, or hospitalization.

This information is for educational purposes only and does not replace professional medical advice. If you or someone you know is experiencing these symptoms, consult a healthcare provider promptly.

model: Qwen

**answer:**

In kidney disease, leg swelling and shortness of breath are critical warning signs indicating disease progression or complications, reflecting impaired fluid excretion and systemic fluid overload.

Leg Swelling: A Sign of Reduced Fluid Excretion

The kidneys act as the body's "treatment plant," filtering out excess water and salt. When kidney function declines, this ability is compromised.

Fluid Accumulation: Excess fluid builds up in the body. Due to gravity, it tends to pool in the lowest areas, such as the ankles and calves.

Hypoalbuminemia: If kidney disease causes significant protein loss through urine, the protein levels in the blood drop. This reduces the blood's ability to hold fluid, allowing it to leak into tissue and form pitting edema.

Shortness of Breath: A Sign of Circulatory Overload

Shortness of breath is often more critical than leg swelling, suggesting that the heart or lungs are affected.

Pulmonary Edema: Excess fluid can enter the lung tissue. When alveoli are filled with fluid, it hinders oxygen exchange, causing chest tightness and breathlessness.

Increased Cardiac Load: Excess fluid increases total blood volume, forcing the heart to work much harder. Over time, this can lead to heart failure, which further worsens fluid retention and shortness of breath.

Summary and Management Advice

These symptoms are key indicators for assessing disease progression.

If you or a family member experiences rapid swelling spreading to the thighs or face, shortness of breath that worsens when lying flat, or a significant decrease in urine output, please seek immediate medical attention at a nephrology department.

**Q39. When should a person with diabetic kidney disease see a kidney specialist?**

model: ChatGPT

**answer:**

A person with diabetic kidney disease should see a kidney specialist (nephrologist) when kidney damage is more advanced, worsening, or difficult to manage.

Common referral triggers include:

See a nephrologist when. What it means

eGFR is below 30 mL/min/1.73 m2 CKD stage 4 to 5 range; specialist care is recommended.

Urine albumin to creatinine ratio (uACR) is above 300 mg/g Severe albuminuria/protein leakage.

eGFR drops quickly For example, a greater than 25% fall or a sustained decline greater than 5 mL/min/1.73 m2 per year.

Blood pressure remains high despite several medicines Especially if still uncontrolled on multiple antihypertensive drugs.

Potassium is persistently high, metabolic acidosis occurs, or CKD complications appear These may need specialist medication/diet adjustment.

There is blood in the urine, recurrent kidney stones, or the cause of CKD is unclear This may suggest another kidney problem in addition to diabetes.

Planning is needed for dialysis, transplant, or advanced CKD education Earlier planning improves safety and options.

The National Kidney Foundation specifically lists nephrology referral when eGFR less than 30 or uACR greater than 300 mg/g, and also for rapid kidney function decline, resistant hypertension, persistent hyperkalemia/metabolic acidosis, unexplained hematuria, recurrent stones, or hereditary/unknown CKD causes.

Even before these thresholds, referral can be helpful if the primary doctor is unsure about treatment, kidney function is steadily declining, albuminuria persists despite treatment, or the patient wants more detailed kidney protection planning. CKD is usually monitored with two key tests: blood creatinine/eGFR and urine uACR.

Seek urgent medical care rather than waiting for a routine referral if there is severe shortness of breath, chest pain, confusion, very little urine, sudden major swelling, very high blood pressure with symptoms, or suspected acute kidney injury.

model: Gemini

**answer:**

According to guidelines from the American Diabetes Association (ADA) and KDIGO, people with diabetic kidney disease should be referred to a nephrologist if:

Their eGFR falls below 30 mL/min/1.73 m².

Kidney function declines rapidly or urinary albumin levels continue to rise despite appropriate treatment.

The cause of kidney disease is uncertain, such as when kidney function worsens suddenly, blood is found in the urine, or diabetic retinopathy is absent.

Complications are difficult to control, including resistant high blood pressure, severe anemia, metabolic bone disease, or persistent high potassium levels.

If kidney function is stable and the eGFR remains above 30 mL/min/1.73 m², care can usually be provided by a primary care doctor or endocrinologist. Management may include controlling blood pressure and blood sugar and using kidney-protective medications such as SGLT2 inhibitors, ACE inhibitors, or ARBs when appropriate.

model: DeepSeek

**answer:**

A person with diabetic kidney disease (DKD) should see a kidney specialist (nephrologist) in the following situations:

Persistently elevated urine albumin excretion Urine albumin to creatinine ratio (UACR) greater than or equal to 300 mg/g (or greater than or equal to 30 mg/g with rapid decline in eGFR or other concerns).

Declining kidney function Estimated glomerular filtration rate (eGFR) less than 45 mL/min/1.73 m2, or a persistent drop in eGFR of greater than or equal to 5 mL/min/1.73 m2 per year or greater than or equal to 10 mL/min/1.73 m2 over 2 to 3 years.

Difficult to control blood pressure or diabetes Especially when medication adjustments are complex or limited due to kidney disease.

Unexplained or rapid worsening of kidney function eGFR decline out of proportion to expected DKD progression.

Electrolyte or acid base disorders Such as hyperkalemia, metabolic acidosis, or hypocalcemia/hyperphosphatemia.

Anemia of CKD Hemoglobin consistently less than 10 to 11 g/dL without other obvious causes.

Possible non diabetic kidney disease Features like active urine sediment (red blood cell casts), rapidly rising creatinine, or nephrotic syndrome.

Need for kidney replacement therapy planning eGFR less than 20 to 30 mL/min/1.73 m2 to discuss dialysis or transplant options.

General guideline: Many experts recommend a nephrology referral when eGFR falls below 45 to 60 mL/min/1.73 m2 or UACR exceeds 300 mg/g, as early specialist involvement can slow progression, manage complications, and improve outcomes. Patients with type 1 diabetes and microalbuminuria also often benefit from early referral.

model: Doubao

**answer:**

1. Timely referral thresholds (core indications)

(1) Early screening abnormalities in diabetic patients

Persistent albuminuria

UACR (urinary albumin to creatinine ratio) greater than or equal to 30 mg/g on two separate urine tests within 3 to 6 months, even with normal estimated glomerular filtration rate (eGFR). This marks the onset of diabetic kidney damage.

Declined renal filtration function

eGFR less than 60 mL/min/1.73m2 confirmed twice, regardless of urine protein levels.

(2) Moderate to advanced chronic kidney disease (CKD) stages

CKD stage G3a (eGFR 45 to 59): Mandatory nephrology co management to slow progression.

CKD stage G3b G5 (eGFR less than 45): Formal long term follow up by a kidney specialist to prepare for renal replacement therapy (dialysis/transplant) if needed.

2. Urgent referral situations (see nephrologist within 1 to 2 weeks)

Rapid renal function drop: eGFR falls by greater than or equal to 15 mL/min/1.73m2 within 12 months.

Severe proteinuria: UACR greater than 300 mg/g or overt nephrotic range proteinuria with edema, hypoalbuminemia.

Difficult to control hypertension despite multiple antihypertensive drugs, especially elevated nighttime blood pressure.

Persistent electrolyte disorders: hyperkalemia, metabolic acidosis, hyperphosphatemia.

Severe complications: refractory anemia of CKD, recurrent heart failure, severe pruritus, bone pain (renal osteodystrophy).

3. Red flags for emergency nephrology visit

Severe hyperkalemia (K plus greater than 5.5 mmol/L) with arrhythmia risk

Acute kidney injury: sudden sharp rise in serum creatinine

Refractory volume overload: severe whole body edema, pulmonary edema, oliguria/anuria

4. Other special scenarios requiring nephrology consultation

Uncertain diagnosis: Cannot distinguish pure DKD from other kidney diseases (e.g, glomerulonephritis) based on lab results.

Planning medication adjustment: Need to use SGLT 2 inhibitors, RAAS blockers (ACEI/ARB), or renoprotective drugs with significant renal dose adjustments.

Pre surgical evaluation: Major surgery for diabetic patients with impaired kidney function.

Pregnancy in DKD patients: High risk of rapid kidney function deterioration.

Supplementary management principle

All type 1 diabetes patients with disease duration greater than 5 years and all newly diagnosed type 2 diabetes patients should complete annual UACR and eGFR screening by primary care or endocrinologists. Once any above abnormal marker appears, joint care with a nephrologist is required without delay.

model: Kimi

**answer:**

People with diabetic kidney disease (DKD) should see a nephrologist (kidney specialist) based on both kidney function levels and clinical warning signs. Here are the evidence based thresholds and red flags:

By Kidney Function (eGFR)

eGFR less than 30 mL/min/1.73 m2 (G4 G5, or stages 4 to 5): Referral is strongly recommended. This is when complications of advanced kidney disease become more likely and treatment planning for potential dialysis or transplant should begin.

eGFR 30 to 44 mL/min/1.73 m2 (G3b): Consider referral, especially if there is rapid progression or other risk factors.

eGFR 45 to 59 mL/min/1.73 m2 (G3a): Usually managed by the primary care physician or endocrinologist unless other concerns exist.

By Albuminuria/Proteinuria

UACR greater than or equal to 300 mg/g (A3/severe albuminuria): Referral is advisable, as this indicates significant kidney damage.

Nephrotic range proteinuria: UACR greater than 2200 mg/g or urine protein greater than 3.5 g/day this warrants prompt nephrology evaluation.

Rapid Progression (Refer Regardless of Current Stage)

eGFR decline greater than 5 mL/min/1.73 m2 per year, or

greater than 10% drop in eGFR within 12 months

Other Red Flags Requiring Referral

Refractory hypertension (blood pressure difficult to control despite 3 plus medications)

Persistent electrolyte abnormalities (e.g, high potassium, high phosphorus)

Recurrent or unexplained hematuria (blood in urine)

Rapidly worsening swelling (edema) or signs of fluid overload

Uncertain diagnosis if it's unclear whether kidney disease is from diabetes or another cause (e.g, suspicion of glomerulonephritis, vasculitis, or another primary kidney disease)

Anemia of chronic kidney disease that is difficult to manage

Preparing for kidney replacement therapy (dialysis or transplant) ideally referred well before it becomes urgent

Bottom Line

At the latest, when eGFR falls below 30. Ideally earlier if there is severe albuminuria, rapid decline, difficult to control blood pressure, or diagnostic uncertainty. Early nephrology involvement (even at eGFR 30 to 45) has been associated with better outcomes, fewer hospitalizations, and more time to plan for treatment options.

This information is for educational purposes and is not a substitute for personalized medical advice. If you or someone you know has DKD, the treating physician should make referral decisions based on the complete clinical picture.

model: Qwen

**answer:**

A person with diabetic kidney disease should consult a nephrologist when the estimated glomerular filtration rate (eGFR) is below 30, urine protein increases suddenly, or blood pressure is difficult to control.

Urgent Situations for Immediate Referral

Severe Kidney Function Decline: An eGFR below 30 mL/min/1.73m2 indicates critically impaired waste clearance. At this stage, early evaluation for dialysis or transplantation options is necessary.

Rapidly Worsening Proteinuria: A sharp spike in urine albumin to creatinine ratio (UACR), such as 24 hour urine protein exceeding 1 gram, suggests accelerating damage.

Uncontrolled Blood Pressure: Persistent high blood pressure despite the use of multiple antihypertensive medications.

Atypical Symptoms: The presence of significant blood in the urine (hematuria), red blood cell casts, or abnormal kidney size and structure on ultrasound may indicate a non diabetic kidney disease requiring specialized intervention.

Early Referral Recommendations

Persistent Abnormalities: An eGFR consistently below 60 mL/min/1.73m2, or a UACR persistently greater than 30 mg/g for over 3 months.

Complex Comorbidities: Concurrent severe hypertension, hyperuricemia, lipid abnormalities, or cardiovascular disease.

Treatment Plateau: Lack of improvement in kidney function despite standard therapy with cardio renal protective medications like SGLT2 inhibitors or RAAS inhibitors.

Division of Labor Between Specialists

Endocrinologist: Focuses on comprehensive glycemic control and adjusting diabetes medication regimens to address the metabolic root cause.

Nephrologist: Specializes in slowing the progression of renal function decline, precisely managing blood pressure, and handling complications such as anemia and mineral bone metabolism disorders.

Early consultation with a nephrologist is essential for preserving residual kidney function and delaying the progression to end stage renal disease. If key indicators are abnormal or complex situations arise, seeking expert help promptly is the key to better long term outcomes.

**Dimension 8-Emotional support and risk communication**

**Q40. I just found out I have protein in my urine and I am scared. What should I do next?**

model: ChatGPT

**answer:**

I'm really sorry you're scared. Protein in urine is a warning sign to follow up, not a diagnosis by itself. It can happen temporarily from dehydration, fever, stress, strenuous exercise, diet, or pregnancy but if it persists, it can be an early sign of kidney disease or kidney damage.

What to do next:

Contact your primary care doctor/clinic this week and ask what your exact result was: "trace/1 plus /2 plus " on dipstick, or a number such as urine albumin to creatinine ratio (uACR) or protein to creatinine ratio (UPCR).

Ask for confirmation testing. A common next step is a uACR urine test plus a blood creatinine/eGFR test to check kidney filtering. NIDDK notes that uACR less than or equal to 30 mg/g is normal, while greater than 30 mg/g may be a sign of kidney disease, and providers often repeat the urine test once or twice to confirm.

Do not panic if it was one test. The National Kidney Foundation describes persistent protein as two positive tests over 3 or more months, so a single abnormal test often needs repeat testing before conclusions are made.

Seek urgent care now if you have any of these: shortness of breath, chest pain, confusion, very little urine, new/worsening swelling of face/legs/feet, blood in urine, severe vomiting, or severe side/back pain. These can be signs of more serious kidney or fluid balance problems.

If you are pregnant or recently postpartum, call your OB/maternity unit today, especially if you also have high blood pressure, headache, vision changes, upper abdominal pain, or swelling protein in urine with high blood pressure can be an early sign of pre eclampsia.

Before the appointment, write down: your urine result, blood pressure readings if you have them, whether you recently exercised hard, had fever/dehydration, are menstruating, have diabetes/high blood pressure, and any medicines such as ibuprofen/naproxen. MedlinePlus notes diabetes, high blood pressure, heart disease, family history, and some medicines can affect kidney risk.

You're doing the right thing by taking it seriously early. The key next step is confirming whether the protein is temporary or persistent and checking kidney function.

model: Gemini

**answer:**

It is completely understandable to feel scared when you see a test result you weren't expecting, but finding protein in your urine (a condition called proteinuria) is a very common starting point for a lot of people, and it doesn't automatically mean something is permanently wrong.

Let's take a deep breath and break down exactly what this means and what your next steps should be.

What Does Protein in Your Urine Mean?

Normally, your kidneys act like fine filters, keeping important things like protein in your blood while filtering out waste. If your kidneys are stressed, or if the filters are temporarily wider than usual, protein can leak into your urine.

Here is the most important thing to know right now: Proteinuria can be temporary.

It is often caused by everyday things that have nothing to do with kidney disease, such as:

Dehydration

Recent intense exercise

A recent high fever or illness

High stress

What You Should Do Next

To figure out if this is just a temporary spike or something that needs a bit more attention, here is a clear path forward:

Schedule a Follow Up with Your Doctor

Because a single test only shows a snapshot in time, the absolute first step is to have your doctor repeat the urine test. They will often order a more precise test called a UACR (Urine Albumin to Creatinine Ratio) to see exactly how much protein is present.

Review Your Vitals and Medical History

When you see your doctor, make sure to check your blood pressure and blood sugar. The two most common manageable causes of persistent protein in the urine are high blood pressure and diabetes. Catching and managing these early is incredibly effective at protecting your kidneys.

Stay Hydrated

Drink a normal, healthy amount of water. Don't go overboard and chug gallons to try and "flush it out," as this can throw off your body's electrolyte balance, but do ensure you aren't dehydrated.

Avoid High Dose NSAIDs

Until you speak with your doctor, try to avoid overusing over the counter pain relievers like ibuprofen (Advil, Motrin) or naproxen (Aleve), as these can put extra stress on the kidneys. Acetaminophen (Tylenol) is generally a safer choice for temporary pain relief in the meantime.

When to seek immediate care: If your protein result is accompanied by sudden, severe swelling in your legs, ankles, or around your eyes, or if you experience a sharp decrease in how much you are urinating, contact a doctor right away.

For now, try not to jump to worst case scenarios. Getting a repeat test is the best way to get clear, accurate answers. Do you happen to know if this was a quick "dipstick" test at a clinic, or part of a larger lab panel? If you have other symptoms or health conditions, sharing them could help me give more relevant information.

model: DeepSeek

**answer:**

First of all, it's completely understandable to feel scared, but please know that protein in urine (proteinuria) is often temporary or benign it doesn't automatically mean serious kidney disease.

Here's what you should do next, step by step:

1. Don't panic confirm the result with your doctor

A single positive dipstick test can be caused by dehydration, fever, intense exercise, stress, a urinary tract infection, or even standing for a long time (orthostatic proteinuria).

Your doctor will likely repeat the test, ideally with a first morning urine sample, and possibly a urine protein to creatinine ratio (UPCR) or albumin to creatinine ratio (UACR) for accuracy.

2. Check if you have any other symptoms

If you have none of the below, the cause is more likely benign:

Foamy or bubbly urine (like beer foam that doesn't disappear quickly)

Swelling in feet, ankles, legs, or around eyes

Fatigue, nausea, loss of appetite

High blood pressure (you can check at a pharmacy or home monitor)

If you do have swelling or high blood pressure, contact your doctor this week. Otherwise, scheduling a routine follow up in 1 to 2 weeks is fine.

3. Expect basic follow up tests

Your doctor will likely order:

Blood tests: creatinine, eGFR, blood pressure checks

Repeat urinalysis

Possibly a renal ultrasound if proteinuria persists

4. Ask your doctor these specific questions

"Is this likely transient or persistent proteinuria?"

"Do I need a 24 hour urine collection or a protein to creatinine ratio?"

"Could my medications (NSAIDs like ibuprofen, certain antibiotics) be causing this?"

5. Protect your kidneys while you wait

Hydrate well (unless your doctor tells you otherwise)

Avoid NSAIDs (ibuprofen, naproxen, aspirin unless prescribed)

Avoid high protein supplements and excessive salt

If you have diabetes or high blood pressure, keep them tightly controlled

When to seek immediate care Go to urgent care or ER if you develop:

Severe swelling with trouble breathing

Very high blood pressure (e.g, greater than 160/100)

Decreased urine output or confusion

Finally, remember: most people with mild proteinuria never develop kidney failure, especially if caught early. The fear you feel is normal, but the next step is simple call your doctor for a repeat test and blood pressure check. You're already doing the right thing by looking into it.

model: Doubao

**answer:**

1. Calm down first: Not all urine protein means severe kidney disease

Temporary, harmless proteinuria is very common:

Strenuous exercise, high fever, severe stress, cold/flu, dehydration

Eating a huge amount of protein in one meal

Standing for long hours (orthostatic proteinuria, common in young people)

These causes disappear on their own after adjustment, with no permanent kidney damage.

Persistent proteinuria is the only sign that needs close monitoring.

2. Repeat the urine test correctly (most critical next step)

Recommended tests to ask your doctor for:

Urine routine plus Urine microalbumin/creatinine ratio (UACR)

This is the gold standard to quantify tiny amounts of protein; a random spot urine sample works, no need for full 24 hour collection at first.

If UACR is abnormal: 24 hour urine total protein quantification to measure exact daily protein loss.

Test preparation rules to avoid false positives:

Avoid heavy exercise 24 hours before testing

Skip high protein supplements/extra meat the day before

Drink enough water to avoid concentrated urine

Collect midstream clean urine (wash private area first)

Do not test during menstruation (women) or urinary tract infection

3. Check for underlying root causes with blood work

Ask your doctor to order these blood tests to assess kidney function and triggers:

Renal function: Creatinine, eGFR, urea nitrogen, uric acid

Blood glucose / HbA1c (rule out diabetes, the top cause of kidney protein loss)

Blood lipid panel

Blood pressure measurement (hypertension damages kidney filters)

Autoimmune markers (if swelling, joint pain exist: rule out lupus nephritis)

Infection screening (urine culture to rule out UTI, which causes temporary protein)

4. Daily lifestyle adjustments you can start immediately

Diet

Do not cut protein drastically (malnutrition worsens kidney injury). Maintain moderate, balanced protein: 0.8 to 1.0g per kg of body weight daily; limit processed meat, sausage, high protein shakes.

Strictly reduce salt: under 5g salt per day. High sodium raises blood pressure and worsens urine protein. Avoid pickles, fast food, canned food.

Limit sugary drinks, refined carbs to protect blood sugar.

Habits

Monitor blood pressure at home if you have hypertension; target less than 130/80 mmHg for kidney protection.

Avoid intense workouts temporarily until recheck results come back; light walking is fine.

Stay hydrated with plain water; avoid excessive energy drinks and alcohol.

Stop over the counter painkillers (ibuprofen, naproxen) they damage kidneys and boost urine protein. Use paracetamol only if necessary, per dosage instructions.

5. Warning signs that require urgent medical visit

Go to hospital right away if you have any of these:

Swelling of face, eyelids, legs/ankles that does not fade

Foamy urine that lasts for minutes every time you pee

Persistent fatigue, nausea, poor appetite

Uncontrolled high blood pressure

Blurred vision, frequent thirst/frequent urination (signs of diabetes)

Dark tea colored urine

6. General treatment logic after diagnosis

Transient proteinuria (exercise/infection/dehydration): No medication needed, repeat test after 1 to 2 weeks of rest and hydration, it will resolve.

Mild persistent proteinuria plus high blood pressure: Doctors usually prescribe ACEI/ARB blood pressure drugs, which lower urine protein and protect kidneys (must use under medical supervision).

Diabetes related proteinuria: Strict blood sugar plus blood pressure control is core treatment.

Severe protein loss / nephropathy: Further kidney ultrasound or kidney biopsy may be arranged by nephrologists for targeted therapy.

Final tip

Schedule an appointment with a nephrologist (kidney specialist) instead of only a general practitioner if your repeat urine test still shows protein. Early detection and intervention almost always prevent progressive kidney damage, so there is no need for extreme fear most mild proteinuria can be well controlled long term.

model: Kimi

**answer:**

I understand how scary this can feel. Let me be direct and helpful: protein in your urine (proteinuria) is a sign that your kidneys need to be checked by a doctor, but it does not automatically mean something terrible. The cause could range from something temporary and harmless (like a recent intense workout, fever, or dehydration) to something that needs ongoing treatment.

What you should do next:

Make an appointment with a doctor as soon as you can ideally within the next few days to a week. A primary care physician is a good starting point; they may refer you to a nephrologist (kidney specialist).

Don't panic, but don't ignore it. One positive test often needs confirmation. Your doctor will likely order a repeat urine test and possibly blood tests to check kidney function.

Write down any symptoms you've noticed: swelling in legs/face, foamy or bubbly urine, fatigue, high blood pressure, changes in urination, or recent illness.

Note recent factors that can temporarily cause protein in urine: strenuous exercise, fever, dehydration, urinary tract infection, or certain medications (like NSAIDs such as ibuprofen). Share these with your doctor.

Why this matters: Your kidneys normally filter waste while keeping protein in your blood. When protein leaks into urine, it suggests the kidney filters may be irritated or damaged. Catching it early is important because many causes are very treatable.

I am not a doctor, and I cannot diagnose you or replace medical advice. If you are experiencing severe symptoms like major swelling, very high blood pressure, or dramatically reduced urine, seek urgent care.

You are doing the right thing by paying attention to this. Please schedule that appointment.

model: Qwen

**answer:**

Don't worry, finding protein in your urine doesn't mean your kidneys are damaged. It is often temporary, and the next step is to retest and consult a specialist if needed.

Why Protein Might Appear in Urine

Temporary Factors: Recent intense exercise, stress, fever, or being on your period can cause a temporary leak. This is a false alarm.

Kidney or Chronic Issues: If it happens repeatedly, it could indicate kidney problems or damage from chronic conditions like high blood pressure or diabetes.

Next Steps to Take

Retest Properly: Wait until you feel healthy and have not exercised intensely. If you are menstruating, wait until it ends. When you go, provide a "midstream sample" (start peeing, catch the middle part, then finish) to avoid contamination.

Advanced Testing: If the second test is still positive, your doctor will likely order blood tests to check your kidney function, an ultrasound, or a test to measure exactly how much protein is leaking.

Daily Habits: Eat a low salt diet (less than 5g of salt per day) and avoid unregulated supplements to reduce the burden on your kidneys.

A single positive result is just an early warning sign. Stay calm, follow these steps to get retested, and most issues can be managed effectively.

**Q41. I am afraid I will end up on dialysis like a relative. Is there anything I can do now?**

model: ChatGPT

**answer:**

Yes. Having a relative on dialysis does not mean you will end up there, but it is a reason to be proactive. A close family history of CKD increases risk, and the biggest modifiable drivers are usually blood pressure, diabetes/blood sugar, urine protein, medicines, weight, and smoking.

The most useful next step is to ask your primary care doctor for two kidney tests:

eGFR from a blood test, which estimates kidney filtering, and uACR from a urine test, which checks for albumin/protein leakage. The National Kidney Foundation emphasizes these two tests because early kidney damage can be silent.

Things you can do now:

Know and control your blood pressure. If you already have CKD or albumin in the urine, blood pressure control is one of the strongest ways to slow progression. Doctors often use ACE inhibitors or ARBs when appropriate, especially with albuminuria, but they need monitoring for potassium and kidney function.

If you have diabetes or prediabetes, tighten the plan with your clinician. Good glucose control can reduce albuminuria risk; A1c goals should be individualized rather than one size fits all.

Ask whether kidney protective medicines apply to you. SGLT2 inhibitors are now commonly used for many people with CKD, with or without diabetes, especially when albuminuria is present; they can slow CKD progression and lower kidney failure and heart failure risks. They are prescription medicines, so this is a discussion with your clinician.

Be careful with over the counter pain medicines. NSAIDs such as ibuprofen and naproxen can worsen kidney function, especially in people with CKD, diabetes, high blood pressure, dehydration, or long term/high dose use. Ask what pain relief options are safest for you.

Reduce kidney strain through lifestyle basics: keep salt intake down, maintain a healthy weight, avoid smoking, exercise regularly, and avoid excessive protein intake unless a clinician or renal dietitian recommends otherwise. NIDDK notes that sodium restriction, weight loss, and avoiding excessive dietary protein may reduce albuminuria.

A simple script for your appointment:

"Because I have a relative who needed dialysis, can we check my eGFR and urine albumin to creatinine ratio? Do I have any signs of CKD, high blood pressure, diabetes, or protein in the urine? Are there medicines or diet changes that would protect my kidneys?"

Seek urgent care sooner if you have severe swelling, shortness of breath, very little urination, chest pain, confusion, or very high blood pressure.

model: Gemini

**answer:**

It is completely understandable to feel anxious after watching a loved one go through dialysis. It's a demanding process, and wanting to take proactive steps for your own health is a very smart, positive reaction to that fear.The most reassuring thing to know is that kidney disease is not an inevitable destination, even with a family history. There are clear, powerful actions you can take right now to protect your kidneys. Because early kidney disease rarely has symptoms, prevention is all about managing the hidden risk factors. 1. Know Your "Kidney Numbers"The single most important step is to ask your doctor for two simple, routine tests at your next checkup. Do not rely on how you "feel."eGFR (Estimated Glomerular Filtration Rate): A simple blood test that measures how well your kidneys are filtering waste. uACR (Urine Albumin Creatinine Ratio): A quick urine test that checks for microscopic traces of protein (albumin). If protein is leaking into your urine, it's an early warning sign of kidney stress. 2. Fiercely Protect Your Blood Pressure and Blood SugarAccording to the National Kidney Foundation (NKF), diabetes and high blood pressure are the top two causes of kidney failure. They quietly damage the delicate, microscopic blood vessels inside your kidneys over time. Blood Pressure: Aim to keep it under control (ideally below 130/80 mmHg, though your doctor will give you a specific target).Blood Sugar: If you have diabetes or prediabetes, keeping your A1C in a healthy range is the absolute best way to shield your kidneys from harm.3. Be Smart About Over the Counter Pain RelieversThis is a trap that catches many people off guard. Common over the counter NSAIDs (Non Steroidal Anti Inflammatory Drugs) like ibuprofen (Advil, Motrin) and naproxen (Aleve) can reduce blood flow to the kidneys. Taking them occasionally is usually fine, but heavy or daily use can cause chronic kidney damage. Peers Tip: If you need daily pain management, talk to your doctor about kidney safe alternatives, like acetaminophen, or alternative therapies.4. Adopt Kidney Friendly HabitsWhat is good for your heart is almost always good for your kidneys.Watch the Salt: Try to stay under 2,300 mg of sodium per day. Cook with herbs and spices instead of reaching for the salt shaker, as high sodium drives up blood pressure. Stay Hydrated: Drink plenty of water throughout the day, which helps your kidneys clear toxins. (You don't need to overdo it; just listen to your thirst). Move Regularly: Aim for 30 minutes of moderate activity (like brisk walking) most days of the week to keep your blood vessels healthy. Ditch the Tobacco: Smoking damages blood vessels throughout the entire body, significantly reducing critical blood flow to your kidneys.

Taking control of these factors drastically changes your trajectory. Your next best step is simply to call your primary care doctor, mention your relative's history, and ask to have your eGFR and uACR checked.

model: DeepSeek

**answer:**

This is a very understandable fear, especially with a family history. The good news is: yes, there is a great deal you can do right now to protect your kidneys, regardless of your genetic risk.

Kidney failure (dialysis) is rarely sudden. It's usually the end result of years of gradual damage from conditions you can monitor and manage. Your relative's experience is a warning, not a prophecy.

Here is the evidence based action plan to lower your risk as much as possible.

1. First, find out why your relative needed dialysis

This is the single most important question. Ask your relative (or a family member) the cause. The answer changes your game plan entirely.

If it was diabetes (most common cause): Your focus is blood sugar control and insulin resistance.

If it was high blood pressure (2nd most common): Your focus is salt intake, stress, and cardiovascular health.

If it was polycystic kidney disease (genetic): Your focus is imaging (ultrasound) to see if you inherited it, then blood pressure control.

If it was glomerulonephritis (immune): Your focus is avoiding NSAIDs and monitoring urine for blood/protein.

2. Get two simple tests done this month

You cannot manage what you don't measure. Ask your doctor for:

Blood test: eGFR (estimated Glomerular Filtration Rate). This tells you your current kidney function %.

Urine test: ACR (Albumin to Creatinine Ratio). This detects tiny amounts of protein leakage, which is the earliest sign of damage (even when eGFR is normal).

If both are normal today, your risk of needing dialysis in the next 5 to 10 years is very low if you maintain healthy habits.

3. The three most powerful things you can control starting today

A. Stop over the counter NSAIDs (if you use them)

What to avoid: Ibuprofen (Advil, Motrin), Naproxen (Aleve), Diclofenac. Even a few times a week over years can silently scar kidneys.

Safe alternatives for pain: Acetaminophen (Tylenol) is kidney safe. For inflammation, try topical diclofenac gel (minimal absorption) or consult your doctor.

B. Master your blood pressure (even if you think it's fine)

Kidneys love low, stable pressure. Ideal for kidney protection is less than 120/80 (not just less than 140/90).

Action: Buy a home BP monitor (30 to 50). Check it twice weekly. If consistently greater than 125/85, see your doctor. Treating mild high BP can cut kidney decline risk by 50%.

C. Rethink protein and salt

Salt: Directly raises BP and makes kidneys work harder. Aim for less than 2,300 mg/day (1 tsp). Avoid processed foods, deli meats, canned soups, frozen dinners.

Protein: You need protein, but excess (especially from red meat/animal sources) increases kidney filtration pressure. Keep portions palm sized. Plant proteins (beans, lentils, tofu) are gentler.

4. What about water, baking soda, and "kidney cleanses"?

Water: Drink when thirsty. Do not force gallons of water (it does not "flush" kidneys and can backfire).

Baking soda: Do not take this. People online recommend it for kidneys, but it can cause dangerous fluid retention, high blood pressure, and metabolic issues.

Avoid all "kidney cleanse" teas/supplements. Many contain aristolochic acid, heavy metals, or unstudied herbs that cause kidney failure. No supplement is proven to reverse or prevent CKD.

5. The timeline of reassurance

Now to 5 years: Your lifestyle changes have the biggest impact here. You can stop early damage completely.

5 to 20 years: If you maintain BP less than 120/80, avoid NSAIDs, and control blood sugar (if prediabetic), your risk of ever needing dialysis drops by about 80 to 90% compared to someone with untreated risk factors.

After 60: Some age related decline is normal (eGFR about 70 to 80 instead of 100). That rarely leads to dialysis unless combined with the problems above.

Your immediate next steps (this week)

Call your relative or family to learn the exact cause of their kidney failure.

Schedule an annual physical and specifically ask for eGFR and urine ACR.

Empty your medicine cabinet of ibuprofen/naproxen. Replace with acetaminophen.

Buy a home blood pressure monitor (Omron or similar).

If your tests come back normal and you follow the steps above, your fear of dialysis can shift from "likely" to "extremely unlikely." You have a window of years often decades to act before any irreversible damage occurs. You are not doomed by your genes; you are warned by them. That warning gives you power.

model: Doubao

**answer:**

Practical steps to slow kidney decline and avoid dialysis

Many people with early kidney concerns fear progressing to dialysis, but proactive lifestyle and medical management can drastically lower this risk even if you already have mild kidney damage. Below is clear, actionable advice:

1. Strictly control blood pressure (the 1 cause of kidney failure)

Target BP: Keep it below 130/80 mmHg, ideally 120/70 for kidney protection.

Take prescribed kidney friendly meds (ACE inhibitors / ARBs) consistently these directly shield nephrons, do not stop them without your doctor's approval.

Limit salt entirely: No pickles, processed meats, fast food, canned soups, sauces (soy sauce, ketchup). Aim for under 5g salt per day.

2. Manage blood sugar if you have diabetes (second leading cause of dialysis)

HbA1c target: Under 7% for most adults with kidney disease.

Avoid sugary drinks, pastries, refined carbs (white bread, white rice). Pair carbs with protein/fiber to prevent blood sugar spikes.

Check fasting glucose regularly and adjust insulin/anti diabetic drugs per your nephrologist.

3. Optimize your diet to reduce kidney workload

Core rules:

Moderate high quality protein (not zero protein): Too much protein creates waste the kidneys must filter. Stick to the protein amount your nephrologist recommends (usually 0.6 to 0.8g per kg of body weight daily). Choose egg white, fish, lean chicken over red meat in large portions.

Restrict phosphorus: Cut dairy, processed cheese, nuts, cola, packaged snacks high phosphorus damages blood vessels in kidneys.

Adjust potassium if lab results show high levels: Limit bananas, oranges, potatoes, tomatoes, avocados.

Drink fluid as advised: Do not overhydrate; if you have swelling/elevated creatinine, follow your doctor's fluid limit.

4. Avoid all kidney toxic substances

Skip over the counter painkillers: Ibuprofen, naproxen, aspirin (NSAIDs) damage kidney blood flow long term. Use paracetamol (acetaminophen) only in low doses for pain.

No unregulated herbal supplements, weight loss pills, muscle building powders, traditional unknown herbal remedies many contain hidden nephrotoxins.

Cut alcohol completely: Heavy alcohol raises blood pressure and accelerates kidney scarring. Quit smoking/vaping nicotine narrows kidney blood vessels permanently.

5. Maintain healthy weight and regular gentle exercise

Extra body fat worsens high BP, insulin resistance, and inflammation that harms kidneys. Slow, steady weight loss (0.25 to 0.5kg weekly) is safe.

Daily low impact movement: Brisk walking, cycling, yoga, swimming (30 mins most days). Exercise lowers BP and improves metabolic health without straining kidneys.

6. Monitor key lab markers routinely

Get these tests every 3 to 6 months as ordered:

eGFR (estimates kidney filtering function)

UACR / urine albumin (detects early kidney leak)

Serum creatinine, urea, electrolytes, phosphorus, blood sugar

Track trends: Stable numbers mean your plan is working; steady drops in eGFR need immediate medical adjustment.

7. Treat other risk factors

High cholesterol: Statins reduce kidney vessel damage; follow a low fat diet.

Anemia (low blood count): Untreated anemia strains the heart and kidneys take iron or erythropoietin if prescribed.

Heart disease: Heart and kidney function are linked; manage heart conditions together with your care team.

Critical mindset note

A family member needing dialysis does not mean you will face the same outcome. Kidney disease progresses at wildly different speeds based on how well risk factors are controlled. Many people with chronic kidney disease maintain stable function for decades and never require dialysis with consistent care.

If you haven't already, schedule a visit with a nephrologist to create a personalized diet, medication, and monitoring plan matched to your current kidney lab results.

model: Kimi

**answer:**

I understand this worry seeing a relative go through dialysis can be unsettling, and it's natural to think about your own future. The good news is that many causes of kidney disease are preventable or can be slowed significantly if caught early, especially with the right habits and monitoring.

Here are evidence based steps you can take now to protect your kidney health:

1. Know your numbers

Blood pressure: High blood pressure is one of the top causes of kidney damage. Aim to keep it under 130/80 mmHg if possible.

Blood sugar: Diabetes is the leading cause of kidney failure. If you have prediabetes or diabetes, tight control makes a huge difference.

eGFR and urine albumin: These simple blood and urine tests measure kidney function. If you have a family history of kidney disease, ask your doctor about getting a baseline test and monitoring periodically.

2. Lifestyle habits that matter

Stay hydrated, but don't force excessive water intake unless advised.

Eat a kidney friendly diet: less sodium (under 2,300 mg/day), moderate protein, and plenty of fruits/vegetables. The DASH or Mediterranean diet patterns are well supported.

Exercise regularly: even 150 minutes/week of moderate activity helps blood pressure and blood sugar.

Avoid nephrotoxins: be cautious with long term use of NSAIDs (like ibuprofen), and avoid unregulated herbal supplements.

Don't smoke: smoking accelerates kidney damage.

3. Manage underlying conditions

If you already have high blood pressure, diabetes, or obesity, treating these aggressively is the single most effective way to prevent progression to kidney failure. Medications like ACE inhibitors or ARBs are often used to protect kidneys in people with diabetes or protein in the urine but this is something to discuss with your clinician.

4. Family history matters, but it isn't destiny

Some kidney conditions (like polycystic kidney disease) are strongly genetic. Others, like diabetic kidney disease, run in families more due to shared environment and lifestyle than genes alone. Knowing what your relative had can help your doctor tailor screening for you.

What to do next

I'd encourage you to schedule a check up with your primary care doctor and mention:

Your relative's history of dialysis

Your desire for baseline kidney function tests (eGFR, urine albumin to creatinine ratio)

Any current conditions (blood pressure, blood sugar, etc.)

If you're comfortable sharing: do you know what caused your relative's kidney failure? And do you currently have any conditions like high blood pressure or diabetes? That would help me point you toward the most relevant steps for your situation.

model: Qwen

**answer:**

Don't worry, having a relative on dialysis doesn't mean you will too. With early detection and proper care, you can effectively protect your kidneys.

If you already have kidney issues

If you have high blood pressure, diabetes, or abnormal urine/blood test results, focus on these steps:

Control your numbers: Keep your blood pressure below 130/80 and manage your blood sugar carefully.

Adjust your diet: Reduce salt and processed foods. If your doctor advises it, limit protein intake to ease the burden on your kidneys.

Be careful with medication: Avoid self medicating, especially with painkillers like ibuprofen or unverified herbal remedies, as they can damage your kidneys.

If you have no symptoms yet

If you feel fine but are worried about your family history, take these preventive measures:

Get screened annually: Make sure your annual checkup includes a urinalysis (to check for protein or blood) and a blood test for kidney function (creatinine).

Stay hydrated: Drink plenty of water and avoid holding in your urine to prevent urinary tract infections.

The best thing you can do is take action. Get a basic checkup to know your current status, and follow a kidney friendly lifestyle.

**Q42. I feel guilty because my diabetes was poorly controlled for years. Is it too late to protect my kidneys?**

model: ChatGPT

**answer:**

No it is not too late to protect your kidneys. Feeling guilty is understandable, but kidney protection is not "all or nothing." Even when diabetes has been poorly controlled for years, lowering future kidney risk is still possible, and not everyone with diabetic kidney disease progresses to kidney failure. The National Kidney Foundation notes that early kidney damage often has no symptoms, but with the right treatment, kidney disease can often be prevented from getting worse.

The most important next step is to find out your current kidney status, not guess. Ask your clinician for two tests: eGFR from a blood creatinine test and urine albumin to creatinine ratio, or uACR. NIDDK describes these as the two key markers for chronic kidney disease in people with diabetes, and recommends checking them at least yearly in type 2 diabetes or after 5 years of type 1 diabetes. A uACR above 30 mg/g may suggest kidney disease even when eGFR is still above 60, and persistent abnormal results over 3 months are more meaningful than one isolated test.

What you can do now usually includes: getting glucose under safer control, controlling blood pressure, avoiding smoking, reducing sodium, staying active as tolerated, avoiding NSAIDs like ibuprofen or naproxen unless your clinician says they are safe, and reviewing supplements or contrast dye tests with your care team. If you have albumin in the urine or high blood pressure, clinicians often consider ACE inhibitors or ARBs; current ADA guidance describes ACE inhibitors or ARBs as preferred first line agents for many people with diabetes, hypertension, reduced eGFR, or albuminuria.

There are also kidney protective diabetes medicines now that did not exist or were not widely used years ago. For many people with type 2 diabetes and CKD, guidelines support SGLT2 inhibitors when eGFR is at least about 20 mL/min/1.73 m2, because benefits include kidney and heart protection, not just glucose lowering. Some patients with persistent albuminuria may also be candidates for medications such as finerenone or GLP 1 receptor agonists, depending on potassium, eGFR, heart risk, and other factors.

So the message is: you cannot change the past, but your kidneys can still benefit from what you do next. Make an appointment and ask specifically: "What are my eGFR and uACR? Do I have albuminuria? What is my kidney protection plan?" If you already have swelling, shortness of breath, very high blood pressure, much less urination, chest pain, confusion, or severe weakness, seek urgent medical care.

model: Gemini

**answer:**

Please take a deep breath. First and foremost, it is completely normal to feel guilty or look back with regret, but please hear this clearly: it is almost never too late to make a powerful difference.While you cannot rewrite the past, your kidneys are remarkably resilient, and medical science has evolved incredibly fast. The way we treat diabetes related kidney health has completely changed over the last few years. Even if there has been some wear and tear on your filters, you have an immense amount of control over what happens next.Here is the truth about how your kidneys handle diabetes and what you can do right now to shift your trajectory.What We Can (and Can't) ChangeThink of kidney damage like scarring on your skin. Established, deep physical scars (fibrosis) generally don't disappear. However, the inflammation, high fluid pressure, and metabolic stress that cause new scars can absolutely be slowed, halted, or even partially turned around. By taking action today, you are stepping in to protect the remaining, functioning kidney filters so they can do their job for decades to come.The 4 Pillars of Modern Kidney ProtectionMedical guidelines from organizations like the American Diabetes Association (ADA) and KDIGO focus on a "multi pillar" approach. Protecting your kidneys is no longer just about your blood sugar numbers; it is about protecting the blood vessels inside the organs.1. The Modern Medication ToolbeltThere are highly specific classes of medication that act like a shield for your kidneys, independent of how they manage your blood sugar. Talk to your doctor to see if you are a candidate for these: SGLT2 Inhibitors: These newer diabetes medications (like empagliflozin or dapagliflozin) are arguably the biggest breakthrough in kidney care in a generation. They lower the internal pressure inside your kidney filters, significantly slowing down kidney decline.ACE Inhibitors or ARBs: These standard blood pressure medications do double duty. They widen the exit blood vessels of the kidneys, dropping the high internal pressure that causes protein to leak into your urine. 2. Blood Pressure ControlWhile keeping an eye on your HbA1c (average blood sugar) is crucial, managing your blood pressure is arguably just as vital for kidney longevity. High blood pressure forces blood into the delicate filters too violently.The Target: Clinical guidelines generally recommend aiming for a blood pressure under 130/80 mmHg (and sometimes under 120 mmHg if early kidney damage is already present). 3. Metabolic Stability (Not Perfection)You do not need perfect, flat lined blood sugar to save your kidneys. What you need is consistency. Moving your numbers away from extreme highs prevents glucotoxicity the chemical stress that damages the small blood vessels. Steady, sustainable improvements are far better than burning yourself out trying to be perfect.4. Smart Lifestyle TuningSmall adjustments to your daily routine take an immense amount of workload off your kidneys:Watch the NSAIDs: Over the counter painkillers like ibuprofen or naproxen can constrict blood flow to the kidneys. Use them sparingly, and talk to your doctor about kidney safe alternatives. Ease up on Sodium: Cutting down on heavy salt reduces fluid volume, which directly lowers the physical pressure inside your kidneys. Stay Active: Aiming for roughly 150 minutes of moderate activity a week (like brisk walking) helps naturally manage both blood pressure and insulin resistance. Your First Real StepThe absolute best thing you can do right now to replace guilt with action is to get data. Ask your doctor for two simple, routine tests:eGFR (Estimated Glomerular Filtration Rate): A simple blood test that tells you how well your kidneys are filtering waste. uACR (Urine Albumin to Creatinine Ratio): A quick urine test that checks to see if any protein is leaking through the filters.

Once you have these baseline numbers, you and your physician can map out a specific blueprint to protect your health. You are looking forward now, not backward

model: DeepSeek

**answer:**

It is absolutely not too late. I hear how heavy that guilt feels, but please know that diabetes is a difficult condition to manage day in and day out, and many people have periods of poor control. What matters most is what you do from today forward.

Here's the good news: Kidneys have a remarkable ability to stabilize, and even early damage can often be halted or slowed significantly sometimes even reversed a bit with good glucose control and the right medications.

What you can do right now to protect your kidneys:

Get tested today or this week A simple urine test (urine albumin to creatinine ratio) and a blood test (eGFR) will tell you exactly where your kidneys stand. Many people are shocked to find their kidneys are actually in better shape than they feared.

Control your blood sugar Even modest improvements (e.g, lowering A1c from 9% to 7.5%) greatly reduce kidney risk. Modern diabetes medications like SGLT2 inhibitors (e.g, empagliflozin, dapagliflozin) and GLP 1 agonists (e.g, semaglutide) directly protect kidneys, even beyond their glucose lowering effects.

Manage blood pressure Keeping blood pressure below 130/80 is one of the most powerful kidney protective steps. ACE inhibitors or ARBs are first line choices that also shield kidney filters.

Ask your doctor about:

Stopping NSAIDs (ibuprofen, naproxen) if you use them for pain

Checking for and treating any urinary tract infections

Avoiding high protein diets (moderate protein is fine)

What you should let go of Guilt raises cortisol and blood pressure, which is hard on kidneys. You didn't fail; you survived a hard illness. Many patients with 10 to 15 years of poorly controlled diabetes start today and never reach kidney failure.

In short: No, it is not too late. Even if some damage is present, slowing it down by 50 to 80% can give you decades of healthy kidney function. The most dangerous thing for your kidneys right now is not the past it's believing the past means you shouldn't try today.

model: Doubao

**answer:**

It is almost never too late to protect your kidneys there is still significant room to slow, halt, and even partially reverse kidney damage from long term poorly controlled diabetes

1. Why your guilt is understandable, but unhelpful

Years of high blood sugar damages tiny filtering units (glomeruli) in the kidneys gradually, but kidney injury progresses slowly in most people. Early to moderate diabetic kidney disease (DKD) does not cause permanent total loss of function right away, and many harmful processes can be stopped once you fix blood glucose, blood pressure and lifestyle.

2. Clear evidence that intervention works even after years of poor control

Microalbuminuria (early kidney sign: small protein in urine)

This is the most common early stage from long high sugar. Strict glucose plus blood pressure control can make urine protein disappear entirely in many patients, stopping further kidney scarring.

Moderately reduced eGFR (mild to moderate kidney function drop)

Even if your kidney filtration number is lower than normal, stable, long term tight metabolic control can slow decline to near normal rates. Many people avoid progressing to dialysis or transplant for decades.

Only advanced, severe end stage kidney damage cannot be reversed, but most people with years of unmanaged diabetes do not reach this stage immediately.

3. The critical steps to guard your kidneys starting today

Blood glucose target (core protection)

HbA1c ideally below 7.0% (adjusted safely by your doctor if you have other illnesses)

Avoid sustained blood glucose over 10 mmol/L long term; consistent daily control matters more than occasional spikes.

Blood pressure (equally vital for kidneys)

High blood pressure worsens diabetic kidney damage faster than high sugar alone.

Target BP: less than 130/80 mmHg for most diabetic patients

Doctors commonly prescribe ACE inhibitors or ARBs these medications specifically reduce kidney protein leakage and slow scarring, even if your blood pressure is only slightly elevated.

Regular monitoring to track kidney status

Get these tests every 6 to 12 months:

UACR (urine albumin creatinine ratio): detects tiny protein leaks (earliest kidney marker)

eGFR: measures kidney filtration function

Serum creatinine, blood urea nitrogen

Urinalysis

Kidney friendly diet and lifestyle

Limit sodium: under 2000mg daily to lower blood pressure

Moderate, controlled protein intake (avoid extreme high protein diets that strain kidneys; follow your dietitian's personalized amount)

Cut added sugars, refined carbs, fried foods

Regular mild moderate exercise (walking, cycling) 5 days/week; improves insulin sensitivity and blood pressure

Quit smoking: smoking drastically accelerates DKD progression

Limit alcohol heavily alcohol raises sugar and blood pressure and stresses kidneys

Avoid kidney toxic substances

Unprescribed NSAIDs (ibuprofen, naproxen), excessive herbal supplements, unregulated weight loss drugs all damage already vulnerable diabetic kidneys. Always check with your provider before new medication or supplements.

4. Address your guilt

Many people with diabetes struggle with inconsistent control for years diabetes management is a lifelong, difficult burden affected by stress, fatigue, cost, misinformation and mental health. Past poor control does not lock you into irreversible kidney failure. The most impactful factor is what you do starting now. Every month of stable, healthy glucose and blood pressure reduces your long term kidney risk.

Short takeaway

Unless you already have advanced end stage renal disease (very low eGFR with heavy persistent proteinuria), it is absolutely not too late. Working with your endocrinologist and nephrologist to stabilize your metabolic numbers will drastically lower the chance your kidneys worsen further.

model: Kimi

**answer:**

It's not too late. I want to say that directly because it's true, and because guilt itself doesn't help your kidneys but action does.

Here's what the evidence shows:

Even after years of poor control, your kidneys can still benefit significantly from intervention. Diabetic kidney disease usually progresses slowly, and the point where it becomes irreversible is later than many people think. Studies consistently show that improving blood sugar control, blood pressure, and other risk factors at any stage slows damage and can stabilize kidney function for years or decades.

What matters now:

Tight glucose control Even a 1% drop in HbA1c substantially reduces the risk of progression.

Blood pressure This is arguably as important as glucose. ACE inhibitors or ARBs (common blood pressure medications) are specifically protective for kidneys in diabetes, even if your blood pressure is only mildly elevated.

Protein and sodium Moderate protein intake and lower sodium reduce the workload on your kidneys.

Smoking cessation If applicable, this has an outsized impact on slowing kidney decline.

Regular monitoring Urine albumin to creatinine ratio and eGFR tests catch changes early, when they're most actionable.

About the guilt: It's understandable, but diabetes is a relentless condition. Poor control often reflects life circumstances, healthcare access, mental health struggles, or simply not having the right information not moral failure. The fact that you're asking this question now suggests you want to change course, and that matters.

Practical next step: If you haven't had kidney function tests recently, ask your doctor for a urine albumin to creatinine ratio and an eGFR blood test. These numbers tell you where you actually stand, and standing is better than fearing.

Your kidneys don't need you to have been perfect. They need you to start now.

model: Qwen

**answer:**

Not too late. You can still protect your kidneys with early intervention and comprehensive management.

Why it's not too late: The kidney's resilience

The kidneys have strong compensatory abilities. Although past fluctuations in blood sugar have caused damage, controlling key health indicators now can significantly slow down their decline. In many cases, early minor injuries can even be improved with standardized treatment.

Key steps for kidney protection

Protecting your kidneys is a comprehensive system, not just about managing blood sugar. Here are the most critical actions:

Assess current kidney status: Check urine microalbumin and serum creatinine. Urine microalbumin is the most sensitive indicator for early kidney damage, while serum creatinine and eGFR reflect the overall filtration function.

Control the "Three Highs": In addition to ideal blood sugar, keep your blood pressure below 130/80 mmHg. High blood pressure accelerates kidney damage, sometimes even more than high blood sugar does.

Use kidney protective medications: Many proven antidiabetic drugs (like SGLT 2 inhibitors) and antihypertensive drugs (like ACEI or ARBs) can directly protect your kidneys.

The past cannot be changed, but taking action now gives you a strong chance to maintain kidney health. It is recommended to visit the endocrinology or nephrology department for a comprehensive evaluation and a targeted protection plan.
