# Supplementary material for: Safety, accuracy, empathy and information quality of six large language models answering patient-facing questions about diabetic kidney disease: a cross-sectional comparative study
Source: Front Med (Lausanne). 2026 Oct 30;13:2007153. doi: 10.3389/fmed.2026.2007153 (PMC13759355; doi:10.3389/fmed.2026.2007153)
Supplement: Supplementary file 6 [file Table6.docx]

**Supplementary Material 6.**

**Professional Characteristics and Expertise of the Five Independent Raters**

| **Rater ID** | **Professional background** | **Academic qualification / academic role** | **Professional title and years of experience** | **Main expertise relevant to this study** |
| --- | --- | --- | --- | --- |
| Rater 1 | Nephrology physician at a tertiary A hospital; diabetic kidney disease (DKD) expert | Master's supervisor | Associate Chief physician; 16 years of clinical experience | Nephrology research and DKD diagnosis and treatment; CKD complication management |
| Rater 2 | Nephrology physician specializing in chronic kidney disease (CKD)/DKD at a tertiary A hospital | Master's degree; Master's supervisor | Associate Chief physician; 13 years of clinical experience | Nephrology research and DKD diagnosis and treatment; CKD complication management |
| Rater 3 | Endocrinology physician and diabetes management expert at a tertiary A hospital | Master's degree | Associate chief physician; 14 years of clinical experience | Diabetes research and glycemic management; individualized glucose-lowering therapy, glucose monitoring, hypoglycemia risk management, and medication selection in patients with DKD |
| Rater 4 | Diabetes specialist nurse and patient education expert at a tertiary A hospital | Doctoral degree | Associate chief nurse; 10years of clinical experience | Diabetes/DKD patient education and self-management; diet, exercise, glucose monitoring, medication adherence, and health literacy assessment |
| Rater 5 | Diabetes specialist nurse and patient education expert at a tertiary A hospital | Bachelor’s degree in nursing | Chief Nurse; 32 years of clinical experience | Diabetes/DKD patient education and self-management; diet, exercise, glucose monitoring, medication adherence, and health literacy assessment |

Note:In this table, Chief Nurse and Associate Chief physician refer to senior professional titles rather than administrative positions.
