# Supplementary material for: Safety, accuracy, empathy and information quality of six large language models answering patient-facing questions about diabetic kidney disease: a cross-sectional comparative study
Source: Front Med (Lausanne). 2026 Oct 30;13:2007153. doi: 10.3389/fmed.2026.2007153 (PMC13759355; doi:10.3389/fmed.2026.2007153)
Supplement: Supplementary file 8 [file Table8.docx]

**Supplementary material 8**

## Table 1. Pairwise comparisons of accuracy scores across the six models

| **Comparison** | **N pairs** | **Mean difference** | **Mean difference 95% CI** | **Median difference** | **Median difference 95% CI** | **Rank-biserial correlation** | **Rank-biserial 95% CI** | **Adjusted *P*** |
| --- | --- | --- | --- | --- | --- | --- | --- | --- |
| ChatGPT vs Gemini | 42 | 0.75 | 0.65-0.84 | 0.80 | 0.60-1.00 | 1.000 | 1.000-1.000 | <0.001 |
| ChatGPT vs DeepSeek | 42 | 0.46 | 0.33-0.60 | 0.40 | 0.40-0.70 | 0.813 | 0.597-0.968 | <0.001 |
| ChatGPT vs Doubao | 42 | 1.20 | 1.06-1.34 | 1.20 | 1.00-1.40 | 1.000 | 1.000-1.000 | <0.001 |
| ChatGPT vs Kimi | 42 | 0.95 | 0.81-1.08 | 1.00 | 0.80-1.20 | 1.000 | 1.000-1.000 | <0.001 |
| ChatGPT vs Qwen | 42 | 0.71 | 0.61-0.80 | 0.80 | 0.60-1.00 | 1.000 | 1.000-1.000 | <0.001 |
| Gemini vs DeepSeek | 42 | -0.29 | -0.38 to -0.19 | -0.20 | -0.40 to -0.00 | -0.937 | -1.000 to -0.775 | <0.001 |
| Gemini vs Doubao | 42 | 0.45 | 0.33-0.58 | 0.40 | 0.20-0.60 | 0.961 | 0.867-1.000 | <0.001 |
| Gemini vs Kimi | 42 | 0.20 | 0.13-0.28 | 0.20 | 0.00-0.40 | 0.872 | 0.668-0.980 | <0.001 |
| DeepSeek vs Doubao | 42 | 0.74 | 0.60-0.86 | 0.80 | 0.60-1.00 | 1.000 | 1.000-1.000 | <0.001 |
| DeepSeek vs Kimi | 42 | 0.49 | 0.40-0.58 | 0.40 | 0.40-0.60 | 1.000 | 1.000-1.000 | <0.001 |
| DeepSeek vs Qwen | 42 | 0.25 | 0.16-0.34 | 0.20 | 0.00-0.40 | 0.926 | 0.778-1.000 | <0.001 |
| Doubao vs Kimi | 42 | -0.25 | -0.36 to -0.14 | -0.20 | -0.40 to -0.00 | -0.745 | -0.933 to -0.472 | <0.001 |
| Doubao vs Qwen | 42 | -0.49 | -0.61 to -0.37 | -0.40 | -0.70 to -0.20 | -1.000 | -1.000 to -1.000 | <0.001 |
| Kimi vs Qwen | 42 | -0.24 | -0.31 to -0.17 | -0.20 | -0.40 to -0.00 | -1.000 | -1.000 to -1.000 | <0.001 |
| Gemini vs Qwen | 42 | -0.04 | -0.08 to -0.00 | 0.00 | 0.00-0.00 | -0.495 | -1.000 to -0.028 | 0.084 |

Note: Pairwise comparisons were performed using paired Wilcoxon signed-rank tests with Benjamini-Hochberg correction. Paired mean/median differences and rank-biserial correlations are reported with bootstrap 95% CIs.
